# *CD86* Is Associated with Immune Infiltration and Immunotherapy Signatures in AML and Promotes Its Progression

**DOI:** 10.1155/2023/9988405

**Published:** 2023-04-07

**Authors:** Qianqian Zhang, Ruixue Ma, Huimin Chen, Wentong Guo, Zhenyu Li, Kailin Xu, Wei Chen

**Affiliations:** ^1^Department of Hematology, The Affiliated Hospital of Xuzhou Medical University, Xuzhou, Jiangsu, China; ^2^Blood Diseases Institute, Xuzhou Medical University, Xuzhou, Jiangsu, China; ^3^Jiangsu Key Laboratory of Bone Marrow Stem Cells, Xuzhou, Jiangsu, China; ^4^Department of Hematology, The First People's Hospital of Suqian, Suqian, Jiangsu, China

## Abstract

**Background:**

Cluster of differentiation 86 (*CD86*), also known as B7-2, is a molecule expressed on antigen-presenting cells that provides the costimulatory signals required for T cell activation and survival. *CD86* binds to two ligands on the surface of T cells: the antigen *CD28* and cytotoxic T lymphocyte-associated protein 4 (CTLA-4). By binding to *CD28*, *CD86*—together with *CD80*—promotes the participation of T cells in the antigen presentation process. However, the interrelationships among *CD86*, immunotherapy, and immune infiltration in acute myeloid leukemia (AML) are unclear.

**Methods:**

The immunological effects of *CD86* in various cancers (including on chemokines, immunostimulators, MHC, and receptors) were evaluated through a pan-cancer analysis using TCGA and GEO databases. The relationship between *CD86* expression and mononucleotide variation, gene copy number variation, methylation, immune checkpoint blockers (ICBs), and T-cell inflammation score in AML was subsequently examined. ESTIMATE and limma packages were used to identify genes at the intersection of *CD86* with StromalScore and ImmuneScore. Subsequently, GO/KEGG and PPI network analyses were performed. The immune risk score (IRS) model was constructed, and the validation set was used for verification. The predictive value was compared with the TIDE score.

**Results:**

*CD86* was overexpressed in many cancers, and its overexpression was associated with a poor prognosis. *CD86* expression was positively correlated with the expression of *CTLA4*, *PDCD1LG2*, *IDO1*, *HAVCR2*, and other genes and negatively correlated with *CD86* methylation. The expression of *CD86* in AML cell lines was detected by QRT-PCR and Western blot, and the results showed that *CD86* was overexpressed in AML cell lines. Immune infiltration assays showed that *CD86* expression was positively correlated with CD8 T cell, Dendritic cell, macrophage, NK cell, and Th1_cell and also with immune examination site, immune regulation, immunotherapy response, and TIICs. ssGSEA showed that *CD86* was enriched in immune-related pathways, and *CD86* expression was correlated with mutations in the genes *RB1*, *ERBB2*, and *FANCC*, which are associated with responses to radiotherapy and chemotherapy. The IRS score performed better than the TIDE website score.

**Conclusion:**

*CD86* appears to participate in immune invasion in AML and is an important player in the tumor microenvironment in this malignancy. At the same time, the IRS score developed by us has a good effect and may provide some support for the diagnosis of AML. Thus, *CD86* may serve as a potential target for AML immunotherapy.

## 1. Introduction

Acute myeloid leukemia (AML) is a common hematological disease characterized by the clonal proliferation, abnormal differentiation, and cell death evasion of bone-marrow-derived hematopoietic stem and progenitor cells [1]. These cells proliferate in the peripheral blood and infiltrate the bone marrow. The tumor microenvironment in AML is characterized by immunosuppression, which promotes immune tolerance and the immune escape of malignant cells [2]. The main components of the AML bone marrow microenvironment (BMM) include T cells, B cells, and NK cells [3]. The immune imbalance of T helper cells (Th cells) is a major contributor to the sudden progression of AML [4].

T-cell-mediated cellular immunity is primarily achieved by the specific binding of antigen peptides to the major histocompatibility complex (MHC) (first signal) and the binding of costimulatory molecules located on the surface of antigen-presenting cells (APCs) to their receptors (second signal) [5]. The absence of costimulatory molecules leads to immune unresponsiveness, which promotes tumor escape in AML. Owing to advancements in research, immunotherapies that retarget effector cells (T cells, NK cells) have been developed, and these have become the key for AML treatment [6]. Typically, tumors suppress the immune system, resulting in the impairment of T-cell function. The goal of immunotherapy is to eliminate this impairment. Studies have shown that vaccines for AML/dendritic cell fusion can amplify T-cell populations and prevent AML recurrence [7]. Therefore, effective immunotherapy approaches that target specific proteins are the key to AML treatment.

B7-2, better known as *CD86*, is a member of the B7 family [8]. *CD28* and cytotoxic T lymphocyte antigen 4 (CTL1-4) are regulated. *CD86* can bind to *CD28*, leading to signal production and the recognition of antigenic peptides by T-cell receptors (TCRs), which leads to T-cell proliferation and IL-2 production [9]. *CD86* has been reported to be overexpressed in samples from AML patients [10]. *CD86* is a marker for monocytes and dendritic cells and is involved in the progression of AML [11]. Improvements in sequencing technology have promoted extensive research on molecular networks using gene sequencing data from public databases [12]. However, the correlation between *CD86* and immunomodulators (chemokines, receptors, and MHC proteins), immunotherapy results, and immune checkpoint proteins in AML has not been reported. Therefore, it is very important to explore the associations among *CD86*-related molecules, immune infiltration, and immunotherapy.

## 2. Methods

### 2.1. Data

The Cancer Genome Atlas (TCGA) data: Pan-carcinoma (33 species) RNA sequencing (RNA-SEQ) data (FPKM values) were downloaded from the UCSC Xena data portal (https://xenabrowser.net/). They were converted to TPM format, and somatic mutation data and survival information were downloaded. Log2 transformation was performed on the RNA-SEQ data, and somatic mutation data were analyzed using MuTect. Copy number variation (CNV) data processed using GISTIC were downloaded from the UCSC Xena data portal. Further, the methylation data were downloaded from the LinkedOmics data portal.

Then, information was obtained from the GEO database (https://www.ncbi.nlm.nih.gov/geo/) LAML GEO queue, which contains detailed survival data. The information including data from the GSE10358, GSE37642 (including data from the GPL570 and GPL96 platforms), GSE146173, GSE106291, and GSE12417 databases (including data from the GPL97 and GPL96 platforms). The sample data for leukemia was retained. Further, three GEO databases containing information on responses to immunotherapy were downloaded: GSE78220 (melanoma), GSE135222 (non-small-cell lung cancer), and GSE91061 (melanoma). Moreover, complete expression data and detailed clinical information of patients from the IMvigor210 study (AML immunotherapy-related data) were obtained from https://research-pub.Gene.com/imvigor210corebiologies/under the 3.0 license.

### 2.2. Differential Gene Analysis

The R limma package was used to filter out immune-related genes (IRGs) (https://Bioconductor.org/packages/limma/). The differentially expressed genes (DEGs) were identified based on a cutoff value of false detection rate (FDR) < 0.05 and Log2 |fold change| > 1. The differentially expressed IRGs were then extracted from the list of all DEGs.

### 2.3. Analysis of Immunological Characteristics of AML

First, using the web portal TISIDB (https://cis.hku.hk/TISIDB) [13], genes related to the immune response, including those encoding immune stimulants, MHC proteins, immune receptors, and chemokines, were identified. ‘ggplot2' in R software was used for visualization, and the R package ‘Corrlot' was used to calculate the Spearman correlation coefficients between the expression of *CD86* and that of the abovementioned genes. In order to calculate the correlation between *CD86* expression and that of various oncogenes expressed in the tumor microenvironment, single-sample gene enrichment analysis (ssGSEA) was performed and the correlation between *CD86* and immune cell scores was calculated. The association of *CD86* with immune risk scores (IRSs) and the inflammatory coefficient of T cells was calculated using the generalized T-cell inflammation score formula [14].

Then R package ‘limma' was used to analyze the differences in the expression of chemokines, immunostimulators, MHC proteins, and immune receptors based on high vs. low *CD86* expression. CIBERSORT, MCPcounter, TIMER, Quantiseq, and Xcell were used to examine the immune-infiltrating cells in AML. The correlation between *CD86* and common immune checkpoint blockers (ICBs) was calculated. Further, StromalScore and ImmuneScore were calculated for AML samples using the R package ‘ESTIMATE.' ‘Limma was used to identify the DEGs in the high vs. low *CD86* expression, StromalScore, and ImmuneScore groups. Then, ‘ggplot2' was used to draw volcano maps and heat maps of the DEGs. A total of 308 up-regulated genes and 16 down-regulated genes were identified through this analysis.

### 2.4. Immune Risk Score (IRS) Calculation

IRSs were calculated based on the time of patient enrollment. The 324 DEGs were randomly sampled from TCGA to establish the training and validation sets at a 1 : 1 ratio. The R package ‘SurvMiner' was used to conduct univariate Cox regression analysis for the DEGs, and the optimal characteristic genes were identified according to the Least Absolute Shrinkage and Selection Operator (LASSO) method. Multivariate Cox regression analysis was performed, and based on the median IRS, the sample was divided into groups. The Kaplan–Meier method was used to compare survival outcomes between these groups. Univariate Cox analysis was used to screen PRGs with a prognostic value. The *P* value threshold for significance was set at 0.05, and 17 survival-related genes were selected for further analysis. LASSO-penalized Cox regression analysis and GLMNET R software package were used to establish a prognostic model to reduce overfitting. Finally, six genes and their coefficients were retained to determine the penalty parameter (*λ*) with the minimum criterion. The risk score was obtained using the formula IRS = ∑_*i*=1_^*n*^*β∗xi*, where *β* = the regression coefficient. AML patients were divided into two groups: high-risk group and low-risk group. The ‘SurvMiner' R software package was used to compare survival status between the two risk groups, and ‘Survival' and ‘timeROC R software packages were used for receiver operating characteristic (ROC) curve analysis. In addition, univariate and multivariate Cox regressions were used to determine the independent prognostic value of the three genes. To verify the validity of the model, analyses were performed using data from the GEO internal test queue or ICGC external validation queue. Median risk scores were obtained using the GEO training cohort, whereas patients in the GEO test cohort were divided into low- and high-risk groups.

### 2.5. GO, KEGG and PPI Analysis

Based on *CD86* expression, StromalScore, and ImmuneScore, the patients were divided into two groups. Using the limma package and subsequent filtering based on a |Log2FC| ≥ 1 and FDR < 0.05, DEGs were identified in the high vs. low *CD86* expression, StromalScore, and ImmuneScore groups. The ‘cluster analyzer' R package was used for Gene Ontology (GO) and Kyoto Encyclopedia of Genes and Genomes (KEGG) analysis. Normalized *P* values < 0.05 and an FDR *q* < 0.05 were considered statistically significant.

PPI analysis was performed for the 324 DERs using STRING, Cytoscape was used for visualization, and the MCODE plug-in was used to identify critical clusters.

### 2.6. Cell Culture

Myelodysplastic SKM-1 cells and human myeloid leukemia OCI-AML2, SH-1, KU812, MEG01, and K562 cells were purchased from the Shanghai Cell Bank, Chinese Academy of Sciences. SKM-1 cells were cultured in DMEM high-glucose medium (Gibco), and OCI-AML2, SH-1, HL-60, MEG01, and K562 cells were cultured in IMDM medium (Gibco). All media were supplemented with 10% fetal bovine serum (Biological Industries) and 1% cyanin-streptomycin (Biosharp). All cells were cultured at 37.5°C in a 5% CO_2_ incubator.

### 2.7. Total RNA Extraction and RT-PCR

The TRIzol reagent (Invitrogen) was used to extract total RNA from cells after treatment. The Prime Script RT Master Mix kit (TaKaRa) was used to reverse transcribe the extracted RNA into cDNA. Subsequent RT-PCR was performed based on the manufacturer's instructions of the amplification kit. PCR primers: *CD86*: R: 5′-CTGCTCATCTATACACGGTTACC-3′; F: 5′-GGAAACGTCGTACAGTTCTGTG-3′; GAPDH : R: 5′-AGAAGGCTGGGGCTCATTTG-3′, F: 5′-AGGGGCCATCCACAGTCTTC-3′.

### 2.8. Western Blot Assay

After cell digestion and centrifugation, RIPA lysis buffer (Beyotime Biotech) was added, and the cells were lysed on ice for 30 min. Then, cells were centrifuged at 12000 rpm for 30 min. The supernatant was removed, and protein levels were quantified using the BCA kit (Beyotime Biotech). The proteins were separated using SDS-PAGE and electrotransferred to PVDF membranes. The *CD86* primary antibody (Proteintech) was incubated overnight at 4°C after 2 hours of rapid blocking solution (BSA; Beyotime Biotech). On the following day, the corresponding secondary antibody was added. Protein bands were detected using the ECL exposure solution.

### 2.9. Statistical Analysis

Data were plotted using R package (V 4.0.0). The *T* test and *U*test were used to compare variables between two groups. Categorical variables were evaluated using the Chi-square test. Pearson and Spearman coefficients were used for correlation analysis. The Kaplan–Meier method was used to plot survival outcomes, and the logarithmic rank sum test was used to analyze statistical differences. *P* < 0.05 was considered statistically significant.

## 3. Results

### 3.1. *CD86* Is Overexpressed in Many Cancers and Is Associated with the Prognosis and Immune Response of AML

Using TCGA data on the expression profiles of 33 cancers, *CD86* expression was examined. The findings showed that *CD86* was highly expressed in most of the cancers, such as breast cancer, cholangiocarcinoma, colorectal cancer, esophageal cancer, glioma, renal clear cell carcinoma, renal papillary cell carcinoma, lung adenocarcinoma, lung squamous cell carcinoma, pancreatic cancer, rectal adenocarcinoma, gastric cancer, thyroid cancer, and endometrial cancer (Supplementary Figure 1). *CD86* was also overexpressed in AML (Supplementary Figure 1). Then, based on the median expression value of *CD86*, patients were divided into high- and low-expression groups.

**Figure 1:**
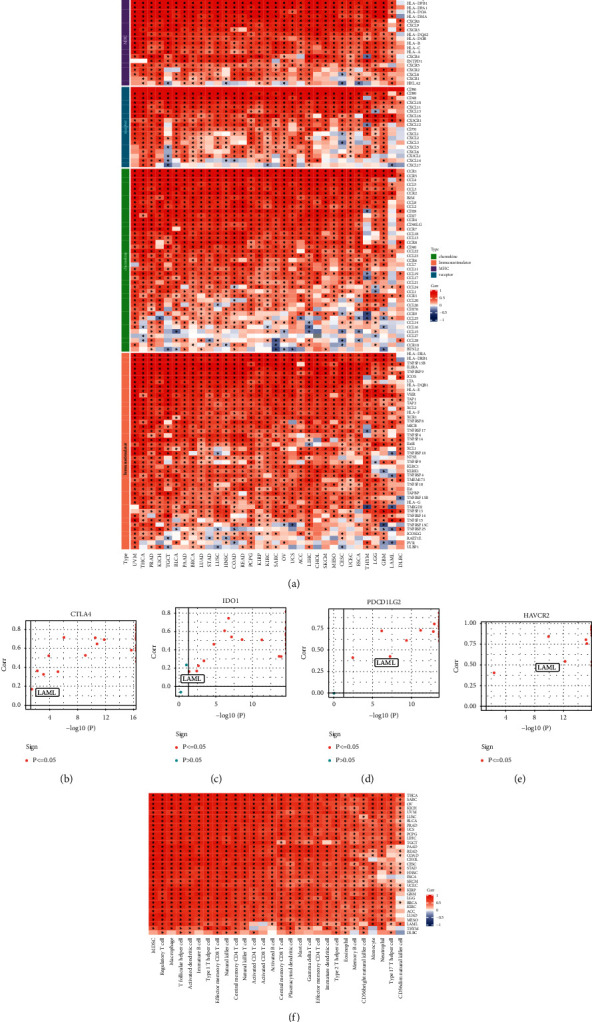
*CD86* was associated with immunoassay sites in pan-cancers. (a) Correlation between *CD86* and immunomodulators (chemokines, receptors, MHC, and immunostimulators). (b–e) Correlation between *CD86* and four immune checkpoints, PDCD1, CTLA4, CD274, and LAG3. The dots represent cancer types. The *Y*-axis represents the Pearson correlation, while the *X*-axis represents –log10P. (f) Correlation between diffuse carcinoma and 28 tumor-associated immune cells calculated with the ssGSEA algorithm. The color indicates visual cues the correlation coefficient (red is positive, blue is negative). The asterisks indicate a statistically significant *P* value calculated using Spearman correlation analysis. (^*∗*^*P* < 0.05).

Kaplan–Meier analysis was performed to examine high vs. low *CD86* expression in various cancers using TCGA data, and log-rank tests were used for survival analyses. The results showed that low *CD86* expression was associated with bladder urothelial carcinoma, cervical squamous cell carcinoma, and endocervical adenocarcinoma. In AML, low expression of *CD86* had a statistically significant better prognosis (Supplementary Figure 2). The results of univariate Cox regression analysis were then used to create a forest map, which showed that *CD86* expression was statistically significant in various cancers (Supplementary Figure 3A). Subsequently, using the TISIDB website established by Ru et al., four gene sets—chemokines, immunostimulators, MHC proteins, and receptors—were downloaded (Supplementary Table 1). Spearman correlation coefficients were used to analyze the association between *CD86* and these four gene sets in different cancer types (Figure 1(a)). Subsequently, the correlation between key pan-carcinoma molecules (including CTLA4, PDCD1LG2, IDO1, and HAVCR2) and *CD86* was calculated. These genes were found to be positively correlated with *CD86* in AML (Figures 1(b)–1(d)). ssGSEA method was then used to evaluate the scores of 28 immune cell types in different cancer types, and then calculated the correlation between *CD86* and them. The results showed that *CD86* expression was positively correlated with 28 types of immune cells (Figure 1(f)).

### 3.2. Single Nucleotide Variation (SNV), Gene Copy Number Variation (CNV), and Methylation Analysis of *CD86* in AML

**Figure 2:**
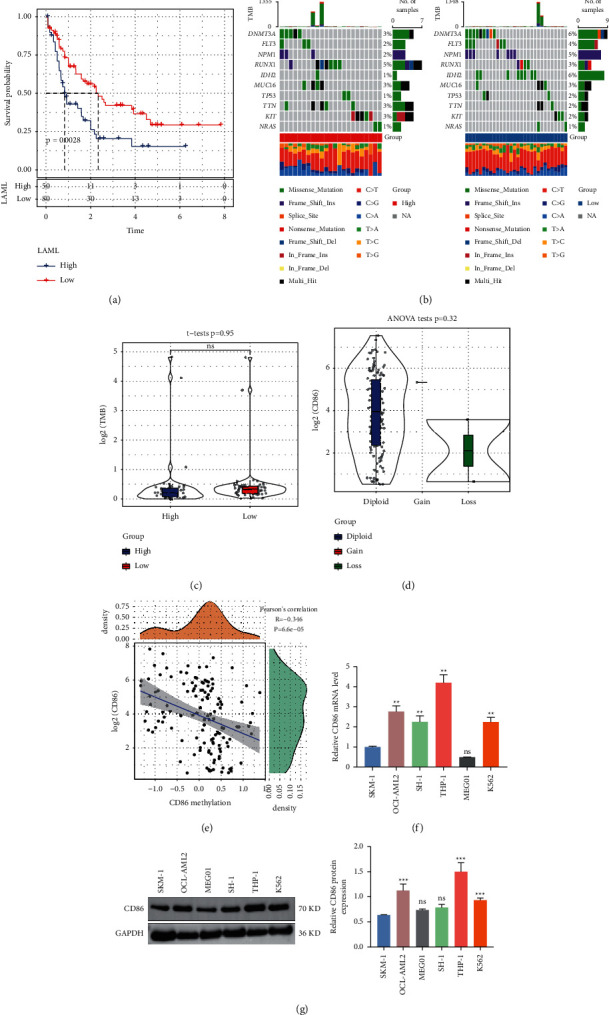
SNV, CNV, and methylation analysis of *CD86* in AML. (a) KM curve with *CD86* as cutoff in LAML; (b) mutation distribution of the top 10 genes with the highest mutation frequency in the median group of *CD86* expression; (c) comparison of TMB distribution of *CD86* expression median group; (d) *CD86* gene expression difference among *CD86* gene amplification groups; (e) correlation analysis between expression of *CD86* gene and methylation. (f) The mRNA expression of *CD86* in AML cell lines was detected by QRT-PCR. (g) Western blot was used to detect the expression of *CD86* in AML cell lines.

Site mutations are a key pathogenic factor causing abnormal proliferation in AML. To investigate whether *CD86* is mutated in AML, SNV, and gene CNV data for AML were analyzed. The results showed that *CD86* was not mutated in AML. The AML samples were divided into two groups according to a *CD86*-expression-based cutoff. The group with high *CD86* expression had a higher risk, indicating that the high *CD86* level was a risk factor for leukemia (Figure 2(a)). Then, the 10 genes with the highest mutation frequencies in the high- vs. low-expression groups were plotted. Accordingly, we found that *DNMT3A*, *FLT3*, *NPM1*, *IDH2*, and other genes had a relatively high mutation frequency in the low expression group (Figure 2(b)). Differences in tumor mutation load (TMB) were examined in the *CD86* high- vs. low-expression groups, but the results revealed no significant differences (Figure 2(c)). The amplification and deletion of *CD86* was examined. However, most samples showed no copy number changes in the *CD86* gene (Figure 2(d)). The expression of the *CD86* gene was compared across different groups. Meanwhile, the correlation between the expression of *CD86* and the degree of methylation was calculated and plotted. *CD86* expression showed a significant negative correlation with *CD86* methylation (Figure 2(e)). All previous experiments were conducted using public databases. To validate whether *CD86* is associated with AML, we examined *CD86* expression in vitro. QRT-PCR and Western blot were used to detect *CD86* expression in SKM-1 (myelodysplastic syndrome), OCI-AML2 (human myeloid leukemia cell), SH-1 (human myeloid leukemia cell), HL-60 (human myeloid leukemia cell), MEG01 (human megakaryoblastic leukemia cell), and K562 cells (human myeloid leukemia cell). The results showed that *CD86* was overexpressed in OCI-AML2, THP-1, SH-1, and K652 cells (Figures 2(f) and 2(g)). These results demonstrated that while *CD86* was not mutated in AML and was not related to the TMB, the degree of *CD86* methylation decreased with an increase in *CD86* expression.

### 3.3. Immune Status of *CD86* High- vs. Low-Expression Groups in AML

**Figure 3:**
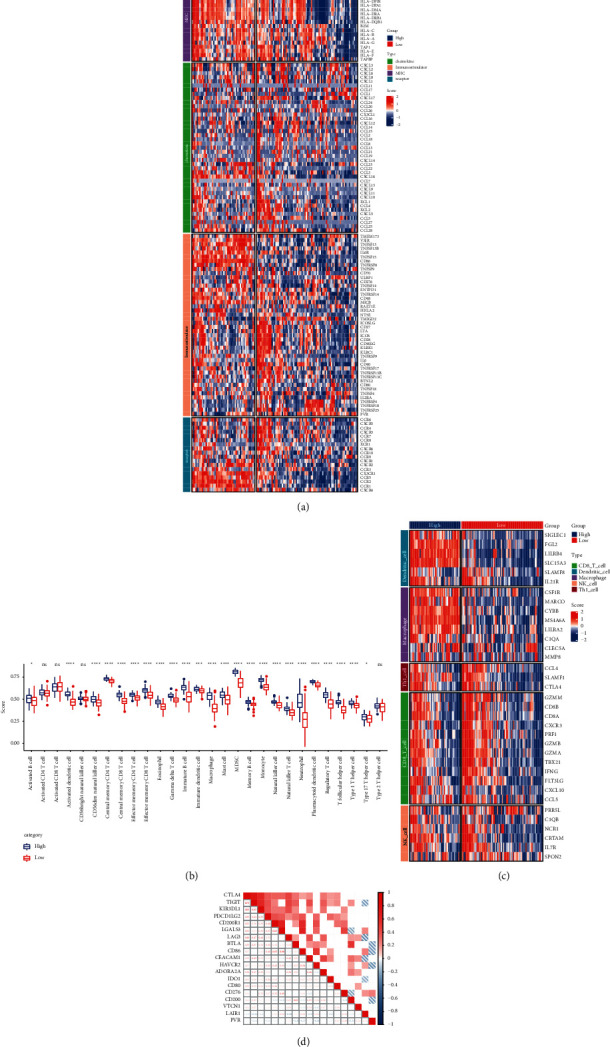
*CD86* was correlated with immunoassay sites. (a) Differences in expression of immunomodulators (chemokines, receptors, MHC, and immune stimulants) in LAML between the high and low *CD86* groups; (b) differences in immune cell scores between high *CD86* and low *CD86* groups; (c) effector gene differences in immune cells associated with 5 TIICs (CD8 + T cells, NK cells, macrophages, Th1 cells, and dendritic cells) between the high and low *CD86* groups; (d) correlation between *CD86* and immune checkpoints. The colors and values represent Spearman correlation coefficients. (^*∗*^*P* < 0.05; ^*∗∗*^*P* < 0.01; ^*∗∗∗*^*P* < 0.001; ^*∗∗∗∗*^*P* < 0.0001; blank, *P* > 0.05).

To further understand the association between *CD86* expression and immunoassay sites in AML, the differences in chemokine, immunostimulator, MHC protein, and immune receptor expression were compared between the high vs. low *CD86* expression groups (Supplementary Table 2). A heat map was drawn to represent the DEGs (Figure 3(a)). The distribution of 28 types of immune cells in the high vs. low *CD86* expression groups was analyzed. The results showed that for 24 types of immune cells, the group with the high expression of *CD86* had a higher immune score (Figure 3(b)). To further understand the correlation between *CD86* expression and tumor-infiltrating immune cells (TIICs) in AML, CIBERSORT, MCPcounter, TIMER, Quantiseq, and Xcell were used. Immune infiltration analysis was performed, and correlation between *CD86* expression and immune scores was calculated. Further, given that CD8+ T cell recruitment and dendritic cell, macrophage, NK cell, and Th1 activation are required during the migration of immune cells to tumors, the marker genes of these cell types were analyzed in the *CD86* high- vs. low-expression groups (Supplementary Table 3). The heat map is shown in Figure 3(c). In addition, the correlation between *CD86* and immune checkpoints was calculated. The results indicated that *CD86* was positively correlated with these aforementioned immunoassay sites (Figure 3(d)).

### 3.4. *CD86* Is Associated with Immune Checkpoint Blockers (ICBs) in AML

**Figure 4:**
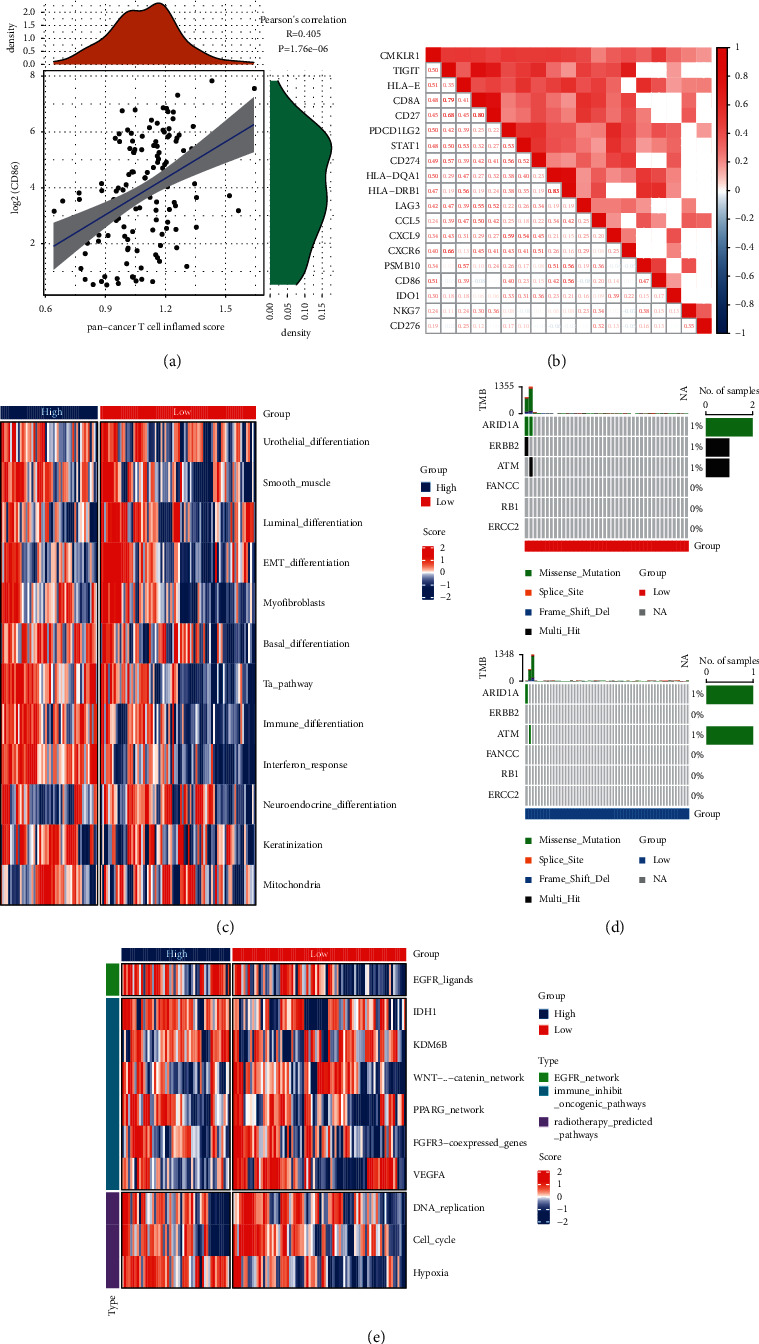
*CD86* predicts progression of immune checkpoint blockades (ICBs) in LAML. (a)–(b) Correlations between *CD86* and the pan-cancer T-cell inflamed score and the individual genes included in the T-cell inflamed signature. The T-cell inflamed score is positively correlated with the clinical response to cancer immunotherapy; (c) correlations between *CD86* and molecular subtypes using seven different algorithms and AML signatures; (d) mutational profiles of neoadjuvant chemotherapy-related genes in low- and high-*CD86* groups. (e) correlations between *CD86* and the enrichment scores of several therapeutic signatures such as targeted therapy and radiotherapy.

Immune checkpoint blockers (ICBs) are the key to enhancing the body's endogenous anti-tumor immune effect. It is critical to find markers that predict the clinical efficacy of ICBs. The correlation between *CD86* expression and the inflammatory scores of ubiquitous T cells were examined. Interestingly, a significant positive correlation was identified (Figure 4(a)). This suggested that high *CD86* expression promotes the inflammatory response. In addition, the correlation between *CD86* and the immune characteristics of different ICB response subgroups, including immune regulators, tumor-infiltrating immune cell-effector genes, immune checkpoints, and immunotherapy-related genes was examined. *CD86* was also found to be positively correlated with these factors (Figure 4(b)). Subsequently, ssGSEA was used to evaluate the scores for tumor and immune-related pathways, including Immune_differentiation and Interferon_response. The results showed that high *CD86* expression was present in these pathways (Figure 4(c)). Molecular subtypes also have a great impact on adjuvant chemotherapy. Subsequently, based on a literature survey [15–17], we compared the mutations in *RB1*, *ERBB2*, *FANCC*, and other genes that could be associated with chemoradiotherapy responses. Accordingly, different mutation frequencies were observed in the high and low *CD86* groups (Figure 4(d)). Common pathways of tumor growth (EGFR_network, Immune_inhibit_Oncogenic_pathways, and Radiotherapy_predicted_pathways) were compared between the high- and low-*CD86*-expression groups (Figure 4(e)). The results suggested that *CD86* is associated with different subtypes of ICB, and that the high expression of *CD86* can result in higher mutation frequencies in chemoradiation-related genes.

### 3.5. Identification of Immune-Associated Differential Genes (DERs), Protein–Protein Interaction (PPI) Network, and KEGG/GO Analysis

**Figure 5:**
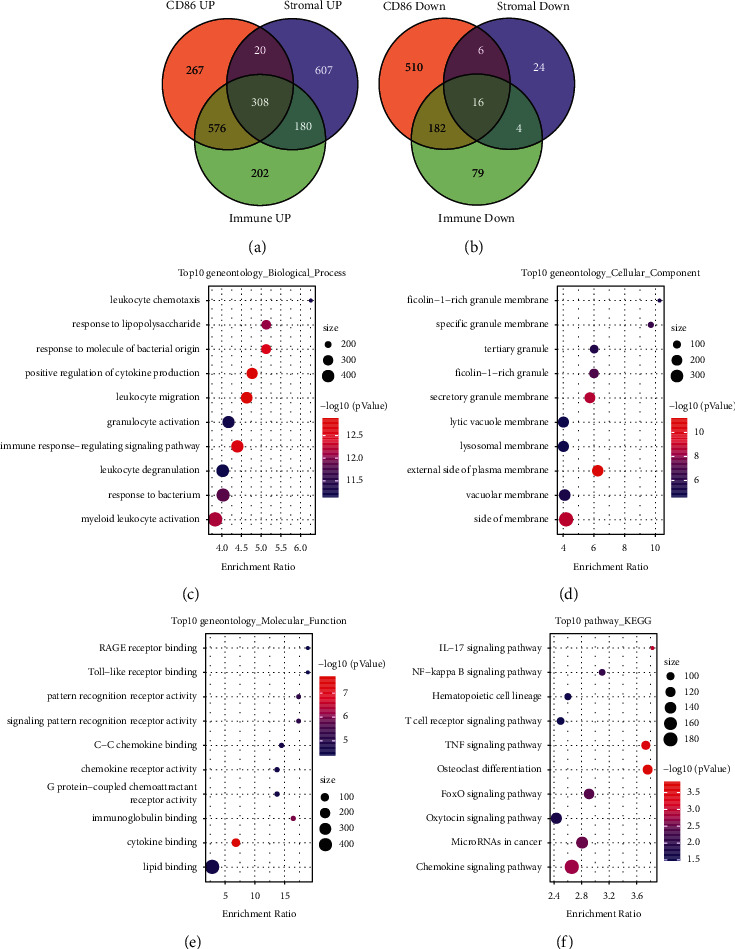
*CD86*, StromalScore, and ImmuneScore differential gene screening and PPI analysis. (a) Intersection of up-regulated genes in *CD86*, StromalScore, and ImmuneScore; (b) intersection of down-regulated genes in *CD86*, StromalScore, and ImmuneScore; (c)–(f) GO and KEGG functional enrichment analysis of differentially expressed genes in *CD86*, StromalScore, and ImmuneScore.

**Figure 6:**
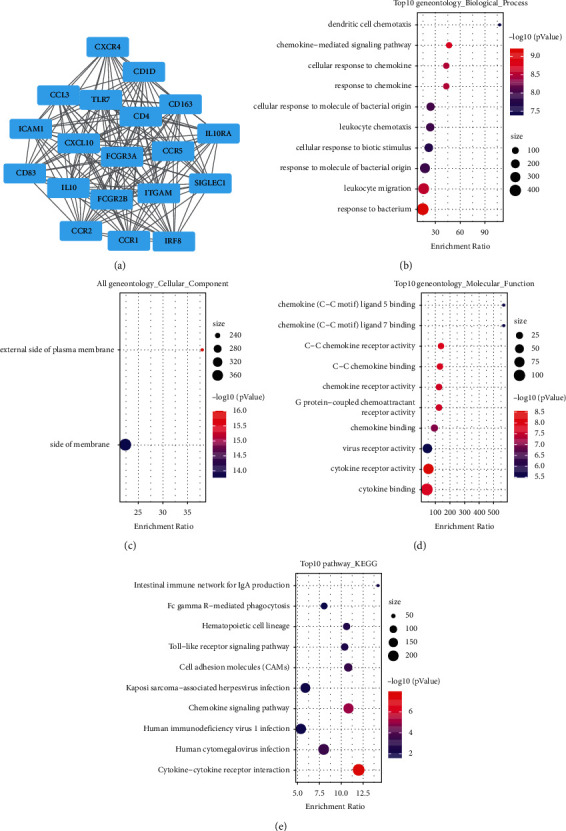
PPI model and KEGG/GO analysis. (a) PPI analysis diagram of module Mcode1; (b)–(e) GO and KEGG functional enrichment analysis of Mcode1 gene.

To determine whether *CD86* is associated with the tumor microenvironment in AML, StromalScore and ImmuneScore scores were calculated for AML samples using the ESTIMATE algorithm. Then, samples were divided based on *CD86* expression cutoffs, and limma package was used to identify the DEGs in high vs. low *CD86* expression, StromalScore, and ImmuneScore groups (Supplementary Table 4). Volcano and heat maps of the DEGs were plotted (Supplementary Figure 4). The DEGs common to the *CD86*, StromalScore, and ImmuneScore groups were determined. Accordingly, 308 common up-regulated genes and 16 common down-regulated genes were identified (Figures 5(a) and 5(b)). Then, we used WebGestaltR for the GO and KEGG functional enrichment analysis of the DERs. The DERs were found to be closely related to tumorigenesis and immune pathways (Figures 5(c)–5(f)). There were 3 clusters with more than five genes, namely, Mcode1, Mcode2, and Mcode4. Subsequently, WebGestaltR was used for GO and KEGG functional enrichment analysis to identify the functions of the clusters (Supplementary Figures 5 and 6). The Mcode1 module was closely related to immune pathways (Figure 6(a)), including the toll-like receptor (TLR) signaling pathway and cytokine-cytokine receptor interaction (Figures 6(b)–6(e)).

### 3.6. IRS Model Construction and Verification

**Figure 7:**
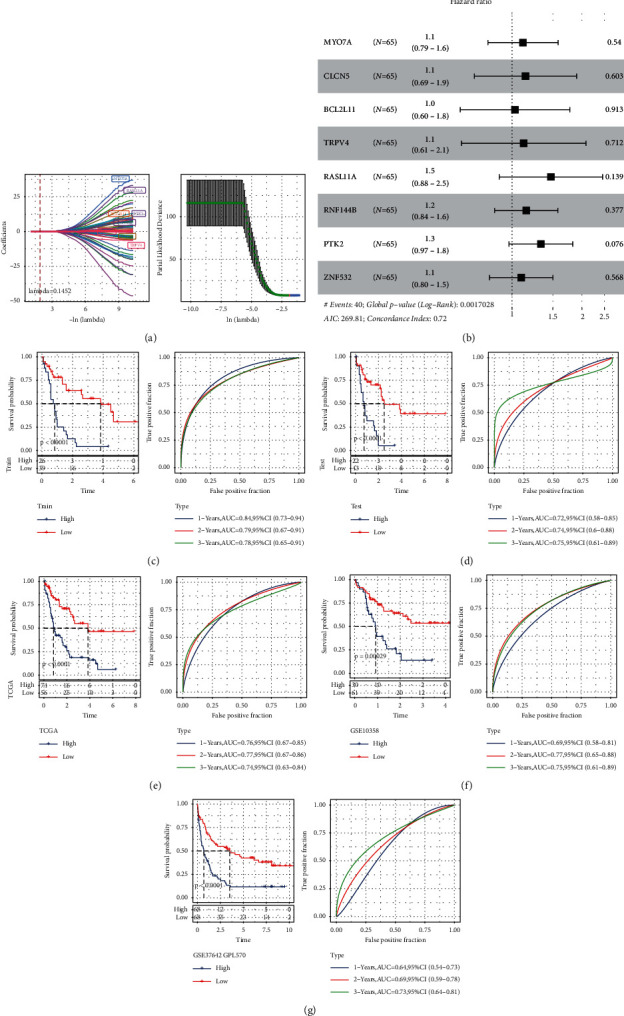
IRS construction and validation. (a) LASSO coefficient profiles of 40 prognostic RNAs in GEO training cohort. The coefficient profile plot was developed against the log (lambda) sequence; (b) the forest map shows the genetic multifactorial results of the final IRS model; (c) KM and ROC analysis of IRS model on TCGA training dataset; (d) KM and ROC analysis of IRS model on TCGA validation dataset; (e) KM and ROC analysis of IRS model on all TCGA data sets; (f) KM and ROC analysis of IRS model on all datasets of GSE10358; (g) KM and ROC analysis of the IRS model on the entire dataset of GSE37642.

After a series of analyses, 324 DERs were identified. Subsequently, 65 prognostic genes were obtained through random sampling based on TCGA samples (training: test = 1 : 1) and univariate Cox regression (*P* < 0.05, Supplementary Table 5). Then, the LASSO method was used to select the best genes, and six genes were obtained according to the minimum lambda cutoff of 0.1452 (Figure 7(a)). Multivariate Cox regression analysis was performed using these six genes, and the risk coefficients of related genes were obtained and represented by a forest map (Figure 7(b)). Then, the risk score of each sample in the TCGA training and verification datasets was calculated. The samples were divided into two groups (high vs. low expression) based on the best cutoff, and Kaplan–Meier curves were drawn. Further, ROC curve analysis was also performed. The results showed that the low-expression group had a better survival prognosis (Figures 7(c) and 7(d)). Subsequently, IRS model validation was performed using all TCGA datasets, GSE10358 datasets, and GSE37642 (GPL570) datasets. The results showed that patients in the low-expression group had a good prognosis (Figures 7(e)–7(g)). In order to further verify the accuracy of the IRS, evaluations were performed using the following GEO datasets: GSE146173, GSE106291, GSE37642 (a subset of the GPL96 platform), GSE12417 (a subset of the GPL97 platform), and GSE12417 (a subset of the GPL96 platform). Here too, the results revealed a better prognosis in the low-risk group (Supplementary Figures 7A–7E).

### 3.7. Association between IRS and Immunity

**Figure 8:**
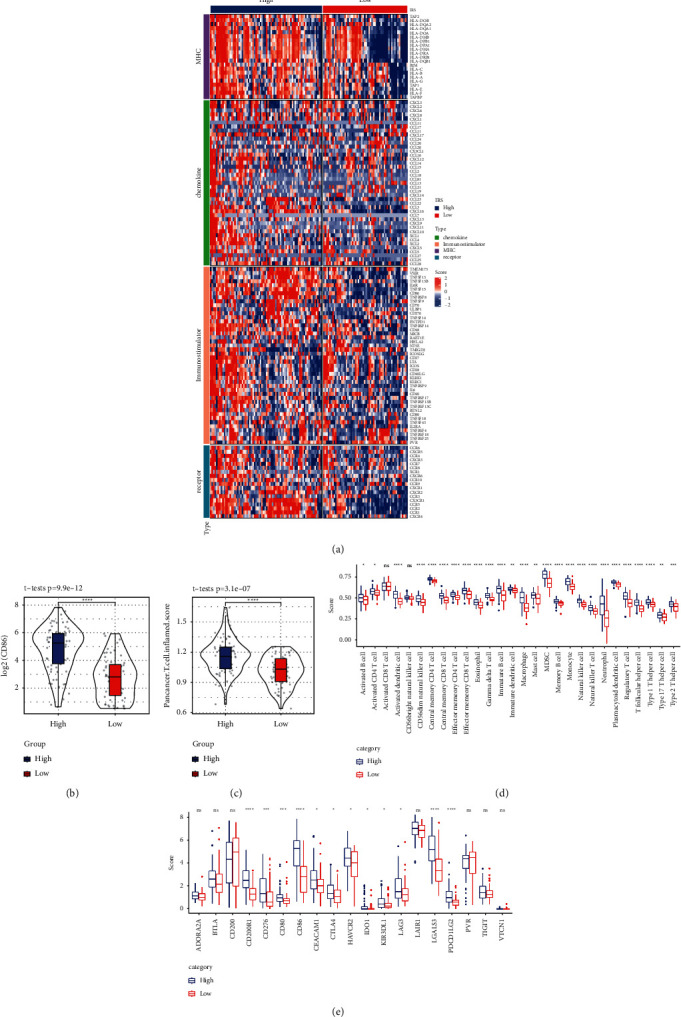
Different risk IRS typing groups were associated with immunity. (a) Differential expression of high- and low-risk group and concentration of chemokine, immunostimulator, MHC, and receptor genes; (b) The *CD86* expression level in IRS high- and low-risk group; (c) T-cell validation score of generalized carcinoma in IRS high- and low-risk group; (d) ssGSEA showed the correlation between IRS high- and low-risk group and 28 kinds of immune cells; (e) correlation between high- and low-risk groups and immunoassay sites.

Meanwhile, based on the TCGA dataset, we compared the differential expression of high and low IRC expression groups and concentration of chemokine, immunostimulator, MHC, and receptor genes. These were represented by heat maps (Figure 8(a)). The difference in *CD86* expression between the high- and low-expression groups was detected (Figure 8(b)). Analyses of inflammation scores for pan-cancer T cells revealed significantly higher scores in the high-expression group (Figure 8(c)). Subsequently, we plotted the correlation between IRC and 28 types of immune cells using ssGSEA method. The results suggested that the high-expression group was enriched for a variety of immune cells (Figure 8(d)). Differences in immunoassay sites and IRC groupings were also examined (Figure 8(e)). The results suggested that a high IRS is correlated with immune cells in AML.

### 3.8. Performance Comparison between IRS and TIDE

**Figure 9:**
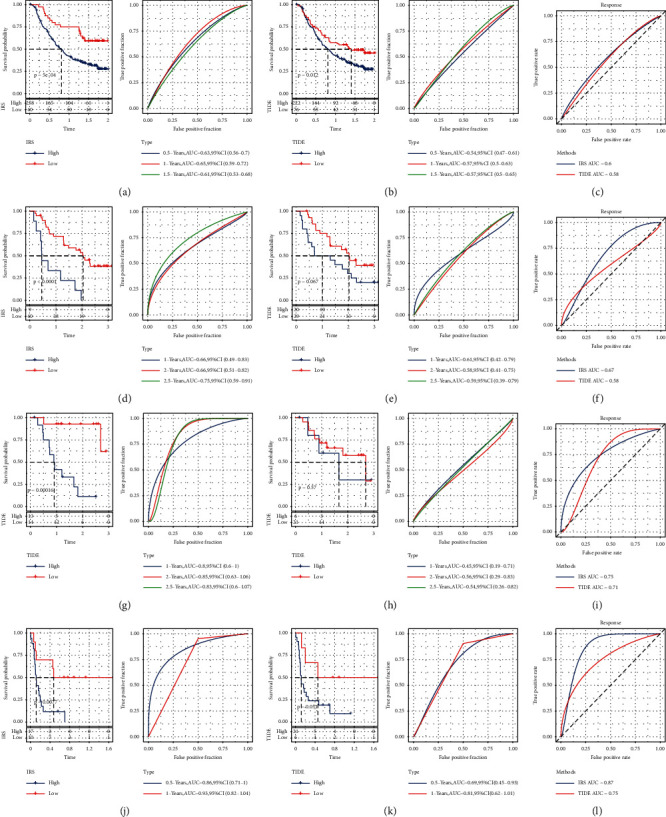
Performance comparison of IRS and TIDE (a) IRS survival curve and ROC curve of dataset IMvigor210; (b) TIDE survival curve and ROC curve of dataset IMvigor210; (c) ROC curves of IRS and TIDE effect on immunotherapy in dataset IMvigor210; (d) IRS survival curve and ROC curve of dataset GSE91061; (e) TIDE survival curve and ROC curve of dataset GSE91061; (f) ROC curve of IRS and TIDE effect on immunotherapy in dataset GSE91061; (g) IRS survival curve and ROC curve of dataset GSE78220; (h) TIDE survival curve and ROC curve of dataset GSE78220; (i) ROC curves of IRS and TIDE effect on immunotherapy in dataset GSE78220; (j) IRS survival curve and ROC curve of dataset GSE135222; (k) TIDE survival curve and ROC curve of dataset GSE135222; (l) ROC curves of IRS and TIDE effects on immunotherapy in dataset GSE135222.

To verify the effect of the IRS model constructed by us, we collected data from the IMvigor210, GSE91061, GSE78220, and GSE135222 datasets after immunotherapy. We used our method to calculate the IRS, and the TIDE website was used to evaluate the TIDE score (https://tide.dfci.harvard.edu/) for immune treatment effects. The predictive value of the IRS and TIDE for the response to treatment was then compared. Survival prediction curves and Kaplan–Meier curves (median cutoff) were used for analysis. Our IRS score was found to be better than the TIDE score (Figures 9(a)–9(k)).

## 4. Discussion

AML is a malignancy tightly linked to the bone marrow microenvironment [18]. The BMM is mainly composed of immune cells and stromal cells, with the former playing a key role in AML progression [2]. T cells are important cells of the immune system [19]. According to findings, a high lymphocyte count in the bone marrow is directly associated with better overall survival in patients with AML.

In AML, T cell dysfunction is caused by the immunological conflict between a dysfunctional cytokine regulatory network and overactivated T cells [20]. The complete activation of T cells requires two stimuli. During the first signal, TCRs need to bind to the antigenic peptide-bound MHC on APCs [21]. The secondary signal is provided by costimulatory molecules on APCs that interact with receptors on the surface of T cells. The most important costimulatory molecules are *CD86* and *CD80*.

A molecule can be a central target for cancer immunotherapy depending on its specific expression in the tumor microenvironment. *CD86* (B7-2), a member of the B7 family of proteins, is one of the surface proteins of APCs [22]. The B7 family has been implicated in the progression of AML. The levels of *CD80* (B7-1) are elevated in AML [23]. Moreover, programmed cell death ligand (PD-L1, B7H-1) is abnormally expressed in AML patients and is directly associated with a poor prognosis [24]. T cells can be activated to exert immune effects only when *CD86* is expressed on APC membranes and binds to *CD28* on the surface of T cells [11]. Using data from public databases, we found that *CD86* is overexpressed in many cancers, and especially in AML. We also demonstrated this in AML cell lines. In AML, a high expression of *CD86* was found to be associated with a poor prognosis. Further, clinical data from GEO and TCGA datasets show that high *CD86* expression is directly associated with a poor prognosis in AML.

In AML, the immunomodulatory network in the BMM is an important factor promoting cancer progression [25]. These regulatory networks include chemotactic cytokines, immunostimulatory molecules, MHC, and receptors. Interestingly, the expression of *CD86* is positively correlated with the expression of these genes [26]. Using TCGA data, we observed increased infiltration of dendritic cells, NK cells, CD4+ cells, CD8+ cells, macrophages, and Th1 cells in the group with high *CD86* expression. This was confirmed using external validation data. The upregulation of immune inspection sites by infiltrating immune cells is also a key factor in cancer progression. Some immune targets—including cytotoxic T lymphocyte antigen 4 (CTLA4), programmed cell death protein 1 ligand 2 (PDCD1LG2), indoleamine 2,3-dioxygenase 1 (IDO1), and hepatitis A virus cellular receptor 2 (HAVCR2)—promote the progression of AML [27–29]. However, immune checkpoints act as double-edged swords in AML. Clinical studies on targeted ICBs have shown that drug resistance is a key factor leading to a poor prognosis after AML treatment using ICBs. Our study showed that *CD86* expression was positively correlated with these immunoassay sites. This may be because *CD86* promotes the expression of immune-infiltrating cells in BMM, thus stimulating the expression of immune checkpoints. This indicates that AML patients with low *CD86* expression may not be responsive to ICBs. Meanwhile, we calculated the differentially expressed genes based on *CD86* expression and the immune and matrix components in the BMM. These DEGs were mainly concentrated in the TLR signaling pathway, cytokine–cytokine receptor interaction, and other immune-related pathways.

Our findings also confirmed the involvement of *CD86* in the immune response in AML. The pan-cancerT-cell inflammation score indicates the efficacy of anti-PD-1 immunotherapy for various cancers [30]. The high expression of *CD86* was positively correlated with a high T-cell inflammation score [31, 32], indicating that high *CD86* expression was negatively correlated with the effects of immunotherapy.

In AML, mutation sites not only affect disease classification but also affect risk stratification and chemotherapeutic resistance. For example, *FLT3* mutations are detected in about one-third of AML patients, and these mutations are directly related to the poor prognosis of AML [33]. However, interestingly, the mutation rates of *DNMT3A*, *FLT3*, *NPM1*, and *IDH2* were higher in the low *CD86* expression group in our study [34, 35]. The mutation rate of *RUNX1* was higher in the *CD86* group, which could be because of the number of samples. Our study also showed that *CD86* expression was negatively correlated with DNA methylation. This was noteworthy because methylation has been found to predict chemotherapy outcomes in AML [36]. Meanwhile, we predicted that mutations in *RB1*, *ERBB2*, and *FANCC* increased as *CD86* expression increased, suggesting that *CD86* may be related to radiotherapy and chemotherapy resistance in AML. However, further verification is still needed.

The IRS is a genetic prognostic model calculated using a formula to assess the risk of a disease. An IRS can predict the survival and prognosis of AML patients undergoing chemotherapy. Immune risk scores can be used to predict the benefit of adjuvant chemotherapy in different risk groups of patients. Wang Yun et al. [37] constructed different algorithms to evaluate the prognostic models of AML immune components after receiving different degrees of radiotherapy, and the results were highly accurate. However, in cases of AML, the IRS is currently inaccurate and inconsistent. Hence, we developed an IRS model to predict the overall prognosis of AML. Verification with external datasets showed that our model is superior to the TIDE score. This complements the enrichment of AML risk scores.

Nevertheless, there are some limitations to our study. First, all our samples were obtained from public databases, and a large number of patient samples are still needed for follow-up verification. Second, no *in vivo* experiments or mechanistic studies were performed. This area needs to be explored further.

## 5. Conclusion

This study found that *CD86* is involved in the progression of AML and is closely related to the BMM in AML. The expression of *CD86* could be used to predict immunotherapy efficacy. Therefore, the development of *CD86*-targeting drugs could lead to advancements in AML treatment.

## Data Availability

The data used to support the findings of this study are included within the article.

## Supplementary Materials

Supplementary Materials Supplementary Figure 1. *CD86* mRNA expression in various cancers. Supplementary Figure 2. OS survival and prognosis of *CD86* in high- and low-expression group in various cancers. Supplementary Figure 3. *CD86* and univariate analysis in various cancers. Supplementary Figure 4. Heat and volcano maps showed the high and low expression of *CD86*, immune score and matrix score. A. Gene heat map and volcano map of *CD86* high- and low-expression group; B. Gene heat map and volcano map of the high- and low-expression immune score group; C. Gene heat map and volcano map of high- and low-expression group with matrix score. Supplementary Figure 5. The PPI network, GO and KEGG analyzed of Mcode2. A. the PPI network diagram of Mcode2; B-C. Enrichment of the top ten of Go analysis; D-E. KEGG was used to analyze the top ten enrichment pathways. Supplementary Figure 6. The PPI network, GO and KEGG analyzed of Mcode4. A. the PPInetwork diagram of Mcode4; B-C. Enrichment of the top ten of Go analysis; D-E. KEGG was used to analyze the top ten enrichment pathways. Supplementary Figure 7. IRS different risk groups scores of survival prognosis in GEO data set were correlated with GSE146173, GSE106291, GSE37642 (the GPL96 platform), GSE12417 (the GPL97 platform), And GSE12417 (the GPL96 Platform) OS curve and ROC curve. Supplementary Table 1. Chemokines, immunostimulators, MHC proteins, and receptors in TISIDB website. Supplementary Table 2. Differences in expression of chemokines, immune stimulators, MHC proteins and immune receptors between *CD86*high-expression group and low expression group. Supplement Table 3. Differences between *CD86* high- and low-expression group and CD8+ T cell recruitment, macrophage, NK cell, and Th1. Supplementary Table 4. Differential genes in StromalScore, ImmuneScore and *CD86* high- and low-expression groups. Supplement Table 5. 65 genes screened out by Cox regression analysis.
