# Supplementary material for: CD86 Is Associated with Immune Infiltration and Immunotherapy Signatures in AML and Promotes Its Progression
Source: J Oncol. 2023 Apr 7;2023:9988405. doi: 10.1155/2023/9988405 (PMC10104747; doi:10.1155/2023/9988405)
Supplement: Supplementary Materials — Supplementary Figure 1. CD86 mRNA expression in various cancers. Supplementary Figure 2. OS survival and prognosis of CD86 in high- and low-expression group in various cancers. Supplementary Figure 3. CD86 and univariate analysis in various cancers. Supplementary Figure 4. Heat and volcano maps showed the high and low expression of CD86, immune score and matrix score. A. Gene heat map and volcano map of CD86 high- and low-expression group; B. Gene heat map and volcano map of the high- and low-expression immune score group; C. Gene heat map and volcano map of high- and low-expression group with matrix score. Supplementary Figure 5. The PPI network, GO and KEGG analyzed of Mcode2. A. the PPI network diagram of Mcode2; B-C. Enrichment of the top ten of Go analysis; D-E. KEGG was used to analyze the top ten enrichment pathways. Supplementary Figure 6. The PPI network, GO and KEGG analyzed of Mcode4. A. the PPInetwork diagram of Mcode4; B-C. Enrichment of the top ten of Go analysis; D-E. KEGG was used to analyze the top ten enrichment pathways. Supplementary Figure 7. IRS different risk groups scores of survival prognosis in GEO data set were correlated with GSE146173, GSE106291, GSE37642 (the GPL96 platform), GSE12417 (the GPL97 platform), And GSE12417 (the GPL96 Platform) OS curve and ROC curve. Supplementary Table 1. Chemokines, immunostimulators, MHC proteins, and receptors in TISIDB website. Supplementary Table 2. Differences in expression of chemokines, immune stimulators, MHC proteins and immune receptors between CD86high-expression group and low expression group. Supplement Table 3. Differences between CD86 high- and low-expression group and CD8+ T cell recruitment, macrophage, NK cell, and Th1. Supplementary Table 4. Differential genes in StromalScore, ImmuneScore and CD86 high- and low-expression groups. Supplement Table 5. 65 genes screened out by Cox regression analysis. [file 9988405.f1.zip › 9988405.f1/Supplementary table 1 (1).pdf]

Supplementary table 1: chemokines, immunostimulators, MHC proteins, and receptors in TISIDB website

Genes	Type	Alisa
CCL1	chemokine	CCL1
CCL2	chemokine	CCL2
CCL3	chemokine	CCL3
CCL4	chemokine	CCL4
CCL5	chemokine	CCL5
CCL7	chemokine	CCL7
CCL8	chemokine	CCL8
CCL11	chemokine	CCL11
CCL13	chemokine	CCL13
CCL14	chemokine	CCL14
CCL15	chemokine	CCL15
CCL16	chemokine	CCL16
CCL17	chemokine	CCL17
CCL18	chemokine	CCL18
CCL19	chemokine	CCL19
CCL20	chemokine	CCL20
CCL21	chemokine	CCL21
CCL22	chemokine	CCL22
CCL23	chemokine	CCL23
CCL24	chemokine	CCL24
CCL25	chemokine	CCL25
CCL26	chemokine	CCL26
CCL27	chemokine	CCL27
CCL28	chemokine	CCL28
CX3CL1	chemokine	CX3CL1
CXCL1	chemokine	CXCL1
CXCL2	chemokine	CXCL2
CXCL3	chemokine	CXCL3
CXCL5	chemokine	CXCL5
CXCL6	chemokine	CXCL6
CXCL8	chemokine	CXCL8
CXCL9	chemokine	CXCL9
CXCL10	chemokine	CXCL10
CXCL11	chemokine	CXCL11
CXCL12	chemokine	CXCL12
CXCL13	chemokine	CXCL13
CXCL14	chemokine	CXCL14
CXCL16	chemokine	CXCL16
CXCL17	chemokine	CXCL17

XCL1	chemokine	XCL1
XCL2	chemokine	XCL2
CCR1	receptor	CCR1
CCR2	receptor	CCR2
CCR3	receptor	CCR3
CCR4	receptor	CCR4
CCR5	receptor	CCR5
CCR6	receptor	CCR6
CCR7	receptor	CCR7
CCR8	receptor	CCR8
CCR9	receptor	CCR9
CCR10	receptor	CCR10
CXCR1	receptor	CXCR1
CXCR2	receptor	CXCR2
CXCR3	receptor	CXCR3
CXCR4	receptor	CXCR4
CXCR5	receptor	CXCR5
CXCR6	receptor	CXCR6
XCR1	receptor	XCR1
CX3CR1	receptor	CX3CR1
B2M	MHC	B2M
HLA-A	MHC	HLA-A
HLA-B	MHC	HLA-B
HLA-C	MHC	HLA-C
HLA-DMA	MHC	HLA-DMA
HLA-DMB	MHC	HLA-DMB
HLA-DOA	MHC	HLA-DOA
HLA-DOB	MHC	HLA-DOB
HLA-DPA1	MHC	HLA-DPA1
HLA-DPB1	MHC	HLA-DPB1
HLA-DQA1	MHC	HLA-DQA1
HLA-DQA2	MHC	HLA-DQA2
HLA-DQB1	MHC	HLA-DQB1
HLA-DRA	MHC	HLA-DRA
HLA-DRB1	MHC	HLA-DRB1
HLA-E	MHC	HLA-E
HLA-F	MHC	HLA-F
HLA-G	MHC	HLA-G
TAP1	MHC	TAP1
TAP2	MHC	TAP2
TAPBP	MHC	TAPBP
BTNL2	Immunostim	BTNL2
VISTA	Immunostim	VSIR
CD27	Immunostim	CD27
B7-H3	Immunostim	CD276
CD28	Immunostim	CD28
CD40	Immunostim	CD40
CD40LG	Immunostim	CD40LG
CD48	Immunostim	CD48
CD70	Immunostim	CD70
B7.1	Immunostim	CD80

B7.2	Immunostim	CD86
CD39	Immunostim	ENTPD1
B7-H7	Immunostim	HHLA2
ICOS	Immunostim	ICOS
B7-H2	Immunostim	ICOSLG
CD25	Immunostim	IL2RA
IL6	Immunostim	IL6
IL6R	Immunostim	IL6R
NKG2A	Immunostim	KLRC1
NKG2D	Immunostim	KLRK1
LTA	Immunostim	LTA
MICB	Immunostim	MICB
CD73	Immunostim	NT5E
CD155	Immunostim	PVR
RAET1E	Immunostim	RAET1E
STING	Immunostim	TMEM173
TMIGD2	Immunostim	TMIGD2
CD267	Immunostim	TNFRSF13B
BAFF-R	Immunostim	TNFRSF13C
HVEM	Immunostim	TNFRSF14
BCMA	Immunostim	TNFRSF17
GITR	Immunostim	TNFRSF18
DR3	Immunostim	TNFRSF25
OX40	Immunostim	TNFRSF4
CD30	Immunostim	TNFRSF8
4-1BB	Immunostim	TNFRSF9
APRIL	Immunostim	TNFSF13
BAFF	Immunostim	TNFSF13B
LIGHT	Immunostim	TNFSF14
TL1A	Immunostim	TNFSF15
GITRL	Immunostim	TNFSF18
OX-40L	Immunostim	TNFSF4
4-1BB-L	Immunostim	TNFSF9
ULBP1	Immunostim	ULBP1
