# Supplementary material for: CD86 Is Associated with Immune Infiltration and Immunotherapy Signatures in AML and Promotes Its Progression
Source: J Oncol. 2023 Apr 7;2023:9988405. doi: 10.1155/2023/9988405 (PMC10104747; doi:10.1155/2023/9988405)
Supplement: Supplementary Materials — Supplementary Figure 1. CD86 mRNA expression in various cancers. Supplementary Figure 2. OS survival and prognosis of CD86 in high- and low-expression group in various cancers. Supplementary Figure 3. CD86 and univariate analysis in various cancers. Supplementary Figure 4. Heat and volcano maps showed the high and low expression of CD86, immune score and matrix score. A. Gene heat map and volcano map of CD86 high- and low-expression group; B. Gene heat map and volcano map of the high- and low-expression immune score group; C. Gene heat map and volcano map of high- and low-expression group with matrix score. Supplementary Figure 5. The PPI network, GO and KEGG analyzed of Mcode2. A. the PPI network diagram of Mcode2; B-C. Enrichment of the top ten of Go analysis; D-E. KEGG was used to analyze the top ten enrichment pathways. Supplementary Figure 6. The PPI network, GO and KEGG analyzed of Mcode4. A. the PPInetwork diagram of Mcode4; B-C. Enrichment of the top ten of Go analysis; D-E. KEGG was used to analyze the top ten enrichment pathways. Supplementary Figure 7. IRS different risk groups scores of survival prognosis in GEO data set were correlated with GSE146173, GSE106291, GSE37642 (the GPL96 platform), GSE12417 (the GPL97 platform), And GSE12417 (the GPL96 Platform) OS curve and ROC curve. Supplementary Table 1. Chemokines, immunostimulators, MHC proteins, and receptors in TISIDB website. Supplementary Table 2. Differences in expression of chemokines, immune stimulators, MHC proteins and immune receptors between CD86high-expression group and low expression group. Supplement Table 3. Differences between CD86 high- and low-expression group and CD8+ T cell recruitment, macrophage, NK cell, and Th1. Supplementary Table 4. Differential genes in StromalScore, ImmuneScore and CD86 high- and low-expression groups. Supplement Table 5. 65 genes screened out by Cox regression analysis. [file 9988405.f1.zip › 9988405.f1/Supplementary table 2 (1).pdf]

Supplementary Table 2: Differences in expression of chemokines, immune stimulators, MHC proteins and immune receptors between CD86 high expression group and low expression group

	logFC	AveExpr	t	P.Value	adj.P.Val	B
CCL1	-0.462567	1.1481064	-1.653331	0.1006921	0.2071248	-5.648527
CCL2	0.1080616	0.569383	0.8224799	0.4123196	0.5683743	-6.663081
CCL3	0.8892518	2.613315	4.483828	1.60E-05	0.0001599	2.3323578
CCL4	-0.048188	2.5345371	-0.270802	0.7869755	0.8693542	-6.963631
CCL5	0.3347618	5.8737699	1.5872378	0.1149039	0.2283994	-5.753417
CCL7	0.0265143	0.0657784	0.5958763	0.5523006	0.6901541	-6.823065
CCL8	0.0060869	0.2202482	0.0749199	0.9403943	0.9645833	-6.997466
CCL11	0.0072089	0.0162463	0.8209828	0.4131688	0.5690417	-6.664304
CCL13	-0.042796	0.2832065	-0.537659	0.5917379	0.7233178	-6.855962
CCL14	-0.126571	0.3836673	-1.443669	0.1512533	0.27971	-5.967049
CCL15	-0.030807	0.0758897	-1.732878	0.0855042	0.1829479	-5.516858
CCL16	-0.035597	0.1623506	-1.292326	0.1985514	0.3403719	-6.171
CCL17	-0.031916	0.2360376	-0.681163	0.4969875	0.6440214	-6.768806
CCL18	-0.098187	0.7177704	-0.537288	0.5919932	0.7235389	-6.856161
CCL19	-0.102716	0.2201384	-1.443978	0.1511665	0.2796559	-5.96661
CCL20	-0.000402	0.2312239	-0.005405	0.9956957	0.9975024	-7.000256
CCL21	-0.047775	0.2633422	-0.680912	0.4971459	0.6440214	-6.768976
CCL22	0.3066726	0.325295	4.6152296	9.35E-06	0.0001018	2.846681
CCL23	1.1965472	2.7304292	3.5473641	0.000543	0.0030628	-1.001459
CCL24	0.3565349	1.1206307	1.6784617	0.0956749	0.1991479	-5.607569
CCL25	0.1567266	0.724642	1.123387	0.2633569	0.4151417	-6.372652
CCL26	-0.001159	0.1722887	-0.02864	0.9771961	0.9863833	-6.999861
CCL27	0.0222895	0.0448076	1.2584392	0.210503	0.3550345	-6.213657
CCL28	0.1004546	2.5010798	0.4933719	0.6225877	0.74792	-6.878735
CX3CL1	-0.038709	0.0989784	-1.430341	0.155034	0.2847802	-5.985889
CXCL1	0.4170342	1.7234051	1.7800218	0.0774237	0.1694978	-5.436037
CXCL2	-0.23333	3.8771162	-0.863933	0.3892261	0.5456624	-6.628337
CXCL3	-0.085496	2.15947	-0.375439	0.7079495	0.8125448	-6.929865
CXCL5	-0.012032	0.396596	-0.104301	0.9170924	0.9512132	-6.994834
CXCL6	-0.089238	0.2956513	-1.369741	0.1731443	0.3084108	-6.069413
CXCL8	0.4854155	4.8988748	1.4694085	0.1441536	0.2700191	-5.930186
CXCL9	0.14753	0.6178084	0.81861	0.4145167	0.570227	-6.666239
CXCL10	0.8639656	1.2405271	4.2728545	3.72E-05	0.0003239	1.5294177
CXCL11	0.1745495	0.2638565	2.1953481	0.0299252	0.0805118	-4.635826
CXCL12	-0.298925	3.4022286	-0.67177	0.50293	0.6491868	-6.775135
CXCL13	-0.025339	0.3784491	-0.339269	0.7349581	0.8324961	-6.942772
CXCL14	0.210799	0.5921051	1.4667924	0.1448631	0.2708529	-5.933961
CXCL16	2.2016654	2.8792448	10.449706	6.71E-19	1.18E-16	32.428706
CXCL17	-0.34978	1.7260152	-2.852663	0.0050505	0.0191546	-3.057305
XCL1	-0.04079	0.7389787	-0.365421	0.7153946	0.8183869	-6.93357
XCL2	-0.074123	1.585167	-0.440231	0.6605059	0.7769248	-6.903488
CCR1	2.8949414	4.3754328	12.729886	1.47E-24	7.55E-22	45.247781
CCR2	3.1918027	3.9896663	12.050278	7.07E-23	2.55E-20	41.439391
CCR3	0.3372474	0.7144292	2.9485177	0.0037909	0.0152012	-2.796536
CCR4	0.0179573	1.5909334	0.1089279	0.9134287	0.9489282	-6.994341
CCR5	2.0661275	2.615431	10.470434	5.96E-19	1.06E-16	32.545228

CCR6	0.0327559	0.3757899	0.5993014	0.5500217	0.6884562	-6.821025
CCR7	0.142433	3.6394909	0.6283335	0.530895	0.6722377	-6.803265
CCR8	-0.10575	0.5826647	-1.239501	0.2174075	0.3628119	-6.237015
CCR9	0.0141856	1.2921943	0.1188077	0.9056123	0.9440188	-6.993217
CCR10	0.0960317	0.8841812	1.0940037	0.2759894	0.4291796	-6.404904
CXCR1	0.4310979	1.2165166	2.4047855	0.0176004	0.0528311	-4.173466
CXCR2	1.131885	2.6118279	5.5854781	1.32E-07	2.48E-06	6.9521578
CXCR3	-0.064158	2.2793732	-0.33399	0.7389287	0.8353129	-6.944546
CXCR4	0.8130352	8.1650359	4.4051717	2.20E-05	0.0002081	2.0296636
CXCR5	-0.038215	0.3715621	-0.642922	0.5214142	0.6648067	-6.794025
CXCR6	-0.065934	2.1240486	-0.54143	0.5891437	0.721159	-6.853933
XCR1	0.0799702	0.3134821	0.992747	0.3226894	0.479171	-6.509612
CX3CR1	2.9149394	4.9457563	9.8724082	1.80E-17	2.37E-15	29.191766
B2M	0.4206834	10.588844	4.4502953	1.83E-05	0.0001783	2.202834
HLA-A	0.2668634	9.1002307	1.9550087	0.0527417	0.1258093	-5.118074
HLA-B	0.6468637	9.869248	4.6507528	8.07E-06	8.99E-05	2.9875499
HLA-C	0.4921117	9.1037541	3.8076694	0.0002158	0.0014107	-0.136147
HLA-DMA	1.1735018	6.9038045	5.159937	9.07E-07	1.35E-05	5.0881498
HLA-DMB	1.8181002	4.7936246	7.4299209	1.32E-11	6.05E-10	15.932876
HLA-DOA	0.7374904	3.8670778	2.4415485	0.0159783	0.0488206	-4.088328
HLA-DOB	0.4161891	3.0448099	2.5969572	0.010496	0.0346365	-3.71549
HLA-DPA1	1.6228543	6.4088635	5.2243175	6.81E-07	1.04E-05	5.3641012
HLA-DPB1	1.4556864	7.0307646	5.0314375	1.60E-06	2.20E-05	4.5441154
HLA-DQA1	1.4711116	4.2468382	4.9893114	1.92E-06	2.57E-05	4.3677574
HLA-DQA2	0.8514862	3.2067699	2.1978591	0.0297414	0.0801201	-4.630513
HLA-DQB1	1.8713123	5.3110256	5.9080954	2.89E-08	6.38E-07	8.4247154
HLA-DRA	1.7951665	10.15013	6.2203556	6.38E-09	1.63E-07	9.8944187
HLA-DRB1	1.8404215	8.3925353	6.5690255	1.13E-09	3.42E-08	11.582554
HLA-E	0.5592047	9.1048175	4.304974	3.27E-05	0.0002918	1.6498061
HLA-F	0.7838752	4.4640923	4.0686323	8.18E-05	0.000627	0.7799735
HLA-G	0.4011164	2.4843648	2.6670089	0.0086323	0.0295128	-3.54066
TAP1	0.3087528	5.3907941	2.3551162	0.0200232	0.0585337	-4.286617
TAP2	0.2794076	4.5975606	2.3406188	0.0207838	0.0603029	-4.319235
TAPBP	0.1815401	7.55017	2.6180163	0.0099008	0.0330085	-3.663371
BTNL2	-0.016188	0.2761892	-0.302763	0.7625572	0.8519067	-6.954476
VSIR	1.7867308	7.0377514	8.9819863	2.74E-15	2.64E-13	24.25121
CD27	0.1032421	3.435623	0.6396986	0.5235016	0.6666672	-6.796085
CD276	0.3098782	1.3974076	1.4135531	0.1598987	0.2912315	-6.009378
CD28	-0.004849	2.1374245	-0.025716	0.9795234	0.9878239	-6.99994
CD40	0.3881258	2.5208415	1.976877	0.0501856	0.1210325	-5.076361
CD40LG	-0.156378	2.2987725	-0.878143	0.3814969	0.5383171	-6.616038
CD48	1.0910446	5.1777971	4.6852871	6.99E-06	7.93E-05	3.1252322
CD70	0.1015928	1.5425073	0.3884847	0.698297	0.8061256	-6.92489
CD80	-0.102394	0.9839942	-0.853134	0.3951642	0.551556	-6.637551
CD86	3.1820475	3.8908726	16.484874	1.45E-33	2.81E-29	65.609523
ENTPD1	0.4822608	4.2967044	3.77323	0.0002445	0.001567	-0.25346
HHLA2	0.73891	0.9628246	5.3540035	3.81E-07	6.25E-06	5.9266476
ICOS	0.0639916	1.5924252	0.3986607	0.6908015	0.8008935	-6.920892
ICOSLG	-0.057115	0.5687271	-0.607817	0.5443758	0.683665	-6.815902
IL2RA	0.3000976	2.3749164	0.9666603	0.3355204	0.4922518	-6.534967
IL6	0.1076263	0.2517914	1.8304567	0.069487	0.1562714	-5.34729

IL6R	0.8730454	6.1449863	6.436	2.20E-09	6.23E-08	10.932933
KLRC1	-0.03064	0.6626205	-0.277583	0.7817761	0.8653756	-6.961774
KLRK1	-0.075618	1.024701	-0.636499	0.5255777	0.6682615	-6.798119
LTA	0.1336307	1.1410355	1.0785295	0.2828076	0.4368096	-6.421552
MICB	0.4651475	4.7169043	5.6731259	8.77E-08	1.73E-06	7.3473721
NT5E	0.2859555	1.837652	1.2162812	0.2260959	0.3725194	-6.265182
PVR	-0.54375	3.9830109	-2.363333	0.0196031	0.0575205	-4.268047
RAET1E	-0.060613	0.4970636	-0.658082	0.5116566	0.6563937	-6.784201
TMEM173	0.2709699	7.3467821	2.1702444	0.0318176	0.0844708	-4.688632
TMIGD2	-1.090223	3.5393041	-3.307245	0.0012205	0.0059359	-1.754836
TNFRSF13B	0.1469521	0.8255735	1.4106044	0.1607652	0.2924369	-6.013476
TNFRSF13C	0.0896496	2.9972156	0.5932478	0.5540527	0.6915442	-6.824622
TNFRSF14	0.418859	4.9473379	2.2592841	0.0255413	0.0711268	-4.498795
TNFRSF17	-0.031134	2.5679468	-0.170401	0.8649615	0.9194459	-6.985761
TNFRSF18	-0.851536	1.7879866	-3.262457	0.0014132	0.0066992	-1.890459
TNFRSF25	-0.682691	3.3376142	-3.270195	0.001378	0.0065548	-1.867141
TNFRSF4	-0.836884	2.2250361	-2.926764	0.0040484	0.0160418	-2.856379
TNFRSF8	1.2369701	1.5169365	8.1975253	2.11E-13	1.34E-11	19.986243
TNFRSF9	0.2600499	0.7123209	3.0925374	0.0024323	0.0105511	-2.390658
TNFSF13	1.1758544	4.1076171	8.6855458	1.43E-14	1.17E-12	22.627623
TNFSF13B	1.0393953	6.4554854	6.5843516	1.04E-09	3.19E-08	11.657822
TNFSF14	0.4250124	2.1575881	2.4702958	0.014804	0.0459321	-4.020934
TNFSF15	0.6825729	0.509486	11.161494	1.14E-20	2.81E-18	36.436104
TNFSF18	0.0687802	0.1783799	1.6602265	0.0992948	0.2048639	-5.637348
TNFSF4	-0.143532	3.6603202	-0.700098	0.4851254	0.6339396	-6.755784
TNFSF9	0.7827279	2.2943841	3.6391601	0.0003942	0.0023475	-0.701975
ULBP1	0.034404	0.2495607	0.6867393	0.4934781	0.6414886	-6.765008
