# Supplementary material for: CD86 Is Associated with Immune Infiltration and Immunotherapy Signatures in AML and Promotes Its Progression
Source: J Oncol. 2023 Apr 7;2023:9988405. doi: 10.1155/2023/9988405 (PMC10104747; doi:10.1155/2023/9988405)
Supplement: Supplementary Materials — Supplementary Figure 1. CD86 mRNA expression in various cancers. Supplementary Figure 2. OS survival and prognosis of CD86 in high- and low-expression group in various cancers. Supplementary Figure 3. CD86 and univariate analysis in various cancers. Supplementary Figure 4. Heat and volcano maps showed the high and low expression of CD86, immune score and matrix score. A. Gene heat map and volcano map of CD86 high- and low-expression group; B. Gene heat map and volcano map of the high- and low-expression immune score group; C. Gene heat map and volcano map of high- and low-expression group with matrix score. Supplementary Figure 5. The PPI network, GO and KEGG analyzed of Mcode2. A. the PPI network diagram of Mcode2; B-C. Enrichment of the top ten of Go analysis; D-E. KEGG was used to analyze the top ten enrichment pathways. Supplementary Figure 6. The PPI network, GO and KEGG analyzed of Mcode4. A. the PPInetwork diagram of Mcode4; B-C. Enrichment of the top ten of Go analysis; D-E. KEGG was used to analyze the top ten enrichment pathways. Supplementary Figure 7. IRS different risk groups scores of survival prognosis in GEO data set were correlated with GSE146173, GSE106291, GSE37642 (the GPL96 platform), GSE12417 (the GPL97 platform), And GSE12417 (the GPL96 Platform) OS curve and ROC curve. Supplementary Table 1. Chemokines, immunostimulators, MHC proteins, and receptors in TISIDB website. Supplementary Table 2. Differences in expression of chemokines, immune stimulators, MHC proteins and immune receptors between CD86high-expression group and low expression group. Supplement Table 3. Differences between CD86 high- and low-expression group and CD8+ T cell recruitment, macrophage, NK cell, and Th1. Supplementary Table 4. Differential genes in StromalScore, ImmuneScore and CD86 high- and low-expression groups. Supplement Table 5. 65 genes screened out by Cox regression analysis. [file 9988405.f1.zip › 9988405.f1/Supplementary table 3 (1).pdf]

Supplementary Table 3: Differences between CD86 high-low expression group and CD8+ T cell recruitment, macrophage, NK cell, and Th1

	logFC	AveExpr	t	P.Value	adj.P.Val	B
PRF1	-0.317407	4.4241961	-1.35834	0.1767237	0.3126403	-6.084735
GZMB	0.3267575	2.8684711	1.2922731	0.1985698	0.3403719	-6.171068
CXCL10	0.8639656	1.2405271	4.2728545	3.72E-05	0.0003239	1.5294177
GZMA	0.0939196	3.8879433	0.4200374	0.675156	0.7890539	-6.912157
CCL5	0.3347618	5.8737699	1.5872378	0.1149039	0.2283994	-5.753417
CD8A	0.0033851	2.9310479	0.0166581	0.9867351	0.9924607	-7.000132
IFNG	-0.006866	1.1570379	-0.045391	0.9638657	0.9792131	-6.999241
TBX21	-0.087613	2.1673691	-0.45898	0.6470202	0.7667628	-6.895075
CXCR3	-0.064158	2.2793732	-0.33399	0.7389287	0.8353129	-6.944546
CD8B	-0.013057	2.0540622	-0.074622	0.9406306	0.9645837	-6.997488
GZMM	-0.143331	2.5900803	-0.815418	0.4163342	0.5719232	-6.668832
FLT3LG	0.1194119	1.9298247	1.1661922	0.2456856	0.3958162	-6.324168
SIGLEC1	2.1803998	1.8598039	8.254204	1.54E-13	1.02E-11	20.290751
SLAMF8	0.0495674	1.099478	0.3895149	0.6975368	0.805726	-6.92449
SLC15A3	2.7201401	2.5655756	15.549806	2.22E-31	1.08E-27	60.676681
LILRB4	3.3745861	2.7990174	13.621738	9.53E-27	7.42E-24	50.203425
FGL2	3.3057428	5.3996481	14.042572	9.02E-28	8.78E-25	52.520264
IL21R	0.4592228	1.6303061	3.2646737	0.001403	0.0066543	-1.883784
CLEC5A	0.6773409	3.5851859	1.8234176	0.0705524	0.1580813	-5.359818
C1QA	1.9572286	3.165396	5.3284741	4.27E-07	6.90E-06	5.8152113
CSF1R	1.8133661	6.3393394	8.3043888	1.17E-13	8.02E-12	20.560896
CYBB	3.0996452	6.7547591	10.518005	4.54E-19	8.33E-17	32.812709
MS4A6A	3.6332928	4.8210416	13.06732	2.17E-25	1.41E-22	47.1293
MARCO	2.493451	1.8232444	10.239743	2.22E-18	3.55E-16	31.249387
LILRA2	1.785648	4.773508	8.8251014	6.58E-15	5.82E-13	23.39035
MMP8	0.4825895	2.1774615	1.2191882	0.2249946	0.3714597	-6.261684
C1QB	0.9369498	2.7609898	2.4195981	0.01693	0.0511979	-4.139304
SPON2	-0.288398	3.5991016	-1.686416	0.0941296	0.1965857	-5.594481
IL7R	-0.036262	4.197528	-0.157701	0.8749388	0.9255441	-6.987843
PRR5L	-0.197802	3.5523947	-0.830036	0.4080499	0.5640868	-6.656874
NCR1	0.0525845	1.5478045	0.2922177	0.7705891	0.8576792	-6.957609
CRTAM	0.6293126	1.5968009	4.887975	2.97E-06	3.75E-05	3.947635
CCL4	-0.048188	2.5345371	-0.270802	0.7869755	0.8693542	-6.963631
CTLA4	0.3180816	1.387833	2.0848426	0.039053	0.0992864	-4.864042
SLAMF1	0.5814518	1.9687097	3.8168069	0.0002088	0.0013724	-0.10488
