# Supplementary material for: CD86 Is Associated with Immune Infiltration and Immunotherapy Signatures in AML and Promotes Its Progression
Source: J Oncol. 2023 Apr 7;2023:9988405. doi: 10.1155/2023/9988405 (PMC10104747; doi:10.1155/2023/9988405)
Supplement: Supplementary Materials — Supplementary Figure 1. CD86 mRNA expression in various cancers. Supplementary Figure 2. OS survival and prognosis of CD86 in high- and low-expression group in various cancers. Supplementary Figure 3. CD86 and univariate analysis in various cancers. Supplementary Figure 4. Heat and volcano maps showed the high and low expression of CD86, immune score and matrix score. A. Gene heat map and volcano map of CD86 high- and low-expression group; B. Gene heat map and volcano map of the high- and low-expression immune score group; C. Gene heat map and volcano map of high- and low-expression group with matrix score. Supplementary Figure 5. The PPI network, GO and KEGG analyzed of Mcode2. A. the PPI network diagram of Mcode2; B-C. Enrichment of the top ten of Go analysis; D-E. KEGG was used to analyze the top ten enrichment pathways. Supplementary Figure 6. The PPI network, GO and KEGG analyzed of Mcode4. A. the PPInetwork diagram of Mcode4; B-C. Enrichment of the top ten of Go analysis; D-E. KEGG was used to analyze the top ten enrichment pathways. Supplementary Figure 7. IRS different risk groups scores of survival prognosis in GEO data set were correlated with GSE146173, GSE106291, GSE37642 (the GPL96 platform), GSE12417 (the GPL97 platform), And GSE12417 (the GPL96 Platform) OS curve and ROC curve. Supplementary Table 1. Chemokines, immunostimulators, MHC proteins, and receptors in TISIDB website. Supplementary Table 2. Differences in expression of chemokines, immune stimulators, MHC proteins and immune receptors between CD86high-expression group and low expression group. Supplement Table 3. Differences between CD86 high- and low-expression group and CD8+ T cell recruitment, macrophage, NK cell, and Th1. Supplementary Table 4. Differential genes in StromalScore, ImmuneScore and CD86 high- and low-expression groups. Supplement Table 5. 65 genes screened out by Cox regression analysis. [file 9988405.f1.zip › 9988405.f1/Supplementary table 4 (1).pdf]

Supplementary Table 4: Differential genes in StromalScore, ImmuneScore and CD86 high-low expression groups

CD86_limma_res

	logFC	AveExpr	t	P.Value	adj.P.Val	B
CD86	3.18204747	3.890872624	16.48487438	1.45E-33	2.81E-29	65.60952262
ARHGEF10L	2.86437811	2.02562405	15.78819445	6.09E-32	5.93E-28	61.94410625
CLEC4A	2.737294976	3.669721727	15.68289461	1.08E-31	6.99E-28	61.38507548
SLC15A3	2.720140055	2.565575558	15.54980626	2.22E-31	1.08E-27	60.67668072
TLR8	3.795621285	3.367211846	15.20520009	1.45E-30	5.66E-27	58.83308663
TLR7	2.896875107	2.431583542	15.16411674	1.82E-30	5.91E-27	58.61241378
TLR5	2.644017815	2.382632377	14.87980143	8.68E-30	2.41E-26	57.0802405
FGD2	2.875012095	3.63376478	14.78492376	1.46E-29	3.56E-26	56.56702998
SAMHD1	2.905147697	7.041226851	14.70547878	2.27E-29	4.91E-26	56.13657602
SLC7A7	2.891797863	3.926749247	14.53247862	5.91E-29	1.05E-25	55.19697965
TLDC2	2.320448799	3.688747872	14.53149033	5.94E-29	1.05E-25	55.19160336
MPEG1	4.409773366	6.077885154	14.41231293	1.15E-28	1.87E-25	54.54256968
IGSF6	2.157071988	4.615148262	14.3568826	1.57E-28	2.19E-25	54.24022307
MEFV	3.252748053	3.011967381	14.3562065	1.57E-28	2.19E-25	54.23653343
ADAP2	2.617989984	3.31793606	14.34190697	1.70E-28	2.21E-25	54.15848673
SIGLEC9	3.160120269	3.413608415	14.32560219	1.86E-28	2.27E-25	54.0694713
RBM47	3.080798987	3.442842442	14.28536963	2.33E-28	2.67E-25	53.84971335
CTSS	2.349811896	7.500201545	14.16265085	4.62E-28	4.82E-25	53.17844814
MYCL	2.158820983	1.484927119	14.1592925	4.70E-28	4.82E-25	53.1600582
FGL2	3.305742798	5.399648057	14.04257238	9.02E-28	8.78E-25	52.52026358
KCTD12	3.524758931	4.685683084	14.02117848	1.02E-27	9.42E-25	52.40285874
SCIMP	2.730707382	3.739953669	13.77232177	4.09E-27	3.62E-24	51.03419459
CD101	2.442834885	2.635624261	13.69539975	6.30E-27	5.33E-24	50.61005284
CD1D	3.261614647	3.613320376	13.65828582	7.76E-27	6.30E-24	50.4052319
LILRB4	3.374586079	2.799017395	13.62173818	9.53E-27	7.42E-24	50.20342459
SIRPD	2.131570481	1.716863208	13.57482399	1.24E-26	9.29E-24	49.94421524
CTSZ	1.659276257	7.337766283	13.44698971	2.55E-26	1.84E-23	49.23701083
MS4A4A	2.998781623	3.116552624	13.26855003	6.97E-26	4.85E-23	48.24773004
CD300E	4.189415111	3.451381237	13.10277877	1.78E-25	1.19E-22	47.32658539
MS4A6A	3.633292819	4.821041588	13.06731991	2.17E-25	1.41E-22	47.12929971
POU2F2	2.063834388	5.268235315	12.96027341	3.99E-25	2.50E-22	46.53320082
CD14	4.258982927	4.724960659	12.92717434	4.81E-25	2.93E-22	46.34873269
CD300LB	2.872851603	3.30360258	12.8469797	7.58E-25	4.47E-22	45.90150122
IL1RN	2.696880085	3.106985169	12.83216101	8.24E-25	4.72E-22	45.81881591
NCF2	2.726345234	6.16819615	12.80427369	9.66E-25	5.37E-22	45.66317383
LRP1	3.384845137	4.014806711	12.76822462	1.19E-24	6.41E-22	45.46191028
SLC37A2	2.203712036	2.676983118	12.75903972	1.25E-24	6.57E-22	45.41061815
CCR1	2.894941413	4.37543277	12.7298863	1.47E-24	7.55E-22	45.24778088
CLEC7A	3.32407002	3.767697805	12.69220804	1.83E-24	9.11E-22	45.03725431
RNASE6	3.063764383	5.996590633	12.6468988	2.36E-24	1.15E-21	44.78398223
SIGLEC7	2.366681194	2.466187909	12.62687485	2.65E-24	1.26E-21	44.67201456
GPBAR1	2.170595198	2.655034188	12.5264299	4.68E-24	2.14E-21	44.11002829

LRRC25	3.023780491	4.601091751	12.52493632	4.72E-24	2.14E-21	44.10166771
AL049634.2	1.088301955	0.759371869	12.49458426	5.62E-24	2.48E-21	43.93174164
CD300C	2.66129357	4.224754744	12.43973757	7.67E-24	3.32E-21	43.62456367
NLRP12	2.805142429	3.048773692	12.33705864	1.38E-23	5.83E-21	43.049099
LILRA1	2.775736369	4.152441497	12.30400512	1.66E-23	6.89E-21	42.86374569
TNFRSF1B	2.680085284	6.132103482	12.24360077	2.35E-23	9.52E-21	42.52489256
CHST15	2.772243675	3.220297497	12.23079149	2.52E-23	1.00E-20	42.45301547
IFI30	2.263451423	2.217785321	12.20488898	2.93E-23	1.14E-20	42.30764695
IL13RA1	2.38893334	4.875219807	12.14601976	4.10E-23	1.56E-20	41.97716243
NCF1	2.765975341	3.496803105	12.13605807	4.34E-23	1.62E-20	41.92122505
1-Mar	1.772162103	3.907991349	12.09440528	5.50E-23	2.02E-20	41.68729311
CCR2	3.191802728	3.989666273	12.05027767	7.07E-23	2.55E-20	41.43939129
LY96	2.239550087	3.912163435	11.96561828	1.15E-22	4.06E-20	40.96359644
STX11	2.250550902	5.098080974	11.93729738	1.35E-22	4.69E-20	40.80437665
SLC11A1	3.068252004	3.668955998	11.88141697	1.86E-22	6.34E-20	40.49014436
CUEDC1	1.673031308	2.087713464	11.8404563	2.35E-22	7.87E-20	40.25975162
C9orf72	1.570326387	4.692087057	11.7723583	3.46E-22	1.14E-19	39.87661679
C19orf38	2.140806967	4.756748242	11.7681724	3.55E-22	1.15E-19	39.85306201
KLF4	2.956170529	4.945466196	11.76529707	3.61E-22	1.15E-19	39.83688182
LILRB1	2.453946658	3.486813013	11.68077828	5.85E-22	1.84E-19	39.36118545
SULF2	2.42228661	3.940552996	11.62440289	8.07E-22	2.49E-19	39.04380175
SIRPB1	2.859975617	3.958171753	11.60577854	8.98E-22	2.73E-19	38.93893619
VCAN	4.213490866	5.315015266	11.56243564	1.15E-21	3.45E-19	38.69486704
LILRB2	3.205617317	4.319628714	11.55458104	1.20E-21	3.55E-19	38.65063343
LGALS2	3.477046661	3.003776548	11.45522049	2.13E-21	6.17E-19	38.09099939
ASGR2	2.486962483	2.342679339	11.44135915	2.30E-21	6.59E-19	38.01291698
MS4A14	2.439589792	3.232899066	11.43203633	2.43E-21	6.85E-19	37.96039938
NLRC4	1.995356388	3.335477271	11.38598798	3.16E-21	8.78E-19	37.70098488
MAFB	3.459582915	3.802632868	11.38147068	3.24E-21	8.89E-19	37.67553545
ADA2	2.060104253	6.704439663	11.36776048	3.51E-21	9.48E-19	37.59829435
SLC43A2	1.99008467	3.388876315	11.36029048	3.66E-21	9.76E-19	37.5562089
FGR	2.993100232	6.247745024	11.30047574	5.16E-21	1.36E-18	37.2192026
HK3	3.468402772	4.349380146	11.29556825	5.30E-21	1.38E-18	37.19155205
COTL1	1.712896894	6.377000959	11.23417463	7.54E-21	1.93E-18	36.84563
NAGA	1.523535043	5.860961359	11.18632617	9.91E-21	2.50E-18	36.57602204
CSF2RA	1.829822799	5.141437202	11.17237164	1.07E-20	2.68E-18	36.49739333
TNFSF15	0.682572868	0.509485973	11.16149432	1.14E-20	2.81E-18	36.43610372
SLC46A2	1.55584724	1.531478475	11.1114717	1.52E-20	3.67E-18	36.15424776
SLC6A12	1.177282346	0.789451266	11.11084813	1.53E-20	3.67E-18	36.15073428
MYOF	2.644892332	2.827258831	11.10390651	1.59E-20	3.77E-18	36.11162182
PILRA	1.893059347	5.048142853	11.07508532	1.87E-20	4.38E-18	35.94923152
LILRA5	3.362182477	3.447420747	11.07367894	1.89E-20	4.38E-18	35.94130751
DTX4	1.897800654	2.660175077	11.06159705	2.02E-20	4.63E-18	35.87323455
LILRA6	2.596859398	2.622608198	11.05015844	2.16E-20	4.89E-18	35.80878686
C5AR1	3.306824661	4.493231657	11.02587177	2.48E-20	5.56E-18	35.67195319
PSAP	1.630442414	9.466183206	10.99516195	2.96E-20	6.55E-18	35.49893745
OSBPL11	1.208181739	4.487273237	10.9384167	4.10E-20	8.96E-18	35.17926474

FCER1G	2.490632121	7.540687066	10.91131094	4.79E-20	1.03E-17	35.0265783
JAML	2.669936064	4.187177829	10.89940842	5.12E-20	1.10E-17	34.95953459
LILRB3	2.360769286	3.663464921	10.8944301	5.27E-20	1.11E-17	34.93149358
UNC5A	1.799457837	1.401855363	10.88383892	5.60E-20	1.17E-17	34.87183869
SLC8A1	1.787751175	1.386920272	10.82244161	7.96E-20	1.65E-17	34.52605234
VDR	1.926848008	2.954289216	10.72901954	1.36E-19	2.78E-17	34.00003696
MBD2	0.75129893	5.096147659	10.72411506	1.40E-19	2.83E-17	33.97242721
RAB39A	1.521008448	1.329752205	10.67515335	1.85E-19	3.71E-17	33.69682899
S100A9	4.288401307	9.319288163	10.64848165	2.15E-19	4.27E-17	33.54672314
MS4A7	2.315517742	4.713307771	10.6467293	2.17E-19	4.27E-17	33.53686176
NAIP	1.724583354	2.530954584	10.6452569	2.19E-19	4.27E-17	33.52857584
MTMR11	2.487589197	2.109279975	10.61646347	2.59E-19	4.98E-17	33.36655319
LRRK2	2.417984586	2.52631382	10.60184721	2.81E-19	5.36E-17	33.28431549
PLBD1	3.427189703	3.923940727	10.59704442	2.89E-19	5.46E-17	33.25729417
TNFSF10	1.847834516	4.758249823	10.53269778	4.17E-19	7.81E-17	32.89533717
KIAA0513	1.902119899	3.393287331	10.52092612	4.46E-19	8.27E-17	32.82913464
CYBB	3.099645166	6.754759134	10.51800526	4.54E-19	8.33E-17	32.81270877
MEGF9	1.7870733	5.259485285	10.49185201	5.27E-19	9.59E-17	32.66564538
ITGAM	2.354192563	5.560710542	10.47521004	5.80E-19	1.04E-16	32.57207776
CCR5	2.066127503	2.615431007	10.47043415	5.96E-19	1.06E-16	32.54522772
ZNF385A	1.76347798	5.513079175	10.46886754	6.01E-19	1.06E-16	32.53642041
CXCL16	2.20166537	2.879244845	10.44970646	6.71E-19	1.18E-16	32.4287063
S100A4	1.700360247	8.209092696	10.43236221	7.41E-19	1.29E-16	32.33121746
LY86	2.934145998	5.038448468	10.36595849	1.08E-18	1.86E-16	31.95808446
ACPP	1.860354621	3.363903568	10.33958298	1.26E-18	2.15E-16	31.80992729
GABARAP	0.877056688	5.055255466	10.33281553	1.31E-18	2.21E-16	31.77191789
SRGAP2	1.224092136	3.871591001	10.33156665	1.32E-18	2.21E-16	31.76490374
IL10RA	2.07572509	4.86945179	10.31295527	1.46E-18	2.44E-16	31.66038445
GASK1B	2.536254016	3.242688766	10.306868	1.52E-18	2.49E-16	31.62620245
ITGB2	1.979044123	7.508271431	10.30586125	1.52E-18	2.49E-16	31.62054941
SECTM1	2.634918742	2.843133595	10.2917457	1.65E-18	2.68E-16	31.54129324
CD4	2.089177026	5.787242216	10.28330847	1.73E-18	2.79E-16	31.49392422
MARCO	2.493450985	1.82324443	10.23974264	2.22E-18	3.55E-16	31.24938735
HVCN1	1.498915241	4.424530386	10.23687685	2.26E-18	3.58E-16	31.2333048
PTPRO	1.636359687	1.852962199	10.2215214	2.47E-18	3.87E-16	31.14713843
LYZ	3.463545538	10.98525357	10.21517191	2.56E-18	3.98E-16	31.11151212
SIRPA	1.425307342	5.722664984	10.20426461	2.72E-18	4.20E-16	31.05031717
RASSF4	2.077273021	4.12388081	10.1872416	3.00E-18	4.60E-16	30.95482262
ZDHHC7	0.869896225	5.775033454	10.1833671	3.07E-18	4.66E-16	30.93308985
HEXB	1.181238864	5.337256006	10.1707654	3.29E-18	4.97E-16	30.86241014
PTAFR	2.370962434	4.962578672	10.16646218	3.38E-18	5.05E-16	30.83827644
APOA2	1.761114338	2.219255462	10.16399247	3.42E-18	5.07E-16	30.82442601
IRF8	2.666330937	5.103193362	10.16251068	3.45E-18	5.07E-16	30.81611612
P2RY13	2.436966793	4.020890502	10.16186944	3.47E-18	5.07E-16	30.8125201
TYMP	2.144370103	5.257839545	10.13672391	4.00E-18	5.81E-16	30.67152316
OAS1	1.76948904	3.819790372	10.05832319	6.25E-18	9.01E-16	30.23214293
S100A8	4.063328511	7.835949218	10.04996683	6.56E-18	9.38E-16	30.18533293

CPPED1	1.258772498	5.054380153	10.02620995	7.51E-18	1.07E-15	30.05227698
THEMIS2	1.570023278	6.067769506	10.01510247	8.00E-18	1.13E-15	29.99007904
SCPEP1	2.298982684	5.313692399	9.999215053	8.75E-18	1.23E-15	29.90112868
FKBP15	1.014488867	4.845502874	9.980555355	9.73E-18	1.35E-15	29.79667766
CASP1	1.545714536	5.3299624	9.967251855	1.05E-17	1.45E-15	29.7222228
CLEC1A	0.732915471	0.519701502	9.958822835	1.10E-17	1.51E-15	29.6750547
CARD16	1.269757546	4.021903748	9.95549142	1.12E-17	1.53E-15	29.65641369
SGK1	1.989927757	2.824965197	9.914738908	1.42E-17	1.91E-15	29.42844287
SRGAP2B	0.819023193	1.249030641	9.900531397	1.53E-17	2.06E-15	29.34899256
TFEB	1.548273803	3.251001517	9.898315587	1.55E-17	2.07E-15	29.33660273
DOK2	1.758605705	4.973141898	9.893486365	1.60E-17	2.11E-15	29.30960106
CX3CR1	2.914939376	4.945756337	9.872408181	1.80E-17	2.37E-15	29.19176589
C1orf162	1.705060638	5.92191534	9.870777335	1.82E-17	2.37E-15	29.18265016
GPR65	1.628447704	2.970233865	9.867512458	1.85E-17	2.40E-15	29.16440147
PLD2	1.63516889	2.516458522	9.840549572	2.16E-17	2.78E-15	29.01372511
UBXN11	1.418622007	5.094976191	9.808963109	2.58E-17	3.31E-15	28.83727968
AP1S2	1.571555792	5.05034129	9.778980804	3.06E-17	3.89E-15	28.66986571
GIMAP1	1.440047119	3.270798128	9.751086304	3.59E-17	4.53E-15	28.51417272
NADK	1.098750132	5.62475828	9.734123583	3.95E-17	4.96E-15	28.41952579
FCN1	3.276862928	5.620795074	9.727650221	4.10E-17	5.11E-15	28.3834125
ATP6V0D1	0.98860227	4.981982177	9.694596471	4.94E-17	6.12E-15	28.19906756
KCNQ1	1.913712077	3.175357884	9.683962659	5.25E-17	6.46E-15	28.13978082
BCL6	2.058933751	4.502893014	9.674209522	5.54E-17	6.79E-15	28.08541252
CD163	3.30996502	3.301821017	9.649148291	6.39E-17	7.77E-15	27.94574746
NLRP3	1.504726832	4.987100716	9.635957759	6.89E-17	8.32E-15	27.87225911
PRKACA	0.879357594	4.735777721	9.628045639	7.20E-17	8.65E-15	27.82818567
UNC93B1	1.386709383	5.499997075	9.604792715	8.22E-17	9.81E-15	27.69869043
ARPC1B	1.092910932	7.015667008	9.595029631	8.68E-17	1.03E-14	27.64433437
CDA	2.852000692	4.441763504	9.517282919	1.35E-16	1.59E-14	27.21179237
HMOX1	2.417412737	3.843200019	9.489128004	1.58E-16	1.85E-14	27.05529449
FAM20C	1.882903583	2.611780418	9.477508268	1.69E-16	1.97E-14	26.99072922
CTSB	1.391410585	6.799956979	9.451504975	1.95E-16	2.26E-14	26.84628991
MNDA	2.45050637	7.196184452	9.420981062	2.32E-16	2.67E-14	26.67682695
ITGA7	1.723213163	2.464233437	9.411052396	2.46E-16	2.81E-14	26.6217254
CLEC10A	2.613839652	2.091705368	9.39469717	2.69E-16	3.07E-14	26.53098035
P2RX7	1.455060921	4.362406599	9.367365455	3.14E-16	3.56E-14	26.37939612
ADAP1	1.345109492	3.353238454	9.357844337	3.32E-16	3.73E-14	26.32660972
CD68	1.160076127	1.972828693	9.341734764	3.63E-16	4.06E-14	26.23731817
SGSH	1.813412125	2.999590106	9.335089438	3.77E-16	4.19E-14	26.20049288
NPC2	0.957784914	6.122565241	9.328251964	3.92E-16	4.33E-14	26.16260781
HTR7	1.760774261	1.453878532	9.324757005	4.00E-16	4.39E-14	26.14324491
NOD2	2.049422072	3.361229903	9.322445606	4.05E-16	4.43E-14	26.13043995
IL31RA	1.30860862	0.800184855	9.320824836	4.09E-16	4.44E-14	26.12146137
IFNGR1	1.213193955	6.613125242	9.306766135	4.42E-16	4.78E-14	26.0435925
MAP2K1	0.880658956	5.353249431	9.264908356	5.60E-16	6.02E-14	25.81187959
CFP	1.783589296	5.439347941	9.252093027	6.02E-16	6.43E-14	25.74097718
EMID1	-2.017618717	4.730993841	-9.237229489	6.54E-16	6.96E-14	25.65876644

SRGAP2C	0.827159432	2.2190975	9.233816033	6.67E-16	7.05E-14	25.6398901
MYD88	1.065477781	6.81040454	9.216122	7.37E-16	7.75E-14	25.54206426
TMEM132A	0.683299757	0.581457498	9.21351397	7.47E-16	7.82E-14	25.52764822
IER3	1.938093283	2.800883896	9.194777036	8.30E-16	8.60E-14	25.42410234
EVI2B	1.143445371	8.239165693	9.151261501	1.06E-15	1.09E-13	25.18378465
MSR1	1.819133606	1.175253943	9.139103898	1.14E-15	1.16E-13	25.11668454
GNS	1.210826294	6.338024125	9.110684583	1.33E-15	1.36E-13	24.95990427
TLR4	2.132524102	4.863340009	9.104542462	1.38E-15	1.40E-13	24.9260334
TNFAIP2	2.16568954	5.991487958	9.100252355	1.41E-15	1.42E-13	24.90237832
CRISPLD2	1.906017248	2.270341399	9.100074124	1.41E-15	1.42E-13	24.90139563
PEA15	0.921904878	5.611009231	9.093491082	1.47E-15	1.46E-13	24.86510228
ARHGAP30	0.879042655	6.895900002	9.073897619	1.64E-15	1.63E-13	24.75711299
ABI3	1.875594614	3.334619586	9.072180864	1.65E-15	1.63E-13	24.74765344
TIGAR	1.074961199	3.15987954	9.069177388	1.68E-15	1.65E-13	24.73110479
DPYD	1.618364087	4.926667897	9.061573088	1.75E-15	1.72E-13	24.68921156
RASSF3	0.913473759	5.580033678	9.011329416	2.32E-15	2.25E-13	24.41260088
VSIR	1.786730824	7.03775136	8.981986304	2.74E-15	2.64E-13	24.25121043
GIMAP8	1.997004167	3.311034213	8.969911911	2.93E-15	2.81E-13	24.18483361
TBC1D2	1.208379151	4.769329342	8.968581821	2.95E-15	2.82E-13	24.17752288
CAPN2	1.488627557	5.179673564	8.950928397	3.26E-15	3.09E-13	24.08051532
ARRB2	1.143340424	6.792434636	8.947349528	3.32E-15	3.14E-13	24.06085424
ANXA5	2.407784662	5.291382602	8.944060252	3.39E-15	3.18E-13	24.04278565
DHRS9	2.217163272	2.857617515	8.931792301	3.63E-15	3.38E-13	23.97540877
TMEM127	0.676623173	5.737906135	8.931642891	3.63E-15	3.38E-13	23.97458832
PTPRE	1.194085716	5.477677298	8.92742838	3.72E-15	3.44E-13	23.9514467
SOAT1	0.872523165	4.834296189	8.910195077	4.09E-15	3.77E-13	23.85684552
LGALS3	2.054065429	4.435377141	8.909645472	4.10E-15	3.77E-13	23.85382918
MAP3K3	0.88099827	5.553838743	8.904000701	4.24E-15	3.87E-13	23.82285206
CTSH	1.496549924	4.45395517	8.894110261	4.48E-15	4.07E-13	23.76858662
KMO	0.917663359	1.929862187	8.86523961	5.26E-15	4.76E-13	23.6102631
FPR1	2.873638081	3.767089206	8.862327495	5.34E-15	4.81E-13	23.59430002
KCNE1	1.657017744	1.811483873	8.844691666	5.90E-15	5.29E-13	23.49765361
FCGR2A	2.003525712	4.491287686	8.838868071	6.09E-15	5.44E-13	23.46574958
LILRA2	1.785647984	4.77350802	8.825101378	6.58E-15	5.82E-13	23.3903498
RNF135	0.775490665	4.899659455	8.824915457	6.58E-15	5.82E-13	23.38933171
USP3	0.812622977	5.167163092	8.819457625	6.79E-15	5.98E-13	23.35944721
FBP1	2.362977858	3.899805908	8.796648201	7.71E-15	6.76E-13	23.23460148
PRLR	1.647464957	1.188476145	8.794081175	7.82E-15	6.82E-13	23.22055591
ARHGAP24	1.247447504	1.541763167	8.788064784	8.08E-15	7.02E-13	23.18764091
NPTN	0.688872292	5.329660546	8.785859387	8.18E-15	7.08E-13	23.1755768
ARHGAP26	1.14412492	4.232097788	8.784427705	8.25E-15	7.10E-13	23.1677455
S100A12	3.718381071	5.358097062	8.776011083	8.65E-15	7.41E-13	23.12171288
LACTB	0.804933113	4.17897374	8.771291478	8.88E-15	7.54E-13	23.09590488
SGMS2	2.012691005	2.44308821	8.757293477	9.59E-15	8.12E-13	23.01938016
SLC12A9	1.159384358	4.336475342	8.752166627	9.87E-15	8.32E-13	22.99136003
ALOX5AP	2.091458725	5.747483354	8.737493474	1.07E-14	8.98E-13	22.91118815
AQP9	2.529661077	2.724124409	8.715447194	1.21E-14	1.01E-12	22.79079332

HCK	1.9567191	6.00499591	8.701345657	1.31E-14	1.09E-12	22.71382461
MSANTD3	-0.745063841	3.349751704	-8.694489963	1.36E-14	1.13E-12	22.67641631
GLIPR2	1.452596767	5.75363068	8.692529424	1.38E-14	1.13E-12	22.66571994
TNFSF13	1.175854394	4.107617136	8.685545802	1.43E-14	1.17E-12	22.62762341
AOAH	1.846664422	6.124721998	8.672410538	1.54E-14	1.25E-12	22.55598995
EHD4	1.274366614	3.221929073	8.660001235	1.65E-14	1.34E-12	22.48834089
SHTN1	1.914482635	1.810420318	8.653717065	1.71E-14	1.38E-12	22.45409232
SIGLEC11	1.22414827	0.919149087	8.644488542	1.80E-14	1.44E-12	22.40380866
FGD4	1.8639103	3.06556247	8.638554113	1.86E-14	1.49E-12	22.37148091
LFNG	1.659608324	4.239876057	8.633552976	1.91E-14	1.52E-12	22.34424171
ZBTB7B	1.362973698	5.450325155	8.631782295	1.93E-14	1.53E-12	22.33459849
RIN2	1.629933096	2.534822554	8.626998098	1.98E-14	1.56E-12	22.30854609
SLC66A3	0.792256795	5.435098051	8.618775233	2.07E-14	1.63E-12	22.26377713
MCEMP1	2.011582878	3.912467619	8.604223063	2.24E-14	1.76E-12	22.1845759
PKIB	1.403235367	1.289237565	8.574768094	2.64E-14	2.06E-12	22.02437277
GLTP	0.669898186	4.796375396	8.570130328	2.71E-14	2.11E-12	21.99916158
MS4A4E	2.491347368	4.989269457	8.565596012	2.78E-14	2.16E-12	21.97451626
SLC31A1	0.934749695	4.050980923	8.56398235	2.80E-14	2.17E-12	21.96574638
S100A6	1.73044616	7.328031731	8.542781724	3.15E-14	2.43E-12	21.85056688
ATG3	0.809144219	5.264297105	8.541098675	3.18E-14	2.44E-12	21.84142643
FCGR2B	1.338255647	1.712670385	8.532345856	3.34E-14	2.55E-12	21.79389869
GIMAP4	2.122273075	4.412642555	8.52873279	3.41E-14	2.59E-12	21.77428361
RAB31	2.225709506	4.434589745	8.524584627	3.49E-14	2.64E-12	21.75176628
GM2A	0.893575596	5.823188851	8.515255827	3.67E-14	2.76E-12	21.70113794
MTMR14	0.647828348	5.279052807	8.515244265	3.67E-14	2.76E-12	21.7010752
GRN	1.697572542	8.333753343	8.504066238	3.91E-14	2.92E-12	21.6404308
CD209	1.069101063	0.798878388	8.471659386	4.67E-14	3.47E-12	21.4647366
RCN3	1.609067644	3.033534293	8.443876452	5.45E-14	4.03E-12	21.31425886
EVI2A	1.121275248	6.220863724	8.426778481	5.98E-14	4.41E-12	21.22172164
UBE2D1	1.033392998	4.663274446	8.416287794	6.34E-14	4.66E-12	21.16497033
NFAM1	2.413022396	4.829114395	8.41064726	6.54E-14	4.78E-12	21.13446508
IL17RA	1.650986144	6.196942285	8.403137984	6.82E-14	4.97E-12	21.09386226
MFSD2A	1.36556328	2.56398814	8.388253818	7.40E-14	5.37E-12	21.01341376
IFNGR2	0.986204027	5.898829393	8.380487245	7.72E-14	5.58E-12	20.97145183
CPM	1.825113073	2.211191747	8.379329677	7.77E-14	5.58E-12	20.96519858
IFNK	1.122795439	1.430294495	8.379204073	7.78E-14	5.58E-12	20.96452007
MVP	1.159219751	5.112826719	8.376822818	7.88E-14	5.64E-12	20.95165721
STS	1.388315644	3.004809289	8.36854508	8.24E-14	5.88E-12	20.90695141
FAM49A	1.552706459	4.219680262	8.367755091	8.28E-14	5.88E-12	20.90268556
GPR132	1.328166858	3.868632396	8.359759037	8.65E-14	6.12E-12	20.85951432
ANXA2	1.975437861	5.247050435	8.357493828	8.76E-14	6.18E-12	20.84728648
NAAA	1.007843558	4.83813799	8.340886683	9.60E-14	6.74E-12	20.75766862
TYROBP	1.642795383	8.420192138	8.336024381	9.86E-14	6.90E-12	20.73143977
IQSEC2	1.401022393	2.861766531	8.334105085	9.96E-14	6.95E-12	20.72108769
KDM5B	-1.046126419	5.705007534	-8.33133493	1.01E-13	7.03E-12	20.70614757
STX12	0.666971035	5.003269211	8.31535704	1.10E-13	7.65E-12	20.62000331
NLRP1	1.477505636	5.010788772	8.311157075	1.13E-13	7.80E-12	20.59736739

MYO1F	1.111681085	6.912238258	8.310008796	1.14E-13	7.82E-12	20.59117928
CSF1R	1.813366093	6.339339396	8.304388757	1.17E-13	8.02E-12	20.56089631
FBXL5	0.825157036	6.50106245	8.304226876	1.17E-13	8.02E-12	20.56002412
NACC2	1.573613634	3.554447265	8.301234584	1.19E-13	8.12E-12	20.54390304
CAP1	0.830232994	8.214306905	8.29588876	1.23E-13	8.32E-12	20.5151065
KCNK13	0.724298798	0.419936514	8.295452475	1.23E-13	8.32E-12	20.51275659
BST1	1.609232054	4.817481794	8.294581553	1.24E-13	8.33E-12	20.50806576
GUCY2C	0.739946306	0.469180177	8.292325116	1.25E-13	8.41E-12	20.49591314
OSTF1	0.863234387	6.633833229	8.28863516	1.28E-13	8.55E-12	20.47604206
DAPP1	1.248273629	4.797974564	8.270456955	1.41E-13	9.41E-12	20.37818757
PTPRJ	1.698562135	4.429822461	8.269931483	1.42E-13	9.41E-12	20.37535987
FCGRT	1.716431571	5.530985589	8.263821671	1.47E-13	9.70E-12	20.34248538
SIGLEC1	2.180399765	1.859803923	8.254203973	1.54E-13	1.02E-11	20.2907511
UNC119	1.01318381	3.949262843	8.248060357	1.60E-13	1.05E-11	20.25771363
TLR1	1.364709788	4.200024446	8.243173739	1.64E-13	1.08E-11	20.231441
ODF3B	1.653701249	4.003443613	8.239825851	1.67E-13	1.09E-11	20.21344398
HSPA6	1.406435898	1.778903607	8.238526188	1.68E-13	1.10E-11	20.20645806
FLVCR2	1.184127799	2.515657	8.236853105	1.70E-13	1.10E-11	20.19746544
PAK1	1.193940606	5.556851513	8.236211061	1.70E-13	1.10E-11	20.19401468
OGFRL1	1.268681533	4.912040448	8.23038013	1.76E-13	1.13E-11	20.16267918
ITGAL	1.45920057	6.810286802	8.226927665	1.79E-13	1.15E-11	20.14412876
HORMAD1	1.223617131	1.475646376	8.223518528	1.83E-13	1.17E-11	20.12581347
FCGR1B	1.277876709	1.772438313	8.214576874	1.92E-13	1.22E-11	20.07778619
TNFRSF8	1.236970115	1.516936533	8.197525267	2.11E-13	1.34E-11	19.98624313
METTL9	0.807257533	5.780470408	8.161583414	2.56E-13	1.63E-11	19.79347813
ARPC5	0.815001692	5.605474429	8.154509812	2.66E-13	1.68E-11	19.75557162
GPCPD1	0.85160654	6.065804439	8.141844257	2.86E-13	1.80E-11	19.6877242
ZNF273	-0.971987022	3.143555416	-8.117096505	3.27E-13	2.05E-11	19.5552499
ZNF529	-0.773027543	4.19175227	-8.114090445	3.32E-13	2.08E-11	19.53916717
ITGAX	1.951441361	5.574569174	8.112308781	3.35E-13	2.09E-11	19.52963598
RGS2	2.185197382	5.67184092	8.111218643	3.37E-13	2.10E-11	19.52380451
ACVRL1	1.038267764	0.99021796	8.10556689	3.48E-13	2.16E-11	19.49357556
SH2B3	0.837359317	6.301788747	8.100532596	3.58E-13	2.21E-11	19.46665479
VNN2	2.233862656	3.797260043	8.09192176	3.75E-13	2.31E-11	19.42062087
SLC2A9	1.268287521	2.689136643	8.072808331	4.16E-13	2.55E-11	19.31849552
NAGK	0.792203905	5.247299873	8.069235017	4.24E-13	2.60E-11	19.29941147
VNN3	1.627498709	1.52826499	8.063843662	4.37E-13	2.66E-11	19.27062292
CDH23	1.103607254	1.753270731	8.061264397	4.43E-13	2.69E-11	19.25685245
OTUD1	1.119191467	4.231977993	8.060941024	4.44E-13	2.69E-11	19.25512609
FCGR1A	1.569786158	3.174202038	8.056716406	4.54E-13	2.74E-11	19.23257458
CDC42EP3	1.597468195	4.271784698	8.050527647	4.70E-13	2.83E-11	19.19954517
ZSWIM6	0.903493793	4.711825204	8.045467778	4.83E-13	2.90E-11	19.17254674
DUSP6	1.786473722	6.221837491	8.023760897	5.43E-13	3.24E-11	19.05678585
MOB3A	0.849043215	6.726632632	8.01620961	5.66E-13	3.37E-11	19.01653943
ARPC4	0.587042627	5.881482007	8.003163619	6.08E-13	3.60E-11	18.94703691
TBC1D9	1.540802193	2.592882436	7.991462517	6.47E-13	3.83E-11	18.88473098
F5	1.686127123	2.113048935	7.989667824	6.54E-13	3.86E-11	18.87517727

IL10RB	0.786348164	5.803915927	7.981697588	6.83E-13	4.01E-11	18.83275781
HCLS1	0.738368961	7.022324465	7.968202957	7.34E-13	4.31E-11	18.76096826
EFHD2	1.004374209	7.37431481	7.967318028	7.38E-13	4.31E-11	18.75626197
CDK6	-1.169560325	7.442171529	-7.961279723	7.63E-13	4.44E-11	18.7241533
ZNF10	-0.848013103	2.960462244	-7.955845386	7.85E-13	4.56E-11	18.69526319
ZNF875	-0.626872423	4.471518727	-7.944849594	8.34E-13	4.81E-11	18.63682735
LST1	1.734120029	5.94027799	7.944826295	8.34E-13	4.81E-11	18.63670356
PIK3R5	1.094451726	4.979591384	7.935428348	8.77E-13	5.05E-11	18.58678083
FRAT1	0.964277338	4.289900507	7.934807942	8.80E-13	5.05E-11	18.58348588
RHBDF2	1.234730059	4.767639266	7.923387041	9.36E-13	5.34E-11	18.52284543
BRI3	0.896815867	4.142097122	7.918636941	9.61E-13	5.47E-11	18.49763297
ASGR1	0.848553164	1.500019672	7.916740866	9.71E-13	5.51E-11	18.48757046
LIPN	1.640071403	1.935434975	7.8959436	1.09E-12	6.14E-11	18.37725266
NAMPT	1.596537725	4.648581306	7.888596539	1.13E-12	6.38E-11	18.33830427
CST3	2.090614574	6.849885462	7.880139771	1.18E-12	6.65E-11	18.29348843
AGTRAP	1.257688334	5.675226034	7.862750701	1.30E-12	7.29E-11	18.20138856
LTA4H	1.006612189	6.13045807	7.861850194	1.31E-12	7.30E-11	18.19662101
FRAT2	0.866973716	5.237327918	7.856430398	1.34E-12	7.50E-11	18.16793094
IL4I1	1.092336952	1.333752756	7.855637759	1.35E-12	7.51E-11	18.16373562
PPM1M	1.089086671	4.674538714	7.850421437	1.39E-12	7.70E-11	18.13613008
RASSF2	0.966710329	6.725843193	7.841014874	1.46E-12	8.08E-11	18.08636523
PPT2	-0.924547859	3.893757702	-7.830531643	1.55E-12	8.52E-11	18.03092875
CD93	2.238873333	5.066013782	7.825462934	1.59E-12	8.74E-11	18.00413413
CREB5	1.511537021	2.288509032	7.81931113	1.64E-12	9.01E-11	17.9716221
IQGAP1	1.172195212	6.465019587	7.80494245	1.78E-12	9.70E-11	17.89571911
AL928654.[illegible]	0.95211165	1.122376697	7.80053392	1.82E-12	9.91E-11	17.87244075
CD36	2.26149908	4.978541522	7.797136188	1.85E-12	1.01E-10	17.85450285
ADGRE3	1.744311248	1.892010387	7.788505382	1.94E-12	1.05E-10	17.80895
AKIRIN2	0.601206521	6.411861219	7.777565343	2.06E-12	1.11E-10	17.7512348
CACNA2D3	1.513208729	1.810206296	7.771310161	2.13E-12	1.14E-10	17.71824792
CD180	1.930883936	4.719905431	7.766655965	2.18E-12	1.17E-10	17.69371
IRF5	1.012890148	5.830236857	7.762929377	2.23E-12	1.19E-10	17.6740664
WARS	1.026212804	5.771233178	7.749722738	2.39E-12	1.27E-10	17.60447861
BBOF1	-0.655884823	2.032859034	-7.738981325	2.53E-12	1.35E-10	17.54791172
MYO7A	1.435294512	1.324821171	7.731140502	2.64E-12	1.40E-10	17.50663782
SLC31A2	1.010737157	2.554655528	7.717480963	2.84E-12	1.50E-10	17.43477034
ZNF461	-0.607770238	3.089933572	-7.715268817	2.87E-12	1.52E-10	17.42313581
SORT1	2.331558674	3.576674497	7.713894597	2.90E-12	1.52E-10	17.41590886
NUMB	0.649541782	5.475464827	7.713377533	2.90E-12	1.52E-10	17.41318976
PTPN18	0.867373683	6.231498401	7.709799596	2.96E-12	1.55E-10	17.39437622
PUDP	0.888538982	3.557066386	7.693980265	3.22E-12	1.68E-10	17.31123284
CXorf21	1.282202537	6.084469411	7.692115199	3.25E-12	1.69E-10	17.3014345
ZMYND15	0.950074712	0.843572233	7.680692364	3.46E-12	1.80E-10	17.24144222
PIK3AP1	0.924687109	6.522425452	7.674501279	3.58E-12	1.85E-10	17.2089405
FCAR	2.266738577	3.726637299	7.672294668	3.62E-12	1.87E-10	17.19735864
SPI1	0.967490748	7.805695382	7.670407892	3.66E-12	1.88E-10	17.18745646
ACTB	0.733907899	11.84515227	7.665213117	3.76E-12	1.93E-10	17.16019788

SMAP2	0.945012253	7.363702843	7.661426671	3.84E-12	1.96E-10	17.1403335
CD1A	0.815341521	0.643199317	7.6582657	3.90E-12	1.99E-10	17.12375324
FCER2	1.829065648	3.23790388	7.656715028	3.93E-12	2.00E-10	17.11562041
S100A11	1.634324642	8.116365333	7.646830421	4.15E-12	2.11E-10	17.06379277
ALDH5A1	-1.063731075	4.385145854	-7.644478482	4.20E-12	2.13E-10	17.05146457
SPOCK1	1.121901714	0.784689594	7.623222572	4.71E-12	2.38E-10	16.94011078
LAPTM5	0.969819397	9.662457066	7.612799286	4.98E-12	2.51E-10	16.88554823
ACER3	0.787588874	4.579518147	7.597053857	5.41E-12	2.72E-10	16.80317887
TMEM170B	1.43450087	4.776909936	7.59665479	5.42E-12	2.72E-10	16.80109205
TFDP2	-1.008538261	5.575139698	-7.586137488	5.74E-12	2.86E-10	16.7461094
SMIM14	1.165591525	3.394846774	7.573730232	6.13E-12	3.05E-10	16.68128329
SYK	0.771290759	7.190146149	7.569111573	6.28E-12	3.12E-10	16.65716169
SNX27	0.641009295	5.280422108	7.560633262	6.57E-12	3.25E-10	16.61289702
PLD6	-0.79459325	2.947256086	-7.559512658	6.61E-12	3.27E-10	16.60704783
ZNF8	-0.712704205	3.954527042	-7.552708352	6.86E-12	3.38E-10	16.57153863
MYBPH	1.334398363	0.907683858	7.551288624	6.91E-12	3.39E-10	16.56413112
TMSB4X	0.785251548	9.891855347	7.545835786	7.11E-12	3.49E-10	16.53568555
GPR183	1.321300286	5.881356274	7.538433987	7.40E-12	3.62E-10	16.49708546
PLEKHO2	0.836498112	5.426521826	7.537624521	7.43E-12	3.62E-10	16.49286501
ZNF703	1.389193294	1.678454142	7.530311018	7.72E-12	3.76E-10	16.45474114
CD200R1	1.309241952	2.099388365	7.524747347	7.96E-12	3.86E-10	16.4257483
BCKDHB	-0.642643368	4.00574761	-7.520534037	8.14E-12	3.94E-10	16.4037978
EHBP1L1	0.958755974	6.17191974	7.509214133	8.64E-12	4.17E-10	16.34484673
PJVK	-0.670567443	2.128649171	-7.507809541	8.71E-12	4.19E-10	16.33753437
CMBL	-1.218812433	3.843238225	-7.504243132	8.87E-12	4.26E-10	16.31896988
UPK3A	1.147394449	0.888237028	7.502442545	8.96E-12	4.29E-10	16.30959844
MGAT1	0.667838413	6.207336938	7.501659384	8.99E-12	4.30E-10	16.30552263
KIAA0930	0.854389764	5.667695376	7.498812685	9.13E-12	4.36E-10	16.29070891
RPGRIP1	0.696137294	1.063012381	7.490644487	9.54E-12	4.54E-10	16.24821511
FFAR2	1.473940708	1.744588345	7.487524923	9.70E-12	4.60E-10	16.23199078
NDUFAF6	-0.686031275	3.969244202	-7.474684281	1.04E-11	4.91E-10	16.16523639
C9orf43	-1.530458427	3.183069606	-7.46650099	1.08E-11	5.12E-10	16.12271734
SERPINB2	2.155308026	2.753633584	7.458184732	1.13E-11	5.34E-10	16.07952603
SNX18	0.814728306	4.369589038	7.453436321	1.16E-11	5.46E-10	16.05487312
CCDC69	0.876696247	5.761811834	7.444940081	1.22E-11	5.69E-10	16.01077751
ZNF571	-0.845392652	2.542803688	-7.443125588	1.23E-11	5.73E-10	16.00136282
KYNU	1.475252986	2.184306621	7.439079868	1.25E-11	5.84E-10	15.98037442
GNGT2	0.984755194	1.510502407	7.436592915	1.27E-11	5.90E-10	15.96747482
KCNC3	1.354883158	1.472291828	7.434346155	1.29E-11	5.96E-10	15.95582254
SERTAD3	0.69844479	3.757423982	7.43148304	1.31E-11	6.03E-10	15.94097569
PDK4	1.91356514	2.195121961	7.430086229	1.31E-11	6.05E-10	15.93373327
HLA-DMB	1.818100182	4.793624578	7.429920934	1.32E-11	6.05E-10	15.93287625
CASTOR2	-0.933310966	3.887576021	-7.427022131	1.34E-11	6.13E-10	15.91784788
RPGRIP1L	-0.607277264	1.628124875	-7.415849773	1.42E-11	6.49E-10	15.8599483
TGFBI	2.089937168	2.420146301	7.415567605	1.42E-11	6.49E-10	15.85848644
CEBPB	1.461295407	5.441123409	7.414866763	1.43E-11	6.50E-10	15.8548556
ANKRD27	-0.886407202	5.406489645	-7.413183374	1.44E-11	6.54E-10	15.84613506

ZNF566	-0.722664771	3.155348321	-7.411826762	1.45E-11	6.57E-10	15.83910791
TMEM71	0.821843964	5.680512432	7.410310456	1.46E-11	6.61E-10	15.83125416
TADA2B	0.61762122	3.488385362	7.406283084	1.49E-11	6.73E-10	15.81039735
EPHB2	0.874608558	0.60080451	7.405133247	1.50E-11	6.76E-10	15.80444344
ARAP1	0.972777873	6.048627619	7.397847647	1.56E-11	7.01E-10	15.76672683
CDC42EP1	1.592870571	1.759294306	7.387398395	1.65E-11	7.36E-10	15.71265819
VMP1	0.835947487	6.108875047	7.387244314	1.65E-11	7.36E-10	15.71186114
OAZ1	0.601437471	7.988516991	7.381258423	1.70E-11	7.58E-10	15.68090173
TPP1	0.680224453	8.568303369	7.380405903	1.71E-11	7.60E-10	15.67649326
B4GALT1	0.674007701	5.868795708	7.362196353	1.88E-11	8.33E-10	15.58237846
PRKCD	1.207322251	6.83528728	7.362060002	1.88E-11	8.33E-10	15.58167409
JAK2	0.886203668	5.527801712	7.360815201	1.90E-11	8.37E-10	15.57524388
ZDHHC9	-0.758845159	4.104839287	-7.354986716	1.96E-11	8.61E-10	15.54514176
TREM1	1.891501459	4.039106566	7.353544841	1.97E-11	8.66E-10	15.53769645
TASP1	-0.62260108	3.809497116	-7.351531127	1.99E-11	8.73E-10	15.52729937
SNN	0.901958324	4.624059473	7.350214923	2.01E-11	8.77E-10	15.52050424
GAA	1.308728186	5.339856175	7.343960877	2.07E-11	9.02E-10	15.4882234
EVI5	0.940473689	3.167106133	7.337575469	2.14E-11	9.29E-10	15.45527596
TLR6	1.33307555	3.723108823	7.336735835	2.15E-11	9.31E-10	15.45094448
DNAJC5	0.681896893	5.821633326	7.335266982	2.17E-11	9.36E-10	15.44336749
GRK4	-0.665302898	2.724808258	-7.326499887	2.27E-11	9.78E-10	15.39815571
AHNAK	1.259906471	7.843533598	7.324465727	2.30E-11	9.87E-10	15.38766871
ADAM22	-0.960279578	1.600604217	-7.321844139	2.33E-11	9.98E-10	15.37415501
RGS19	0.860012386	6.585377363	7.317379335	2.38E-11	1.02E-09	15.35114447
SERPINB8	1.507070394	4.943419245	7.311425507	2.46E-11	1.05E-09	15.32046875
ZCWPW1	0.790534178	3.577620512	7.30374777	2.56E-11	1.09E-09	15.28092603
ITGB7	1.387258837	4.276810181	7.300618942	2.60E-11	1.10E-09	15.26481647
CLBA1	-0.704484298	2.276878736	-7.300565048	2.60E-11	1.10E-09	15.264539
PPT1	0.765370348	7.358764495	7.300206473	2.61E-11	1.10E-09	15.26269297
METTL7B	2.079086601	3.135184607	7.294559286	2.69E-11	1.13E-09	15.23362482
ZNF138	-0.757634897	4.311833695	-7.281454506	2.88E-11	1.21E-09	15.16620525
ZFP36	1.268475787	8.546543736	7.272364783	3.02E-11	1.27E-09	15.11947105
VPS37C	0.706720006	3.836060615	7.269436533	3.07E-11	1.28E-09	15.10442077
FAM49B	0.602741815	5.563100923	7.264922317	3.14E-11	1.31E-09	15.08122403
PRNP	0.976554284	5.928159528	7.25488672	3.31E-11	1.38E-09	15.02967648
SNX11	0.601092485	4.569563705	7.248832034	3.42E-11	1.42E-09	14.99859105
SLC26A11	1.079652613	1.902615127	7.247218752	3.45E-11	1.43E-09	14.9903101
CATSPER1	0.963644407	1.665102469	7.246731675	3.46E-11	1.43E-09	14.98781009
LGALS1	1.897667089	7.670338398	7.24543661	3.48E-11	1.44E-09	14.98116328
RIN1	1.216737217	2.352769366	7.24506789	3.49E-11	1.44E-09	14.97927094
ZNF366	1.00437586	1.062885044	7.242420375	3.54E-11	1.45E-09	14.96568462
PFKFB4	1.163240307	3.267448177	7.24180798	3.55E-11	1.46E-09	14.96254227
SERPINA1	2.558622214	5.451767005	7.233179021	3.71E-11	1.52E-09	14.91827672
CD53	0.800601716	8.671482078	7.23296623	3.71E-11	1.52E-09	14.91718541
C3orf33	-0.625509207	2.588402458	-7.230997242	3.75E-11	1.53E-09	14.90708793
ZNF165	-0.640862697	1.757063446	-7.22898247	3.79E-11	1.54E-09	14.89675685
SIRPB2	1.598490309	5.355715365	7.220879695	3.96E-11	1.60E-09	14.85522067

IRAK1BP1	-0.75123692	2.713129458	-7.211772786	4.15E-11	1.68E-09	14.80856039
GYG1	0.698147093	5.268926279	7.19763316	4.47E-11	1.80E-09	14.7361634
LCP1	0.949940673	9.478132629	7.19660901	4.49E-11	1.81E-09	14.73092192
SIGLEC14	1.590328383	4.806286001	7.195068802	4.53E-11	1.82E-09	14.72303991
CEBPD	1.698692356	5.724702761	7.192317993	4.60E-11	1.84E-09	14.70896441
ZNF431	-0.679857183	4.22101982	-7.18996187	4.65E-11	1.86E-09	14.69691027
C16orf96	-0.770336917	1.434893467	-7.182514557	4.84E-11	1.93E-09	14.65882011
CREB3L4	-0.711456963	4.482782426	-7.177652397	4.96E-11	1.97E-09	14.63396101
SLC1A3	1.382766734	2.854774884	7.176739143	4.99E-11	1.98E-09	14.62929255
RAB11FIP1	1.000676359	5.584896219	7.176068107	5.00E-11	1.98E-09	14.62586245
RCBTB2	1.068172938	4.419327273	7.171396226	5.13E-11	2.03E-09	14.60198516
TEDC1	0.922514496	2.788610537	7.17048752	5.15E-11	2.03E-09	14.59734166
ANKRD33B	-1.266281076	3.003574041	-7.161754925	5.39E-11	2.12E-09	14.55273075
SMCO4	1.301471992	4.116323225	7.113312585	6.94E-11	2.72E-09	14.30568232
GLT1D1	1.630661148	1.684444456	7.110983426	7.03E-11	2.75E-09	14.29382211
NUAK2	1.14676389	2.625451277	7.103853768	7.29E-11	2.85E-09	14.25752788
FAM227A	-0.689080677	0.994665353	-7.100630321	7.41E-11	2.89E-09	14.24112376
SIGLEC10	1.304255249	4.240194862	7.100246402	7.43E-11	2.89E-09	14.2391702
UPP1	1.491507104	3.457215536	7.099497834	7.46E-11	2.90E-09	14.2353613
OCEL1	0.675889038	3.63978286	7.09905068	7.48E-11	2.90E-09	14.23308614
MICAL2	1.255586784	3.008725041	7.096255921	7.59E-11	2.93E-09	14.21886758
PLB1	1.069832562	3.438253841	7.093611826	7.69E-11	2.97E-09	14.20541778
HIP1R	-1.06140055	3.042560501	-7.089942933	7.84E-11	3.02E-09	14.18675869
ST14	1.800026358	2.721226034	7.080444298	8.23E-11	3.16E-09	14.13847042
CBX2	-1.605884087	3.960311897	-7.069928443	8.70E-11	3.33E-09	14.08504372
HDAC9	1.373995256	2.755337467	7.068809864	8.75E-11	3.34E-09	14.07936272
KCNMB1	1.226516324	2.092498651	7.064093002	8.97E-11	3.42E-09	14.05541119
RGS9BP	-0.840240433	0.854319345	-7.038331274	1.02E-10	3.90E-09	13.92472036
EGR2	1.760052553	2.422666244	7.037597053	1.03E-10	3.91E-09	13.92099868
CYTIP	1.127294538	5.522631923	7.035113614	1.04E-10	3.95E-09	13.90841169
SQOR	0.888921972	4.744048682	7.031000096	1.06E-10	4.03E-09	13.88756715
TMEM167A	0.741842784	5.774934442	7.030771461	1.07E-10	4.03E-09	13.88640874
VSIG2	1.521553037	2.999690491	7.003206454	1.23E-10	4.63E-09	13.7468687
ZNF439	-1.01584655	4.635037369	-7.001706116	1.24E-10	4.65E-09	13.73928062
PECAM1	1.396086782	6.525270106	6.992746855	1.30E-10	4.86E-09	13.6939835
CLEC12A	1.276521416	6.355024102	6.966129834	1.49E-10	5.56E-09	13.55956299
SGTB	0.786692931	3.181079758	6.95918162	1.54E-10	5.75E-09	13.52451105
AANAT	0.882374751	1.678927891	6.954434566	1.58E-10	5.88E-09	13.50057241
CRYBG1	0.999187684	5.507423869	6.950693576	1.61E-10	5.96E-09	13.48171236
GKAP1	-0.73519332	2.962791204	-6.943355406	1.67E-10	6.18E-09	13.44473053
CHP1	0.620297484	6.29288143	6.941761595	1.69E-10	6.22E-09	13.4367006
SNX21	0.62494766	2.486741328	6.915106721	1.94E-10	7.11E-09	13.30253162
MYL12A	0.617413402	7.208842864	6.911979373	1.97E-10	7.21E-09	13.28680523
SOX12	-0.930089704	5.050093335	-6.900789923	2.08E-10	7.63E-09	13.23056373
SNX30	0.770095147	3.909478506	6.894662567	2.15E-10	7.84E-09	13.19978339
HLX	1.177603616	3.726118575	6.891544065	2.19E-10	7.95E-09	13.1841226
MEX3B	-1.422496409	3.932182179	-6.878630181	2.34E-10	8.48E-09	13.1193049

PELI1	1.301609062	4.575684199	6.878118357	2.34E-10	8.49E-09	13.11673709
VSIG4	2.151471314	3.359857215	6.876988992	2.36E-10	8.52E-09	13.11107139
APOBEC3A	2.025986904	3.09209265	6.875700043	2.37E-10	8.56E-09	13.10460562
ITPK1	0.713586569	4.934491374	6.873242749	2.40E-10	8.65E-09	13.09228062
S1PR3	1.376616817	1.708505766	6.872211128	2.41E-10	8.68E-09	13.08710694
AEBP1	-1.513094604	3.86911051	-6.859533032	2.58E-10	9.23E-09	13.02355415
TK2	0.725920629	3.576658187	6.847383672	2.74E-10	9.81E-09	12.96270248
CD1C	1.990448489	2.358355123	6.8456561	2.77E-10	9.88E-09	12.95405375
NIBAN2	1.659718985	3.846634564	6.844431807	2.78E-10	9.92E-09	12.94792519
HPSE	1.449006737	2.930659831	6.844157929	2.79E-10	9.92E-09	12.94655428
HERPUD1	0.837144338	5.507596729	6.837858179	2.88E-10	1.02E-08	12.91502762
TDRD7	0.916275593	4.279783036	6.835556363	2.91E-10	1.03E-08	12.90351169
ZCCHC18	-0.792556837	1.172526374	-6.824024767	3.09E-10	1.09E-08	12.84584643
CASP6	-0.719648908	5.213041028	-6.820550632	3.15E-10	1.11E-08	12.82848242
ARHGAP27	0.854159157	5.095147166	6.816045544	3.22E-10	1.13E-08	12.80597174
ZNF749	-0.759057001	3.218541898	-6.81580669	3.22E-10	1.13E-08	12.80477844
CD1E	1.452860223	1.004033052	6.815170459	3.23E-10	1.13E-08	12.80159998
RRAS	1.240502862	4.442522319	6.812604863	3.28E-10	1.15E-08	12.78878426
SLC24A4	1.536612642	2.665330398	6.811560131	3.29E-10	1.15E-08	12.78356624
FTH1	0.959376155	7.943376372	6.80701812	3.37E-10	1.17E-08	12.76088502
SLA	1.198324974	5.03414878	6.800149188	3.49E-10	1.21E-08	12.72659738
ZNF70	-0.844179388	4.464593005	-6.798005819	3.53E-10	1.22E-08	12.71590163
NUDT16	0.94448046	4.155256848	6.792899252	3.62E-10	1.25E-08	12.69042541
TBC1D32	-0.673723245	2.728638095	-6.792191285	3.64E-10	1.25E-08	12.68689414
ZNF493	-0.825889284	3.681322645	-6.792066377	3.64E-10	1.25E-08	12.68627112
SH3BGRL3	0.944094318	8.963439244	6.787234223	3.73E-10	1.28E-08	12.66217356
EGFL8	-0.641106926	2.447257518	-6.786661191	3.74E-10	1.28E-08	12.65931643
RIPOR2	1.060739258	6.114790125	6.781699346	3.84E-10	1.31E-08	12.63458145
RAB29	0.909208814	4.251233131	6.781327121	3.84E-10	1.31E-08	12.63272624
ADAM9	1.196253707	3.621388102	6.775938165	3.95E-10	1.35E-08	12.60587239
VASP	0.733312051	6.474371156	6.772202385	4.03E-10	1.37E-08	12.58726242
SH3TC1	1.352681359	3.768880312	6.771400188	4.04E-10	1.38E-08	12.58326687
PGAM1	0.70581659	5.341474244	6.768824856	4.10E-10	1.39E-08	12.57044125
RNF13	0.638920545	5.284437	6.767324124	4.13E-10	1.40E-08	12.5629684
IL10	1.23932047	1.073275893	6.759882119	4.29E-10	1.45E-08	12.52592265
IMPDH1	0.793595113	6.141065089	6.756050116	4.37E-10	1.48E-08	12.50685472
TARBP1	-0.813034353	6.217062419	-6.751177949	4.48E-10	1.51E-08	12.48261834
ZNF134	-0.62138572	4.132876927	-6.750256391	4.50E-10	1.51E-08	12.47803502
CCDC171	-0.826330499	2.031705122	-6.74843364	4.55E-10	1.52E-08	12.46897053
NT5M	-0.949029336	4.115793437	-6.745773108	4.61E-10	1.54E-08	12.45574187
TCIRG1	1.210280382	6.746062304	6.743270751	4.67E-10	1.56E-08	12.44330193
TNFRSF11A	1.020609541	1.094071037	6.738589719	4.78E-10	1.59E-08	12.42003704
TARSL2	-0.640049885	3.709314692	-6.738414915	4.78E-10	1.59E-08	12.41916841
CLCN5	1.080682522	2.431983634	6.737764695	4.80E-10	1.59E-08	12.41593744
TNFAIP8L2	0.855007713	5.842560513	6.734888016	4.87E-10	1.61E-08	12.40164487
ZNF280B	-0.636876223	2.073650487	-6.731588258	4.95E-10	1.64E-08	12.38525384
RTL10	-0.598611293	4.268597529	-6.722184124	5.20E-10	1.71E-08	12.33856121

ANG	0.780510026	1.097202738	6.719956924	5.26E-10	1.73E-08	12.32750745
IL27	0.688727693	1.139291197	6.715690763	5.37E-10	1.77E-08	12.30633905
SAMD4A	0.786592506	0.79867859	6.706437211	5.63E-10	1.85E-08	12.26044558
LYN	0.875550782	6.598695186	6.694937617	5.97E-10	1.95E-08	12.20345492
GNAI2	0.650911601	7.849138032	6.694762238	5.97E-10	1.95E-08	12.20258613
NOTCH2	1.083386756	5.69612951	6.687313391	6.20E-10	2.02E-08	12.16569597
CSTA	2.038243828	5.372707026	6.685144595	6.27E-10	2.04E-08	12.15495879
MSI2	-1.326151042	4.894079552	-6.685053552	6.27E-10	2.04E-08	12.15450809
C1QTNF3	-0.641365635	3.164871215	-6.684494563	6.29E-10	2.04E-08	12.15174097
TRIB1	1.35380979	5.715973837	6.682628769	6.35E-10	2.06E-08	12.14250564
PPARD	1.005736169	3.356654163	6.680689217	6.41E-10	2.07E-08	12.13290654
FAM151B	0.995572315	2.210438702	6.677744861	6.51E-10	2.10E-08	12.1183371
VN1R1	-0.809163888	2.742939661	-6.668041188	6.84E-10	2.20E-08	12.07034267
ZNF114	-0.796300194	1.615716629	-6.665650599	6.92E-10	2.22E-08	12.05852397
SH3BP2	0.931056108	5.368334244	6.662382863	7.04E-10	2.26E-08	12.0423721
NPL	1.471333293	3.043714223	6.661705342	7.06E-10	2.26E-08	12.03902371
EBPL	-0.702473989	5.968493281	-6.661137384	7.08E-10	2.26E-08	12.03621691
CCDC138	-0.646327177	2.993252468	-6.651474144	7.44E-10	2.37E-08	11.98847985
CPVL	1.957018341	4.473917581	6.65011575	7.49E-10	2.39E-08	11.98177198
AKAP13	0.704508949	5.7125159	6.648981765	7.53E-10	2.40E-08	11.97617277
LIPA	0.875886715	5.767390783	6.647162115	7.60E-10	2.41E-08	11.96718896
PLXNB2	1.077577865	6.963815539	6.646300968	7.64E-10	2.42E-08	11.9629378
KDM7A	1.129505633	3.878429953	6.645734475	7.66E-10	2.42E-08	11.96014138
FXYD6	1.42799273	1.768818323	6.643932992	7.73E-10	2.44E-08	11.95124936
ATP6V1B2	0.766320356	6.083095861	6.642516296	7.78E-10	2.45E-08	11.94425745
TLR2	1.420503934	6.243757747	6.641846446	7.81E-10	2.46E-08	11.94095175
ENHO	0.967804295	0.641646404	6.639427801	7.91E-10	2.49E-08	11.92901711
SHKBP1	0.753135224	6.58080839	6.629355168	8.32E-10	2.61E-08	11.87933712
TMCC3	0.889761142	1.22545433	6.627324446	8.40E-10	2.63E-08	11.86932569
QPCT	1.654711164	2.227826729	6.625875505	8.47E-10	2.65E-08	11.86218334
RNF166	0.793200038	5.704947975	6.621577879	8.65E-10	2.70E-08	11.84100329
CASP5	1.06349305	0.987997287	6.617524966	8.83E-10	2.75E-08	11.82103539
ZNF222	-0.603903911	2.503171466	-6.614357579	8.97E-10	2.78E-08	11.80543446
APOBR	1.1485393	5.659912954	6.610859394	9.13E-10	2.83E-08	11.78820843
SLC16A6	1.02970139	1.403485118	6.602149718	9.54E-10	2.95E-08	11.74533895
MILR1	1.128528442	5.375234278	6.601939983	9.55E-10	2.95E-08	11.74430696
DESI1	0.658803189	4.496755104	6.598442003	9.72E-10	2.99E-08	11.72709778
CD300LF	1.208724606	5.758063142	6.597013493	9.79E-10	3.01E-08	11.72007116
OGFR	0.675681602	5.214758512	6.592866304	1.00E-09	3.06E-08	11.69967599
TNFSF13B	1.039395297	6.455485449	6.58435156	1.04E-09	3.19E-08	11.65782169
ZNF84	-0.751828594	4.144247408	-6.579228183	1.07E-09	3.27E-08	11.6326505
CDADC1	-0.646798022	4.085404162	-6.575048298	1.09E-09	3.33E-08	11.61212185
POR	0.767649482	3.976913168	6.570106085	1.12E-09	3.41E-08	11.58785747
HLA-DRB1	1.840421453	8.392535262	6.56902553	1.13E-09	3.42E-08	11.58255356
ZSCAN31	-0.826042855	2.681562919	-6.564435181	1.15E-09	3.50E-08	11.5600266
PLA2G12A	-0.655746836	3.868826904	-6.556695248	1.20E-09	3.62E-08	11.52206082
ZNF382	-0.83756045	2.408869137	-6.556430766	1.20E-09	3.62E-08	11.52076388

SCGB1A1	0.811440529	0.779970444	6.546704488	1.26E-09	3.79E-08	11.47308713
TNFSF12	1.371107951	3.125894119	6.54007773	1.30E-09	3.92E-08	11.44062388
RP2	0.695528844	5.46664387	6.53975545	1.31E-09	3.92E-08	11.43904551
MAST3	0.809770644	4.944478148	6.539092534	1.31E-09	3.92E-08	11.43579899
STAC3	0.898308772	4.699850086	6.53393416	1.35E-09	4.02E-08	11.41054228
FFAR4	0.647965919	0.537889487	6.529483939	1.38E-09	4.11E-08	11.38876085
MRVI1	0.878542672	1.621305751	6.52614246	1.40E-09	4.17E-08	11.37241097
TRIM38	0.613569932	5.755880372	6.512175919	1.50E-09	4.46E-08	11.30411783
TIFAB	1.624715891	1.906984931	6.509898889	1.52E-09	4.50E-08	11.29299062
NUCB2	-0.911048583	6.633682321	-6.497129422	1.62E-09	4.78E-08	11.23062592
FPR2	1.722116895	2.345868024	6.495348492	1.63E-09	4.81E-08	11.22193293
JAZF1	1.104569105	3.56795389	6.495267208	1.63E-09	4.81E-08	11.2215362
SMIM17	-0.631364547	1.230758383	-6.491789388	1.66E-09	4.88E-08	11.20456399
KLHL23	-1.05787876	3.347207636	-6.488908951	1.69E-09	4.95E-08	11.19051055
PGBD1	-0.811076352	3.118669066	-6.478991223	1.77E-09	5.18E-08	11.1421467
TP53BP1	-0.68370059	4.420865244	-6.478072548	1.78E-09	5.20E-08	11.13766866
MOB3B	1.031557311	2.714334801	6.477506389	1.78E-09	5.21E-08	11.1349091
DPYSL2	1.624673101	4.533313298	6.476758408	1.79E-09	5.22E-08	11.13126351
MATK	-0.941572675	4.282970203	-6.474050032	1.82E-09	5.28E-08	11.11806487
PLEC	1.274180311	5.86894994	6.472711808	1.83E-09	5.30E-08	11.11154438
OASL	1.098437955	1.806095849	6.472614573	1.83E-09	5.30E-08	11.11107063
ZNF610	-0.91644196	2.332072247	-6.470995197	1.84E-09	5.34E-08	11.1031812
TTC8	-0.824922472	2.402473251	-6.463185221	1.92E-09	5.53E-08	11.06514581
TM7SF3	-0.900287447	7.259520097	-6.459563204	1.95E-09	5.62E-08	11.0475141
IQSEC1	1.077644726	5.009006179	6.458024079	1.97E-09	5.65E-08	11.04002327
PMM1	0.794567508	2.032967349	6.451554646	2.03E-09	5.82E-08	11.00854681
SYT17	0.86835364	0.786717116	6.446131891	2.09E-09	5.97E-08	10.98217521
TEC	-0.875211091	5.699855455	-6.443768692	2.11E-09	6.03E-08	10.97068617
NHS	0.627099115	0.676771251	6.440253803	2.15E-09	6.13E-08	10.95360199
CAST	1.050792934	4.950903746	6.43979908	2.15E-09	6.14E-08	10.95139214
MRC2	-1.754902314	3.620030146	-6.437555993	2.18E-09	6.20E-08	10.94049245
IL6R	0.873045362	6.1449863	6.436000007	2.20E-09	6.23E-08	10.93293267
ATP2C1	-0.593204093	5.711109494	-6.43580775	2.20E-09	6.23E-08	10.93199865
RXRA	1.229171336	4.117097117	6.435656241	2.20E-09	6.23E-08	10.93126261
ITPRIP	0.796175864	4.467103504	6.435058301	2.21E-09	6.23E-08	10.92835783
C5AR2	1.100314671	1.765420941	6.434979301	2.21E-09	6.23E-08	10.92797407
AIF1	0.961401642	7.835952418	6.429698365	2.27E-09	6.39E-08	10.9023256
SLC49A3	1.085991225	2.871521809	6.428455883	2.28E-09	6.42E-08	10.89629267
RASSF5	0.695711571	5.323003895	6.427084449	2.29E-09	6.45E-08	10.8896343
ACOT9	0.761209557	4.231825351	6.412373655	2.47E-09	6.90E-08	10.81825838
PLPPR2	0.937138845	4.71332495	6.410153874	2.50E-09	6.97E-08	10.8074954
ZNF85	-0.785399762	3.443608212	-6.406767413	2.54E-09	7.08E-08	10.79107925
CAMSAP2	0.682520989	1.048112294	6.403019089	2.59E-09	7.20E-08	10.77291413
RARA	0.87664126	5.016438459	6.401838856	2.60E-09	7.23E-08	10.76719561
TBC1D8	1.027156501	3.871018173	6.397966046	2.65E-09	7.35E-08	10.7484347
ZNF559-ZNI	-0.609971393	1.462369953	-6.39523965	2.69E-09	7.44E-08	10.73523082
OR2B11	0.744074381	1.080094146	6.390946897	2.75E-09	7.58E-08	10.71444697

CES1	2.244667697	1.904643207	6.387020217	2.80E-09	7.71E-08	10.69544179
HPS4	-0.721351284	5.051665412	-6.382639428	2.86E-09	7.87E-08	10.67424581
FOLR2	1.226495839	1.528494562	6.37884697	2.92E-09	8.01E-08	10.65590247
CRIP1	1.136610443	2.768382449	6.374318096	2.98E-09	8.18E-08	10.6340046
ZNF486	-0.999009935	3.126163436	-6.362071533	3.17E-09	8.66E-08	10.57483065
BEST1	0.850191289	2.276921244	6.358309081	3.23E-09	8.80E-08	10.55666275
CTSD	1.194675807	8.014973105	6.357292214	3.25E-09	8.83E-08	10.55175352
OSTM1	0.653658101	4.207079086	6.35674181	3.25E-09	8.85E-08	10.54909645
MCMBP	0.610459102	5.455970868	6.348858003	3.38E-09	9.17E-08	10.51105054
ZCCHC24	1.015822329	2.596728912	6.340211177	3.53E-09	9.56E-08	10.46935062
ZNRF1	-0.809544358	4.537789952	-6.338245631	3.57E-09	9.64E-08	10.45987577
SAT1	1.222018083	7.790548298	6.337910597	3.57E-09	9.64E-08	10.45826089
ZNF738	-0.630377011	3.62665185	-6.33725202	3.58E-09	9.66E-08	10.45508667
AC093323.1	-0.598879815	4.187770586	-6.325177111	3.81E-09	1.02E-07	10.39691827
PRSS57	-1.72671553	8.24163735	-6.32394466	3.83E-09	1.02E-07	10.39098444
QSOX2	-0.693243589	4.960639311	-6.321202328	3.88E-09	1.04E-07	10.37778323
SIDT2	0.839346876	4.685085183	6.319236663	3.92E-09	1.04E-07	10.36832263
IGF2R	1.486142463	3.903582084	6.318883574	3.93E-09	1.04E-07	10.36662339
SRD5A1	0.7619128	2.3576173	6.318559418	3.93E-09	1.05E-07	10.36506345
ZBTB12	-0.598059653	3.540669385	-6.317986076	3.94E-09	1.05E-07	10.36230444
C1orf127	0.903250872	1.293468037	6.317641599	3.95E-09	1.05E-07	10.36064683
KCNH3	0.842936107	1.615784539	6.314804395	4.01E-09	1.06E-07	10.34699608
RASGRP4	1.248557341	5.714455948	6.31284709	4.04E-09	1.07E-07	10.33758069
SLC35F6	0.744345123	4.484837117	6.310582652	4.09E-09	1.08E-07	10.32668979
TMEM150B	1.511212877	3.217428568	6.303880255	4.23E-09	1.11E-07	10.29446634
COQ10A	-0.592101824	3.422443121	-6.300951077	4.29E-09	1.13E-07	10.28038923
FOSL2	0.983470092	6.887586668	6.29593969	4.40E-09	1.16E-07	10.25631335
WRN	-0.660728686	4.645307106	-6.295389839	4.41E-09	1.16E-07	10.25367234
WNT5B	0.641386669	0.867034989	6.295102883	4.41E-09	1.16E-07	10.25229411
FTL	0.799611033	11.45654338	6.292038921	4.48E-09	1.17E-07	10.2375801
PLD3	1.008043205	6.092690116	6.290648638	4.51E-09	1.18E-07	10.23090481
BCR	-0.85665789	5.344748003	-6.289297217	4.54E-09	1.19E-07	10.22441686
MALT1	0.653492109	3.635856619	6.288854909	4.55E-09	1.19E-07	10.22229357
STAT6	0.59719232	7.244053141	6.28252146	4.70E-09	1.22E-07	10.19189861
CR1	1.681204477	2.757106434	6.280589218	4.74E-09	1.23E-07	10.18262877
ZNF106	0.698395579	5.4041042	6.279805039	4.76E-09	1.24E-07	10.17886713
SMPDL3A	1.422273106	1.446687814	6.277647544	4.81E-09	1.25E-07	10.16851911
FUCA1	0.887005431	4.563612851	6.274102605	4.90E-09	1.27E-07	10.15152053
CDC42BPG	-0.775879606	2.090279903	-6.270018772	5.00E-09	1.29E-07	10.13194416
HFE	0.735857693	0.972314187	6.258554638	5.28E-09	1.36E-07	10.0770254
ZNF74	-0.670704703	3.76921638	-6.253445384	5.42E-09	1.39E-07	10.05256674
PPP1R9B	0.595355993	6.907087375	6.251479494	5.47E-09	1.40E-07	10.04315859
AGFG1	0.610233306	5.424966404	6.249811251	5.52E-09	1.41E-07	10.03517612
CKAP4	1.560412495	3.842491992	6.239019199	5.82E-09	1.49E-07	9.983563973
HLA-DRA	1.795166474	10.15012988	6.220355568	6.38E-09	1.63E-07	9.894418719
BLOC1S1	0.643687808	4.883621373	6.215594492	6.53E-09	1.66E-07	9.871700664
GLIPR1	0.954593777	6.364975625	6.21463344	6.56E-09	1.67E-07	9.867116012

SELPLG	1.008937055	7.445011457	6.207116843	6.80E-09	1.73E-07	9.831271564
GAS7	1.198162234	5.46310221	6.206839988	6.81E-09	1.73E-07	9.829951765
CALHM2	0.991744257	4.511265773	6.200527692	7.03E-09	1.78E-07	9.799868942
ARHGAP31	1.125971758	2.588767677	6.200055674	7.04E-09	1.78E-07	9.797620086
DYTN	-1.324971163	2.507354925	-6.197071646	7.15E-09	1.81E-07	9.783405248
GSR	0.604835235	5.830020636	6.196604439	7.16E-09	1.81E-07	9.781179979
PPFIBP2	0.928983059	1.726621015	6.196085902	7.18E-09	1.81E-07	9.778710328
PRDM11	-0.682049325	2.987104248	-6.191059143	7.36E-09	1.85E-07	9.754775016
SLC39A3	-0.781624606	4.899989664	-6.190683563	7.37E-09	1.85E-07	9.75298708
SLCO3A1	0.699496671	4.080369001	6.186615198	7.52E-09	1.89E-07	9.733623514
ABCD1	0.811162806	4.409227841	6.180977563	7.73E-09	1.94E-07	9.706802261
GNB2	0.668157297	6.805626854	6.177408319	7.87E-09	1.97E-07	9.689828264
IFT22	-0.607096029	2.105950461	-6.175979295	7.92E-09	1.98E-07	9.683033834
TNFSF8	1.18445916	3.753683616	6.170729661	8.13E-09	2.03E-07	9.658081247
TRAF3IP2	-0.612538096	4.153976412	-6.170286344	8.15E-09	2.03E-07	9.655974594
WDR31	-0.685190513	2.405144535	-6.167149792	8.27E-09	2.06E-07	9.641071965
SHF	-0.859711531	2.354750277	-6.16004936	8.56E-09	2.13E-07	9.607350971
ZNF107	-0.95423243	4.725086367	-6.157783003	8.66E-09	2.15E-07	9.596592133
NEDD9	0.858551791	3.128009152	6.157100317	8.69E-09	2.15E-07	9.593351709
DOK4	-1.008985436	2.100129598	-6.155202497	8.77E-09	2.17E-07	9.584344577
TEX30	-0.763436581	4.471910262	-6.152595923	8.88E-09	2.20E-07	9.571976125
PADI2	1.575029969	2.976435652	6.151505851	8.93E-09	2.20E-07	9.566804463
ANO6	0.724510087	5.154085785	6.14928016	9.02E-09	2.23E-07	9.556246598
TMEM220	-0.662101488	4.264433752	-6.147345192	9.11E-09	2.24E-07	9.5470695
PPM1H	-1.093115927	3.284255232	-6.145472799	9.19E-09	2.26E-07	9.538190671
CD302	0.88527293	5.153021578	6.139979419	9.44E-09	2.31E-07	9.512149714
RNF175	1.116922291	2.777523816	6.138791896	9.50E-09	2.32E-07	9.506522012
RNPEP	0.631154659	5.97306119	6.133702437	9.74E-09	2.38E-07	9.482409644
EPHB3	1.176696662	1.559184593	6.130032392	9.91E-09	2.41E-07	9.4650288
DNMT3B	-1.281158355	4.505694077	-6.125869717	1.01E-08	2.46E-07	9.445321781
CAPZA2	0.599394829	5.158923997	6.119816359	1.04E-08	2.53E-07	9.416676876
ARHGAP45	0.860809157	6.827619553	6.119010187	1.05E-08	2.54E-07	9.412863182
NOL4L	-0.815023669	4.843784781	-6.117202871	1.05E-08	2.56E-07	9.404314447
SDCBP	0.701701104	6.746171862	6.116687072	1.06E-08	2.56E-07	9.401874934
TMEM67	-0.794780716	2.174968661	-6.113317831	1.08E-08	2.60E-07	9.385942601
MX2	1.039558226	3.61685978	6.111714406	1.08E-08	2.62E-07	9.378362077
CORO1C	0.677966449	5.828314827	6.110391027	1.09E-08	2.63E-07	9.372106342
PNPLA1	0.961053432	1.251526513	6.109076515	1.10E-08	2.64E-07	9.365893256
PDZD7	0.941294206	1.496407969	6.105302941	1.12E-08	2.69E-07	9.348061402
ADAT2	-0.625064873	4.158160382	-6.103612751	1.13E-08	2.70E-07	9.340076436
CMKLR1	1.20786654	1.841052602	6.102742757	1.13E-08	2.71E-07	9.335966797
P3H4	-0.768558986	2.044164965	-6.097834547	1.16E-08	2.77E-07	9.312787621
PDE4A	1.236458356	2.198651345	6.094756341	1.18E-08	2.81E-07	9.298255912
PID1	1.098849312	1.097646982	6.09264761	1.19E-08	2.84E-07	9.288303265
YPEL2	0.65360559	4.270941153	6.089293963	1.21E-08	2.88E-07	9.272478835
SLC9A9	1.214891658	2.325691058	6.087952889	1.22E-08	2.90E-07	9.266152217
APLP2	0.883194679	8.197501409	6.087373042	1.22E-08	2.90E-07	9.263416981

PHOSPHO2	-0.704669927	3.339245817	-6.086803928	1.22E-08	2.91E-07	9.260732516
TPK1	0.70004007	2.17795796	6.082731477	1.25E-08	2.96E-07	9.241527112
ARHGAP23	-0.833652555	3.912503516	-6.063746104	1.37E-08	3.24E-07	9.152086817
METRNL	1.214026373	3.931491566	6.063508211	1.37E-08	3.24E-07	9.150967078
TMEM144	0.901967835	2.130255596	6.061093096	1.38E-08	3.27E-07	9.139600755
ZNF660	-0.793752903	3.738619893	-6.057568325	1.41E-08	3.33E-07	9.123016512
RBP7	1.108613896	1.493807902	6.054624285	1.43E-08	3.37E-07	9.10916873
YPEL1	-0.742744675	3.127185552	-6.052600082	1.44E-08	3.40E-07	9.099649714
MYB	-0.707706002	7.576041513	-6.05019808	1.46E-08	3.43E-07	9.08835635
CD1B	0.779060387	0.41282489	6.044433744	1.50E-08	3.52E-07	9.061264603
ZC3HAV1L	-0.865670279	4.575340843	-6.043216632	1.51E-08	3.54E-07	9.055546144
NINJ1	1.211601133	4.521351628	6.042446906	1.52E-08	3.55E-07	9.05193
CYTH4	1.348994417	5.469444478	6.042144549	1.52E-08	3.55E-07	9.050509612
ZNF880	-0.690763364	3.145857649	-6.030283437	1.61E-08	3.75E-07	8.994820488
KIF1C	0.88776036	3.084379362	6.021496369	1.68E-08	3.90E-07	8.95360356
SNX10	1.197312915	4.968440172	6.01922085	1.70E-08	3.94E-07	8.942935386
TKT	0.730256581	6.988423029	6.011785331	1.76E-08	4.08E-07	8.908091584
ZNF221	-0.825387388	2.857087093	-6.011585741	1.76E-08	4.08E-07	8.907156613
CRYBG3	1.201998033	2.563058645	6.009916368	1.77E-08	4.11E-07	8.899337162
SLC30A1	0.723989788	4.008707634	5.9952616	1.90E-08	4.39E-07	8.83074542
IPCEF1	1.024067377	2.515343029	5.994563551	1.91E-08	4.40E-07	8.827480534
TMEM44	-0.721809681	3.976651619	-5.993947717	1.91E-08	4.41E-07	8.82460036
DENND1A	0.589325616	4.728069909	5.991879458	1.93E-08	4.45E-07	8.814928592
CEL	-0.831882684	2.169194817	-5.991656416	1.94E-08	4.45E-07	8.813885697
SLC16A3	0.995249576	4.271781795	5.98694541	1.98E-08	4.53E-07	8.791863147
RAB3D	0.840684558	5.737338163	5.985230548	2.00E-08	4.56E-07	8.783849089
GLRX	0.60553836	4.452542989	5.983193429	2.02E-08	4.60E-07	8.774330702
ZBTB10	-0.689546637	3.65811377	-5.979745888	2.05E-08	4.67E-07	8.758226302
EPB41L3	1.449903568	1.288164705	5.979103389	2.06E-08	4.68E-07	8.755225592
ALDH3B1	1.013421085	5.13310413	5.978606583	2.06E-08	4.68E-07	8.752905445
RMDN2	0.775645637	1.396508695	5.973164856	2.12E-08	4.79E-07	8.727499001
TPMT	0.586449667	4.405723877	5.969505825	2.15E-08	4.86E-07	8.71042296
SLC2A6	1.232718704	3.273328709	5.964528711	2.21E-08	4.97E-07	8.687205128
ORMDL3	-0.617265886	4.357926618	-5.961031517	2.24E-08	5.05E-07	8.67089753
TNNI2	1.392379589	2.286635468	5.959709312	2.26E-08	5.07E-07	8.664733422
NRGN	1.712381086	5.738642237	5.957542728	2.28E-08	5.12E-07	8.654634499
ZNF233	-0.622333207	2.639812913	-5.95363835	2.32E-08	5.21E-07	8.636440571
LRP5	-1.116483255	4.538757739	-5.951054623	2.35E-08	5.27E-07	8.62440442
ALS2	-1.001382484	4.445526631	-5.944313454	2.43E-08	5.44E-07	8.593014963
MAPK7	0.826189424	3.361176524	5.944053563	2.43E-08	5.44E-07	8.591805213
SLC49A4	0.729764778	3.72477268	5.943832811	2.44E-08	5.44E-07	8.590777673
ZNF496	-0.726964022	4.720642108	-5.942543458	2.45E-08	5.46E-07	8.584776522
SMIM24	-1.430542882	6.145034541	-5.913185529	2.82E-08	6.25E-07	8.448333245
SLC25A24	0.803130683	4.40504962	5.908121819	2.89E-08	6.38E-07	8.424838129
HLA-DQB1	1.87131234	5.311025635	5.908095365	2.89E-08	6.38E-07	8.424715412
SLC43A1	-0.672266119	5.34114339	-5.907389348	2.90E-08	6.40E-07	8.421440481
SUCLG2	1.269737905	3.960445568	5.899190003	3.02E-08	6.64E-07	8.383423361

ZNF467	1.237667983	4.617135123	5.899142736	3.02E-08	6.64E-07	8.383204292
GPSM1	-1.173299957	4.720733457	-5.898757823	3.02E-08	6.64E-07	8.381420348
APAF1	0.706129112	5.422319953	5.898440111	3.03E-08	6.65E-07	8.379947911
CDKN2D	0.897910857	4.977938426	5.892050318	3.12E-08	6.85E-07	8.350344029
CDKN2B	0.95901413	1.153040081	5.888292519	3.18E-08	6.96E-07	8.332942735
LRP12	-0.86919956	2.314729506	-5.885865405	3.21E-08	7.03E-07	8.321706842
ZFHX2	-0.592747092	1.602069911	-5.885456829	3.22E-08	7.04E-07	8.319815673
CSK	0.600169748	6.565500723	5.87461158	3.39E-08	7.40E-07	8.269643945
GRK3	0.778231938	4.961485295	5.871692258	3.44E-08	7.49E-07	8.256147799
AHR	1.494710103	5.62403484	5.870892749	3.45E-08	7.50E-07	8.252452306
MCOLN1	0.723518184	4.216811789	5.865058503	3.55E-08	7.71E-07	8.225494005
RELL2	0.972692259	2.331983565	5.864322172	3.56E-08	7.72E-07	8.222092736
ZNF574	-0.634645537	4.159374256	-5.860272917	3.63E-08	7.87E-07	8.203392754
RNASE1	1.416831718	2.32543554	5.858820286	3.65E-08	7.91E-07	8.19668613
CYP1B1	1.602295823	2.426508509	5.856732669	3.69E-08	7.98E-07	8.187049524
HOOK3	0.619856184	4.920527283	5.853574616	3.75E-08	8.09E-07	8.172475468
HSPA4L	-0.900037747	1.259430006	-5.85306937	3.76E-08	8.09E-07	8.170144231
ZNF433	-0.626387976	3.966435672	-5.85071209	3.80E-08	8.18E-07	8.159269145
FAHD2B	-0.697147036	2.413324083	-5.846991738	3.87E-08	8.30E-07	8.142110793
UBTD1	0.946839108	3.256853734	5.835632147	4.08E-08	8.73E-07	8.089759105
FAM229B	-0.698956642	1.561647513	-5.835322687	4.09E-08	8.73E-07	8.088333754
PYCARD	0.783858441	5.782293231	5.82927796	4.21E-08	8.97E-07	8.060500948
MYOZ3	-0.856330573	2.185421438	-5.828982815	4.21E-08	8.98E-07	8.059142387
MICAL1	1.002189315	6.061981847	5.825201099	4.29E-08	9.13E-07	8.041738569
TMEM120A	0.704351541	3.988183242	5.822061935	4.35E-08	9.26E-07	8.027296812
IL4R	0.861724892	4.892206994	5.81627819	4.47E-08	9.50E-07	8.000700456
RCN1	-0.60457515	4.504932108	-5.810846126	4.59E-08	9.73E-07	7.975735271
LTBR	0.595073354	5.092811826	5.804949101	4.72E-08	9.99E-07	7.94864853
ZNF253	-0.761925481	4.032726467	-5.797774467	4.88E-08	1.03E-06	7.915714951
TCEAL4	-0.697638348	4.858847369	-5.797317312	4.89E-08	1.03E-06	7.913617282
DNMT3A	-0.587725693	4.729137366	-5.79342564	4.98E-08	1.05E-06	7.895764169
S100A10	1.772534413	6.32798947	5.792424269	5.01E-08	1.05E-06	7.891171491
RUFY4	0.905040943	0.969611965	5.786683495	5.14E-08	1.08E-06	7.864851012
GPR137B	0.884002802	3.149216545	5.777116142	5.38E-08	1.12E-06	7.821020204
TAS2R60	1.108359015	2.097394499	5.776627725	5.39E-08	1.12E-06	7.818783762
LAP3	0.622306118	5.448073351	5.776615664	5.40E-08	1.12E-06	7.81872854
GPRIN3	1.122105938	2.606896478	5.774863506	5.44E-08	1.13E-06	7.810706399
RAB24	0.783765631	4.156407807	5.773915738	5.46E-08	1.13E-06	7.8063677
PKM	0.74191131	7.65479618	5.768519778	5.61E-08	1.16E-06	7.781673984
PTPN6	0.756453375	7.234023399	5.767670303	5.63E-08	1.16E-06	7.777787735
ZNF781	-0.679491095	1.31321807	-5.766425342	5.66E-08	1.17E-06	7.772092788
DBN1	-1.11562246	3.504567534	-5.764955244	5.70E-08	1.17E-06	7.765368906
BCL2A1	1.462566663	4.49759975	5.76485052	5.70E-08	1.17E-06	7.764889959
ZNF607	-0.680535918	2.817822889	-5.764028051	5.73E-08	1.18E-06	7.761128658
LPXN	0.77335463	5.992287907	5.759952472	5.84E-08	1.20E-06	7.742494938
SBF2	0.817410127	3.306455983	5.747809252	6.18E-08	1.27E-06	7.687021529
METAP1D	-0.637968802	3.688895941	-5.74749529	6.19E-08	1.27E-06	7.685588179

FCGR3A	1.792931264	4.109797777	5.746744313	6.21E-08	1.27E-06	7.68215989
ARHGEF11	1.485765085	3.607491639	5.746482985	6.22E-08	1.27E-06	7.680966965
MCTP1	0.772839175	3.917358502	5.732253885	6.65E-08	1.35E-06	7.616061265
SPIN4	-0.911592228	4.922614286	-5.73207834	6.65E-08	1.35E-06	7.615261113
WAS	0.641356408	7.211238062	5.731047334	6.69E-08	1.36E-06	7.610561975
CD74	1.449878671	10.44922754	5.729932622	6.72E-08	1.36E-06	7.605481884
ZNF248	-0.604255633	3.542671017	-5.728561196	6.77E-08	1.37E-06	7.599232667
PTTG2	0.686011963	1.654831213	5.726768641	6.82E-08	1.38E-06	7.591065812
EXT1	0.799578964	1.35437553	5.725878616	6.85E-08	1.38E-06	7.587011431
VNN1	2.177578536	3.874046957	5.725664716	6.86E-08	1.38E-06	7.586037098
PLOD1	0.732917173	4.906085531	5.724812483	6.89E-08	1.39E-06	7.582155307
RNPEPL1	0.709757595	5.531318233	5.717060035	7.14E-08	1.44E-06	7.546859799
AIM2	1.032380245	2.083669993	5.709283748	7.41E-08	1.48E-06	7.511484211
PEX3	-0.591300383	4.301889541	-5.707952781	7.45E-08	1.49E-06	7.505432287
SLC26A6	0.787866742	3.885408723	5.704732921	7.57E-08	1.51E-06	7.490795005
ZNF506	-0.596625676	4.600922204	-5.704248806	7.58E-08	1.51E-06	7.488594673
GMIP	0.655122507	6.658274326	5.702659905	7.64E-08	1.52E-06	7.481373797
ANXA4	0.601401104	3.873726865	5.695440485	7.90E-08	1.57E-06	7.448579668
VOPP1	0.775176776	3.248072356	5.695302639	7.91E-08	1.57E-06	7.447953744
BMP1	-0.718630402	4.161042053	-5.695000262	7.92E-08	1.57E-06	7.44658076
FLOT1	0.952326874	5.537973537	5.694325754	7.94E-08	1.57E-06	7.443518214
CYP27A1	1.605940643	1.991508029	5.689142495	8.14E-08	1.61E-06	7.419991295
GRIK5	-1.367837048	4.429489501	-5.681887234	8.42E-08	1.66E-06	7.387080942
DNAAF3	-0.778898984	0.864056491	-5.675620409	8.67E-08	1.71E-06	7.358674352
ZEB2	0.681017306	5.77756038	5.672267537	8.81E-08	1.73E-06	7.343483957
MYH9	0.623846316	8.511742757	5.667888823	8.99E-08	1.76E-06	7.323653999
C20orf96	-0.661216425	5.317968145	-5.65517091	9.54E-08	1.86E-06	7.266110093
STK26	-0.665412093	6.802646228	-5.653161414	9.63E-08	1.88E-06	7.257024941
ZNF503	1.289561277	2.369906249	5.650881468	9.73E-08	1.90E-06	7.246719397
SSH2	0.718798497	5.505463186	5.650166906	9.76E-08	1.90E-06	7.243490032
DPY19L2	-1.457553067	3.546105084	-5.648689146	9.83E-08	1.91E-06	7.236812273
KIAA0040	0.731469527	4.660755946	5.632997114	1.06E-07	2.04E-06	7.16596722
RTN4R	-1.278936423	3.204676537	-5.630080959	1.07E-07	2.07E-06	7.152814665
CD300A	1.188650202	5.193974522	5.627774908	1.08E-07	2.09E-06	7.142416731
CTSL	1.46848951	3.106139485	5.627376571	1.09E-07	2.09E-06	7.140620899
SPECC1	0.71260832	4.197202171	5.618704627	1.13E-07	2.17E-06	7.101543924
RHOU	1.232440841	3.456651518	5.615109435	1.15E-07	2.20E-06	7.085354154
C19orf18	-0.772075781	2.621794338	-5.613268473	1.16E-07	2.21E-06	7.077066405
NPM2	-1.166600842	2.692981452	-5.611941164	1.17E-07	2.23E-06	7.07109207
GPSM3	0.609838506	7.358656276	5.609302358	1.18E-07	2.25E-06	7.059217101
TMIGD3	0.839791432	1.44600248	5.59785094	1.25E-07	2.37E-06	7.007723368
VWCE	-1.01694947	2.724728412	-5.593957059	1.27E-07	2.40E-06	6.990228219
MEGF6	-1.132295797	5.135140711	-5.593705584	1.27E-07	2.40E-06	6.989098595
PTPRC	0.713967695	7.534960082	5.591361746	1.28E-07	2.42E-06	6.978571615
SLA2	-0.936429945	4.712406593	-5.589079885	1.30E-07	2.44E-06	6.96832556
CXCR2	1.131885022	2.611827913	5.585478073	1.32E-07	2.48E-06	6.9521578
CLEC12B	0.899717632	2.936843801	5.579195188	1.36E-07	2.55E-06	6.923970429

ZNF551	-0.681335701	4.14984802	-5.574836848	1.39E-07	2.59E-06	6.904428611
TMEM104	0.727186101	4.783512073	5.570421588	1.41E-07	2.65E-06	6.884641049
CD83	0.876224975	4.630808514	5.565795742	1.44E-07	2.70E-06	6.863919958
GDF11	-0.719386592	3.973118774	-5.565163067	1.45E-07	2.71E-06	6.861086755
ZNF550	-0.671847879	3.978201666	-5.562893187	1.46E-07	2.73E-06	6.850923555
METTL7A	0.684295149	6.584850233	5.561688647	1.47E-07	2.74E-06	6.845531357
ANKRD36	-0.673940097	4.104311334	-5.555892578	1.51E-07	2.82E-06	6.819594838
ZYX	0.733340239	8.346953231	5.55347824	1.53E-07	2.84E-06	6.808795915
SLC25A42	-0.670601691	3.774887605	-5.550458517	1.55E-07	2.87E-06	6.795293245
POLR3G	-0.6086921	2.814148392	-5.550360532	1.55E-07	2.87E-06	6.794855177
AHDC1	-0.850939449	4.876069136	-5.549759118	1.56E-07	2.88E-06	6.792166524
CDC42SE1	0.597197079	6.876851489	5.547962979	1.57E-07	2.89E-06	6.784137846
RASGEF1B	1.029107286	2.921523395	5.54742023	1.57E-07	2.90E-06	6.781712093
EMP3	0.887326101	7.359197373	5.54706939	1.58E-07	2.90E-06	6.780144128
VIPR1	0.988800129	2.077591009	5.54246684	1.61E-07	2.95E-06	6.759580171
CIDEB	0.673195514	1.56708099	5.536269008	1.66E-07	3.02E-06	6.731905088
C20orf27	0.82862638	5.496546352	5.527963981	1.72E-07	3.13E-06	6.694850524
ASPHD2	0.628758479	2.319940704	5.523634029	1.75E-07	3.19E-06	6.675545098
IGF1R	-1.266117399	3.494910287	-5.519174225	1.79E-07	3.25E-06	6.65567043
TNS3	1.212925341	4.626499807	5.518423184	1.80E-07	3.25E-06	6.652324462
KIAA1586	-0.585841767	4.224961342	-5.517605524	1.80E-07	3.26E-06	6.648682016
PRRG4	0.908306188	4.073730879	5.516826344	1.81E-07	3.27E-06	6.645211299
MEF2D	0.628055562	6.700936633	5.516570563	1.81E-07	3.27E-06	6.644072034
MLNR	-0.819738748	1.656779304	-5.514220843	1.83E-07	3.30E-06	6.633607745
ABHD6	0.781177212	2.550325937	5.513939005	1.84E-07	3.30E-06	6.632352788
ZNF438	0.776573921	4.095194159	5.511489589	1.86E-07	3.33E-06	6.621447772
ACSS2	0.696926802	4.736895704	5.509623073	1.87E-07	3.36E-06	6.613139887
AIFM3	1.054383264	2.59887262	5.509597726	1.87E-07	3.36E-06	6.613027078
SCGB2B2	-0.656179515	1.639199995	-5.507897462	1.89E-07	3.37E-06	6.605460706
ITPKA	-0.97896377	1.855193202	-5.50020282	1.95E-07	3.49E-06	6.571236656
TENT5C	-1.13135727	4.749344928	-5.499682721	1.96E-07	3.49E-06	6.568924435
EPS8	1.301103205	2.167072984	5.499125679	1.96E-07	3.50E-06	6.566448129
CHST10	-0.67146422	3.239815041	-5.497453512	1.98E-07	3.52E-06	6.559015502
ZDHHC23	-0.878682868	3.257395735	-5.497201338	1.98E-07	3.52E-06	6.557894734
TBC1D1	0.614113359	5.301589547	5.497110379	1.98E-07	3.52E-06	6.557490479
TMSB10	0.623951987	10.85398166	5.496073993	1.99E-07	3.53E-06	6.552884717
DGAT2	0.832521859	1.544983241	5.496014391	1.99E-07	3.53E-06	6.552619858
CACNA1C	-0.735045065	1.15738422	-5.48912185	2.06E-07	3.64E-06	6.52200284
ZNF627	-0.610419547	3.483666695	-5.482853041	2.12E-07	3.73E-06	6.494177091
HAVCR2	1.180185337	4.085692229	5.480135216	2.14E-07	3.77E-06	6.482119423
PAPSS2	0.837753841	2.609208898	5.47737479	2.17E-07	3.82E-06	6.469876547
ZNF792	-0.744883405	4.022376914	-5.476931666	2.17E-07	3.82E-06	6.467911586
STK17B	0.741751992	6.587985643	5.468235533	2.26E-07	3.95E-06	6.429369929
ZBTB8A	-0.923688179	2.504547824	-5.467549201	2.27E-07	3.95E-06	6.426329691
DNAJC18	-0.606400484	2.687696674	-5.466894242	2.28E-07	3.96E-06	6.423428647
SPNS2	-1.338184274	5.127367429	-5.464896614	2.30E-07	3.99E-06	6.414581781
KIF13B	0.606475141	3.77378432	5.464781294	2.30E-07	3.99E-06	6.414071125

TMEM17	-0.61025502	1.94516242	-5.460998877	2.34E-07	4.06E-06	6.397325707
CYTL1	-2.007222648	6.140596357	-5.454691011	2.41E-07	4.17E-06	6.369415707
DLEU7	0.707901179	1.064411904	5.453045254	2.43E-07	4.19E-06	6.362137135
JPH4	0.716049745	1.408421505	5.451102361	2.45E-07	4.22E-06	6.353546197
BIN2	0.666372216	7.2474826	5.4492552	2.47E-07	4.25E-06	6.345380326
MLC1	-1.113418866	6.332371237	-5.4475031	2.49E-07	4.28E-06	6.337636293
RXFP2	1.620411936	2.850948663	5.443409682	2.54E-07	4.35E-06	6.319550001
UCP2	0.696612105	7.66296931	5.440789229	2.57E-07	4.40E-06	6.307976284
OGDH	0.592200187	6.023561353	5.440200654	2.57E-07	4.41E-06	6.305377212
TUBA4A	1.210454678	4.333136835	5.439462232	2.58E-07	4.42E-06	6.302116679
TOB1	0.694864016	4.874731783	5.438788966	2.59E-07	4.43E-06	6.299144091
ZNF726	-0.661765844	2.171217051	-5.43159608	2.68E-07	4.56E-06	6.267400523
ABCC3	0.985096889	1.29277221	5.42954344	2.70E-07	4.60E-06	6.258346646
GYG2	-1.000948069	1.3142317	-5.425596772	2.75E-07	4.68E-06	6.240944505
MSRB1	0.872824122	5.248645699	5.425589196	2.75E-07	4.68E-06	6.240911111
GCNT2	0.895852445	2.184992366	5.420141821	2.82E-07	4.79E-06	6.216904889
TTYH2	0.744543406	3.886071104	5.412265369	2.92E-07	4.95E-06	6.182220572
FMN1	0.782507506	0.923432447	5.410448765	2.95E-07	4.99E-06	6.174225561
SYTL3	0.932807801	3.348075545	5.410210003	2.95E-07	4.99E-06	6.173174874
TTYH3	0.861499798	5.7893808	5.406932223	2.99E-07	5.06E-06	6.158753768
OAS2	1.215157628	4.696756134	5.406901082	2.99E-07	5.06E-06	6.158616785
FYB1	0.803694158	6.701804837	5.403758404	3.04E-07	5.13E-06	6.14479528
NFKBIZ	0.883745468	6.31298943	5.400423304	3.08E-07	5.19E-06	6.130133018
PLXNB1	-1.216747652	2.672848689	-5.399366552	3.10E-07	5.21E-06	6.125488354
EXTL2	-0.618799699	3.824774173	-5.397262377	3.13E-07	5.26E-06	6.116241729
ACSL1	1.146413918	5.533806126	5.394261439	3.17E-07	5.32E-06	6.103058271
RNF43	-0.719812618	2.523651255	-5.386457651	3.29E-07	5.49E-06	6.068796935
HTATIP2	0.927525839	3.857149017	5.384679563	3.31E-07	5.53E-06	6.060994882
PREX1	0.703207909	6.625880698	5.383040657	3.34E-07	5.56E-06	6.053804976
CLIP2	-1.10682845	5.202856227	-5.380544258	3.38E-07	5.60E-06	6.042855891
ARPIN	0.830640499	1.133339315	5.370862025	3.53E-07	5.84E-06	6.000420373
DHRS11	-0.689457911	2.992882016	-5.362704653	3.66E-07	6.05E-06	5.964705507
PDZD8	-0.916189762	4.840937365	-5.358715798	3.73E-07	6.14E-06	5.947253866
CRIP3	0.736220369	1.167778945	5.358428927	3.73E-07	6.15E-06	5.945999092
SPTLC3	-0.928018454	1.678293324	-5.354138233	3.80E-07	6.25E-06	5.927236685
HHLA2	0.738909988	0.962824639	5.354003474	3.81E-07	6.25E-06	5.926647562
BCL2L11	0.837738965	3.444237968	5.352853809	3.83E-07	6.28E-06	5.921621987
ZNF254	-0.597884705	3.906700092	-5.351255678	3.85E-07	6.31E-06	5.91463715
SMPDL3B	-0.959885457	2.613124206	-5.344918785	3.97E-07	6.48E-06	5.886953958
PLEKHG4	-1.021429458	2.985653958	-5.340001805	4.05E-07	6.61E-06	5.865488101
ZNF318	0.662763199	4.346796393	5.339892611	4.06E-07	6.61E-06	5.865011539
FOLR3	1.205895226	1.401628761	5.339755369	4.06E-07	6.61E-06	5.864412573
TRIM7	0.664510635	0.944271274	5.339047724	4.07E-07	6.62E-06	5.86132436
CTTNBP2NL	0.837684733	1.899796714	5.336439745	4.12E-07	6.68E-06	5.84994519
CABLES1	-1.08951134	2.5999054	-5.333367856	4.18E-07	6.76E-06	5.836546413
CCDC169	-0.689311698	1.444418423	-5.329068807	4.26E-07	6.89E-06	5.817803317
C1QA	1.957228616	3.16539599	5.328474117	4.27E-07	6.90E-06	5.815211333

KLHL2	0.682631894	4.40120512	5.323989503	4.36E-07	7.03E-06	5.795670837
SEMA4A	1.011170193	5.010098683	5.32223311	4.39E-07	7.08E-06	5.788020684
SLC22A18	0.793772869	2.727337272	5.321830285	4.40E-07	7.08E-06	5.786266365
RAB9B	-0.779806005	1.819259657	-5.317680876	4.48E-07	7.21E-06	5.768200435
ZFP36L1	1.206569195	5.058603032	5.308944185	4.66E-07	7.47E-06	5.730191514
BASP1	1.673488468	4.435116186	5.308892286	4.67E-07	7.47E-06	5.729965847
CCIN	0.643658575	1.317007983	5.307209412	4.70E-07	7.52E-06	5.722649139
C11orf95	-0.71837362	3.689603382	-5.306592627	4.71E-07	7.53E-06	5.719967885
ZNF850	-0.590483481	3.422713181	-5.30297933	4.79E-07	7.64E-06	5.704264364
TESPA1	-1.078928624	5.272023924	-5.30121716	4.83E-07	7.68E-06	5.696608384
C17orf97	-0.678282418	2.744146697	-5.300621112	4.84E-07	7.69E-06	5.694019143
IL12RB1	0.930424068	3.551821413	5.297656769	4.91E-07	7.77E-06	5.68114476
JMY	-0.919444926	3.292008705	-5.297393961	4.91E-07	7.77E-06	5.680003587
C20orf203	-0.83827496	1.569777238	-5.296976998	4.92E-07	7.78E-06	5.67819311
CXorf65	-0.781226779	3.628133056	-5.290233945	5.07E-07	8.01E-06	5.648927053
RARG	0.751228966	2.52109688	5.290085655	5.08E-07	8.01E-06	5.648283714
SLCO4C1	1.164717275	2.738794491	5.288321783	5.12E-07	8.05E-06	5.640632269
PRR5	-0.785979024	3.030468842	-5.28676687	5.15E-07	8.10E-06	5.633888615
EPCAM	-1.376462453	1.649854596	-5.285533716	5.18E-07	8.14E-06	5.628541334
REXO5	-0.673958786	4.307199943	-5.266512727	5.64E-07	8.81E-06	5.546162701
TRPV4	0.632811989	0.482502313	5.260765152	5.79E-07	9.02E-06	5.521307886
SERTAD1	0.590178479	2.922549773	5.247582329	6.14E-07	9.55E-06	5.464366117
HRH2	1.273077268	4.929201733	5.247381561	6.15E-07	9.55E-06	5.463499635
RAVER2	-0.702643615	4.569672917	-5.246150861	6.18E-07	9.59E-06	5.458188591
CEP70	-0.90013787	3.708791317	-5.245918144	6.19E-07	9.60E-06	5.457184399
STXBP2	0.672437764	5.418660382	5.242629054	6.28E-07	9.72E-06	5.442994801
SETD7	0.923096216	3.020349159	5.241453026	6.31E-07	9.76E-06	5.437922644
HNMT	1.487040822	2.276150172	5.240081516	6.35E-07	9.81E-06	5.43200831
B3GNTL1	-0.614075434	4.917659041	-5.238203634	6.41E-07	9.89E-06	5.423911979
TMEM217	-0.958637563	2.770891201	-5.231536798	6.60E-07	1.02E-05	5.395183628
PRRT3	-0.724930844	2.860881034	-5.228496691	6.69E-07	1.03E-05	5.382091229
GPR84	0.912401969	1.736992922	5.226352984	6.75E-07	1.03E-05	5.372862193
HLA-DPA1	1.622854279	6.408863457	5.224317467	6.81E-07	1.04E-05	5.364101196
ERG	-1.181219247	5.243629013	-5.210591146	7.24E-07	1.10E-05	5.305080011
CLEC1B	0.907970946	3.465904803	5.204748343	7.44E-07	1.13E-05	5.279987388
ZNF320	-0.622370874	3.675807756	-5.203139034	7.49E-07	1.14E-05	5.273079231
CCDC88B	0.936018299	5.217910386	5.202882496	7.50E-07	1.14E-05	5.271978136
KIF17	1.16177306	1.541567176	5.200349101	7.58E-07	1.15E-05	5.261106388
SPACA9	-0.813950074	2.548510473	-5.195929613	7.73E-07	1.17E-05	5.242148947
CLDN23	0.610299363	0.932103604	5.19245023	7.85E-07	1.18E-05	5.227231478
CBR1	0.864175086	3.772782139	5.188311865	8.00E-07	1.20E-05	5.209497173
NKD1	-0.779328801	1.089040709	-5.187245336	8.04E-07	1.20E-05	5.204928227
GPR35	0.815786322	2.561719693	5.18334166	8.18E-07	1.23E-05	5.18821033
ZNF429	-0.716303245	3.588071789	-5.179229623	8.33E-07	1.25E-05	5.170608991
CARNS1	-0.83597793	2.28576933	-5.178776784	8.35E-07	1.25E-05	5.168671193
PI4K2A	0.870562319	2.731147188	5.177908646	8.38E-07	1.25E-05	5.164956556
PPP1R18	0.676279989	6.500101841	5.176271766	8.44E-07	1.26E-05	5.157953686

ZNF544	-0.772291185	4.52241739	-5.174057569	8.52E-07	1.27E-05	5.148483251
ZNF30	-0.599051006	2.391737753	-5.173940042	8.53E-07	1.27E-05	5.147980646
LRP10	0.713988318	5.321186168	5.171157439	8.63E-07	1.29E-05	5.13608299
HLA-DMA	1.173501813	6.903804456	5.159936975	9.07E-07	1.35E-05	5.088149796
KCTD17	0.810781964	2.526598002	5.158139648	9.14E-07	1.36E-05	5.080478048
ADD3	0.753766325	6.038658067	5.155331957	9.26E-07	1.37E-05	5.068497145
VAV2	0.927497433	3.616443724	5.152993136	9.35E-07	1.38E-05	5.058520259
ANKRD36B	-0.625075871	3.379357734	-5.151301345	9.42E-07	1.39E-05	5.051305303
RNH1	0.622103284	5.510955069	5.141131007	9.86E-07	1.45E-05	5.007964804
KCNJ2	0.714528064	0.694091132	5.132309526	1.02E-06	1.50E-05	4.970417979
LSP1	1.359290761	5.8697923	5.131850034	1.03E-06	1.50E-05	4.968463409
ZNF793	-0.938480922	1.732457968	-5.130484667	1.03E-06	1.50E-05	4.96265614
ZNF66	-0.76109145	3.833187001	-5.129570716	1.04E-06	1.51E-05	4.958769429
PTK7	-1.095706457	3.915828454	-5.128245456	1.04E-06	1.51E-05	4.953134376
COL8A2	0.998633639	1.711654353	5.125676026	1.06E-06	1.53E-05	4.942211799
DDX60L	0.725430059	4.717952687	5.124033208	1.06E-06	1.54E-05	4.935230112
CD320	-0.864727459	3.432647752	-5.123514988	1.07E-06	1.54E-05	4.933028076
PLAUR	1.077903531	4.809658668	5.12210481	1.07E-06	1.55E-05	4.927036648
ZNF43	-0.678309884	4.443733078	-5.120726395	1.08E-06	1.55E-05	4.92118122
INSL6	0.725362853	2.668830473	5.120235019	1.08E-06	1.56E-05	4.919094131
ANKRD13B	-0.750631075	3.163708925	-5.119348751	1.09E-06	1.56E-05	4.915330103
EMILIN2	0.980620978	5.968945213	5.118123993	1.09E-06	1.57E-05	4.910129194
SAMSN1	0.734027101	5.484405426	5.118059802	1.09E-06	1.57E-05	4.909856631
GPC2	-0.670392477	1.809951269	-5.111854424	1.12E-06	1.61E-05	4.883518471
SFXN3	0.906431937	5.274079278	5.105316523	1.15E-06	1.65E-05	4.855791769
TOM1	0.659286363	4.725915838	5.105031811	1.16E-06	1.65E-05	4.854584861
CARMIL2	-0.926558356	4.461667792	-5.096330874	1.20E-06	1.71E-05	4.817722648
2-Mar	0.608251316	4.658895998	5.095637767	1.20E-06	1.71E-05	4.814788035
TNKS1BP1	-0.625552718	2.922289478	-5.09051096	1.23E-06	1.75E-05	4.793089348
TMPRSS9	-0.625884922	2.897603485	-5.0869752	1.25E-06	1.78E-05	4.77813304
MTSS1	1.171933448	4.09110557	5.086774996	1.25E-06	1.78E-05	4.77728638
KCNQ5	-1.041025992	3.928254486	-5.083699307	1.27E-06	1.80E-05	4.764282121
NPW	-1.273330371	3.642083622	-5.08156676	1.28E-06	1.81E-05	4.755268607
CAMK1	0.973663964	2.334759533	5.080769785	1.29E-06	1.82E-05	4.751900718
MTHFD1L	-0.623244252	4.529466333	-5.077701499	1.30E-06	1.84E-05	4.73893792
NCEH1	0.679806527	3.871304157	5.069055775	1.35E-06	1.90E-05	4.702439713
CIB3	-0.701720656	2.106977147	-5.068145521	1.36E-06	1.91E-05	4.698599453
NLRC3	-0.752905056	4.770953717	-5.066901688	1.37E-06	1.92E-05	4.693352599
H2AFJ	1.020021374	3.124568811	5.064322676	1.38E-06	1.94E-05	4.682476298
RHOG	0.618075609	7.922617139	5.064275209	1.38E-06	1.94E-05	4.682276151
ASAP1	0.695557136	5.167334825	5.059872508	1.41E-06	1.97E-05	4.663717556
MRC1	1.659892297	2.444747647	5.050840007	1.47E-06	2.04E-05	4.625676749
AC099489.1	-1.388392521	2.892580398	-5.050659459	1.47E-06	2.04E-05	4.624916823
TAS2R5	-0.730009958	2.926068206	-5.048943577	1.48E-06	2.05E-05	4.617695597
STX3	0.702272808	4.525083153	5.044109865	1.51E-06	2.09E-05	4.597361908
ACSM3	-1.335277433	4.376431386	-5.040370489	1.53E-06	2.13E-05	4.581640616
CYBA	0.645728408	7.595451565	5.033999077	1.58E-06	2.18E-05	4.554871521

HLA-DPB1	1.455686392	7.030764599	5.031437453	1.60E-06	2.20E-05	4.544115397
ZFP2	-0.588613647	1.673371363	-5.028022099	1.62E-06	2.23E-05	4.529780204
TIMP2	0.606240621	6.460786622	5.027487148	1.62E-06	2.23E-05	4.527535458
PSTPIP1	0.927354731	4.858259303	5.0262447	1.63E-06	2.24E-05	4.522322543
CCDC57	-0.751450251	4.434190289	-5.024874975	1.64E-06	2.25E-05	4.516576619
ARMH2	0.662557399	1.745376735	5.020542997	1.67E-06	2.29E-05	4.4984111
KIT	-1.299798903	6.506151086	-5.018380608	1.69E-06	2.31E-05	4.489347364
MDFI	-1.541656132	2.764564686	-5.013255046	1.73E-06	2.35E-05	4.467873841
SLC38A5	-0.662700151	4.436262652	-5.008092141	1.77E-06	2.40E-05	4.446258757
CORO1A	0.735418121	7.907143827	5.006633412	1.78E-06	2.41E-05	4.440154336
C11orf80	-0.591399875	3.036836531	-5.006329282	1.78E-06	2.42E-05	4.438881777
CTXN1	-0.641423724	0.939489116	-5.005574121	1.79E-06	2.42E-05	4.435722209
OSCAR	1.445517086	5.371567358	5.001862545	1.81E-06	2.45E-05	4.420197773
QPRT	-0.860376789	3.746716093	-4.999923154	1.83E-06	2.47E-05	4.412088946
SASH3	0.65384342	7.225464281	4.998036892	1.84E-06	2.48E-05	4.404204283
DPEP2	0.692701756	5.382775216	4.994836114	1.87E-06	2.51E-05	4.390829469
CDC42EP2	0.682358711	2.778643968	4.991143415	1.90E-06	2.55E-05	4.375406272
HLA-DQA1	1.471111604	4.246838202	4.989311401	1.92E-06	2.57E-05	4.367757401
PARD6G	-0.643144223	1.39632659	-4.989061446	1.92E-06	2.57E-05	4.366713958
SAMD10	-0.732005282	4.277036018	-4.987014759	1.94E-06	2.59E-05	4.358171325
TREML4	0.836237546	1.200027807	4.985028402	1.95E-06	2.61E-05	4.349882764
TAF4B	-0.637186891	3.598942249	-4.980884544	1.99E-06	2.65E-05	4.332598667
NPEPL1	0.599356325	3.386423924	4.979856338	2.00E-06	2.66E-05	4.328311502
WDR49	1.308685196	3.092078391	4.976251421	2.03E-06	2.69E-05	4.31328532
IL17RE	-1.67107167	2.667896123	-4.974949397	2.04E-06	2.71E-05	4.307859969
GNRHR	-0.594328001	1.519444516	-4.972115693	2.06E-06	2.74E-05	4.296055634
ZNF385C	-1.007509604	2.269813488	-4.970183287	2.08E-06	2.75E-05	4.288008437
CENPV	-0.595786512	4.32840902	-4.968114392	2.10E-06	2.78E-05	4.279395193
FAM107B	0.819433863	5.595981479	4.967922011	2.10E-06	2.78E-05	4.278594395
HIP1	0.97762356	4.81977791	4.963676283	2.14E-06	2.82E-05	4.260926599
ST3GAL5	0.803482687	3.468989217	4.957091813	2.20E-06	2.90E-05	4.233546789
GAMT	-0.740031654	4.293350169	-4.952902145	2.24E-06	2.94E-05	4.216137954
GATA2	-1.327103233	5.438546215	-4.952500715	2.25E-06	2.94E-05	4.214470464
ZNF737	-0.852619365	3.708071005	-4.949689518	2.27E-06	2.97E-05	4.202795661
FZD7	-0.62061443	1.492248314	-4.948570789	2.29E-06	2.98E-05	4.198150868
MDK	-1.063988692	4.379403112	-4.945326865	2.32E-06	3.02E-05	4.184686618
TSPO	0.834608749	6.965112357	4.937264021	2.40E-06	3.11E-05	4.151246893
ALDH3A2	0.811063517	3.934722903	4.936864696	2.40E-06	3.11E-05	4.149591702
FZD2	1.218492486	2.761840961	4.936763921	2.40E-06	3.11E-05	4.149174007
ZNF772	-0.708104453	3.292777117	-4.935018936	2.42E-06	3.14E-05	4.141942255
MTSS2	-0.772247567	3.117139247	-4.931782078	2.46E-06	3.17E-05	4.128532331
ULK2	0.827902996	2.719540466	4.928890031	2.49E-06	3.21E-05	4.116555982
ZNF667	-0.992687282	2.064512222	-4.925964681	2.52E-06	3.24E-05	4.104446581
RTKN	-0.816070937	2.594974882	-4.924129813	2.54E-06	3.26E-05	4.096853693
ZNF285	-0.593682698	1.496337091	-4.918919479	2.60E-06	3.33E-05	4.075303262
KCNE3	0.774429695	4.904375174	4.914797574	2.64E-06	3.38E-05	4.058265689
MYO1E	0.766181223	2.069297516	4.90951254	2.70E-06	3.45E-05	4.036434667

KLK1	0.642774525	0.758223441	4.908431495	2.72E-06	3.46E-05	4.031971142
ZBTB47	0.632898677	3.103366389	4.905145084	2.76E-06	3.51E-05	4.018406004
JDP2	0.760842779	3.637464856	4.895023452	2.88E-06	3.65E-05	3.976666507
BLNK	1.454816079	3.212667832	4.890446067	2.93E-06	3.71E-05	3.957809693
SLC25A29	-0.858540345	5.665853485	-4.888151413	2.96E-06	3.74E-05	3.948361268
CRTAM	0.629312631	1.59680086	4.887974989	2.97E-06	3.75E-05	3.947634955
HACD1	-0.762824916	3.732086916	-4.88149319	3.05E-06	3.84E-05	3.920962695
NID1	1.600658949	3.717340521	4.878567954	3.09E-06	3.88E-05	3.908933448
AC092835.1	-0.587112342	1.789830455	-4.878462657	3.09E-06	3.88E-05	3.908500532
BCL2	-0.692864432	4.758614506	-4.870569368	3.20E-06	4.00E-05	3.876066671
ATP7B	-1.172870263	2.51472848	-4.870259752	3.20E-06	4.01E-05	3.874795181
ZNF154	-0.93811629	3.831573709	-4.86929958	3.21E-06	4.02E-05	3.870852424
BTG1	0.711704948	6.529325478	4.863424231	3.29E-06	4.11E-05	3.846738105
SKAP2	1.202057491	5.57343047	4.859438737	3.35E-06	4.18E-05	3.830391749
CDCA7L	-0.693076439	5.333160083	-4.852565781	3.45E-06	4.30E-05	3.80222424
SLC17A9	-0.846939736	6.230776805	-4.844916052	3.56E-06	4.42E-05	3.770905563
GCA	0.697920758	5.784868002	4.839819386	3.64E-06	4.51E-05	3.750058268
SERPINI2	1.242151176	2.748977952	4.837622736	3.68E-06	4.54E-05	3.741077807
TLCD3A	-0.61395956	2.607066279	-4.834377333	3.73E-06	4.60E-05	3.727814926
MEX3A	-0.95772759	1.82568986	-4.83149601	3.77E-06	4.65E-05	3.716045074
CERCAM	-1.042769577	3.69281394	-4.827580026	3.84E-06	4.72E-05	3.700056528
YES1	-0.845179791	3.352428673	-4.822240528	3.93E-06	4.82E-05	3.67827037
PCBP3	-1.250398598	1.799954452	-4.822071729	3.93E-06	4.82E-05	3.677581912
GPR34	0.965748196	2.784779534	4.819956867	3.96E-06	4.86E-05	3.668957698
NHSL2	1.24100294	3.70204852	4.812766117	4.09E-06	4.99E-05	3.639654076
DHCR7	-0.740503419	3.723248752	-4.809146239	4.15E-06	5.06E-05	3.624913882
SORCS2	0.891659501	1.115022906	4.806850271	4.19E-06	5.10E-05	3.615568653
ZSCAN18	-0.677900517	3.239902773	-4.794822534	4.41E-06	5.35E-05	3.566663041
G6PD	0.651952693	6.157802109	4.793195111	4.44E-06	5.38E-05	3.560052363
FNIP2	0.683092842	3.228650157	4.791711053	4.47E-06	5.40E-05	3.554025401
DOC2A	-0.875598797	2.158506533	-4.777752259	4.74E-06	5.70E-05	3.497400396
ERV3-1	-0.711070821	5.126525758	-4.777706288	4.74E-06	5.70E-05	3.497214102
SLC27A3	0.662199515	4.102293502	4.771415538	4.87E-06	5.84E-05	3.471732943
TWNK	-0.588984856	3.977734203	-4.761129126	5.08E-06	6.07E-05	3.430117526
VENTX	1.437806449	2.165945165	4.756455003	5.18E-06	6.18E-05	3.411228301
FGD6	0.827490048	1.984201415	4.755571358	5.20E-06	6.19E-05	3.407658741
GPAT3	1.279880258	3.127638741	4.75230174	5.28E-06	6.27E-05	3.394454875
ZNF528	-0.750042827	3.33942236	-4.750277987	5.32E-06	6.31E-05	3.386285432
CACNB1	0.785589819	2.781458024	4.749714976	5.33E-06	6.32E-05	3.384013115
ZNF69	-0.623197906	4.396928504	-4.749256037	5.34E-06	6.33E-05	3.382160971
MCL1	0.641683394	9.372196859	4.747094343	5.39E-06	6.37E-05	3.373438695
PLBD2	0.688601085	5.026580229	4.747044802	5.39E-06	6.37E-05	3.373238833
ANKRD28	-0.719939637	6.217980677	-4.74680866	5.40E-06	6.38E-05	3.372286194
FOS	1.01952565	9.732916802	4.744173893	5.46E-06	6.44E-05	3.361659311
DOCK10	0.813194922	4.764423056	4.743370444	5.48E-06	6.45E-05	3.358419556
SRGAP3	-0.838618208	1.559060479	-4.74207518	5.51E-06	6.48E-05	3.353197466
RELT	0.745811512	5.080728011	4.739281435	5.57E-06	6.55E-05	3.34193738

MMP17	1.186891575	2.787543631	4.738003512	5.60E-06	6.57E-05	3.336788306
BMF	0.658271136	4.491564154	4.735772072	5.66E-06	6.61E-05	3.327799609
PRKCQ	-0.765566161	4.630898548	-4.7349118	5.68E-06	6.63E-05	3.324335049
PIP4P2	0.613693165	4.405396399	4.733148851	5.72E-06	6.67E-05	3.317236536
DENND11	-0.683392117	5.59249173	-4.731566305	5.76E-06	6.71E-05	3.310865996
GSDMB	-0.657655605	3.554150877	-4.727080176	5.87E-06	6.83E-05	3.292815201
ZNF512B	-0.624925991	4.709668034	-4.726797419	5.87E-06	6.83E-05	3.291677874
TLE4	0.632545405	5.141449307	4.720804834	6.02E-06	6.98E-05	3.267585344
BIVM	-0.858610431	3.378769274	-4.718676868	6.08E-06	7.04E-05	3.25903526
MICAL3	-0.608577803	2.670084132	-4.716859907	6.12E-06	7.08E-05	3.251736925
OR52K2	1.024029168	1.435587744	4.71577175	6.15E-06	7.11E-05	3.247366985
KIF26B	0.931429322	2.257099897	4.710098848	6.30E-06	7.24E-05	3.224596606
PDXK	0.610697371	4.304045738	4.708627666	6.34E-06	7.28E-05	3.218694601
ITM2C	-1.061648038	7.346265919	-4.705412585	6.42E-06	7.36E-05	3.205801038
GIMAP7	1.260994668	4.336643265	4.704582885	6.45E-06	7.38E-05	3.202474667
SCART1	-0.636300793	1.694022584	-4.704319799	6.45E-06	7.38E-05	3.201420009
PKD2	-0.688118142	5.431163538	-4.700981946	6.54E-06	7.48E-05	3.188042847
SHANK3	-1.658537359	4.31415791	-4.69681764	6.66E-06	7.60E-05	3.17136288
ATP6V0A1	0.879104757	4.329968394	4.69666081	6.66E-06	7.60E-05	3.170734908
ARVCF	-0.846186388	2.739481951	-4.693438119	6.75E-06	7.69E-05	3.15783401
SCMH1	-0.586297732	5.806223652	-4.686548597	6.95E-06	7.90E-05	3.130275224
CHST12	-0.86331806	4.448542698	-4.685553147	6.98E-06	7.92E-05	3.12629569
CD48	1.091044598	5.177797106	4.685287092	6.99E-06	7.93E-05	3.125232175
KRT23	0.722358443	0.586116104	4.683373679	7.04E-06	7.98E-05	3.117584858
SLC7A1	-0.589348194	5.112444601	-4.679229364	7.17E-06	8.11E-05	3.101028895
PLEKHA5	-0.700704248	3.397721068	-4.67794653	7.20E-06	8.15E-05	3.095906253
MDFIC	1.086105022	4.598136091	4.676792477	7.24E-06	8.19E-05	3.091298711
CC2D2A	0.680067188	2.220180056	4.676366246	7.25E-06	8.20E-05	3.089597192
STOX2	-0.849136926	0.80288242	-4.668490045	7.49E-06	8.45E-05	3.058175077
RAC3	-0.717938456	2.965766924	-4.668039655	7.51E-06	8.45E-05	3.056379382
PELI3	0.635115016	2.21381072	4.664034206	7.63E-06	8.59E-05	3.040415125
C17orf78	-0.592848639	1.976145036	-4.66091808	7.73E-06	8.67E-05	3.028002103
LONRF3	0.93389728	2.665267586	4.656183557	7.89E-06	8.83E-05	3.009153496
SEPTIN6	-0.636310722	7.675296617	-4.6560486	7.89E-06	8.83E-05	3.008616421
WDR35	-0.873473729	3.347919093	-4.65579625	7.90E-06	8.83E-05	3.007612192
CDK14	-0.806350692	2.897264097	-4.652379433	8.01E-06	8.94E-05	2.994018756
TRIM6	-0.807592795	2.438530984	-4.652174438	8.02E-06	8.94E-05	2.993203429
HLA-B	0.646863692	9.869247975	4.650752812	8.07E-06	8.99E-05	2.987549909
AUTS2	-0.836023215	3.132849782	-4.649977259	8.09E-06	9.01E-05	2.98446621
IRS2	0.952617757	5.226814839	4.649606337	8.11E-06	9.02E-05	2.982991506
CLEC4E	1.459222756	2.27231661	4.643152014	8.33E-06	9.25E-05	2.957343903
RHOBTB1	-0.968998027	2.899972978	-4.6416859	8.38E-06	9.30E-05	2.951521524
AP5B1	0.827365427	5.689619973	4.64037856	8.42E-06	9.34E-05	2.946330788
WT1	-1.128942373	3.087281923	-4.639909226	8.44E-06	9.35E-05	2.944467573
ALOX5	1.389884288	5.458798386	4.635761636	8.58E-06	9.50E-05	2.928007819
NTNG2	-1.617652929	4.286235957	-4.633484181	8.67E-06	9.58E-05	2.918974173
GCAT	-0.795138943	3.117231465	-4.633475485	8.67E-06	9.58E-05	2.918939687

ADGRE1	1.314909156	4.210490769	4.629660459	8.80E-06	9.71E-05	2.903814289
ABHD11	0.60922574	3.123656756	4.629457702	8.81E-06	9.71E-05	2.903010667
SLC2A3	0.936626986	5.846480206	4.629112336	8.82E-06	9.72E-05	2.90164188
MRPL33	0.586292926	5.742167463	4.626580336	8.92E-06	9.81E-05	2.891609032
ANKRD36C	-0.636167012	4.523739324	-4.623621762	9.03E-06	9.92E-05	2.879890879
KIAA0895	-0.626198171	1.275711023	-4.622119673	9.08E-06	9.96E-05	2.873943536
APOL3	0.88454562	3.656558675	4.620890264	9.13E-06	0.000100087	2.869076863
GPR174	-1.019411921	4.759436512	-4.620015752	9.16E-06	0.000100284	2.865615627
IRF4	0.892349173	2.709615396	4.618191473	9.23E-06	0.000100869	2.858396814
SOX4	-0.891855385	6.618047729	-4.617538184	9.26E-06	0.000101079	2.855812192
B4GALT6	-1.072783605	4.68192712	-4.61728881	9.27E-06	0.000101079	2.854825658
STRBP	-0.598359065	4.280179183	-4.615625158	9.33E-06	0.000101687	2.848245147
STMN1	-0.618950912	6.836649088	-4.613219968	9.42E-06	0.000102502	2.838734509
ADM	0.90017701	1.610964733	4.612141906	9.47E-06	0.000102844	2.834472769
ARHGEF40	0.75706173	4.899342421	4.611002207	9.51E-06	0.000103214	2.829968143
NAT14	-0.590585433	3.191038422	-4.60692198	9.67E-06	0.000104675	2.813847693
SERINC2	1.124837258	1.985150598	4.606378684	9.69E-06	0.000104794	2.811701971
FKBP4	-0.604015851	5.613180428	-4.593423002	1.02E-05	0.000110031	2.76058776
VASH1	0.633139379	3.20983687	4.590259484	1.04E-05	0.000111284	2.748122359
ATP9A	-1.100105965	1.684709966	-4.589556485	1.04E-05	0.000111466	2.745353123
DUSP1	0.887432987	9.045362733	4.588835546	1.04E-05	0.000111658	2.742513538
ASNS	-0.694012743	4.08049919	-4.586671793	1.05E-05	0.000112441	2.733993014
JUNB	0.861232823	8.415425677	4.584280848	1.06E-05	0.000113396	2.724581191
OTULINL	1.235355534	4.087141556	4.583172629	1.07E-05	0.000113789	2.720219946
KCNC4	-0.595302597	2.444618195	-4.582101606	1.07E-05	0.000114166	2.716005794
UGT3A2	-1.629293885	1.916854298	-4.578008483	1.09E-05	0.00011585	2.699907122
SLC36A1	0.810863125	4.715602341	4.573545733	1.11E-05	0.0001178	2.682366441
PALM	-1.264674129	2.882472177	-4.571148925	1.12E-05	0.000118559	2.672950945
CDH24	-0.856473117	2.585694145	-4.566440649	1.14E-05	0.000120498	2.654465534
TAS2R41	0.808354856	1.580204981	4.56200909	1.16E-05	0.000122576	2.637079075
ZBED3	-0.748100147	4.343554387	-4.55770365	1.18E-05	0.000124354	2.620199063
SLC9B2	-0.585847434	3.698090096	-4.556823795	1.19E-05	0.000124669	2.616750896
C9orf64	0.701781993	3.040312385	4.549403931	1.23E-05	0.00012779	2.587691394
TSPAN5	-0.825931619	2.341970518	-4.54145289	1.27E-05	0.000131419	2.556589485
PDE7B	0.693239911	1.409501934	4.54055066	1.27E-05	0.00013161	2.553062732
HSPA1A	0.623394507	4.869914883	4.540321488	1.27E-05	0.00013161	2.552166996
LTB4R	0.683736756	5.596236737	4.540314489	1.27E-05	0.00013161	2.552139638
GNPDA1	0.829435618	4.096552253	4.531730087	1.32E-05	0.000135659	2.518610415
PPM1J	0.738276246	0.789126003	4.52994748	1.33E-05	0.000136505	2.511653605
FAM171A2	-0.746247377	1.353417162	-4.521878663	1.37E-05	0.000140701	2.480188977
VIM	0.878271994	9.52145313	4.521721469	1.37E-05	0.000140717	2.479576397
ADAMDEC1	0.845568839	1.087830147	4.520832785	1.38E-05	0.000141079	2.47611351
KANK2	-0.781017052	3.565330098	-4.519742686	1.38E-05	0.000141558	2.471866457
SLC14A1	-0.945249525	2.359903018	-4.512435443	1.42E-05	0.00014553	2.443416414
TBXAS1	0.655105542	5.664427734	4.512276101	1.43E-05	0.000145548	2.442796406
CDKN1A	1.106535231	5.00246818	4.511357537	1.43E-05	0.000146016	2.439222517
ARL5C	0.814816058	1.271552011	4.510690633	1.43E-05	0.000146337	2.436628104

ARHGEF17	-0.99165636	3.981079527	-4.509026436	1.44E-05	0.000147023	2.430155193
FGF17	-0.824943362	1.475995399	-4.505196389	1.47E-05	0.000148943	2.415264779
SLC22A16	-0.634467433	4.65636483	-4.501437015	1.49E-05	0.000151079	2.400658059
HLA-DRB5	1.524097014	6.977939748	4.496813737	1.52E-05	0.000153704	2.382706861
NAA80	-0.633327732	5.054192677	-4.495708611	1.52E-05	0.000154267	2.378417876
IGFLR1	0.589577354	3.143027408	4.494103822	1.53E-05	0.000155164	2.372191071
ADGRA2	1.155823576	3.721480476	4.491545614	1.55E-05	0.00015654	2.362268206
HLA-DQB2	1.282364291	2.484444103	4.491374443	1.55E-05	0.000156567	2.361604409
LRRC56	-0.677402839	2.549040542	-4.4909249	1.55E-05	0.000156772	2.359861181
CPED1	0.845820652	1.204859892	4.486342634	1.58E-05	0.000158727	2.342099393
TAGLN	0.633492774	3.701632326	4.485849626	1.59E-05	0.000158913	2.340189183
MAP1A	-1.259743399	4.023047146	-4.484993898	1.59E-05	0.000159341	2.336873936
EBF4	-0.861517202	1.0456705	-4.484883812	1.59E-05	0.000159341	2.336447477
CCL3	0.889251778	2.613314957	4.483827996	1.60E-05	0.000159861	2.33235776
ZNF711	-1.638607523	2.511757879	-4.482061949	1.61E-05	0.000160679	2.325518522
MPL	-1.363432602	4.319707809	-4.481443469	1.62E-05	0.000161	2.323123843
IRF7	0.793246086	5.416582637	4.479245868	1.63E-05	0.000162274	2.314616953
ZNF117	-0.676618205	4.523526919	-4.473968307	1.67E-05	0.000164705	2.294199992
ZBTB46	-0.669392686	3.313750995	-4.471522953	1.68E-05	0.000166155	2.28474576
RGL1	0.727542927	1.362122904	4.468923513	1.70E-05	0.000167742	2.274699934
INKA1	-0.786743587	5.022102734	-4.467269643	1.71E-05	0.000168527	2.268310586
SLC7A5	-0.89282885	5.700666571	-4.46624733	1.72E-05	0.000169031	2.264361978
PRRT4	-1.429573403	2.861537933	-4.461684277	1.75E-05	0.000171941	2.246745568
AMIGO1	-0.674998844	1.893867609	-4.461304444	1.75E-05	0.000171945	2.245279753
BDH1	-0.595395756	3.636756221	-4.458016581	1.78E-05	0.000173806	2.232595364
CPAMD8	0.592602814	0.830138972	4.452763883	1.81E-05	0.000177265	2.212344936
ADGRA3	-0.755309016	3.629538884	-4.45216488	1.82E-05	0.000177516	2.210036745
ICAM1	0.82600524	4.030184653	4.451660493	1.82E-05	0.000177713	2.20809332
SLC4A3	0.838732125	1.116011062	4.451316659	1.83E-05	0.000177713	2.206768606
AGPAT3	0.712240428	4.471736908	4.451270069	1.83E-05	0.000177713	2.206589111
ITM2A	-1.472725345	6.526220397	-4.448128893	1.85E-05	0.00017953	2.194490429
CCND2	-0.959072569	6.526745153	-4.447830779	1.85E-05	0.000179657	2.193342525
PLCL2	0.677611219	4.307011269	4.444128649	1.88E-05	0.000182086	2.179091974
PIK3C2B	-0.706189723	3.798085672	-4.438212704	1.92E-05	0.000185555	2.156337862
PLXDC2	1.019629124	4.021922973	4.431349164	1.98E-05	0.000190283	2.129966919
FGFR3	-0.767579631	1.59126999	-4.427730777	2.01E-05	0.000192787	2.116076483
LDLRAD3	1.385986563	2.518757348	4.42733539	2.01E-05	0.000192999	2.114559158
ENC1	0.787453688	3.249103841	4.422843138	2.05E-05	0.000195936	2.097326808
ACP6	-0.778010174	2.566904261	-4.417548901	2.09E-05	0.000199657	2.077034534
HSPG2	-1.015911743	4.318884605	-4.412327897	2.14E-05	0.000203336	2.057040446
RNF144A	-0.602130244	4.818229883	-4.411145362	2.15E-05	0.000203979	2.052514289
NEDD4	-0.70743033	3.871216406	-4.411082088	2.15E-05	0.000203979	2.05227213
ARAP3	1.036907321	2.995085263	4.409557674	2.16E-05	0.000205057	2.04643881
CXCR4	0.813035231	8.165035875	4.405171659	2.20E-05	0.000208086	2.029663556
CHST2	0.848233971	3.517207301	4.404725526	2.20E-05	0.000208357	2.027957914
LILRA4	1.11200299	1.542147071	4.403856788	2.21E-05	0.000208982	2.024636946
SYNGAP1	-0.657876606	4.707050942	-4.403073471	2.22E-05	0.000209537	2.021642935

FAM117A	-0.69165739	6.741325759	-4.401397643	2.23E-05	0.000210438	2.015238865
NFATC2	-0.693262669	4.40658915	-4.39795608	2.26E-05	0.000213152	2.002092787
MYO1G	0.671147128	6.308411448	4.397538062	2.27E-05	0.000213405	2.000496559
SNTB2	0.640657214	2.000947153	4.392717684	2.31E-05	0.000217247	1.982097718
MROH7	-0.617001734	1.836548805	-4.391689992	2.32E-05	0.000217826	1.978177057
SPTBN2	-0.630975608	1.840542003	-4.390903092	2.33E-05	0.000218408	1.975175482
EPDR1	-0.694593822	4.904265331	-4.390527239	2.33E-05	0.000218631	1.973741956
TAS2R4	-0.611856405	2.336710112	-4.389078554	2.34E-05	0.000219795	1.968217428
BSPRY	-0.791329362	4.017532361	-4.388036086	2.35E-05	0.000220501	1.964242834
PLCB2	0.764755902	7.018468536	4.38698761	2.36E-05	0.00022132	1.960246039
GPT2	-0.952556885	3.792895552	-4.385856684	2.37E-05	0.000222003	1.955935729
CDH26	-0.674599098	2.038311497	-4.382116346	2.41E-05	0.000225021	1.941685998
ADAMTS14	-1.067756711	2.073883366	-4.380351317	2.43E-05	0.000226179	1.934964806
FAM114A1	0.644544777	1.904552111	4.378745076	2.44E-05	0.000227525	1.928850012
LOXL1	-0.801424381	1.060238705	-4.375963614	2.47E-05	0.000229845	1.9182652
SOD2	0.643436059	4.396461445	4.375128835	2.48E-05	0.000230282	1.915089428
CHD5	-0.712341716	1.113779197	-4.369042497	2.54E-05	0.000235154	1.891948576
TMEM38A	0.667887531	1.364118067	4.365163096	2.58E-05	0.000238478	1.877211155
PCSK4	-0.696583528	2.204446189	-4.355413288	2.68E-05	0.000246279	1.840215517
GCSAML	-1.381977215	3.751802647	-4.352882824	2.71E-05	0.000248067	1.830623699
FLNA	0.664546577	8.61089773	4.348528175	2.75E-05	0.00025157	1.814126919
PNPLA6	0.598464595	5.187376501	4.345807133	2.78E-05	0.000253948	1.803824977
PSRC1	0.679255698	2.831525531	4.34356216	2.81E-05	0.000255863	1.79532905
GOLGA8O	-0.7122215	2.147618627	-4.341587839	2.83E-05	0.000257274	1.787860082
RAB38	-0.941062766	2.658579175	-4.340585934	2.84E-05	0.000257939	1.784070781
PLAGL1	-0.606596613	5.676442381	-4.338107	2.87E-05	0.000259762	1.774698012
DEPP1	0.806712255	3.176148014	4.333788893	2.92E-05	0.000263882	1.758380896
SAMD11	-0.949229871	1.983054079	-4.328928911	2.98E-05	0.000268516	1.740030621
MAPK12	-0.710006016	3.516221513	-4.327416008	3.00E-05	0.000269881	1.73432134
TSPAN13	-1.033193143	4.022557672	-4.324129547	3.03E-05	0.000273037	1.721924263
ARPP21	-1.056055083	0.984070109	-4.323665692	3.04E-05	0.000273413	1.72017509
DEPTOR	-0.869905778	5.004020847	-4.320438124	3.08E-05	0.000276673	1.708007969
BLVRA	1.033815727	4.257384028	4.317404	3.12E-05	0.000279755	1.696576254
MPP7	0.898914803	3.002767384	4.314665004	3.15E-05	0.000282672	1.686261642
RENBP	0.69127543	4.268942176	4.313394926	3.17E-05	0.000283702	1.681480391
ZNF681	-0.64858059	2.512587035	-4.31321257	3.17E-05	0.000283776	1.680793991
PTK6	0.639924897	2.341885982	4.312744473	3.17E-05	0.00028404	1.679032144
DSCC1	-0.597618894	3.874759759	-4.309992436	3.21E-05	0.000286619	1.66867678
SH3BP1	0.654660211	5.49889499	4.302317108	3.31E-05	0.000294493	1.639822121
ADM2	-0.637836589	1.00698596	-4.30079424	3.33E-05	0.000295727	1.634101611
BAG2	-0.613475954	1.86554928	-4.300001806	3.34E-05	0.000296469	1.631125506
ARL10	-0.781809779	2.584327761	-4.299927935	3.34E-05	0.000296469	1.630848091
THBD	0.801667322	2.282469083	4.298967861	3.35E-05	0.000297364	1.627242986
NFKB2	0.692392737	4.401280987	4.298932647	3.35E-05	0.000297364	1.627110769
KIAA1549	-0.815773354	2.775702783	-4.295383071	3.40E-05	0.000301006	1.61378731
CD59	-1.029570676	4.113391864	-4.293538262	3.42E-05	0.000302103	1.606866004
TMEM154	0.616111369	3.804759958	4.288336583	3.50E-05	0.000307476	1.587362462

AMN	-0.882648205	2.833381152	-4.285602201	3.53E-05	0.000310009	1.577117074
IFIT2	0.802165846	2.80014252	4.284539387	3.55E-05	0.000311011	1.573136161
ADPRH	0.708551538	3.419419479	4.282956649	3.57E-05	0.000312549	1.567209177
ADAM15	0.5979363	5.668057705	4.27505869	3.68E-05	0.00032154	1.537657677
CXCL10	0.863965553	1.240527128	4.272854524	3.72E-05	0.000323901	1.52941773
LDLR	0.859018629	4.004546918	4.271332581	3.74E-05	0.000324919	1.523730034
GNA15	-0.71884738	7.052386109	-4.271258251	3.74E-05	0.000324919	1.523452293
STMN3	-0.778417955	5.057844312	-4.268018475	3.79E-05	0.000328199	1.511350052
P2RY6	0.675182091	1.272966713	4.263241662	3.86E-05	0.000333925	1.493518756
TAS2R3	-0.716743217	1.919131534	-4.257408206	3.95E-05	0.000340326	1.471763478
KIF7	-0.739367817	1.933474746	-4.2547512	3.99E-05	0.000342976	1.461861865
LRRC70	-0.627898402	2.779028295	-4.252427127	4.02E-05	0.000345654	1.453204773
NEK6	0.694830236	5.480415464	4.248075084	4.09E-05	0.000350819	1.437003135
TXNRD3	-0.692547721	2.520456627	-4.247077716	4.11E-05	0.000351881	1.433291924
MAPK11	-0.729652435	2.951805427	-4.244488747	4.15E-05	0.000354838	1.423661422
OCIAD2	-0.611968456	3.532009149	-4.234732352	4.31E-05	0.000367499	1.387409129
PEAK3	0.665473406	4.075506288	4.234534531	4.32E-05	0.000367622	1.386674724
BTG2	0.71836443	6.686992103	4.227790508	4.43E-05	0.00037611	1.361653291
CPA3	-1.829712571	6.534279463	-4.223826601	4.50E-05	0.000380971	1.346960587
TSPAN14	0.614556521	4.480868472	4.220286824	4.56E-05	0.000385425	1.333848769
POLE	-0.59792045	5.262275707	-4.214587538	4.67E-05	0.000393733	1.312755289
CAMK2D	0.812354763	2.243508408	4.214463902	4.67E-05	0.000393752	1.31229794
NUDT10	-1.074307292	1.030073846	-4.213411598	4.69E-05	0.000395026	1.308405729
ITPR2	-0.588008504	5.164091276	-4.213130838	4.69E-05	0.000395286	1.307367391
IER5L	0.586459331	2.07073051	4.210186489	4.75E-05	0.000399322	1.296481424
IL6ST	0.777944116	4.461585045	4.205591554	4.83E-05	0.000405819	1.27950435
KIF26A	-0.855189933	1.292643292	-4.203205272	4.88E-05	0.000409071	1.270693201
OBSCN	-0.864805628	2.813105167	-4.201436452	4.91E-05	0.000411182	1.264164425
TLN1	0.586138389	7.945676009	4.200480205	4.93E-05	0.00041218	1.26063575
PRKAR2B	1.054021702	4.443503494	4.198924674	4.96E-05	0.000414144	1.254896937
MYC	-0.758541604	7.091357899	-4.197579315	4.98E-05	0.000415777	1.249934811
TCN2	1.135727532	3.927236873	4.191273317	5.11E-05	0.000424354	1.226692286
BLVRB	0.888992694	5.750857815	4.185221493	5.23E-05	0.000433034	1.204411514
ADCY7	0.831032346	5.104499544	4.179348516	5.35E-05	0.000441201	1.182812558
LMNA	1.199047489	4.744709858	4.179190542	5.35E-05	0.000441201	1.182231898
SV2A	-0.98023075	4.218343853	-4.176311025	5.41E-05	0.000444826	1.171650664
ZNF391	-0.779111897	1.881863542	-4.175992849	5.42E-05	0.000445186	1.170481817
SNX24	0.649394769	1.910797624	4.167081128	5.61E-05	0.000459239	1.137771339
GASK1A	-0.627838334	0.661179444	-4.165087962	5.65E-05	0.000462399	1.130462698
SERPINB9	1.031453868	4.441255004	4.164631686	5.66E-05	0.00046302	1.128789975
ZNF415	-0.708136793	1.61923127	-4.164183142	5.67E-05	0.000463628	1.127145734
SPINT1	1.002368501	3.530351217	4.163706083	5.68E-05	0.000464093	1.125397114
MAP3K6	0.745526086	3.358507655	4.160808694	5.75E-05	0.000469116	1.114780249
SPPL2B	-0.608003414	5.457581431	-4.155925179	5.86E-05	0.000475445	1.096898387
GBP2	0.870811687	4.547173252	4.15252076	5.93E-05	0.000479724	1.084441962
AKAP7	0.791686781	2.953978147	4.148559448	6.02E-05	0.000485488	1.06995772
CTSF	-0.955374424	3.211782352	-4.147140005	6.06E-05	0.000487544	1.064770198

DNM1	-1.064655664	4.057263856	-4.146826493	6.07E-05	0.000487729	1.063624616
DKK2	0.737302853	0.697040809	4.144654457	6.12E-05	0.000490903	1.055689731
FYN	0.684851383	4.568401008	4.143603061	6.14E-05	0.000492192	1.051849912
ZNF471	-0.612712443	2.225187738	-4.139616358	6.24E-05	0.000498772	1.037296764
HK1	0.63995854	5.685478198	4.134883074	6.35E-05	0.000506266	1.020032173
NR5A1	-0.686348521	1.107224275	-4.132841284	6.40E-05	0.000509836	1.012589428
SMO	-0.86915505	1.925346113	-4.131874868	6.42E-05	0.000511523	1.009067623
FSTL3	-0.617055491	4.390491605	-4.127230767	6.54E-05	0.000519663	0.992152412
MAST4	-0.772914835	3.570545019	-4.126824924	6.55E-05	0.00052026	0.990674904
FKBP10	-0.664310553	0.984147583	-4.124176848	6.62E-05	0.000524717	0.981037052
DLL3	-0.774467042	0.596299306	-4.115960544	6.83E-05	0.000539091	0.951163405
SSBP2	-0.670077862	4.658625492	-4.113433401	6.90E-05	0.000543013	0.941984149
SEPTIN5	-0.901641615	2.960497231	-4.106928746	7.07E-05	0.000554681	0.918377372
CALR	-0.653677578	9.999773734	-4.104058197	7.15E-05	0.000560323	0.90796865
SIX3	-1.286652098	0.922184357	-4.103966295	7.15E-05	0.000560323	0.907635502
AIG1	-0.90657777	2.800833549	-4.103309115	7.17E-05	0.000561507	0.905253369
NTN1	-0.834835347	2.36018805	-4.099237792	7.28E-05	0.000569395	0.890502239
SDC3	0.879287411	1.507383271	4.098860454	7.29E-05	0.000569759	0.889135646
LAPTM4B	-1.291902533	4.376953899	-4.098253881	7.31E-05	0.000570851	0.886939042
ADCY9	0.883792084	3.463010723	4.090864383	7.52E-05	0.000585304	0.86019923
MDGA1	-0.901519087	1.844816551	-4.079825058	7.84E-05	0.000605853	0.820321188
HLA-F	0.783875178	4.464092342	4.068632275	8.18E-05	0.000626981	0.779973494
GPHN	-0.711571721	2.787123357	-4.068470645	8.19E-05	0.00062712	0.779391475
DAGLA	0.838237277	2.025476757	4.066528349	8.25E-05	0.000630525	0.772398804
PLA2G4C	0.588034413	0.996607746	4.065751852	8.27E-05	0.000632139	0.769603975
VCL	0.613929672	5.852328408	4.063022141	8.36E-05	0.000637726	0.759782246
TXK	-0.747374455	4.256650874	-4.062412971	8.38E-05	0.000638726	0.757591094
RASL11A	0.60443741	1.794539179	4.056973191	8.55E-05	0.000649989	0.738035748
CERS4	-1.012212272	3.396296174	-4.044500546	8.96E-05	0.000677481	0.693274508
ZBTB38	0.683413395	2.782506436	4.04145399	9.07E-05	0.000683745	0.682357358
ISYNA1	-0.723050334	4.911684487	-4.038566325	9.17E-05	0.000689545	0.672015458
ANKRD18B	-1.202661602	1.795131628	-4.035347432	9.28E-05	0.000696481	0.660494041
PHOSPHO1	0.920568889	2.673896969	4.033827871	9.33E-05	0.000699683	0.655057531
TANC2	-0.61494863	2.503127694	-4.030086858	9.47E-05	0.000707462	0.641680122
DYRK3	-0.759505757	2.885520274	-4.028461007	9.53E-05	0.000710727	0.635869274
SERPING1	-1.177349866	5.191094808	-4.027201523	9.57E-05	0.000713841	0.631369079
LRRC28	-0.64953701	3.402168341	-4.022523152	9.74E-05	0.000724832	0.614662604
ABCA2	-0.779475066	5.238064219	-4.019224689	9.86E-05	0.000733118	0.602892834
RNF144B	0.89567335	3.712354852	4.012848257	0.000101026	0.000748371	0.580161309
RALGPS2	-0.615883034	3.944833935	-4.011767067	0.000101438	0.000751138	0.57630972
SOWAHD	0.606297163	2.833821014	4.009197997	0.000102424	0.000757572	0.567161003
ARMH1	-1.041062106	6.269399984	-4.007581865	0.000103049	0.000760718	0.561408124
ADAM19	0.763966578	2.795049451	4.002216897	0.000105149	0.000773026	0.542323594
SPTBN1	-0.653873366	5.084002893	-4.000916814	0.000105664	0.000775933	0.537701866
PTGS2	1.072077261	3.796980922	3.997368128	0.000107082	0.000784275	0.525092409
IL1R2	1.216442788	1.392422779	3.99559538	0.000107797	0.000788401	0.51879661
HSH2D	-0.797841381	7.758139136	-3.995569947	0.000107808	0.000788401	0.518706303

TBKBP1	-0.594654681	4.541557365	-3.990776671	0.000109765	0.000800307	0.501694259
OGDHL	-0.63616557	0.498057497	-3.985712397	0.000111869	0.000813213	0.483737676
KLF2	0.876005705	6.947316338	3.981367699	0.000113705	0.000824403	0.468346674
CLEC4G	0.747038805	1.395207483	3.970934905	0.000118232	0.000852145	0.431442178
TRPS1	1.087766171	2.871140871	3.96378563	0.000121433	0.000872307	0.406196292
LYST	0.76466	6.006503642	3.9598891	0.000123212	0.000882157	0.392451629
PLD4	1.1363595	4.14240671	3.952473636	0.000126667	0.000904894	0.366323433
FZD1	0.585790895	2.104285752	3.952072358	0.000126856	0.000905916	0.36491063
EMB	0.595111652	7.644681348	3.951366412	0.00012719	0.000907834	0.362425442
INTU	-0.667796857	1.331442177	-3.951268067	0.000127237	0.000907834	0.362079261
MARCKS	1.087250515	3.66690323	3.948441673	0.000128584	0.000916513	0.352132991
TPPP3	1.092714244	2.138262478	3.94821189	0.000128694	0.000916684	0.351324613
KIF1B	0.675768677	3.468558758	3.945054259	0.000130216	0.000925829	0.340219794
PLCH1	-0.842054572	1.413101817	-3.944681796	0.000130396	0.000926775	0.338910367
GIMAP6	0.852911443	4.684548374	3.929407787	0.000138013	0.000972387	0.285296692
CEP85L	0.668580298	2.765529398	3.924880168	0.000140351	0.000986004	0.26943553
SULT1B1	0.781117576	3.370757209	3.923901368	0.000140861	0.000988816	0.266008483
TTLL10	-0.670279691	0.967940429	-3.922896602	0.000141387	0.000991494	0.262491221
COL23A1	-1.396595276	3.257935805	-3.921537317	0.000142102	0.000995427	0.257734063
PRKCA	0.873226702	3.400145147	3.919893978	0.00014297	0.001000787	0.251984512
GPR162	0.845952257	3.580561944	3.918964647	0.000143463	0.001003157	0.248733902
IRX5	-1.019307077	1.109250947	-3.916157487	0.000144962	0.001011824	0.238918702
SYDE1	-0.661989846	1.161576645	-3.914823137	0.00014568	0.001015379	0.234255103
IL15	0.73050633	1.293970894	3.910844212	0.000147842	0.001028233	0.220356038
LGALS9	0.673502329	6.14920072	3.903924186	0.000151673	0.001052249	0.196209682
GOLGA8A	-1.029151774	4.397500854	-3.900063292	0.000153852	0.001065921	0.182752318
TUBB4A	-0.89718488	1.257275098	-3.898882357	0.000154524	0.00106974	0.178638196
MMP15	-0.876679649	1.838019207	-3.898159731	0.000154937	0.001071835	0.176121208
EIF4E3	0.864562159	4.232426005	3.897848373	0.000155115	0.001072306	0.175036827
ID2	0.949637662	5.499037713	3.894881107	0.000156823	0.001082574	0.164706004
RNASE2	1.248633375	9.271069258	3.893386357	0.00015769	0.001087017	0.159504237
TAFA5	-0.750047093	0.629435339	-3.891556618	0.000158758	0.001093989	0.153138842
LRATD2	-0.761152359	2.00643579	-3.889092019	0.000160207	0.001103193	0.144568587
DTNA	0.752524537	0.95046538	3.887999336	0.000160853	0.001107252	0.140770325
CARD9	0.809011015	4.349421058	3.886194173	0.000161927	0.00111228	0.13449726
NCR3LG1	-0.701038495	2.522108946	-3.879561302	0.000165929	0.001138169	0.11146733
PADI4	0.955219514	4.937899704	3.874965614	0.000168758	0.001153504	0.095528893
ZBED2	-0.99344832	3.000289049	-3.873580013	0.000169619	0.001158174	0.09072638
NOG	-1.188504065	3.194090998	-3.873423052	0.000169717	0.001158435	0.090182434
KIF16B	0.739956912	2.908928276	3.872718071	0.000170158	0.001159577	0.087739558
UNC13B	-0.9558419	1.926376316	-3.871302528	0.000171045	0.001164231	0.082835529
GRIFIN	-0.759269699	2.151746092	-3.863221981	0.000176195	0.001193858	0.054868287
FKBP14	-0.719710247	2.549013151	-3.862299562	0.000176792	0.001196238	0.051678679
CLEC4C	1.086824589	1.212924428	3.861214477	0.000177497	0.001199757	0.047927366
AVPR1B	-0.696578458	1.22038524	-3.854508962	0.000181914	0.001225892	0.024763824
GYPC	-0.869472856	6.100321526	-3.852984981	0.000182932	0.001232211	0.019503827
CPNE3	0.770132956	4.862912629	3.84594879	0.000187704	0.00126086	-0.004760107

PHKA1	-0.808237719	1.245446021	-3.845073413	0.000188305	0.001264031	-0.007776342
ACOT11	-0.688998294	3.701998296	-3.843562557	0.000189349	0.001268844	-0.012980923
AP1S3	0.678138316	2.66902886	3.840533104	0.000191457	0.001280641	-0.023411871
PXN	0.596228554	5.381144489	3.840465635	0.000191504	0.001280641	-0.023644106
MFSD2B	-0.805949919	2.713887815	-3.83903565	0.000192507	0.001286023	-0.028565465
TMEM176B	1.3928183	3.247649078	3.831835974	0.000197633	0.001312162	-0.053321381
ABCA7	0.708858683	5.174789045	3.830741513	0.000198424	0.001316064	-0.057081429
ADRA2A	-0.950881962	1.54071635	-3.825218288	0.00020246	0.001338724	-0.076043591
MYCN	-1.28048974	3.690663	-3.820436238	0.000206017	0.001357172	-0.092443611
PLA2G4A	0.854193382	4.231244421	3.817116126	0.000208521	0.001371349	-0.103820327
SLC44A1	0.878722382	4.757534426	3.815904684	0.000209442	0.001375547	-0.107969502
SYNJ2	-0.686789209	3.157471227	-3.810726628	0.000213423	0.001397444	-0.125692475
COCH	-0.803171539	3.131143279	-3.810303707	0.000213751	0.001399123	-0.127139162
TRPM2	0.702804514	4.831524928	3.808617788	0.000215064	0.001406773	-0.132904926
GSAP	0.595969497	3.924007113	3.805379218	0.000217609	0.001420551	-0.143974984
TIE1	-0.845733083	2.950883905	-3.804026798	0.000218679	0.001426185	-0.148595597
ACSM1	-1.125178082	3.342376946	-3.803467371	0.000219124	0.001427092	-0.150506525
TMEM176A	1.14890545	2.231555427	3.801503078	0.000220691	0.00143682	-0.157214513
USP41	-0.687098359	1.479656364	-3.794071216	0.000226719	0.001470046	-0.182569061
PAQR8	0.619640896	3.869196863	3.794000342	0.000226777	0.001470046	-0.182810663
GPR141	0.778709741	4.217098093	3.784364127	0.000234827	0.00151667	-0.215626149
VANGL2	-0.856060298	1.568131374	-3.783976335	0.000235156	0.001517791	-0.216945353
TBL1X	0.645456745	4.41197325	3.783837085	0.000235275	0.001518052	-0.217419032
BTBD11	-0.833881637	3.271351937	-3.780742921	0.000237921	0.001532588	-0.227940696
TFRC	-0.623931209	6.667029328	-3.778626444	0.000239748	0.001541723	-0.235133788
ARMC5	-0.609232091	4.455654674	-3.772812199	0.000244834	0.001568811	-0.2548776
TOM1L1	-0.60090641	1.219158872	-3.772331158	0.00024526	0.001571019	-0.256510013
SLC16A2	-0.760224111	0.861410117	-3.769773795	0.000247533	0.001580898	-0.265185634
HCST	0.680482521	6.63175077	3.757446741	0.000258774	0.00164245	-0.30693798
SRGN	0.982641257	11.38826126	3.754981562	0.00026108	0.001656002	-0.315274521
KCNA5	0.650118189	0.55076681	3.753822184	0.000262171	0.001661787	-0.319193694
ASIC1	-0.669647437	1.040589693	-3.747305134	0.000268384	0.001691854	-0.341205945
NDRG2	0.648550005	1.993951837	3.744981478	0.000270633	0.001702722	-0.349047
DDIT4L	-0.796249124	0.90757825	-3.74303461	0.000272531	0.001712449	-0.355613598
DCLK2	-0.658764602	1.174903951	-3.740518754	0.000275002	0.001724081	-0.36409528
PLS1	-0.762121186	1.675780465	-3.739386359	0.000276122	0.001729984	-0.36791142
SEMA7A	-0.748168186	2.825752264	-3.736965875	0.000278529	0.001743381	-0.376065272
ZSCAN23	-0.672067388	1.096632294	-3.733782069	0.000281725	0.001760556	-0.386784056
SCNN1D	-0.590215921	2.840228125	-3.728355916	0.000287253	0.001788782	-0.405035139
ZNF626	-0.758161397	1.804012548	-3.727855829	0.000287768	0.001790841	-0.406716129
UGT2B28	-0.76546874	1.216189243	-3.727692791	0.000287936	0.001791313	-0.407264122
AKR1B1	0.674632757	4.398770547	3.71833039	0.000297738	0.001842284	-0.438700239
JUP	-1.009516339	6.129561431	-3.710558496	0.000306114	0.001887512	-0.464747573
PLEKHA8	-0.606161136	2.04364349	-3.707395404	0.000309587	0.001907715	-0.47533604
NEXN	0.729651235	2.352830559	3.707162733	0.000309844	0.00190809	-0.476114617
SRC	0.823420101	3.125824526	3.706629978	0.000310433	0.001911113	-0.477897211
ITPRIPL2	0.678198741	3.531187896	3.706340606	0.000310753	0.00191248	-0.47886536

MSRB3	-0.725889236	5.761998138	-3.705513005	0.000311671	0.001916312	-0.481633924
HIVEP3	0.587454532	3.378427903	3.703033096	0.000314438	0.001931489	-0.489926941
COL9A2	0.861023703	3.427958233	3.700482686	0.000317307	0.001944815	-0.498451054
PPFIA4	0.646896467	1.273983735	3.691487819	0.000327625	0.002003008	-0.528476328
STARD8	0.837704209	3.45278422	3.690807737	0.000328417	0.002005965	-0.530744074
CEACAM19	-0.717425502	1.978615498	-3.687022589	0.000332864	0.002029937	-0.543359558
LGR4	-0.680987636	1.207562001	-3.685786211	0.000334328	0.002036954	-0.547478003
TNFAIP3	0.614574408	5.355255476	3.67913605	0.000342312	0.002079733	-0.56961092
B3GALNT1	-0.61937142	3.494199047	-3.674585572	0.000347878	0.002109598	-0.584737095
MYO1B	-0.647830636	1.270944236	-3.673131904	0.000349674	0.00211983	-0.58956602
TGFB1I1	-0.585915298	1.863124898	-3.669943042	0.000353645	0.002139903	-0.600153651
HOXB2	1.257992195	3.181611844	3.669381814	0.000354348	0.002143492	-0.602016262
RTP4	0.744406393	2.912683516	3.663710548	0.000361529	0.002178804	-0.620825223
MOK	-0.604464867	1.809779604	-3.661377028	0.000364524	0.002193454	-0.628557581
STXBP1	-0.728918148	1.702174164	-3.643343991	0.000388469	0.002323174	-0.688177182
GABBR1	1.078841159	3.468308797	3.643078185	0.000388833	0.002324635	-0.689054185
BCL3	0.65294287	4.891348244	3.642880225	0.000389104	0.002324878	-0.689707301
MT1E	0.79629686	1.58692208	3.64128123	0.000391301	0.002335087	-0.694981713
TNFSF9	0.782727947	2.29438405	3.639160147	0.000394233	0.002347546	-0.701975374
NDFIP1	0.763653703	5.279553457	3.637338191	0.000396769	0.002359034	-0.707980105
SASH1	0.611847477	0.656065402	3.636941368	0.000397323	0.002360887	-0.709287612
CCDC188	-0.610206048	2.569436446	-3.636566962	0.000397847	0.002363277	-0.710521153
TSPAN16	0.632939839	2.333898415	3.631618181	0.000404829	0.002394521	-0.726816006
THBS1	1.290388367	3.197779175	3.630220292	0.000406822	0.002405579	-0.73141557
HMGN5	-0.66348358	3.312352424	-3.627348863	0.000410946	0.002425992	-0.740859094
CD52	1.087179433	7.030646135	3.624219221	0.000415485	0.002447136	-0.751144907
TRAF5	-0.618819404	4.128676608	-3.617739045	0.000425035	0.002494323	-0.772419533
CPXM1	-0.8271127	6.970110228	-3.614798956	0.000429435	0.002513328	-0.782061716
SH3D21	-0.602717707	3.11421584	-3.612816595	0.000432427	0.00252858	-0.788559374
GABARAPL1	0.632842078	4.156290138	3.61176007	0.000434029	0.0025364	-0.792021197
TBC1D16	-0.682240918	2.497811255	-3.610310169	0.000436238	0.00254701	-0.79677062
ZNF721	-0.686750443	3.680593962	-3.610102839	0.000436554	0.002548094	-0.79744964
NLGN2	-0.632901757	2.067602948	-3.609062264	0.000438147	0.002555089	-0.800857122
DOCK3	-0.720360123	1.164095548	-3.603626175	0.000446555	0.002600227	-0.81864519
GSTM2	-0.667366079	3.408183972	-3.601862976	0.000449315	0.002611608	-0.824410064
HELZ2	0.692389093	3.37990549	3.600855535	0.000450899	0.002620033	-0.827702913
PTGFR	0.619666403	0.644411715	3.596096921	0.000458453	0.002655206	-0.843246417
BST2	0.59465449	7.070199666	3.594031247	0.000461769	0.002672734	-0.849988496
OR2W3	-0.737092445	3.611189127	-3.591266215	0.000466243	0.002694712	-0.859008235
FKBP5	0.60614032	6.11079954	3.58827902	0.000471123	0.002719685	-0.868746313
DLL1	-0.709838937	1.247493019	-3.583863563	0.000478425	0.002752551	-0.883128313
TRO	-0.85868445	1.48178273	-3.574082926	0.00049498	0.002835532	-0.914934175
BEND6	-0.794394213	1.326802914	-3.571754769	0.000498999	0.002851005	-0.922494678
RGS3	-0.589680336	3.462897387	-3.566530453	0.000508131	0.002896819	-0.939445544
SCN9A	-1.064357314	1.226376003	-3.566486593	0.000508209	0.002896819	-0.939587768
COL2A1	-0.8137677	0.759519545	-3.559659647	0.000520382	0.002954954	-0.961707683
SYTL1	-0.587733151	5.611400073	-3.554577026	0.000529622	0.003000423	-0.978153264

CCL23	1.196547157	2.730429215	3.547364063	0.000543002	0.003062847	-1.001458799
STAP1	-0.674011993	4.332773869	-3.5353212	0.000566053	0.003168987	-1.040283399
PLTP	-0.659553232	5.006503034	-3.534739767	0.000567189	0.00317241	-1.042155115
TIMP1	0.75221611	7.216851635	3.53395815	0.00056872	0.003179341	-1.044670857
MTCL1	-0.60567344	1.310708706	-3.531094017	0.000574361	0.003204434	-1.05388555
TWIST1	-0.640019967	0.560463643	-3.528876306	0.000578765	0.003222538	-1.061016305
OR2L5	-0.623493579	1.344645844	-3.52845824	0.000579599	0.003225335	-1.06236013
CDCA7	-0.770820332	5.705821933	-3.521969564	0.000592686	0.003289576	-1.083200408
STK32B	-0.918865557	2.871828833	-3.520516112	0.000595656	0.00330149	-1.087864266
CTSW	-1.060631025	7.182552098	-3.514206723	0.000608709	0.003364236	-1.10809154
AK5	0.819643068	1.976428208	3.514088547	0.000608956	0.003364646	-1.108470116
HBEGF	0.969520914	4.001547146	3.5136394	0.000609896	0.003367926	-1.109908856
ZNF354C	-0.649977987	2.017775396	-3.50589414	0.000626318	0.003440064	-1.134695208
MYO1C	0.609329707	2.470039854	3.505653885	0.000626834	0.003441926	-1.135463352
A4GALT	-0.820683914	1.735869847	-3.505313526	0.000627566	0.003442119	-1.13655147
ZNF718	-0.708286248	3.456205514	-3.504455012	0.000629415	0.003450257	-1.139295733
BAHCC1	-0.940448334	5.7857261	-3.503085973	0.000632374	0.003459721	-1.143670754
GCH1	0.728033831	4.423953165	3.502214604	0.000634265	0.003468049	-1.146454644
C2	0.843389682	1.535116029	3.499058835	0.000641156	0.003495905	-1.15653205
ECHDC3	0.661980562	2.124525787	3.487542081	0.000666906	0.003617054	-1.193245198
EVPL	-1.056305071	2.310778271	-3.485740413	0.000671021	0.00363836	-1.198979517
OSM	-0.769367046	3.850349015	-3.481871906	0.000679938	0.00368158	-1.211283859
SLC24A3	-1.111901982	2.735013431	-3.481192859	0.000681515	0.00368704	-1.213442498
SLC27A2	-0.686475216	3.911035762	-3.476134156	0.00069337	0.003739737	-1.229512788
ANKRD22	0.734135492	2.133950583	3.473144007	0.000700468	0.003770428	-1.239002684
LTK	-1.072905919	3.495519566	-3.472457414	0.000702107	0.00377744	-1.241180784
LAMC3	-0.6258243	0.867619632	-3.47116387	0.000705205	0.003790966	-1.245283364
GOLGA8B	-0.895469602	3.711290853	-3.468213564	0.000712321	0.003821826	-1.254635766
RHOH	-0.5972725	3.814512814	-3.465351751	0.000719287	0.003850708	-1.263701351
PLAAT5	-0.675005534	0.676961559	-3.463562766	0.000723674	0.003865913	-1.269365311
ZFPM1	-0.60514183	3.276688699	-3.459679122	0.000733285	0.003910585	-1.281652653
PTGER1	-0.633475112	1.337479036	-3.457763296	0.00073807	0.003931794	-1.28770987
SORBS3	0.706167299	3.553110195	3.452447447	0.000751501	0.003993506	-1.304502277
WASF1	-0.872050399	4.650835299	-3.448724203	0.000761045	0.004033208	-1.316250988
ST3GAL4	-0.638656653	4.944078384	-3.446201421	0.000767576	0.004060081	-1.324205661
TFR2	-0.805162608	3.502509664	-3.443481779	0.000774676	0.004082101	-1.332775642
CD96	-1.220247454	5.686555911	-3.442717891	0.000776681	0.004089346	-1.335181755
SERPINB6	0.856067146	5.624853555	3.439154004	0.000786101	0.004133352	-1.3464015
LOXL4	-0.760236396	2.402937218	-3.430245113	0.000810121	0.00423675	-1.374406023
LPAR1	0.733072239	1.597047974	3.42958709	0.000811922	0.00424161	-1.376472082
KCTD15	-0.686411873	3.55893941	-3.428561189	0.000814737	0.004253537	-1.379692543
PTGDR2	-0.758003503	2.228842356	-3.426239954	0.000821142	0.004273714	-1.3869763
LPAR5	-0.682736831	2.771396118	-3.424122956	0.000827024	0.004296285	-1.39361561
ARHGAP4	0.715143974	6.870642265	3.421883195	0.00083329	0.004326528	-1.400636208
DIPK1B	-0.824862653	4.657268312	-3.410207657	0.000866683	0.00447959	-1.437171594
SCUBE1	0.955950114	1.953840674	3.408428467	0.000871881	0.004505257	-1.442729946
CIITA	0.952201926	5.634339601	3.404016537	0.000884896	0.004558976	-1.456502782

GGT1	0.686372954	3.058957359	3.403951775	0.000885088	0.004558976	-1.45670484
CD33	0.673926882	5.67655106	3.401269955	0.00089309	0.004594111	-1.465069347
ACCS	-0.620083799	5.216566894	-3.400348163	0.000895856	0.004604688	-1.467943113
CACNA2D4	0.832624532	3.664286755	3.398159081	0.000902456	0.00463372	-1.474765167
PODXL2	-0.836663969	2.556256237	-3.398072767	0.000902718	0.004633839	-1.47503408
SH2D3C	0.624336849	5.395263747	3.3979081	0.000903216	0.004635175	-1.475547089
CYSTM1	0.612775123	3.2275145	3.391161223	0.000923864	0.004726183	-1.496548684
ADAMTS3	-0.724236264	0.989517068	-3.388122688	0.000933307	0.004766971	-1.505995612
SMARCD3	0.706699005	3.307489448	3.383018787	0.000949373	0.0048376	-1.521847929
CD69	-0.880085256	6.592090314	-3.382778008	0.000950137	0.004839444	-1.522595277
KCNK6	0.632939499	3.561846924	3.382669789	0.000950481	0.004839444	-1.522931161
IGSF9B	-0.707362138	1.386928831	-3.381092875	0.000955502	0.004858654	-1.52782447
DST	-0.696914698	1.940013917	-3.380564406	0.00095719	0.004864412	-1.529463931
PTGR1	-0.624871632	2.188644271	-3.375158807	0.000974621	0.004934713	-1.546221335
PLA2G7	0.641380899	1.0873972	3.368202108	0.000997491	0.005024897	-1.567754146
HYAL3	-0.662866482	4.370707	-3.366069919	0.001004601	0.005049805	-1.574346384
IQSEC3	-0.882155424	2.325788061	-3.363540436	0.001013097	0.005082459	-1.582162432
ATP1B1	-0.680995959	4.400641163	-3.362310818	0.001017251	0.005098043	-1.585960149
EPB41L2	0.920907304	3.406046479	3.35950241	0.001026799	0.005141921	-1.594629647
JAM3	-0.610374474	2.86170811	-3.355330902	0.001041136	0.005197667	-1.607495801
P2RX5	-0.607771753	3.049827086	-3.353286864	0.001048229	0.005223699	-1.613795326
MANSC1	0.616583871	1.859654071	3.343024987	0.00108453	0.005377058	-1.645372747
CCDC102A	-0.625462803	2.587155784	-3.338198641	0.001102007	0.005449821	-1.660196093
KIAA1217	-0.638393216	0.850982508	-3.337522589	0.001104475	0.005460644	-1.662271042
PRODH	-0.708495375	1.304075704	-3.336225731	0.001109226	0.005481346	-1.66625039
ATXN1	0.635030102	3.163401902	3.336113741	0.001109637	0.005481986	-1.666593964
FAM180B	-0.735291246	2.566146029	-3.330342505	0.001131019	0.005575885	-1.684286479
SLAMF7	0.72606755	3.272258668	3.329053416	0.001135847	0.005594426	-1.688234839
COL4A5	-1.270920186	2.475411776	-3.326793662	0.001144357	0.005630641	-1.695153157
NDFIP2	-0.633112765	1.580092693	-3.325135241	0.00115064	0.005656819	-1.70022796
DMXL2	0.85390282	5.466347361	3.323881136	0.001155414	0.005672994	-1.704064136
NFIL3	0.704275838	5.427649714	3.322873	0.001159264	0.005688168	-1.707147034
STAR	-0.64233683	3.802772077	-3.322008076	0.001162577	0.005701548	-1.70979136
FBLN1	-0.686733034	0.894773634	-3.319815299	0.001171015	0.005734262	-1.71649273
ZNF300	-0.61935164	2.376962239	-3.315618694	0.001187325	0.005802449	-1.729307636
EDA2R	-0.784204915	2.526304305	-3.31486004	0.001190296	0.005814049	-1.731622834
C3AR1	0.883624825	5.340604467	3.313513392	0.001195587	0.005832572	-1.735731323
MAML2	0.632866722	3.097252776	3.312910662	0.001197963	0.005841231	-1.73756974
TMIGD2	-1.090223026	3.539304104	-3.307245443	0.001220504	0.005935941	-1.754835719
RTN1	0.599054698	0.898680262	3.306112542	0.001225059	0.005952473	-1.758285493
SLC29A2	-0.686388301	2.857766256	-3.305913083	0.001225863	0.005954891	-1.758892756
ZNF662	-0.797261118	2.834679325	-3.302367151	0.001240232	0.006009689	-1.769683417
ARMCX1	-0.624620121	3.42662247	-3.300457848	0.001248034	0.006039974	-1.775489594
NNMT	-0.609641803	1.293469789	-3.299382788	0.001252447	0.006056813	-1.778757594
NR4A2	0.858396241	5.150946496	3.298816256	0.001254778	0.00606658	-1.780479396
VWA1	-0.618783253	1.440964319	-3.294485181	0.001272737	0.006147295	-1.79363414
PLAU	-0.62934851	3.820487481	-3.290634444	0.001288904	0.006209973	-1.805317714

CLIP4	0.74952	3.102890036	3.285093494	0.001312506	0.006308066	-1.822109344
ARHGAP5	-0.813003398	3.32960725	-3.284891688	0.001313373	0.006309117	-1.822720458
HNRNPLL	0.76049761	2.904487968	3.281366837	0.001328605	0.006375995	-1.833389392
KLF11	0.784425853	4.067129356	3.279599399	0.001336305	0.006408206	-1.838735388
TRIM58	-0.678041298	4.712087492	-3.276594828	0.00134949	0.006457113	-1.847817772
HOXB4	1.221235793	3.062580435	3.273789307	0.001361911	0.006498968	-1.856292106
TNFRSF25	-0.682691081	3.337614165	-3.270194714	0.001377982	0.006554761	-1.867140932
TNFRSF18	-0.8515358	1.787986613	-3.262457307	0.001413176	0.006699245	-1.890458962
DSC2	-0.917948655	1.648103244	-3.257499146	0.001436167	0.006793343	-1.905376667
CARMIL1	0.614478107	1.908409503	3.257060393	0.001438218	0.006799196	-1.906695828
ZNF358	-0.605172292	3.901636617	-3.230551586	0.001567344	0.007309013	-1.986117873
HOXB3	1.39512226	3.63238762	3.228342284	0.001578579	0.007356118	-1.992712215
AKR1C3	-0.650370055	3.623506845	-3.22731287	0.00158384	0.007373569	-1.995783508
DACH1	0.9909765	3.149712919	3.220038766	0.00162148	0.007525402	-2.017462339
COL14A1	-0.622536729	1.121359659	-3.216249875	0.001641415	0.007608845	-2.028737806
CLEC2B	0.747239405	5.137752506	3.200500211	0.001726748	0.007943857	-2.075486597
DOC2B	-0.812453587	1.270610463	-3.199010514	0.001735029	0.007973145	-2.079898261
IGLL1	-1.334905862	6.01328752	-3.197149757	0.001745426	0.008009989	-2.085406343
ZNF257	-0.627238635	2.263021889	-3.197109558	0.001745651	0.008009989	-2.085525306
GPC6	-0.648885546	0.652427234	-3.179004587	0.001849885	0.008422731	-2.13897513
PHACTR3	0.64690485	0.739498618	3.17280157	0.001886907	0.008579256	-2.157228207
AFF2	-0.825016451	2.184508503	-3.170400382	0.001901423	0.008631137	-2.164285797
EPSTI1	0.778142099	2.661008052	3.169756352	0.001905333	0.008640827	-2.166177959
SCML2	-0.597383856	4.209285307	-3.168663318	0.001911988	0.008664947	-2.169388545
SERPINB10	0.947547588	3.449507579	3.159185385	0.001970602	0.008889179	-2.197188528
STON2	-1.007857394	3.673383176	-3.152792818	0.002011073	0.009046576	-2.215898518
OBSL1	-0.861991308	3.252662719	-3.151621223	0.002018574	0.009074023	-2.219324067
RAB7B	-0.915928365	4.760865621	-3.149636042	0.002031342	0.009120885	-2.225125918
ZNF90	-0.682819565	2.100727804	-3.146935039	0.002048835	0.009180365	-2.23301479
PALD1	-0.770276113	3.008189849	-3.143713304	0.002069883	0.009259752	-2.242416996
CAVIN3	0.635042307	0.941463796	3.14095826	0.002088041	0.009321697	-2.250450679
UNCX	-0.885497188	0.81513349	-3.135527904	0.002124265	0.009459542	-2.266267878
P3H3	-0.647824745	1.525808491	-3.133774615	0.002136085	0.009503477	-2.271369735
GXYLT2	-0.657665636	0.921449953	-3.126545174	0.002185468	0.009687747	-2.292380679
LAT2	0.648271589	6.505965975	3.122742326	0.002211868	0.009784712	-2.303416195
CEACAM3	0.615254147	1.080493125	3.114901611	0.002267235	0.00997973	-2.32613283
AGRN	-0.716809303	3.325018785	-3.105125385	0.002338068	0.010233648	-2.35438842
TNNT1	0.796656182	1.50051106	3.103450584	0.002350406	0.010276096	-2.359221326
FSCN1	-0.761356634	5.694196	-3.101772097	0.002362832	0.010318832	-2.364062619
SOX18	-0.618316395	1.330773369	-3.08924535	0.002457509	0.010641549	-2.40012258
NR4A1	0.851529981	3.42797839	3.088434172	0.002463759	0.010656757	-2.402453336
APBA1	-0.629815815	0.90610745	-3.088069987	0.00246657	0.010666545	-2.403499576
SPATC1L	-0.86254811	3.297581519	-3.075837712	0.002562725	0.011013833	-2.438579024
RAB11FIP5	0.605606748	1.325299792	3.07482825	0.002570813	0.011043715	-2.441468576
BEX4	-0.592272262	5.877174841	-3.068495921	0.002622091	0.011219413	-2.459575993
ADAM8	0.762815067	5.263469026	3.068172703	0.002624733	0.011226738	-2.460499378
ZBTB16	-0.704841493	4.115980247	-3.066069327	0.00264199	0.011277283	-2.466506349

NETO1	-0.631164549	0.646035055	-3.065449167	0.002647098	0.011296608	-2.468276767
ATP8B4	-0.607941877	6.143161683	-3.064890018	0.002651712	0.011308856	-2.46987275
DYSF	0.859673264	4.462922695	3.064247374	0.002657023	0.011329024	-2.471706739
KIAA1211	-0.75627754	2.35159748	-3.063414914	0.002663918	0.011348476	-2.474081938
MEST	-0.955610442	5.134202781	-3.061390713	0.002680752	0.011402718	-2.479855119
NRP1	0.707251803	1.411776292	3.061205287	0.002682299	0.011406805	-2.480383802
OR2L2	-0.589382462	1.655025492	-3.055710131	0.002728522	0.011562943	-2.496038981
TNS1	-0.60438718	3.160243055	-3.047659425	0.002797571	0.011819524	-2.518930789
MEF2C	0.709698859	4.916349615	3.047574048	0.002798312	0.011820088	-2.519173274
POGLUT2	-0.67839613	1.910666561	-3.046942156	0.0028038	0.011838133	-2.520967775
CEACAM4	0.724684069	4.750793339	3.044020358	0.002829309	0.011925136	-2.529261169
MARCKSL1	-0.628632383	6.111103551	-3.041806582	0.002848779	0.011991616	-2.535540291
MMP25	0.632860018	3.091352037	3.037640441	0.002885756	0.012104493	-2.547346354
CAPG	0.789793226	6.121946485	3.035594303	0.002904078	0.012169112	-2.553139598
SELENOP	-0.901706216	3.157296277	-3.01994282	0.003047824	0.012689486	-2.597341879
ABCB1	-0.810697227	2.38853912	-3.016534535	0.003079985	0.012801474	-2.60694116
LRG1	0.722333669	3.325351741	3.007620829	0.003165579	0.013076681	-2.632001754
IGFBP2	-1.069404894	3.598587067	-3.007609901	0.003165686	0.013076681	-2.632032437
MFGE8	-0.730576074	3.694619358	-3.001759213	0.003223056	0.0132686	-2.648446412
MX1	0.792704549	3.124276432	2.998568366	0.003254747	0.013369727	-2.657386564
SYCP2L	-0.632263529	1.015778967	-2.984420832	0.003398747	0.013859938	-2.696925732
SPIB	0.656713688	2.103675986	2.980370197	0.003441044	0.014015749	-2.708216399
PPARGC1A	0.624112493	0.889990421	2.980348034	0.003441277	0.014015749	-2.708278138
RUBCNL	0.666849081	2.384711312	2.979047488	0.003454961	0.014063911	-2.711900403
YPEL4	-0.5924277	1.848303647	-2.977525802	0.003471035	0.014122169	-2.716136836
PARP8	0.588105169	5.256291548	2.976638186	0.003480442	0.014154527	-2.718607123
TMEM51	0.738623595	2.779503402	2.971356257	0.003536913	0.014336248	-2.733293783
ADAMTSL4	0.653363088	5.039732256	2.96971684	0.003554611	0.014395991	-2.737847642
BAG3	0.65050649	1.951949149	2.966706845	0.003587318	0.014499078	-2.746202898
OLR1	0.60405058	1.181428371	2.956036054	0.00370551	0.01490168	-2.775763773
RGS10	0.630188441	5.368390318	2.951440139	0.003757511	0.015085858	-2.788467034
CPNE7	-0.891921161	2.695989104	-2.951019717	0.003762301	0.015101975	-2.789628235
GATM	-0.748201199	1.948833415	-2.947070968	0.003807567	0.015261637	-2.800527576
SLC16A9	-0.727813919	1.694195762	-2.946322152	0.003816207	0.015286823	-2.802593023
SALL4	-0.638499447	1.054159873	-2.944340843	0.003839155	0.01534852	-2.808055821
SLC18A2	-0.821891862	2.198108042	-2.937998041	0.003913476	0.015593006	-2.825522404
HAL	0.754744285	4.330075439	2.930712936	0.004000468	0.015887572	-2.845543255
TNFRSF4	-0.836884046	2.225036141	-2.926763514	0.004048367	0.016041788	-2.856378852
ZNF595	-0.805699434	2.182410457	-2.92346361	0.004088792	0.016175308	-2.865422638
TREM2	0.620050165	1.131619772	2.921486023	0.004113195	0.016245744	-2.870838177
P2RY2	0.688303064	4.103501454	2.920427499	0.004126312	0.016294245	-2.873735583
LAMC1	-0.96378293	2.380417352	-2.913907771	0.004207954	0.016576272	-2.891561208
PXDN	-1.08278517	1.715984747	-2.912671581	0.0042236	0.016621083	-2.89493715
TMEM121B	0.73773769	2.213717132	2.900928233	0.004374916	0.017085262	-2.926944746
RETREG1	-0.636726388	2.140024477	-2.900776153	0.004376908	0.017089611	-2.927358511
NME8	0.586816428	3.519589316	2.887660104	0.00455185	0.017646269	-2.96297185
TOX	-0.647307024	3.88739124	-2.883704114	0.004605859	0.017826877	-2.973685546

STARD9	-0.66050941	2.826181571	-2.87405811	0.004740021	0.018233013	-2.999754986
FGFR1	-0.844328848	2.744647083	-2.87043713	0.004791301	0.018405069	-3.009521291
STAB1	1.289247435	6.411409774	2.870365759	0.004792317	0.018405069	-3.00971368
LBP	-0.638516329	1.065092764	-2.858301885	0.004966883	0.018929799	-3.042172839
LPO	-1.063440016	3.69212961	-2.854619481	0.005021316	0.019084924	-3.052056784
ZNF727	-0.760302385	1.481359932	-2.854204965	0.005027477	0.019103171	-3.053168684
IRX1	-0.86982698	1.24955302	-2.853895349	0.005032084	0.019110763	-3.053999109
TRAT1	-0.681829153	3.178373106	-2.851356996	0.005069997	0.019213659	-3.060804253
COL24A1	-0.638264564	3.950625816	-2.850888236	0.005077027	0.019236554	-3.062060381
ATP10A	0.831066066	2.886096562	2.849855083	0.005092552	0.019284115	-3.064828265
EREG	1.23646027	2.976147968	2.847431002	0.00512915	0.019396793	-3.071319063
ECE1	0.602626898	3.4831055	2.840752824	0.005231222	0.019720956	-3.089175597
ARTN	0.773800034	3.043498168	2.840086487	0.005241508	0.019752079	-3.090955264
XK	-0.754355698	2.448518363	-2.836287056	0.005300511	0.019947386	-3.101095829
F2R	-0.699883405	4.166835941	-2.833099758	0.005350475	0.020081049	-3.109593399
SH3BGRL2	-0.592097777	1.931223576	-2.832847208	0.005354452	0.020092102	-3.110266356
FOXO1	0.642612727	3.636399279	2.829994545	0.005399566	0.020230179	-3.11786402
LHX6	-0.681160756	1.146463466	-2.823901768	0.005497085	0.020518876	-3.134068649
NYNRIN	-0.660054904	3.708938249	-2.816479865	0.005618049	0.020887895	-3.153766627
EBF3	-0.589640605	1.144859796	-2.815744881	0.00563016	0.020916935	-3.155714808
PTK2	0.734526255	1.83990499	2.809169188	0.005739571	0.021262528	-3.173124636
GGT5	-0.736429415	2.879328843	-2.806186325	0.005789839	0.021391735	-3.181010212
SDC4	0.625605602	2.737607391	2.805234933	0.005805957	0.021443142	-3.183523782
GRB10	-0.635201567	4.299281878	-2.790114652	0.006067659	0.022228284	-3.223370184
ADGRG1	-0.868332864	4.137731711	-2.787841594	0.006107918	0.022358932	-3.229343881
PRR16	0.733155741	1.153693386	2.77599451	0.006321711	0.022977712	-3.260408719
HOXB7	0.645939905	1.161021691	2.773644019	0.00636493	0.023108497	-3.266558122
ADRA2C	-0.66966146	0.935869611	-2.771387171	0.00640668	0.023234065	-3.272458187
TSPOAP1	-0.654800954	5.985814107	-2.765951791	0.006508255	0.023523523	-3.28665039
EGFL7	-0.862286408	5.570095359	-2.765265183	0.00652119	0.023557148	-3.288441419
TMCC2	-0.7756229	2.896690797	-2.761758678	0.006587614	0.023757407	-3.297582044
LHFPL6	-0.653102135	2.477433964	-2.735022797	0.007114657	0.025329534	-3.366937326
TRIM10	-0.669983106	1.824556382	-2.730015203	0.007217529	0.025642832	-3.379860766
BEX3	-1.021809406	4.16084159	-2.725902932	0.007303016	0.025852247	-3.390457825
ASS1	-0.727031318	1.255468962	-2.721301486	0.007399761	0.026119961	-3.402298594
TMTC2	0.612550907	3.325428293	2.719648538	0.007434797	0.026224605	-3.406547728
MMP19	0.693119126	2.295819826	2.719565367	0.007436564	0.026226083	-3.40676147
ADAMTS1	-0.662382932	1.05159075	-2.718695284	0.00745507	0.026258039	-3.408997155
KLF9	0.597374079	3.230904468	2.714701543	0.007540554	0.026508097	-3.419250928
GABRE	-1.011044624	1.220181643	-2.706556793	0.00771765	0.027006919	-3.440120612
AFF3	0.653073301	3.505241375	2.705151179	0.007748592	0.027085971	-3.443716625
ALDH2	0.639773112	2.725193719	2.696452673	0.007942587	0.027625153	-3.465933148
AIF1L	-0.879032156	3.062323124	-2.696177951	0.007948784	0.027636831	-3.466633767
HOXB5	1.005939164	2.123626153	2.693767758	0.008003348	0.02776206	-3.472777692
PRAME	-1.125799281	2.004672261	-2.68974979	0.008095065	0.028025255	-3.483009158
SELL	0.712787048	8.182077092	2.687371073	0.00814981	0.028174686	-3.489059964
PTGIR	0.707484434	2.96723982	2.684999484	0.008204725	0.028319254	-3.495087875

CST7	-0.86549141	7.705449987	-2.684368419	0.008219394	0.028349771	-3.496691064
HBB	-1.327046385	9.703705774	-2.683041525	0.008250315	0.028441297	-3.500060875
CD24	-0.777534837	2.384555366	-2.670030598	0.008559133	0.029318909	-3.533024808
FLNB	0.721972375	4.791199567	2.663745049	0.008712035	0.029724996	-3.548898259
ZNF135	-0.61993949	2.062446038	-2.656834771	0.008882982	0.03021021	-3.566310753
RUNX3	-0.5918302	5.399924886	-2.653386332	0.008969418	0.03047127	-3.574984967
GFI1	-0.663150223	5.958782307	-2.641099248	0.009283608	0.031354591	-3.605809864
RBPMS2	-0.764313911	2.149465048	-2.636513011	0.009403405	0.031681139	-3.61728259
GBP1	0.697998995	2.531349265	2.634237763	0.009463353	0.031839012	-3.622967612
TGM5	-0.585407427	1.127525146	-2.63007269	0.009573987	0.032155643	-3.633363212
HOXB6	1.022205673	2.072474022	2.623779218	0.009743372	0.032623196	-3.64904307
ME3	-0.590322638	3.522845287	-2.621974622	0.009792437	0.032746692	-3.653532904
HBA2	-1.264211892	7.210575017	-2.620788582	0.009824806	0.032822442	-3.65648226
MYO5C	-0.711113531	3.023256683	-2.618528203	0.009886762	0.032984122	-3.662099885
COBLL1	0.634732588	1.981388628	2.618079126	0.009899113	0.03300835	-3.663215438
MED12L	-0.673835249	2.127656439	-2.617008332	0.009928619	0.033084061	-3.665874708
ZNF532	0.592131046	1.594009767	2.609198801	0.010146219	0.033670775	-3.685239763
POU4F1	-0.953087863	0.93443827	-2.606232156	0.01023	0.033902553	-3.692582426
KCNK17	-0.920635967	3.565807905	-2.595537755	0.010537213	0.034760836	-3.718989483
TP53INP2	0.743971857	4.191302918	2.593475512	0.010597401	0.034935697	-3.724070422
DLC1	-0.649370626	2.652653113	-2.592339043	0.010630701	0.035033606	-3.726868892
MMP2	-0.972204682	3.786392557	-2.585739624	0.010825941	0.035544301	-3.743097635
RASGRP3	-0.606613057	3.537339474	-2.584958812	0.010849253	0.035591111	-3.745015279
CRISPLD1	-0.605088213	2.462416263	-2.584210684	0.010871632	0.035646496	-3.746852162
RHEX	-0.814867359	5.979941335	-2.580562629	0.01098135	0.035976576	-3.755802392
SULT1C4	-0.618027779	2.575292191	-2.579526645	0.01101269	0.036060397	-3.758342025
HBM	-0.89034167	2.946183295	-2.576008604	0.011119713	0.036349676	-3.766959362
ANO7	-0.831334124	2.672652679	-2.565701631	0.011438666	0.037248402	-3.792144937
CFH	-0.667544879	1.901724829	-2.554931781	0.011780698	0.038168206	-3.818364232
CROCC2	0.712388136	1.467934263	2.551113862	0.011904133	0.038487197	-3.82763508
COL5A1	-0.706465996	1.635796742	-2.546132964	0.012066913	0.038916494	-3.8397111
ZNF608	-0.693716957	2.604334456	-2.539067422	0.012301244	0.039508533	-3.856804682
NRXN2	-0.695978147	3.537083538	-2.532597364	0.012519393	0.040057118	-3.872419945
SUCNR1	-0.712157814	4.773387365	-2.52432746	0.012803271	0.040751005	-3.892326591
SPRY2	-0.683434865	4.097991068	-2.523033678	0.012848199	0.04086727	-3.895435549
HBA1	-1.01978923	4.083901644	-2.518876275	0.012993528	0.041248623	-3.905416016
SERPINE1	-0.675320465	2.85918471	-2.513813338	0.013172497	0.041694339	-3.917550222
TCEAL9	-0.81038849	3.783111195	-2.508338478	0.013368506	0.042211768	-3.930646781
NPDC1	-0.902886304	4.190072767	-2.503885858	0.013529835	0.042634636	-3.941278931
IKZF2	-0.752450525	2.828191736	-2.501053739	0.013633353	0.042874119	-3.948032669
CLDN10	-0.627349086	1.193738077	-2.493073146	0.013928872	0.043648285	-3.967026652
ME1	0.622970344	1.68275917	2.482224163	0.014339778	0.044734223	-3.992759104
GPR153	-0.641758961	2.280817935	-2.471051405	0.01477422	0.045874656	-4.019152964
S100B	-0.872722216	3.067827632	-2.462487997	0.015115103	0.04671789	-4.039309376
GUCY1A1	-0.778064545	4.864443092	-2.461077089	0.015171935	0.046856982	-4.042624242
HBG1	-0.717232002	1.465428326	-2.455976115	0.015378994	0.04740618	-4.054594337
NXF3	-0.785060756	2.594140845	-2.45124631	0.01557323	0.047840875	-4.06567324

ABLIM1	-0.614820217	3.562666107	-2.451225306	0.015574098	0.047840875	-4.065722394
SIGLEC12	0.728206913	3.247483145	2.446711849	0.015761492	0.048309756	-4.07627632
KLF1	-0.696012952	3.720762642	-2.44533787	0.015818936	0.048432425	-4.079485613
HLA-DOA	0.737490358	3.8670778	2.441548501	0.015978328	0.048820579	-4.088328178
TRH	-1.29260723	3.452692483	-2.439956851	0.016045703	0.049003355	-4.092038598
DNAJC12	-0.63099998	2.026617617	-2.438803612	0.016094677	0.049106682	-4.094725627
HBD	-0.925731644	6.009335817	-2.437737505	0.016140069	0.049198896	-4.097208609
CAMP	0.632549486	1.662817657	2.437335024	0.016157236	0.049235798	-4.098145738
PHGDH	-0.591318686	3.427439866	-2.435384591	0.016240654	0.049435809	-4.102685088
G0S2	0.79767595	2.389957301	2.432863151	0.016349061	0.049719133	-4.108548465

Immune_limma_res

	logFC	AveExpr	t	P.Value	adj.P.Val	B
SECTM1	2.876106612	2.843133595	10.98780659	3.09E-20	6.01E-16	35.32840235
LILRA5	3.499344636	3.447420747	10.8058952	8.75E-20	6.49E-16	34.31871645
IFI30	2.243877766	2.217785321	10.78249614	1.00E-19	6.49E-16	34.18886269
FFAR2	1.924415359	1.744588345	10.58157551	3.16E-19	1.54E-15	33.07420191
CD300E	3.960842538	3.451381237	10.46986922	5.98E-19	2.33E-15	32.4548593
SLC15A3	2.391095525	2.565575558	10.34829452	1.20E-18	3.79E-15	31.78122112
HSPA6	1.698577735	1.778903607	10.32546408	1.36E-18	3.79E-15	31.65477558
ABI3	2.126102187	3.334619586	10.28194239	1.75E-18	4.25E-15	31.413787
CXCL16	2.289323384	2.879244845	10.19264135	2.91E-18	6.29E-15	30.91955124
CHST15	2.654274754	3.220297497	10.13005823	4.16E-18	8.09E-15	30.57339806
LILRB2	3.119554166	4.319628714	9.935694197	1.26E-17	2.22E-14	29.49964235
MAFB	3.368709057	3.802632868	9.816376294	2.48E-17	3.87E-14	28.84158765
LILRA6	2.565615967	2.622608198	9.808609342	2.59E-17	3.87E-14	28.7987845
GPBAR1	2.009515949	2.655034188	9.732623179	3.98E-17	5.54E-14	28.38025444
LILRB1	2.339831674	3.486813013	9.677519621	5.44E-17	7.06E-14	28.07701111
CD14	3.871728649	4.724960659	9.661432524	5.96E-17	7.25E-14	27.98852536
CLEC7A	3.039213333	3.767697805	9.622600922	7.43E-17	8.51E-14	27.77501988
SGK1	2.059289103	2.824965197	9.602323155	8.33E-17	9.01E-14	27.66357637
MARCO	2.531908266	1.82324443	9.58756791	9.06E-17	9.28E-14	27.58250517
IL10RA	2.092360018	4.86945179	9.532002069	1.24E-16	1.17E-13	27.27736977
SLC11A1	2.874875232	3.668955998	9.524991191	1.29E-16	1.17E-13	27.23888906
TNFRSF1B	2.475204045	6.132103482	9.520051699	1.33E-16	1.17E-13	27.21178019
SLC7A7	2.475429566	3.926749247	9.458476469	1.88E-16	1.59E-13	26.87402745
SLC26A11	1.352759547	1.902615127	9.443496356	2.05E-16	1.65E-13	26.791911
CARD16	1.297662399	4.021903748	9.437106699	2.12E-16	1.65E-13	26.75689122
TNFRSF8	1.422771612	1.516936533	9.424463877	2.28E-16	1.70E-13	26.68761108
LILRB3	2.290558883	3.663464921	9.390400799	2.76E-16	1.99E-13	26.50102828
APOBEC3A	2.619402454	3.09209265	9.339355168	3.68E-16	2.55E-13	26.2216351
OASL	1.484859076	1.806095849	9.333299071	3.81E-16	2.55E-13	26.18850496
GIMAP1	1.478947708	3.270798128	9.327817258	3.93E-16	2.55E-13	26.15851968
ZMYND15	1.132651041	0.843572233	9.299626329	4.61E-16	2.89E-13	26.0043652
C5AR1	3.166549272	4.493231657	9.28187968	5.09E-16	3.09E-13	25.90736474
LRRC25	2.71175368	4.601091751	9.221023325	7.17E-16	4.23E-13	25.57498769

AQP9	2.752587985	2.724124409	9.175399217	9.26E-16	5.30E-13	25.32606985
ZFP36L1	1.873505686	5.058603032	9.062140095	1.75E-15	9.57E-13	24.7091797
HMOX1	2.478381143	3.843200019	9.059951664	1.77E-15	9.57E-13	24.69727495
FCN1	3.295605015	5.620795074	8.990398798	2.61E-15	1.37E-12	24.31922605
CLEC1A	0.726903819	0.519701502	8.982783211	2.73E-15	1.40E-12	24.27786907
FGD2	2.351807474	3.63376478	8.869951966	5.12E-15	2.56E-12	23.66601809
CLEC4A	2.188674627	3.669721727	8.845676739	5.87E-15	2.85E-12	23.53460416
IL4I1	1.240616913	1.333752756	8.809394212	7.18E-15	3.41E-12	23.33834132
KCNH3	1.119748954	1.615784539	8.796503638	7.72E-15	3.57E-12	23.26865706
GIMAP4	2.276011177	4.412642555	8.764076251	9.24E-15	4.18E-12	23.09346523
IER3	1.98294561	2.800883896	8.760244685	9.44E-15	4.18E-12	23.07277488
BCL6	2.042574986	4.502893014	8.749674466	1.00E-14	4.26E-12	23.01570709
LFNG	1.764059588	4.239876057	8.748436968	1.01E-14	4.26E-12	23.009027
POU2F2	1.764134574	5.268235315	8.741887151	1.05E-14	4.33E-12	22.97367443
SLC43A2	1.808586808	3.388876315	8.737614756	1.07E-14	4.34E-12	22.95061762
FTH1	1.186933903	7.943376372	8.592337364	2.40E-14	9.52E-12	22.1682419
CASP5	1.342713612	0.987997287	8.583654543	2.52E-14	9.79E-12	22.12158545
VNN3	1.784675766	1.52826499	8.567869877	2.75E-14	1.05E-11	22.03679874
NUAK2	1.371103618	2.625451277	8.550407975	3.02E-14	1.13E-11	21.94304967
OAS1	1.691168517	3.819790372	8.543715234	3.14E-14	1.15E-11	21.90713097
PSAP	1.48385787	9.466183206	8.530877201	3.37E-14	1.21E-11	21.83825213
SGSH	1.807377047	2.999590106	8.526778988	3.45E-14	1.22E-11	21.81627003
LRP1	2.865027682	4.014806711	8.519163312	3.59E-14	1.25E-11	21.77542822
ZNF703	1.589686476	1.678454142	8.515234639	3.67E-14	1.25E-11	21.75436304
DOK2	1.690590163	4.973141898	8.497056708	4.06E-14	1.36E-11	21.65692808
THEMIS2	1.498021072	6.067769506	8.489270578	4.24E-14	1.39E-11	21.61521077
JAML	2.432953775	4.187177829	8.487332017	4.29E-14	1.39E-11	21.60482574
FGR	2.672443753	6.247745024	8.4626617	4.91E-14	1.56E-11	21.47272019
GIMAP8	2.028924701	3.311034213	8.46035885	4.97E-14	1.56E-11	21.46039407
SNN	1.043657948	4.624059473	8.438183204	5.62E-14	1.74E-11	21.34174425
HK3	3.09018065	4.349380146	8.431625604	5.83E-14	1.77E-11	21.3066743
ADGRE3	1.935894554	1.892010387	8.417559652	6.30E-14	1.89E-11	21.23147492
VNN2	2.410388191	3.797260043	8.385464692	7.51E-14	2.21E-11	21.06001827
LRRK2	2.218027931	2.52631382	8.382976734	7.62E-14	2.21E-11	21.04673476
IL21R	1.03841482	1.63030608	8.353879497	8.94E-14	2.56E-11	20.89146285
SLC9A9	1.604030108	2.325691058	8.336004288	9.86E-14	2.78E-11	20.79615049
IL10	1.504560595	1.073275893	8.27802952	1.36E-13	3.77E-11	20.4874246
MSR1	1.79811697	1.175253943	8.253754816	1.55E-13	4.19E-11	20.35834263
SIGLEC9	2.489080379	3.413608415	8.205702204	2.01E-13	5.37E-11	20.1031494
MAP3K3	0.881007538	5.553838743	8.199110861	2.09E-13	5.49E-11	20.06817924
EHBP1L1	1.071767168	6.17191974	8.189373452	2.20E-13	5.72E-11	20.01653311
CCR5	1.870425212	2.615431007	8.131908491	3.02E-13	7.72E-11	19.71212351
EGR2	2.0468937	2.422666244	8.109490107	3.41E-13	8.58E-11	19.5935442
ARHGEF10L	2.160294115	2.02562405	8.106061606	3.47E-13	8.58E-11	19.57541847
RGS2	2.302341581	5.67184092	8.10540697	3.48E-13	8.58E-11	19.57195782
MEFV	2.537416752	3.011967381	8.096659127	3.65E-13	8.89E-11	19.52572182
RRAS	1.474531401	4.442522319	8.086873307	3.85E-13	9.26E-11	19.47401808

NLRP12	2.328491468	3.048773692	8.074446656	4.12E-13	9.69E-11	19.40838953
COTL1	1.487510141	6.377000959	8.07415702	4.13E-13	9.69E-11	19.40686026
FPR1	2.851314174	3.767089206	8.067273082	4.29E-13	9.84E-11	19.37051831
CEBPB	1.631678004	5.441123409	8.066809989	4.30E-13	9.84E-11	19.36807388
IQSEC1	1.328272023	5.009006179	8.043333109	4.89E-13	1.11E-10	19.24420952
MVP	1.189007662	5.112826719	8.035769025	5.09E-13	1.13E-10	19.20432563
LILRA1	2.29933395	4.152441497	8.034561547	5.12E-13	1.13E-10	19.19795995
TFEB	1.434062855	3.251001517	8.030992768	5.22E-13	1.14E-10	19.17914753
TYMP	1.957831782	5.257839545	8.019156501	5.57E-13	1.20E-10	19.11677307
CYTIP	1.300272867	5.522631923	8.006784634	5.96E-13	1.27E-10	19.05160755
MYD88	1.025308717	6.81040454	7.987241073	6.63E-13	1.40E-10	18.94873296
NFKB2	1.183468738	4.401280987	7.960057522	7.68E-13	1.61E-10	18.80577793
TNFAIP2	2.093921963	5.991487958	7.952279684	8.01E-13	1.65E-10	18.76490444
NCF1	2.289867606	3.496803105	7.951210015	8.06E-13	1.65E-10	18.75928422
STX11	1.87725268	5.098080974	7.948252853	8.19E-13	1.66E-10	18.74374808
CDC42SE1	0.822019692	6.876851489	7.942543926	8.44E-13	1.69E-10	18.71376027
ECE1	1.500159527	3.4831055	7.939238129	8.59E-13	1.71E-10	18.69639884
PRKACA	0.820114198	4.735777721	7.923230654	9.37E-13	1.82E-10	18.61236423
CTSS	1.815842478	7.500201545	7.920419983	9.52E-13	1.83E-10	18.59761481
PLD2	1.505243881	2.516458522	7.917952851	9.64E-13	1.84E-10	18.58466958
GNGT2	1.081425559	1.510502407	7.89295254	1.10E-12	2.09E-10	18.45356667
SLC8A1	1.557240903	1.386920272	7.881319109	1.18E-12	2.20E-10	18.39260755
PPARD	1.190932237	3.356654163	7.869356728	1.25E-12	2.32E-10	18.3299562
ARAP1	1.066073277	6.048627619	7.826444851	1.58E-12	2.90E-10	18.10547608
KLF2	1.5829523	6.947316338	7.818219407	1.65E-12	3.01E-10	18.062495
ARHGAP30	0.839580523	6.895900002	7.789228296	1.93E-12	3.48E-10	17.91112928
CYTH1	0.796631661	5.703419056	7.780848937	2.02E-12	3.61E-10	17.86741594
LY96	1.838628962	3.912163435	7.776624772	2.07E-12	3.66E-10	17.84538555
BCL2A1	1.924189606	4.49759975	7.774499894	2.09E-12	3.67E-10	17.83430519
SLC16A6	1.218840603	1.403485118	7.763422976	2.22E-12	3.86E-10	17.77656075
CX3CR1	2.638922887	4.945756337	7.746507259	2.43E-12	4.19E-10	17.68843392
FKBP7	-0.699273818	2.889541209	-7.706891147	3.01E-12	5.11E-10	17.48230852
EFHD2	1.035070792	7.37431481	7.706133238	3.02E-12	5.11E-10	17.47836871
TLR8	2.795085956	3.367211846	7.673171305	3.60E-12	6.04E-10	17.30715812
ZBP1	0.973985311	1.63652197	7.664656404	3.77E-12	6.27E-10	17.26297289
CD1D	2.499987362	3.613320376	7.660856126	3.85E-12	6.35E-10	17.24325833
TNFSF10	1.60129605	4.758249823	7.658180192	3.90E-12	6.38E-10	17.22937859
RASSF5	0.82948105	5.323003895	7.617721608	4.85E-12	7.81E-10	17.01973961
GPRIN3	1.453654011	2.606896478	7.616703654	4.87E-12	7.81E-10	17.01447023
NLRP1	1.468579423	5.010788772	7.614404047	4.93E-12	7.81E-10	17.0025674
KIAA0513	1.642814103	3.393287331	7.614298387	4.94E-12	7.81E-10	17.00202054
CCR1	2.275214569	4.37543277	7.60591157	5.16E-12	8.10E-10	16.95862152
GPR132	1.312688292	3.868632396	7.593800669	5.51E-12	8.57E-10	16.89598263
SCIMP	2.067884814	3.739953669	7.564764915	6.43E-12	9.91E-10	16.74595685
SLC2A6	1.549211257	3.273328709	7.563605542	6.47E-12	9.91E-10	16.73997087
EPHB2	0.933574543	0.60080451	7.539412199	7.36E-12	1.12E-09	16.61513606
GPR65	1.445434869	2.970233865	7.524643788	7.96E-12	1.20E-09	16.5390065

SH3BP2	1.07073743	5.368334244	7.518552917	8.22E-12	1.23E-09	16.50762509
UNC119	1.002060583	3.949262843	7.505802783	8.80E-12	1.31E-09	16.44196488
NADK	0.980336917	5.62475828	7.496357263	9.25E-12	1.36E-09	16.39334989
TLR7	2.098411501	2.431583542	7.482295896	9.97E-12	1.45E-09	16.3210208
ZFP36	1.364151527	8.546543736	7.482268885	9.97E-12	1.45E-09	16.32088191
DEDD2	0.739759191	5.100145368	7.47809864	1.02E-11	1.47E-09	16.29944093
LGALS3	1.927958109	4.435377141	7.471996372	1.05E-11	1.50E-09	16.26807483
SLC46A2	1.288713571	1.531478475	7.471404206	1.06E-11	1.50E-09	16.26503158
ADAP2	1.933056762	3.31793606	7.46489514	1.09E-11	1.54E-09	16.23158634
ITGAM	2.008338645	5.560710542	7.45506626	1.15E-11	1.61E-09	16.18110426
CEACAM3	1.344525638	1.080493125	7.445410643	1.21E-11	1.69E-09	16.131537
BCL3	1.235457278	4.891348244	7.443686838	1.22E-11	1.69E-09	16.12269043
VDR	1.619527055	2.954289216	7.433151673	1.29E-11	1.77E-09	16.06864114
SYTL3	1.250573039	3.348075545	7.419653643	1.39E-11	1.89E-09	15.99943463
FCGR3A	2.288128579	4.109797777	7.412877139	1.44E-11	1.95E-09	15.96470887
GIMAP7	1.898097109	4.336643265	7.410039178	1.46E-11	1.96E-09	15.9501696
APOA2	1.520232575	2.219255462	7.404944941	1.50E-11	1.99E-09	15.92407655
ADM	1.37748795	1.610964733	7.40459026	1.51E-11	1.99E-09	15.92226011
PXN	1.071235285	5.381144489	7.400192041	1.54E-11	2.03E-09	15.89973818
GBP1	1.776193353	2.531349265	7.389309992	1.63E-11	2.12E-09	15.84403707
CREB5	1.532163176	2.288509032	7.388620918	1.64E-11	2.12E-09	15.84051104
TMCC3	1.01391748	1.22545433	7.379149016	1.72E-11	2.21E-09	15.79205591
RCN3	1.548642159	3.033534293	7.378283654	1.73E-11	2.21E-09	15.78763022
CFP	1.621059789	5.439347941	7.374917121	1.76E-11	2.24E-09	15.77041484
SH3TC1	1.516119549	3.768880312	7.37289196	1.78E-11	2.25E-09	15.76006029
NINJ1	1.480182424	4.521351628	7.369567915	1.81E-11	2.25E-09	15.74306706
ASGR2	2.010363536	2.342679339	7.369524032	1.81E-11	2.25E-09	15.74284274
CRTAM	0.913366019	1.59680086	7.368169605	1.82E-11	2.25E-09	15.7359195
RARG	1.020766485	2.52109688	7.367668668	1.83E-11	2.25E-09	15.73335906
SIGLEC7	1.824946791	2.466187909	7.362719897	1.88E-11	2.30E-09	15.70806805
RNF19B	0.748072913	5.062641531	7.360027056	1.90E-11	2.32E-09	15.69430894
PLEKHO2	0.866933765	5.426521826	7.357101608	1.93E-11	2.34E-09	15.67936359
CD101	1.817341915	2.635624261	7.35111376	2.00E-11	2.40E-09	15.64878056
TLR5	1.900361146	2.382632377	7.327305913	2.26E-11	2.70E-09	15.52727964
C9orf72	1.242885011	4.692087057	7.295365188	2.68E-11	3.18E-09	15.36452136
LIPN	1.636371804	1.935434975	7.292774165	2.71E-11	3.19E-09	15.35133099
CSK	0.744050633	6.565500723	7.29250106	2.72E-11	3.19E-09	15.34994079
PLEC	1.465050097	5.86894994	7.288184032	2.78E-11	3.24E-09	15.32796823
MOB3A	0.837172358	6.726632632	7.27553049	2.97E-11	3.44E-09	15.26359531
FOLR2	1.423976452	1.528494562	7.274180163	2.99E-11	3.45E-09	15.25672841
GBP2	1.4409376	4.547173252	7.267344049	3.10E-11	3.55E-09	15.22197225
C19orf38	1.688806771	4.756748242	7.259616519	3.23E-11	3.68E-09	15.18269997
CAMK2D	1.322534589	2.243508408	7.23453748	3.68E-11	4.17E-09	15.05536274
CD300C	2.039927364	4.224754744	7.231861472	3.74E-11	4.20E-09	15.04178614
SLC6A12	0.95283803	0.789451266	7.229850175	3.78E-11	4.22E-09	15.03158327
TBC1D9	1.514527601	2.592882436	7.222648812	3.92E-11	4.36E-09	14.99506195
PILRA	1.531441381	5.048142853	7.208409424	4.23E-11	4.67E-09	14.92289182

ARHGAP27	0.936267148	5.095147166	7.193704057	4.56E-11	5.02E-09	14.84842183
LSP1	1.859955414	5.8697923	7.182035805	4.85E-11	5.30E-09	14.78937715
GPSM3	0.775630567	7.358656276	7.178291199	4.95E-11	5.35E-09	14.77043684
SULF2	1.906897511	3.940552996	7.178127245	4.95E-11	5.35E-09	14.76960765
SIGLEC1	2.088345287	1.859803923	7.177007275	4.98E-11	5.35E-09	14.76394367
FCGR2B	1.252963019	1.712670385	7.164242591	5.32E-11	5.69E-09	14.6994154
ITGB2	1.655489708	7.508271431	7.160681301	5.42E-11	5.77E-09	14.68142088
KCNQ1	1.657251304	3.175357884	7.155906873	5.56E-11	5.88E-09	14.65730249
UBXN11	1.218851325	5.094976191	7.150886562	5.71E-11	6.00E-09	14.63194929
NPC2	0.847005594	6.122565241	7.145387499	5.87E-11	6.14E-09	14.60418691
HCK	1.805378808	6.00499591	7.14238879	5.97E-11	6.21E-09	14.58905152
HELZ2	1.283957447	3.37990549	7.128295718	6.42E-11	6.65E-09	14.51795544
FCGR1B	1.221519293	1.772438313	7.125848966	6.50E-11	6.66E-09	14.50561823
NAGK	0.766246718	5.247299873	7.12576598	6.51E-11	6.66E-09	14.50519982
PHOSPHO1	1.539246383	2.673896969	7.113923637	6.92E-11	7.03E-09	14.44551303
PIK3R5	1.069174517	4.979591384	7.11356854	6.93E-11	7.03E-09	14.44372396
AL049634.2	0.822321117	0.759371869	7.108077081	7.13E-11	7.19E-09	14.41606126
SLC37A2	1.647172718	2.676983118	7.093618519	7.69E-11	7.71E-09	14.34327115
SAT1	1.400211858	7.790548298	7.092478165	7.74E-11	7.72E-09	14.33753285
RAB24	0.965399105	4.156407807	7.089534341	7.86E-11	7.80E-09	14.32272124
METRNL	1.438578253	3.931491566	7.087386788	7.94E-11	7.85E-09	14.31191767
NCF2	2.033122656	6.16819615	7.086295534	7.99E-11	7.85E-09	14.30642849
CDH23	1.063027189	1.753270731	7.078311392	8.33E-11	8.14E-09	14.26627805
VSIR	1.604920855	7.03775136	7.072798046	8.57E-11	8.34E-09	14.23856401
KLF4	2.287813692	4.945466196	7.066906964	8.84E-11	8.55E-09	14.20896146
ITGAX	1.867448079	5.574569174	7.055371863	9.38E-11	9.04E-09	14.15102858
VIPR1	1.253741526	2.077591009	7.052049442	9.54E-11	9.15E-09	14.13434992
RARA	0.992524744	5.016438459	7.050013059	9.65E-11	9.20E-09	14.12412889
HLA-F	1.292178125	4.464092342	7.049046905	9.69E-11	9.20E-09	14.11928001
CASP1	1.301787775	5.3299624	7.043760757	9.96E-11	9.41E-09	14.09275525
SGTB	0.834983445	3.181079758	7.025458152	1.10E-10	1.03E-08	14.00098338
MPEG1	3.112403257	6.077885154	7.024547061	1.10E-10	1.03E-08	13.99641775
S100A9	3.474694208	9.319288163	7.020718269	1.12E-10	1.04E-08	13.97723385
HLA-E	0.874447677	9.104817459	7.019476455	1.13E-10	1.04E-08	13.9710128
ARRB2	1.023788536	6.792434636	7.019020149	1.13E-10	1.04E-08	13.96872699
MS4A4A	2.183098132	3.116552624	7.001584541	1.24E-10	1.14E-08	13.88143401
PELI1	1.389626679	4.575684199	6.999434286	1.25E-10	1.15E-08	13.87067514
NUCB2	-1.014509054	6.633682321	-6.997461459	1.27E-10	1.15E-08	13.86080531
ZNF385A	1.437762613	5.513079175	6.992449035	1.30E-10	1.18E-08	13.83573421
MBD2	0.604644955	5.096147659	6.991703496	1.30E-10	1.18E-08	13.83200585
ASGR1	0.81952304	1.500019672	6.986359355	1.34E-10	1.20E-08	13.8052855
S100A8	3.384437152	7.835949218	6.981674364	1.37E-10	1.23E-08	13.78186825
KCTD12	2.502112184	4.685683084	6.976480013	1.41E-10	1.25E-08	13.75591312
CTLA4	0.972015934	1.387833039	6.975098709	1.42E-10	1.26E-08	13.74901245
S100A6	1.586884697	7.328031731	6.968597394	1.47E-10	1.29E-08	13.71654153
GIMAP5	0.972171573	1.573096694	6.955456088	1.57E-10	1.38E-08	13.65094794
S100A12	3.344051651	5.358097062	6.945110577	1.66E-10	1.44E-08	13.59934791

TTC26	-0.661961194	2.20658842	-6.944903933	1.66E-10	1.44E-08	13.59831759
FCER1G	1.97445603	7.540687066	6.934247798	1.76E-10	1.52E-08	13.54520468
CD86	2.120068394	3.890872624	6.925624507	1.83E-10	1.57E-08	13.50225061
F5	1.608154144	2.113048935	6.925350377	1.84E-10	1.57E-08	13.50088552
IL1RN	1.97591815	3.106985169	6.924707462	1.84E-10	1.57E-08	13.49768406
IL15	1.230635181	1.293970894	6.919144542	1.90E-10	1.61E-08	13.46998852
ODF3B	1.544304664	4.003443613	6.918953499	1.90E-10	1.61E-08	13.46903757
SIRPB1	2.192499305	3.958171753	6.911742224	1.97E-10	1.66E-08	13.43315082
SIGLEC10	1.348117358	4.240194862	6.910590276	1.98E-10	1.66E-08	13.42741973
IFIT2	1.244847576	2.80014252	6.906599996	2.02E-10	1.69E-08	13.40757088
NAMPT	1.529501876	4.648581306	6.886543569	2.24E-10	1.87E-08	13.30788232
PDK4	1.909754401	2.195121961	6.883866649	2.27E-10	1.88E-08	13.29458683
SH3BP5	1.211130716	2.022147657	6.876907429	2.36E-10	1.94E-08	13.26003332
FCGR2A	1.780565767	4.491287686	6.874946689	2.38E-10	1.96E-08	13.25030082
LILRB4	2.395877638	2.799017395	6.867290035	2.48E-10	2.02E-08	13.21230765
CD4	1.696072617	5.787242216	6.859396281	2.58E-10	2.10E-08	13.17315806
CUEDC1	1.262998419	2.087713464	6.85829716	2.59E-10	2.10E-08	13.16770852
RAB39A	1.209715355	1.329752205	6.856415646	2.62E-10	2.11E-08	13.15838073
GLIPR2	1.301950499	5.75363068	6.856389889	2.62E-10	2.11E-08	13.15825305
PPM1M	1.042964938	4.674538714	6.852253429	2.68E-10	2.14E-08	13.13775027
ATP6V0D1	0.828753872	4.981982177	6.848308024	2.73E-10	2.18E-08	13.11819972
FGL2	2.313108936	5.399648057	6.845953253	2.76E-10	2.19E-08	13.10653364
EXTL2	-0.782923868	3.824774173	-6.826329882	3.06E-10	2.42E-08	13.00938624
TLR1	1.261448299	4.200024446	6.825358633	3.07E-10	2.42E-08	13.0045813
ADAP1	1.148081823	3.353238454	6.822429719	3.12E-10	2.45E-08	12.99009332
STMN1	-0.892723287	6.836649088	-6.82130577	3.13E-10	2.45E-08	12.98453442
VCAN	3.20386564	5.315015266	6.818070968	3.19E-10	2.48E-08	12.96853789
CYTH4	1.558185491	5.469444478	6.817065485	3.20E-10	2.48E-08	12.96356635
HFE	0.826898619	0.972314187	6.81430249	3.25E-10	2.51E-08	12.94990665
SERPINA1	2.582003849	5.451767005	6.810362636	3.32E-10	2.55E-08	12.9304332
ABCC3	1.236932368	1.29277221	6.800924216	3.48E-10	2.67E-08	12.88380318
NPL	1.574172245	3.043714223	6.796782294	3.55E-10	2.71E-08	12.86334965
CD300A	1.448331316	5.193974522	6.791164486	3.66E-10	2.77E-08	12.83561714
MYCL	1.496768006	1.484927119	6.791002455	3.66E-10	2.77E-08	12.83481742
SAMD4A	0.836729666	0.79867859	6.789420482	3.69E-10	2.78E-08	12.82700996
RHBDF2	1.166885496	4.767639266	6.787345818	3.73E-10	2.80E-08	12.8167722
ZNF467	1.450989849	4.617135123	6.780901495	3.85E-10	2.88E-08	12.78498092
CXCL10	1.325744802	1.240527128	6.779352238	3.88E-10	2.90E-08	12.77734018
LGALS2	2.641642822	3.003776548	6.767838033	4.12E-10	3.06E-08	12.72057897
HCLS1	0.692931567	7.022324465	6.757874914	4.33E-10	3.21E-08	12.67150033
RBM47	2.120473342	3.442842442	6.755747826	4.38E-10	3.23E-08	12.6610266
GCSH	-0.601458359	2.239177312	-6.750273131	4.50E-10	3.31E-08	12.6340764
EPSTI1	1.556470705	2.661008052	6.734484818	4.88E-10	3.57E-08	12.55641277
ITGAL	1.336467754	6.810286802	6.72916104	5.02E-10	3.66E-08	12.53024402
PLBD1	2.696656109	3.923940727	6.721436205	5.22E-10	3.79E-08	12.49229031
IRF9	0.7959584	4.424338944	6.718957283	5.28E-10	3.82E-08	12.4801152
TMEM127	0.587363513	5.737906135	6.717955937	5.31E-10	3.83E-08	12.47519773

RTN1	1.149579858	0.898680262	6.711689079	5.48E-10	3.94E-08	12.44442996
CDC42EP2	0.908491412	2.778643968	6.700465301	5.80E-10	4.15E-08	12.38935957
VASP	0.766150847	6.474371156	6.691353082	6.08E-10	4.33E-08	12.3446818
MX1	1.661063456	3.124276432	6.687267564	6.21E-10	4.39E-08	12.32465961
CCDC69	0.854831407	5.761811834	6.682727648	6.35E-10	4.48E-08	12.30241734
1-Mar	1.298988175	3.907991349	6.68036963	6.43E-10	4.51E-08	12.29086761
MCOLN1	0.841642328	4.216811789	6.668695093	6.82E-10	4.76E-08	12.23371352
CRISPLD2	1.626098203	2.270341399	6.664853639	6.95E-10	4.81E-08	12.21491764
MYO1F	1.004132808	6.912238258	6.663124663	7.01E-10	4.84E-08	12.20645961
TM7SF3	-0.970342375	7.259520097	-6.653870659	7.35E-10	5.05E-08	12.16120749
RXRA	1.327613651	4.117097117	6.64765084	7.58E-10	5.18E-08	12.13080947
WARS	0.969441518	5.771233178	6.647606958	7.59E-10	5.18E-08	12.13059505
FAM214B	0.754524464	4.26618798	6.641775987	7.81E-10	5.32E-08	12.10210991
ZBTB7B	1.198335369	5.450325155	6.640252621	7.87E-10	5.34E-08	12.09467002
FCGRT	1.550686159	5.530985589	6.632438873	8.19E-10	5.53E-08	12.05652176
BTG2	1.094442069	6.686992103	6.63044304	8.27E-10	5.57E-08	12.04678117
WAS	0.756188625	7.211238062	6.625294397	8.49E-10	5.70E-08	12.02165993
CD48	1.519319444	5.177797106	6.623111822	8.59E-10	5.72E-08	12.01101356
TBC1D2	1.03400348	4.769329342	6.612065249	9.08E-10	6.01E-08	11.95715551
UNC5A	1.376872188	1.401855363	6.597656769	9.76E-10	6.44E-08	11.88697172
MS4A6A	2.547294846	4.821041588	6.594078259	9.94E-10	6.54E-08	11.86955229
P2RY13	1.933140612	4.020890502	6.589627928	1.02E-09	6.66E-08	11.84789542
RPGRIP1L	-0.587518705	1.628124875	-6.588721463	1.02E-09	6.67E-08	11.84348512
SRC	1.403048677	3.125824526	6.582889557	1.05E-09	6.84E-08	11.81511769
UPP1	1.486284578	3.457215536	6.576432026	1.09E-09	7.05E-08	11.78372139
ZDHHC7	0.686397971	5.775033454	6.553356179	1.22E-09	7.88E-08	11.67165045
MS4A7	1.783310999	4.713307771	6.552893276	1.22E-09	7.88E-08	11.66940427
MSH5	-0.622408329	4.279340664	-6.545241064	1.27E-09	8.16E-08	11.6322842
RNF166	0.82883107	5.704947975	6.54458299	1.28E-09	8.16E-08	11.62909295
CORO7	0.607106673	3.677876191	6.541650713	1.29E-09	8.26E-08	11.61487513
DSCC1	-0.885973182	3.874759759	-6.538851303	1.31E-09	8.34E-08	11.60130448
CD300LB	2.016244355	3.30360258	6.538412075	1.32E-09	8.34E-08	11.5991755
IER5	0.746954093	4.234253417	6.530446093	1.37E-09	8.65E-08	11.56057586
CMKLR1	1.340429956	1.841052602	6.527643862	1.39E-09	8.74E-08	11.547003
KCNJ2	0.911072287	0.694091132	6.523865639	1.41E-09	8.88E-08	11.52870739
S100A4	1.318246823	8.209092696	6.515404573	1.48E-09	9.24E-08	11.48775464
C5AR2	1.168632943	1.765420941	6.499536127	1.60E-09	9.94E-08	11.41102013
JUNB	1.202706931	8.415425677	6.484707405	1.72E-09	1.06E-07	11.33939744
CYP27A1	1.873712956	1.991508029	6.482496982	1.74E-09	1.07E-07	11.32872809
VMP1	0.80083544	6.108875047	6.479652165	1.77E-09	1.08E-07	11.31499928
OGFR	0.702821838	5.214758512	6.47706464	1.79E-09	1.09E-07	11.30251476
TLDC2	1.537093289	3.688747872	6.474479113	1.81E-09	1.11E-07	11.29004236
FCN2	0.634924967	0.575716081	6.471717067	1.84E-09	1.12E-07	11.2767212
IFI35	0.672421441	4.896921101	6.46807418	1.87E-09	1.13E-07	11.25915614
CTSZ	1.131722877	7.337766283	6.460409833	1.94E-09	1.17E-07	11.22221682
FYN	1.041457736	4.568401008	6.45997167	1.95E-09	1.17E-07	11.2201057
LST1	1.576098861	5.94027799	6.457306105	1.97E-09	1.19E-07	11.20726425

MYOF	1.964456702	2.827258831	6.42977704	2.26E-09	1.34E-07	11.07479796
CDKN2D	1.012550157	4.977938426	6.429268295	2.27E-09	1.34E-07	11.07235263
CTSL	1.71475953	3.106139485	6.414964409	2.44E-09	1.44E-07	11.00363974
ZC3HAV1L	-0.955339779	4.575340843	-6.414560497	2.44E-09	1.44E-07	11.00170055
LTB	1.28942547	4.264333184	6.407498961	2.53E-09	1.48E-07	10.96780807
FLOT1	1.099397807	5.537973537	6.398471277	2.65E-09	1.55E-07	10.9245065
MX2	1.132386513	3.61685978	6.378623152	2.92E-09	1.68E-07	10.82941369
MS4A14	1.77462747	3.232899066	6.37799644	2.93E-09	1.68E-07	10.82641356
ITGB7	1.323538637	4.276810181	6.374213971	2.99E-09	1.71E-07	10.80830969
NR4A1	1.673280695	3.42797839	6.36871488	3.07E-09	1.75E-07	10.78199942
ITPKA	-1.156978922	1.855193202	-6.361118684	3.19E-09	1.81E-07	10.74567474
IL32	1.105591662	2.878735507	6.360526299	3.19E-09	1.81E-07	10.74284292
OAS2	1.454336314	4.696756134	6.350242518	3.36E-09	1.90E-07	10.69370414
SLC12A9	0.976767491	4.336475342	6.348098071	3.40E-09	1.92E-07	10.68346252
GZMB	1.486488547	2.868471129	6.34690155	3.42E-09	1.92E-07	10.67774886
CD163	2.628171268	3.301821017	6.345887495	3.44E-09	1.93E-07	10.67290695
DTX4	1.397827723	2.660175077	6.34306953	3.48E-09	1.95E-07	10.65945381
SPI1	0.888402448	7.805695382	6.342528574	3.49E-09	1.95E-07	10.65687161
TCIRG1	1.217853245	6.746062304	6.338891917	3.56E-09	1.97E-07	10.6395153
NLRC4	1.447593296	3.335477271	6.338829939	3.56E-09	1.97E-07	10.63921955
GASK1B	1.938242816	3.242688766	6.337540608	3.58E-09	1.97E-07	10.63306735
LTA	0.725906754	1.141035509	6.334621098	3.63E-09	2.00E-07	10.61913894
ANXA5	1.996341082	5.291382602	6.327404066	3.76E-09	2.06E-07	10.58472214
IL27	0.693863866	1.139291197	6.323598201	3.84E-09	2.09E-07	10.56658078
C1orf162	1.332140165	5.92191534	6.320205448	3.90E-09	2.12E-07	10.55041335
SH3BGRL3	0.940736667	8.963439244	6.297515311	4.36E-09	2.36E-07	10.44240421
NIBAN2	1.641865169	3.846634564	6.288635563	4.56E-09	2.46E-07	10.40019003
SCCPDH	-0.702025035	6.417102158	-6.288227725	4.57E-09	2.46E-07	10.39825192
RGL1	1.014140051	1.362122904	6.284495512	4.65E-09	2.49E-07	10.3805189
CDK6	-1.039815334	7.442171529	-6.28442819	4.65E-09	2.49E-07	10.38019908
ADAM19	1.172253605	2.795049451	6.27769822	4.81E-09	2.57E-07	10.34823679
FGD3	0.69916372	5.893441489	6.276135327	4.85E-09	2.58E-07	10.34081679
RASSF3	0.748233488	5.580033678	6.265390898	5.11E-09	2.72E-07	10.28983262
CYBB	2.320496345	6.754759134	6.258714245	5.28E-09	2.80E-07	10.25817382
2-Mar	0.755022774	4.658895998	6.248291414	5.56E-09	2.94E-07	10.20878716
SH2B3	0.732427132	6.301788747	6.245706444	5.63E-09	2.97E-07	10.19654546
HPSE	1.425111055	2.930659831	6.240910489	5.76E-09	3.03E-07	10.17384021
NACC2	1.349765496	3.554447265	6.217674493	6.46E-09	3.37E-07	10.06396566
REM2	0.604701132	1.368418908	6.209684471	6.72E-09	3.49E-07	10.0262339
OTUD1	0.976235392	4.231977993	6.197012222	7.15E-09	3.70E-07	9.966443838
SMPDL3A	1.481596719	1.446687814	6.181775462	7.70E-09	3.96E-07	9.894640067
MAPK7	0.897840048	3.361176524	6.18130275	7.72E-09	3.96E-07	9.892413903
UCP2	0.811631232	7.66296931	6.172568757	8.06E-09	4.13E-07	9.851298898
IFNGR2	0.835935303	5.898829393	6.171925672	8.08E-09	4.13E-07	9.848272829
ARHGAP45	0.913385815	6.827619553	6.171321047	8.10E-09	4.13E-07	9.845427881
TFE3	0.669589071	5.477672223	6.168679398	8.21E-09	4.15E-07	9.832999874
VPS37C	0.659502269	3.836060615	6.168378943	8.22E-09	4.15E-07	9.831586519

HVCN1	1.126118644	4.424530386	6.168052581	8.24E-09	4.15E-07	9.830051343
BCKDHB	-0.585449996	4.00574761	-6.166768996	8.29E-09	4.17E-07	9.824013893
IFNGR1	0.962548599	6.613125242	6.165844935	8.32E-09	4.18E-07	9.819667914
UNC93B1	1.079605412	5.499997075	6.163591287	8.42E-09	4.21E-07	9.80907017
DNASE1L1	0.665281151	4.082185815	6.160221137	8.56E-09	4.27E-07	9.793225977
CD1A	0.732712233	0.643199317	6.15805671	8.65E-09	4.30E-07	9.783052736
TNFSF8	1.246456671	3.753683616	6.157330175	8.68E-09	4.31E-07	9.779638306
IGF2R	1.535002825	3.903582084	6.153776851	8.83E-09	4.37E-07	9.762942195
HLX	1.139724739	3.726118575	6.152385336	8.89E-09	4.39E-07	9.756405254
RGS19	0.797069509	6.585377363	6.150853451	8.96E-09	4.41E-07	9.749209812
LTA4H	0.887635075	6.13045807	6.149812699	9.00E-09	4.42E-07	9.744321827
SMAP2	0.847975136	7.363702843	6.1484414	9.06E-09	4.44E-07	9.737882079
ARSG	0.771732391	1.722179836	6.140413357	9.42E-09	4.61E-07	9.700197157
FAM89B	0.68536625	4.662041496	6.13637271	9.61E-09	4.68E-07	9.681239749
TECPR1	0.628906361	3.668228676	6.127101616	1.01E-08	4.88E-07	9.637768226
SNX21	0.601370369	2.486741328	6.126447091	1.01E-08	4.88E-07	9.634700547
KCNE1	1.34916249	1.811483873	6.124475554	1.02E-08	4.92E-07	9.625461259
ANKRD34B	0.643948111	0.409009177	6.115267623	1.07E-08	5.13E-07	9.582331095
PEA15	0.736410003	5.611009231	6.108501969	1.10E-08	5.28E-07	9.550663024
RHOC	1.081418466	3.530700423	6.106127565	1.11E-08	5.32E-07	9.539553635
LAPTM5	0.869331273	9.662457066	6.105538153	1.12E-08	5.33E-07	9.53679625
PPP1R9B	0.616154281	6.907087375	6.104526042	1.12E-08	5.34E-07	9.532061734
ARRDC2	0.714094174	5.254869812	6.090973879	1.20E-08	5.67E-07	9.468707697
ARHGAP24	1.014631904	1.541763167	6.084765301	1.24E-08	5.83E-07	9.439709268
PTPRO	1.217297646	1.852962199	6.083425088	1.24E-08	5.85E-07	9.433451646
PTPRE	0.961567196	5.477677298	6.083122371	1.24E-08	5.85E-07	9.432038327
MAST3	0.807658376	4.944478148	6.082687147	1.25E-08	5.85E-07	9.430006431
FAM49A	1.302012219	4.219680262	6.078956158	1.27E-08	5.94E-07	9.412591093
MTSS1	1.426065264	4.09110557	6.078069096	1.28E-08	5.95E-07	9.408451365
RIPOR2	1.028616993	6.114790125	6.0747067	1.30E-08	6.04E-07	9.392762782
FXYD6	1.405687643	1.768818323	6.07308648	1.31E-08	6.07E-07	9.385204699
MYB	-0.747495103	7.576041513	-6.065754076	1.35E-08	6.26E-07	9.351013933
MICAL2	1.17656966	3.008725041	6.06520347	1.36E-08	6.26E-07	9.348447389
IGSF6	1.364872978	4.615148262	6.062284311	1.38E-08	6.32E-07	9.334842413
APOL3	1.165023131	3.656558675	6.061715876	1.38E-08	6.32E-07	9.332193596
FKBP15	0.76062447	4.845502874	6.04067963	1.53E-08	6.97E-07	9.234263865
FTL	0.816034514	11.45654338	6.03394817	1.58E-08	7.18E-07	9.202966559
RENBP	0.962905872	4.268942176	6.033340433	1.58E-08	7.18E-07	9.200141889
FMNL1	0.745750595	6.899504621	6.03270633	1.59E-08	7.19E-07	9.197194841
FAM161A	-0.644775816	2.236113972	-6.029818046	1.61E-08	7.26E-07	9.183773453
S100A11	1.445957652	8.116365333	6.027610692	1.63E-08	7.31E-07	9.173518624
SASH3	0.802167378	7.225464281	6.027411685	1.63E-08	7.31E-07	9.172594187
USP3	0.654429148	5.167163092	6.024887872	1.65E-08	7.38E-07	9.160871923
BEST1	0.859569398	2.276921244	6.023430813	1.66E-08	7.41E-07	9.154105611
NCR3	0.74371314	2.198024648	6.023129577	1.66E-08	7.41E-07	9.15270684
TSPAN14	0.871238378	4.480868472	6.021530765	1.68E-08	7.45E-07	9.145283493
MGAT1	0.597867823	6.207336938	6.016097022	1.72E-08	7.61E-07	9.120062566

KCTD17	0.967056098	2.526598002	6.012704453	1.75E-08	7.72E-07	9.104322216
BDH1	-0.803150317	3.636756221	-6.007147224	1.80E-08	7.91E-07	9.078549223
LCK	1.203102926	3.49076574	5.991221499	1.94E-08	8.52E-07	9.00476309
CTSD	1.201170439	8.014973105	5.979911981	2.05E-08	8.94E-07	8.952430678
GLT1D1	1.508804778	1.684444456	5.978998166	2.06E-08	8.95E-07	8.948204597
FAM110A	0.82542184	3.992871358	5.978793366	2.06E-08	8.95E-07	8.947257515
BIRC3	0.856192836	3.638037633	5.964870478	2.20E-08	9.52E-07	8.882914785
OSBPL11	0.852918745	4.487273237	5.959770945	2.26E-08	9.74E-07	8.85936898
PPFIBP2	0.949885894	1.726621015	5.95818794	2.27E-08	9.77E-07	8.852062143
SLC16A3	1.0451445	4.271781795	5.958120277	2.27E-08	9.77E-07	8.851749853
RASGEF1B	1.147977816	2.921523395	5.956207901	2.30E-08	9.84E-07	8.842924214
PTPN6	0.817774985	7.234023399	5.954991054	2.31E-08	9.88E-07	8.837309276
CTSH	1.187799196	4.45395517	5.953635528	2.32E-08	9.92E-07	8.831055187
CD3E	1.212410805	4.440541852	5.94416366	2.43E-08	1.03E-06	8.787376431
C1QA	2.25153718	3.16539599	5.941097193	2.47E-08	1.05E-06	8.773244037
SIGLEC11	0.987386138	0.919149087	5.940574246	2.47E-08	1.05E-06	8.770834343
AP1S2	1.176070165	5.05034129	5.937317401	2.51E-08	1.06E-06	8.755829786
CCL3	1.182469539	2.613314957	5.936008991	2.53E-08	1.07E-06	8.749803137
TREM1	1.698056571	4.039106566	5.930852454	2.59E-08	1.09E-06	8.726058959
EMP3	0.986533156	7.359197373	5.930646322	2.59E-08	1.09E-06	8.725110031
CD1C	1.879383399	2.358355123	5.923150016	2.69E-08	1.13E-06	8.690613394
PCED1B	1.007313968	2.975299748	5.921492069	2.71E-08	1.13E-06	8.682987146
NFKBIZ	1.002305311	6.31298943	5.916758988	2.77E-08	1.15E-06	8.661222489
LRP10	0.838845327	5.321186168	5.913775154	2.81E-08	1.17E-06	8.647506642
ITK	1.063906404	2.556901192	5.911836714	2.84E-08	1.18E-06	8.638598278
CDA	2.160558257	4.441763504	5.910710879	2.85E-08	1.18E-06	8.633425105
MYO7A	1.240549534	1.324821171	5.907065589	2.90E-08	1.20E-06	8.616678952
PUS7	-0.711850663	3.886679934	-5.893173497	3.10E-08	1.27E-06	8.552913545
MEI1	0.591990728	2.534094588	5.89182899	3.12E-08	1.28E-06	8.546746709
HLA-DOB	0.905619104	3.044809889	5.88529955	3.22E-08	1.31E-06	8.516809569
PRELID1	0.649822323	5.133000233	5.884278963	3.24E-08	1.32E-06	8.512131936
IFIT1	1.145794514	1.941527175	5.874861921	3.39E-08	1.37E-06	8.468992823
NOD2	1.563281253	3.361229903	5.870516982	3.46E-08	1.40E-06	8.449102089
NAGA	1.05029401	5.860961359	5.867738316	3.50E-08	1.41E-06	8.436386012
STAT6	0.596042258	7.244053141	5.856630609	3.69E-08	1.49E-06	8.38558789
ITPRIP	0.780098519	4.467103504	5.855141703	3.72E-08	1.49E-06	8.378782961
AANAT	0.815773751	1.678927891	5.851871353	3.78E-08	1.51E-06	8.363839554
IRF7	1.043177949	5.416582637	5.846532575	3.88E-08	1.55E-06	8.339455027
FCAR	1.952389729	3.726637299	5.842240026	3.95E-08	1.57E-06	8.319858336
ZNF114	-0.758506048	1.615716629	-5.841850187	3.96E-08	1.57E-06	8.31807902
SAMHD1	1.765276849	7.041226851	5.824808119	4.30E-08	1.70E-06	8.240361754
PI4K2A	1.009312868	2.731147188	5.823143872	4.33E-08	1.71E-06	8.232779256
PLPPR2	0.916773453	4.71332495	5.821861628	4.36E-08	1.72E-06	8.226938059
BRI3	0.753634606	4.142097122	5.818942874	4.42E-08	1.74E-06	8.213644589
ITPK1	0.662332728	4.934491374	5.817750729	4.44E-08	1.75E-06	8.208216063
TYROBP	1.331879059	8.420192138	5.805215358	4.71E-08	1.85E-06	8.151174162
ZDHHC9	-0.669671678	4.104839287	-5.804613758	4.73E-08	1.85E-06	8.148438384

PPP1R18	0.78213124	6.500101841	5.803102454	4.76E-08	1.86E-06	8.141566444
CAPN2	1.152379542	5.179673564	5.797097267	4.90E-08	1.91E-06	8.114270925
ZNF138	-0.672644875	4.311833695	-5.796741126	4.91E-08	1.91E-06	8.112652667
RNPEPL1	0.75623627	5.531318233	5.794666323	4.95E-08	1.92E-06	8.103226151
CKAP4	1.551550156	3.842491992	5.78975053	5.07E-08	1.96E-06	8.080899889
NBPF14	0.587303463	2.0994637	5.773355524	5.48E-08	2.11E-06	8.006517508
RELT	0.924745634	5.080728011	5.770453835	5.55E-08	2.13E-06	7.993365627
BLOC1S1	0.640091588	4.883621373	5.769372298	5.58E-08	2.13E-06	7.988464556
TIAM1	1.127476748	1.645129149	5.765631792	5.68E-08	2.16E-06	7.971518274
TRAF1	0.783646132	3.385148349	5.764590738	5.71E-08	2.17E-06	7.966802936
RCBTB2	0.954019514	4.419327273	5.761237369	5.80E-08	2.20E-06	7.951617611
MAML2	1.077239441	3.097252776	5.757262195	5.91E-08	2.24E-06	7.933623188
BIN2	0.733384649	7.2474826	5.749217934	6.14E-08	2.32E-06	7.897231415
DESI1	0.624017048	4.496755104	5.745631191	6.24E-08	2.34E-06	7.881014779
STAR	-1.087873171	3.802772077	-5.739192714	6.44E-08	2.40E-06	7.851919538
S1PR3	1.263994732	1.708505766	5.738312154	6.46E-08	2.40E-06	7.847941804
ARHGEF1	0.712613535	6.747356911	5.738309673	6.46E-08	2.40E-06	7.847930597
CD83	0.946049547	4.630808514	5.734712317	6.57E-08	2.44E-06	7.831684061
IRAK1BP1	-0.665625927	2.713129458	-5.732630093	6.64E-08	2.46E-06	7.822282958
PARP9	0.779843995	4.544839457	5.728336395	6.77E-08	2.50E-06	7.80290352
CPPED1	0.901411164	5.054380153	5.717662229	7.12E-08	2.62E-06	7.754763035
ADA2	1.378117948	6.704439663	5.707639782	7.46E-08	2.73E-06	7.709609881
CHST2	1.110320167	3.517207301	5.706971909	7.49E-08	2.73E-06	7.706602638
CEMIP2	0.831540299	3.526161179	5.701675185	7.68E-08	2.80E-06	7.682760318
SERTAD3	0.603154658	3.757423982	5.700538964	7.72E-08	2.81E-06	7.677647509
NEDD9	0.851106416	3.128009152	5.694591726	7.93E-08	2.88E-06	7.650895731
TGFB1	0.79532929	7.727054924	5.688724171	8.16E-08	2.94E-06	7.624518552
GPCPD1	0.689178908	6.065804439	5.680735902	8.47E-08	3.05E-06	7.588633709
PMM1	0.75840469	2.032967349	5.679335646	8.52E-08	3.06E-06	7.582346566
CRIP3	0.813120236	1.167778945	5.676360466	8.64E-08	3.09E-06	7.568991068
SLC6A6	0.709938157	5.45361585	5.675560475	8.67E-08	3.10E-06	7.565400639
GAA	1.135066025	5.339856175	5.672034507	8.82E-08	3.14E-06	7.549579366
KDM5B	-0.831841366	5.705007534	-5.663032239	9.20E-08	3.27E-06	7.509212016
PTPRC	0.760156245	7.534960082	5.661319685	9.27E-08	3.29E-06	7.501537014
HTR7	1.304059526	1.453878532	5.657831108	9.42E-08	3.34E-06	7.485906835
CSF2RA	1.225943405	5.141437202	5.656414975	9.48E-08	3.35E-06	7.479563643
ADGRD1	0.635159636	0.602957029	5.656289804	9.49E-08	3.35E-06	7.479003021
SEPTIN9	0.700945015	5.643428609	5.655770473	9.51E-08	3.35E-06	7.476677082
MNS1	-0.673143975	2.646466417	-5.655305722	9.53E-08	3.35E-06	7.474595702
KCNMB1	1.090878432	2.092498651	5.65401741	9.59E-08	3.37E-06	7.468826546
PAICS	-0.662092965	5.829185382	-5.647885077	9.87E-08	3.45E-06	7.441376243
MAP3K14	0.710433304	3.988702629	5.644423264	1.00E-07	3.50E-06	7.425887892
NRGN	1.729340711	5.738642237	5.644364526	1.00E-07	3.50E-06	7.425625146
DHRS9	1.681945147	2.857617515	5.642456808	1.01E-07	3.52E-06	7.41709238
CEP85L	0.959562672	2.765529398	5.638716326	1.03E-07	3.58E-06	7.400367093
MAD2L1	-0.658378623	3.759244327	-5.636847206	1.04E-07	3.60E-06	7.392011948
TYMS	-0.782063845	6.430262987	-5.631351695	1.07E-07	3.69E-06	7.367456066

PCLAF	-0.841770054	5.272122357	-5.630691787	1.07E-07	3.70E-06	7.364508326
POR	0.717365594	3.976913168	5.626748757	1.09E-07	3.76E-06	7.346899544
SLC35E4	0.676338151	1.130052147	5.622720434	1.11E-07	3.82E-06	7.32891748
CCND3	0.729374277	6.391962311	5.622625773	1.11E-07	3.82E-06	7.328495014
CLEC17A	0.80376499	1.923756282	5.617440164	1.14E-07	3.90E-06	7.305358528
DPYD	1.212059165	4.926667897	5.614775121	1.15E-07	3.94E-06	7.29347296
SHKBP1	0.697694913	6.58080839	5.612748901	1.16E-07	3.97E-06	7.28443868
TICAM1	0.705861314	4.707214636	5.611240164	1.17E-07	3.99E-06	7.277712967
CCDC138	-0.596775311	2.993252468	-5.607030672	1.19E-07	4.06E-06	7.258953454
SIDT2	0.805073078	4.685085183	5.602973363	1.22E-07	4.13E-06	7.240880144
LYRM7	-0.648621511	4.561675605	-5.602216063	1.22E-07	4.14E-06	7.237507617
CPED1	1.072843882	1.204859892	5.59725663	1.25E-07	4.22E-06	7.215428276
CENPK	-0.701657114	4.203783768	-5.593978571	1.27E-07	4.28E-06	7.200840852
SH3BP1	0.8604688	5.49889499	5.591433124	1.28E-07	4.32E-06	7.189517121
CTSB	1.012295719	6.799956979	5.58922137	1.30E-07	4.35E-06	7.179680381
MRVI1	0.820544149	1.621305751	5.587777874	1.30E-07	4.37E-06	7.17326172
ACP2	0.658903162	4.282247461	5.587185687	1.31E-07	4.38E-06	7.17062879
GZMH	1.327736541	3.070309772	5.584391509	1.33E-07	4.42E-06	7.158207814
LIMD2	0.708393606	6.292400726	5.582387917	1.34E-07	4.45E-06	7.149303546
PECAM1	1.237520345	6.525270106	5.579161337	1.36E-07	4.50E-06	7.134968185
CBX7	0.600609367	3.218508843	5.574801565	1.39E-07	4.58E-06	7.115606119
CAP1	0.653312095	8.214306905	5.570630633	1.41E-07	4.66E-06	7.097091277
RASGRP4	1.192189283	5.714455948	5.566374738	1.44E-07	4.74E-06	7.078207922
ADGRE1	1.615237154	4.210490769	5.562925788	1.46E-07	4.80E-06	7.062911383
FCGR1A	1.256557763	3.174202038	5.555538265	1.51E-07	4.95E-06	7.030166105
TRIM8	0.714913672	6.258041423	5.551800564	1.54E-07	5.02E-06	7.013608762
ZNF660	-0.780387405	3.738619893	-5.548245104	1.57E-07	5.10E-06	6.997864993
SNTB2	0.821380053	2.000947153	5.544965357	1.59E-07	5.15E-06	6.98334753
TMEM63B	0.790058678	3.68528116	5.537873285	1.64E-07	5.31E-06	6.951973053
SH2D3C	1.006246594	5.395263747	5.536884072	1.65E-07	5.33E-06	6.947598835
POLR3G	-0.640011737	2.814148392	-5.53303185	1.68E-07	5.40E-06	6.930569163
TAP1	0.702071304	5.390794118	5.532890121	1.68E-07	5.40E-06	6.929942756
RELB	0.744721775	3.841988545	5.530185572	1.70E-07	5.46E-06	6.91799115
KMO	0.687979792	1.929862187	5.52851625	1.72E-07	5.49E-06	6.910616068
ACPP	1.274169945	3.363903568	5.525494884	1.74E-07	5.56E-06	6.897271097
DUSP6	1.427391307	6.221837491	5.524250615	1.75E-07	5.58E-06	6.89177662
CD1E	1.30159206	1.004033052	5.522977342	1.76E-07	5.61E-06	6.886154847
RIN2	1.241432166	2.534822554	5.519563559	1.79E-07	5.69E-06	6.871086174
MAP2K1	0.641614972	5.353249431	5.517204311	1.81E-07	5.73E-06	6.860675613
FPR2	1.597254687	2.345868024	5.51536166	1.82E-07	5.76E-06	6.852546515
CMBL	-1.017810447	3.843238225	-5.514157858	1.83E-07	5.78E-06	6.847236678
NFAM1	1.86577934	4.829114395	5.51145431	1.86E-07	5.84E-06	6.835314214
RIPOR1	0.669849014	5.44935332	5.509018613	1.88E-07	5.88E-06	6.824576022
NOTCH2	0.981771533	5.69612951	5.505659357	1.91E-07	5.96E-06	6.809770923
SIRPD	1.277582594	1.716863208	5.505606135	1.91E-07	5.96E-06	6.809536401
KCNC3	1.137026577	1.472291828	5.503202212	1.93E-07	6.01E-06	6.798945157
BATF2	0.730942703	0.986689177	5.495011778	2.00E-07	6.22E-06	6.762880895

PTAFR	1.629905838	4.962578672	5.487348904	2.07E-07	6.44E-06	6.729169448
CDC42EP3	1.266094718	4.271784698	5.484376854	2.10E-07	6.51E-06	6.716102224
G6PD	0.767271423	6.157802109	5.472765975	2.22E-07	6.82E-06	6.665094383
PSTPIP1	1.048457459	4.858259303	5.471970507	2.22E-07	6.83E-06	6.661602242
GPR155	0.805226735	2.495819983	5.469975275	2.25E-07	6.88E-06	6.652844465
PDLIM2	0.704406547	3.143425033	5.468253442	2.26E-07	6.92E-06	6.645288313
ARHGAP31	1.074362259	2.588767677	5.467142721	2.27E-07	6.94E-06	6.640414763
TCP11L2	0.712044801	3.511589773	5.458182585	2.37E-07	7.22E-06	6.601122425
CCR2	1.999539292	3.989666273	5.449252567	2.47E-07	7.49E-06	6.56200179
AHI1	-0.594409096	3.490189412	-5.44048107	2.57E-07	7.79E-06	6.523614217
SIRPG	0.943212202	2.172632281	5.439905566	2.58E-07	7.80E-06	6.52109692
ISG20	0.91782374	2.95057698	5.438358446	2.59E-07	7.84E-06	6.514330525
TMEM38A	0.846905491	1.364118067	5.433263804	2.66E-07	8.01E-06	6.49205733
SLC22A18	0.850893451	2.727337272	5.432315228	2.67E-07	8.03E-06	6.487911691
DEPP1	1.029125268	3.176148014	5.431505884	2.68E-07	8.05E-06	6.48437491
S1PR5	0.907017634	1.684389511	5.43115976	2.68E-07	8.05E-06	6.482862469
BLVRA	1.323098023	4.257384028	5.430929791	2.68E-07	8.05E-06	6.481857618
JAZF1	1.010918418	3.56795389	5.425494248	2.75E-07	8.22E-06	6.458114672
LILRA2	1.321072297	4.77350802	5.419993402	2.82E-07	8.42E-06	6.434101529
AKAP13	0.632989989	5.7125159	5.419352252	2.83E-07	8.43E-06	6.431303665
ST3GAL5	0.911215032	3.468989217	5.414888963	2.89E-07	8.58E-06	6.4118324
ICAM1	1.026843295	4.030184653	5.414816764	2.89E-07	8.58E-06	6.41151751
STX16-NPEI	0.639335952	2.386572595	5.411477872	2.93E-07	8.70E-06	6.396958123
CENPH	-0.612399247	4.1948333	-5.40753047	2.99E-07	8.84E-06	6.379752522
CD36	1.809480275	4.978541522	5.403879939	3.04E-07	8.97E-06	6.363847869
CD5	1.062485205	3.298699325	5.403700951	3.04E-07	8.97E-06	6.363068227
SLA	1.056019705	5.03414878	5.401308783	3.07E-07	9.06E-06	6.352649863
ARHGAP9	0.641301643	6.252115692	5.398986988	3.10E-07	9.14E-06	6.342540747
TAGLN	0.780297833	3.701632326	5.397642063	3.12E-07	9.18E-06	6.33668617
WDR31	-0.649223878	2.405144535	-5.393062679	3.19E-07	9.36E-06	6.316758553
CASP6	-0.631890595	5.213041028	-5.391876592	3.21E-07	9.40E-06	6.311598908
GRINA	0.733706868	6.891800304	5.390892195	3.22E-07	9.42E-06	6.307317184
CYP1B1	1.578682075	2.426508509	5.384593675	3.31E-07	9.65E-06	6.279932769
ARNTL	0.897300282	3.075388581	5.379467707	3.39E-07	9.85E-06	6.257661125
EXT1	0.800982565	1.35437553	5.375781691	3.45E-07	1.00E-05	6.241654096
HECA	0.589808821	5.379087477	5.374318394	3.47E-07	1.01E-05	6.235301439
KLRD1	0.838944909	1.783325612	5.372413078	3.50E-07	1.01E-05	6.227031457
IER5L	0.760455923	2.07073051	5.371668917	3.51E-07	1.01E-05	6.223801943
ID2	1.318694531	5.499037713	5.367956483	3.57E-07	1.03E-05	6.20769489
UBE2D1	0.781712809	4.663274446	5.367839386	3.58E-07	1.03E-05	6.207186959
PIK3IP1	0.880333417	4.806297911	5.364932463	3.62E-07	1.04E-05	6.194579814
RNF149	0.618695887	5.946749143	5.362368266	3.67E-07	1.05E-05	6.183462616
APOBR	1.026682085	5.659912954	5.357308418	3.75E-07	1.07E-05	6.161535211
PLEKHA5	-0.827036492	3.397721068	-5.354260104	3.80E-07	1.09E-05	6.148331294
CRIP1	1.042814159	2.768382449	5.347881892	3.91E-07	1.11E-05	6.120719089
TGFBI	1.716079633	2.420146301	5.346255162	3.94E-07	1.12E-05	6.11368006
SLFN5	1.005770445	2.91429314	5.345645321	3.95E-07	1.12E-05	6.111041563

ETS1	1.022418721	4.65559004	5.343916647	3.98E-07	1.13E-05	6.103563427
TNNI2	1.344724482	2.286635468	5.343557444	3.99E-07	1.13E-05	6.10200973
STK10	0.60047638	6.227385701	5.341025491	4.04E-07	1.14E-05	6.091059882
CHST7	0.744178832	2.090390573	5.339811875	4.06E-07	1.14E-05	6.085812559
PFKFB3	0.739649091	4.720387861	5.339788062	4.06E-07	1.14E-05	6.085709608
MAN1C1	0.721735302	1.702334219	5.339704865	4.06E-07	1.14E-05	6.085349915
SOD2	0.802648984	4.396461445	5.338372843	4.09E-07	1.15E-05	6.079591584
CD27	0.823291134	3.435622954	5.33811754	4.09E-07	1.15E-05	6.078488009
ARHGAP26	0.838064612	4.232097788	5.335419502	4.14E-07	1.16E-05	6.066827519
RUFY4	0.892319504	0.969611965	5.326730418	4.31E-07	1.20E-05	6.029300005
DMPK	0.848828532	1.748038919	5.321499705	4.41E-07	1.23E-05	6.006727605
TNFSF12	1.225511857	3.125894119	5.304196212	4.77E-07	1.31E-05	5.932157052
GRK6	0.654755457	6.016918683	5.303778895	4.77E-07	1.31E-05	5.930360501
ICOS	0.813569855	1.592425246	5.30261879	4.80E-07	1.32E-05	5.925366716
CD40	1.009419733	2.520841476	5.30258535	4.80E-07	1.32E-05	5.925222782
ISG15	0.987970648	4.278140893	5.302298835	4.81E-07	1.32E-05	5.923989565
WRN	-0.607264612	4.645307106	-5.297528749	4.91E-07	1.34E-05	5.903464356
AIF1	0.869219627	7.835952418	5.295310738	4.96E-07	1.35E-05	5.893924478
MEGF9	1.172730677	5.259485285	5.294436747	4.98E-07	1.36E-05	5.890166057
IL4R	0.841586877	4.892206994	5.291518402	5.04E-07	1.37E-05	5.877619163
ZNF366	0.831004641	1.062885044	5.287396864	5.14E-07	1.40E-05	5.859906872
SERTAD1	0.62597282	2.922549773	5.287136092	5.15E-07	1.40E-05	5.858786501
RHOG	0.674530973	7.922617139	5.28094593	5.29E-07	1.43E-05	5.832201673
GMIP	0.648738288	6.658274326	5.27966688	5.32E-07	1.44E-05	5.826711028
CD200R1	1.052606059	2.099388365	5.275841474	5.41E-07	1.46E-05	5.810294569
PSMB9	0.685764941	4.691135002	5.273878731	5.46E-07	1.47E-05	5.801874544
SELPLG	0.933945625	7.445011457	5.273767785	5.46E-07	1.47E-05	5.801398652
HLA-DMB	1.473686348	4.793624578	5.271377984	5.52E-07	1.48E-05	5.791149423
ADCY7	1.067132529	5.104499544	5.266481545	5.64E-07	1.52E-05	5.770159155
KDM7A	0.991422255	3.878429953	5.264712351	5.69E-07	1.53E-05	5.762577967
TMEM120A	0.68442347	3.988183242	5.264141448	5.70E-07	1.53E-05	5.760131931
PCDH12	0.67495398	1.872310533	5.263288452	5.73E-07	1.53E-05	5.756477589
HLA-B	0.756581846	9.869247975	5.26322095	5.73E-07	1.53E-05	5.756188416
CALHM2	0.917225448	4.511265773	5.262913427	5.74E-07	1.53E-05	5.754871051
PDE4A	1.157252585	2.198651345	5.253968642	5.97E-07	1.58E-05	5.716575032
RGS14	0.806245898	5.29516835	5.249454181	6.09E-07	1.62E-05	5.697262766
FGD6	0.947300969	1.984201415	5.247745637	6.14E-07	1.62E-05	5.689956621
CPAMD8	0.717582016	0.830138972	5.245338723	6.20E-07	1.63E-05	5.679666664
CAMK1	1.053798398	2.334759533	5.245125349	6.21E-07	1.63E-05	5.678754602
DEF8	0.672425061	4.13572961	5.241086197	6.32E-07	1.66E-05	5.661493824
IPCEF1	0.968864297	2.515343029	5.240398825	6.34E-07	1.66E-05	5.658557281
C3	0.666500196	0.843849365	5.234551482	6.51E-07	1.70E-05	5.63358669
CCR7	1.13769628	3.639490898	5.233867555	6.53E-07	1.70E-05	5.630667207
SIT1	0.941677873	2.751298247	5.233474159	6.54E-07	1.70E-05	5.628988026
ALOX5	1.62206701	5.458798386	5.23164332	6.60E-07	1.71E-05	5.621174304
LYN	0.760332374	6.598695186	5.231496708	6.60E-07	1.71E-05	5.62054866
PTPRJ	1.272201406	4.429822461	5.230331093	6.63E-07	1.72E-05	5.615575011

RIN1	0.997057763	2.352769366	5.227572846	6.72E-07	1.74E-05	5.603808486
CENPU	-0.69221735	5.376229277	-5.227134626	6.73E-07	1.74E-05	5.60193943
PID1	1.023616577	1.097646982	5.222290093	6.88E-07	1.77E-05	5.581283717
FGD4	1.355226103	3.06556247	5.220220293	6.94E-07	1.79E-05	5.572462442
IFI44L	1.575913473	2.290081963	5.219885585	6.95E-07	1.79E-05	5.571036164
CD37	0.671821125	7.306479774	5.215638372	7.08E-07	1.81E-05	5.552942794
MCEMP1	1.465205305	3.912467619	5.215508709	7.09E-07	1.81E-05	5.552390572
PARP12	0.698933046	4.451106803	5.21249015	7.18E-07	1.83E-05	5.539537317
HLA-C	0.680476743	9.103754114	5.21004715	7.26E-07	1.85E-05	5.529138363
CCDC88B	0.987324995	5.217910386	5.207067346	7.36E-07	1.87E-05	5.51645869
SLAMF7	1.133260373	3.272258668	5.202655038	7.51E-07	1.91E-05	5.497692031
ARPC1B	0.743159902	7.015667008	5.201123311	7.56E-07	1.91E-05	5.49117962
SHTN1	1.386125509	1.810420318	5.200572975	7.58E-07	1.92E-05	5.488840067
EPB41L3	1.363908977	1.288164705	5.191459201	7.89E-07	1.99E-05	5.45011954
PLXNB2	0.934023176	6.963815539	5.185520483	8.10E-07	2.04E-05	5.424912155
CLEC4E	1.68768167	2.27231661	5.183356972	8.18E-07	2.05E-05	5.415733608
S100A10	1.706163056	6.32798947	5.178721843	8.35E-07	2.09E-05	5.396077752
EPDR1	-0.841870208	4.904265331	-5.174167209	8.52E-07	2.13E-05	5.376774372
P2RX7	0.998881324	4.362406599	5.172060462	8.60E-07	2.14E-05	5.367849324
BCL2L11	0.858144805	3.444237968	5.169936982	8.68E-07	2.16E-05	5.358855784
FCMR	1.043281622	4.768723256	5.169818711	8.68E-07	2.16E-05	5.358354942
ALDH18A1	-0.58741943	5.848823962	-5.168952219	8.72E-07	2.16E-05	5.354685846
SIGLEC14	1.297452899	4.806286001	5.168792319	8.72E-07	2.16E-05	5.354008804
PTTG2	0.665182204	1.654831213	5.16784318	8.76E-07	2.17E-05	5.349990289
ZCWPW1	0.637819818	3.577620512	5.16591995	8.83E-07	2.18E-05	5.341849083
PFKFB4	0.943655153	3.267448177	5.162229028	8.98E-07	2.21E-05	5.326230604
CORO1A	0.794989727	7.907143827	5.160290334	9.06E-07	2.23E-05	5.318029753
RHOB	1.329224457	6.336828541	5.159879858	9.07E-07	2.23E-05	5.316293657
WIPI1	0.813574087	3.212956555	5.158966054	9.11E-07	2.24E-05	5.312429082
HLA-DRB1	1.60061954	8.392535262	5.151998211	9.40E-07	2.30E-05	5.282975989
TOM1	0.700147397	4.725915838	5.150905195	9.44E-07	2.31E-05	5.278358164
ARHGEF3	0.743586842	4.526246027	5.145944102	9.65E-07	2.35E-05	5.257406346
SMCO4	1.066571264	4.116323225	5.145816949	9.66E-07	2.35E-05	5.256869527
GZMA	1.104915588	3.88794326	5.141797644	9.83E-07	2.39E-05	5.239905038
NPEPL1	0.648593703	3.386423924	5.138967852	9.95E-07	2.42E-05	5.227966389
SPC25	-0.68420453	3.450766308	-5.138391414	9.98E-07	2.42E-05	5.225534971
ARAP3	1.244527297	2.995085263	5.136722293	1.01E-06	2.44E-05	5.218495627
CR1	1.507458679	2.757106434	5.130251946	1.03E-06	2.50E-05	5.191221753
UNG	-0.623111729	5.459445432	-5.125452861	1.06E-06	2.55E-05	5.171007146
RNF157	0.728055555	1.53649929	5.125419122	1.06E-06	2.55E-05	5.170865077
SERPINB9	1.298984697	4.441255004	5.124802297	1.06E-06	2.55E-05	5.168267808
GPR35	0.851641677	2.561719693	5.123162386	1.07E-06	2.57E-05	5.161363625
IFNK	0.819541636	1.430294495	5.122483141	1.07E-06	2.57E-05	5.158504364
TMSB4X	0.614765135	9.891855347	5.120957836	1.08E-06	2.58E-05	5.152084543
LRRC34	-0.682172812	2.400117837	-5.12039915	1.08E-06	2.58E-05	5.149733417
CEP41	-0.587044	3.105974717	-5.120312911	1.08E-06	2.58E-05	5.149370513
CBLN3	0.597948643	1.333940439	5.115977188	1.10E-06	2.63E-05	5.131130349

IFIT3	0.963418157	3.798019871	5.114278547	1.11E-06	2.64E-05	5.12398702
RIN3	0.656658459	5.958027371	5.113840828	1.11E-06	2.64E-05	5.122146524
PTGS2	1.397374262	3.796980922	5.112934819	1.12E-06	2.64E-05	5.118337318
FOXO1	1.15016499	3.636399279	5.112929576	1.12E-06	2.64E-05	5.118315275
FOLR3	1.226371682	1.401628761	5.112745003	1.12E-06	2.64E-05	5.117539314
IL24	0.887208711	2.719323128	5.106411491	1.15E-06	2.71E-05	5.090923864
TUBA4A	1.210963222	4.333136835	5.104675958	1.16E-06	2.73E-05	5.083634383
SLAMF1	0.786426574	1.968709657	5.086200977	1.26E-06	2.94E-05	5.00613807
RAB29	0.763206987	4.251233131	5.079448673	1.29E-06	3.03E-05	4.977860691
FERMT3	0.661486983	7.569900677	5.078902254	1.30E-06	3.03E-05	4.975573474
AGTRAP	0.950344811	5.675226034	5.076545508	1.31E-06	3.06E-05	4.965710409
QPCT	1.410815265	2.227826729	5.069604887	1.35E-06	3.15E-05	4.936681251
CLCN5	0.909590245	2.431983634	5.066863157	1.37E-06	3.18E-05	4.925221206
IL12RB1	0.944842116	3.551821413	5.064159421	1.38E-06	3.21E-05	4.913923988
SH2D1B	0.762772996	1.413644902	5.061976696	1.40E-06	3.24E-05	4.904806661
IRF4	1.016538856	2.709615396	5.061497233	1.40E-06	3.24E-05	4.902804275
TNFRSF14	0.92036925	4.947337915	5.055549118	1.44E-06	3.32E-05	4.877973557
G0S2	1.632497132	2.389957301	5.055067919	1.44E-06	3.32E-05	4.875965611
ZNF273	-0.716161981	3.143555416	-5.052726114	1.45E-06	3.35E-05	4.866195554
RAB11FIP4	0.677779755	1.809958459	5.051662114	1.46E-06	3.36E-05	4.861757519
GABBR1	1.512947734	3.468308797	5.050626104	1.47E-06	3.37E-05	4.857436825
EPS8	1.278275773	2.167072984	5.048271794	1.48E-06	3.40E-05	4.847620335
SLC37A3	0.757379505	1.643015083	5.044010569	1.51E-06	3.46E-05	4.829860541
OGFRL1	0.924806677	4.912040448	5.043192402	1.52E-06	3.47E-05	4.82645175
CST3	1.56767375	6.849885462	5.039181211	1.54E-06	3.52E-05	4.809744951
GNB2	0.597689322	6.805626854	5.03807607	1.55E-06	3.53E-05	4.805143543
ALDH5A1	-0.814137327	4.385145854	-5.037679073	1.55E-06	3.53E-05	4.803490753
CPVL	1.6546663	4.473917581	5.035470084	1.57E-06	3.56E-05	4.794295809
BICDL1	0.668174958	1.671430122	5.025065473	1.64E-06	3.70E-05	4.751022614
CD68	0.778147055	1.972828693	5.020324102	1.67E-06	3.77E-05	4.731322856
RNASE6	1.754141012	5.996590633	5.019230673	1.68E-06	3.78E-05	4.726781567
CDCA7L	-0.751586672	5.333160083	-5.018841271	1.69E-06	3.78E-05	4.72516444
OR52K2	1.13730695	1.435587744	5.016332164	1.70E-06	3.82E-05	4.714746513
MTHFD1L	-0.650294572	4.529466333	-5.015990695	1.71E-06	3.82E-05	4.713328986
CSF1R	1.307222942	6.339339396	5.011474145	1.74E-06	3.88E-05	4.694585686
BBC3	0.611595992	2.393037945	5.006573035	1.78E-06	3.95E-05	4.674259268
ARHGEF11	1.399096566	3.607491639	5.005873372	1.78E-06	3.96E-05	4.671358631
NLRP3	0.988042109	4.987100716	5.002117359	1.81E-06	4.01E-05	4.655791742
TP53BP1	-0.586241213	4.420865244	-5.001749306	1.82E-06	4.01E-05	4.654266757
DGKA	0.610248629	4.755215203	5.00058135	1.82E-06	4.03E-05	4.649427967
METAP1D	-0.599988067	3.688895941	-4.999080357	1.84E-06	4.04E-05	4.643210529
SNX25	0.838810126	1.524095317	4.994742583	1.87E-06	4.12E-05	4.625249562
PAK1	0.86279425	5.556851513	4.993865287	1.88E-06	4.13E-05	4.621618304
TEDC1	0.733010237	2.788610537	4.992920948	1.89E-06	4.14E-05	4.617710022
ASPHD2	0.609786596	2.319940704	4.992241098	1.89E-06	4.14E-05	4.614896673
KIF1C	0.803203197	3.084379362	4.987252941	1.93E-06	4.21E-05	4.594262585
ZNF85	-0.677402475	3.443608212	-4.986765568	1.94E-06	4.21E-05	4.592247251

FCRL6	0.971356592	2.398626527	4.984822546	1.95E-06	4.24E-05	4.584213991
BLVRB	1.089169576	5.750857815	4.984430711	1.96E-06	4.24E-05	4.582594237
CD247	0.973948582	2.978758849	4.984204759	1.96E-06	4.24E-05	4.581660243
PREX1	0.694718541	6.625880698	4.977589496	2.02E-06	4.34E-05	4.554328028
H2AFJ	1.059573277	3.124568811	4.976382874	2.03E-06	4.35E-05	4.549345271
MND1	-0.686193621	3.446220124	-4.975205304	2.04E-06	4.37E-05	4.544483267
ANKRD27	-0.685781191	5.406489645	-4.974538061	2.04E-06	4.38E-05	4.541728666
CDKN2B	0.881094429	1.153040081	4.974201507	2.05E-06	4.38E-05	4.540339356
KCNQ5	-1.077043688	3.928254486	-4.971372901	2.07E-06	4.42E-05	4.528665227
NFKBIA	0.70218856	7.066502596	4.969827915	2.09E-06	4.44E-05	4.522290697
IFITM10	0.73465063	1.337152899	4.969526009	2.09E-06	4.45E-05	4.521045206
TRPV4	0.636065539	0.482502313	4.968280688	2.10E-06	4.45E-05	4.515908258
IL2RB	1.101651971	3.794522532	4.964670699	2.13E-06	4.51E-05	4.50102196
SIPA1	0.721417999	6.606327413	4.964593958	2.13E-06	4.51E-05	4.500705587
POLE2	-0.585694988	4.268390912	-4.957344535	2.20E-06	4.63E-05	4.470833898
GOLGA8N	-0.727359438	3.312459848	-4.953771091	2.23E-06	4.70E-05	4.456120133
GPT2	-1.114290226	3.792895552	-4.953290746	2.24E-06	4.70E-05	4.454142844
ACOT9	0.651530451	4.231825351	4.94540214	2.32E-06	4.84E-05	4.421688754
ABCA7	0.933319907	5.174789045	4.944760654	2.32E-06	4.85E-05	4.419051185
IRF2BPL	0.642067164	4.15996724	4.942404193	2.35E-06	4.88E-05	4.409364223
MAL	1.132666323	2.271359421	4.939678778	2.38E-06	4.91E-05	4.398164454
C9orf43	-1.171189317	3.183069606	-4.939654669	2.38E-06	4.91E-05	4.398065401
MPZL3	0.645136413	2.634933954	4.939349608	2.38E-06	4.91E-05	4.396812051
NREP	-0.701357514	5.67287383	-4.937228265	2.40E-06	4.95E-05	4.388097928
SBNO2	0.638195022	5.133997768	4.935209082	2.42E-06	4.98E-05	4.379805814
HORMAD1	0.876385355	1.475646376	4.935137189	2.42E-06	4.98E-05	4.379510619
PLAAT4	0.767489419	4.401109127	4.933962102	2.43E-06	5.00E-05	4.37468603
SPOCK2	1.04621251	4.148595906	4.933096918	2.44E-06	5.02E-05	4.371134315
YPEL3	0.641056777	5.59276357	4.923816574	2.54E-06	5.20E-05	4.333063613
RGS9BP	-0.668715703	0.854319345	-4.923504756	2.55E-06	5.21E-05	4.331785291
ABCD1	0.711376887	4.409227841	4.92309408	2.55E-06	5.21E-05	4.330101772
SLC43A1	-0.610621441	5.34114339	-4.921899511	2.56E-06	5.22E-05	4.325205313
SLC31A1	0.650295927	4.050980923	4.918520147	2.60E-06	5.29E-05	4.311357898
LGALS1	1.47704558	7.670338398	4.914977075	2.64E-06	5.35E-05	4.296846599
ETV7	0.597649597	0.627806503	4.912106717	2.67E-06	5.40E-05	4.285095725
CLEC10A	1.712030699	2.091705368	4.899593599	2.82E-06	5.68E-05	4.233923174
IRF1	0.661252441	5.688114636	4.894364408	2.89E-06	5.79E-05	4.212564652
SGMS2	1.374295016	2.44308821	4.891998102	2.92E-06	5.84E-05	4.202904627
OR52K1	0.726348782	0.734311087	4.89076831	2.93E-06	5.87E-05	4.197885478
SRD5A1	0.653316829	2.3576173	4.890492428	2.93E-06	5.87E-05	4.196759642
VSIG4	1.730799007	3.359857215	4.887962076	2.97E-06	5.93E-05	4.186435619
KCNJ15	0.592937402	0.467367111	4.884280656	3.01E-06	6.01E-05	4.171421666
ZNF10	-0.615195008	2.960462244	-4.881780596	3.05E-06	6.07E-05	4.161230061
IRF8	1.663105037	5.103193362	4.878104667	3.09E-06	6.16E-05	4.14625144
RASA3	0.681171688	5.278218858	4.875008766	3.14E-06	6.23E-05	4.133642291
GPR137B	0.81030742	3.149216545	4.87250154	3.17E-06	6.27E-05	4.123434736
IQSEC2	0.983905124	2.861766531	4.871510137	3.18E-06	6.29E-05	4.119399473

BMF	0.710542076	4.491564154	4.869979376	3.20E-06	6.32E-05	4.113169988
ANXA2	1.383885907	5.247050435	4.867788667	3.23E-06	6.37E-05	4.104257152
ZC2HC1A	0.867446235	1.270022491	4.86445546	3.28E-06	6.45E-05	4.090701358
KCTD7	0.794065163	1.999590188	4.864221806	3.28E-06	6.45E-05	4.089751351
RASSF4	1.290045155	4.12388081	4.860741139	3.33E-06	6.53E-05	4.075603105
MCUB	0.617661523	4.191990363	4.85978897	3.35E-06	6.54E-05	4.071733929
PLA2G4C	0.723187669	0.996607746	4.857122621	3.38E-06	6.60E-05	4.060901877
TXNIP	0.706121476	9.105441595	4.856807838	3.39E-06	6.60E-05	4.059623338
PLCB2	0.879640063	7.018468536	4.856458269	3.39E-06	6.60E-05	4.058203576
AGPAT3	0.808737011	4.471736908	4.854954959	3.42E-06	6.64E-05	4.052098744
TBX21	0.899042839	2.167369119	4.854666722	3.42E-06	6.64E-05	4.050928384
DOCK9	0.812965154	1.427134182	4.849956568	3.49E-06	6.75E-05	4.03180996
CAST	0.88170048	4.950903746	4.848851898	3.51E-06	6.77E-05	4.027327967
FLNA	0.769207146	8.61089773	4.84858013	3.51E-06	6.78E-05	4.026225427
PLN	0.666270349	1.204614557	4.843237144	3.59E-06	6.92E-05	4.004557995
CD8A	0.953390705	2.931047869	4.837070658	3.69E-06	7.07E-05	3.97957142
AATK	0.852683272	2.285864482	4.834832686	3.72E-06	7.13E-05	3.97050858
DAPP1	0.873994546	4.797974564	4.825584652	3.87E-06	7.38E-05	3.933088562
SKA3	-0.712648508	4.090725407	-4.824140693	3.89E-06	7.42E-05	3.927250371
GRN	1.166910921	8.333753343	4.823522297	3.90E-06	7.43E-05	3.924750452
SRGAP2	0.748740347	3.871591001	4.818246731	3.99E-06	7.57E-05	3.903432492
HK1	0.76960359	5.685478198	4.814331664	4.06E-06	7.68E-05	3.887622554
IL13RA1	1.345122575	4.875219807	4.814070125	4.06E-06	7.68E-05	3.886566714
SGMS1	0.655316022	3.413588929	4.807552262	4.18E-06	7.87E-05	3.860266754
CORO1C	0.587402285	5.828314827	4.802239347	4.27E-06	8.02E-05	3.838847044
GRK3	0.695222745	4.961485295	4.798911625	4.33E-06	8.11E-05	3.825439237
RASSF2	0.69773809	6.725843193	4.796596781	4.38E-06	8.17E-05	3.816116234
ZNF107	-0.820601543	4.725086367	-4.793771558	4.43E-06	8.26E-05	3.804741904
SLC18B1	0.91426343	2.622138592	4.791044625	4.48E-06	8.33E-05	3.793767689
MTMR11	1.491784478	2.109279975	4.789551926	4.51E-06	8.36E-05	3.787762333
BRIP1	-0.679927459	3.548997026	-4.789451086	4.51E-06	8.36E-05	3.787356687
FLVCR2	0.825742473	2.515657	4.787609996	4.55E-06	8.40E-05	3.779951593
SKAP1	0.980226649	3.108107698	4.786972053	4.56E-06	8.41E-05	3.777386167
FOSL2	0.828992855	6.887586668	4.777204595	4.75E-06	8.71E-05	3.73813689
CD28	0.875180352	2.137424488	4.776635259	4.76E-06	8.73E-05	3.7358508
CD6	0.959975155	3.031255374	4.775710982	4.78E-06	8.74E-05	3.732139902
SBF2	0.739560177	3.306455983	4.775243086	4.79E-06	8.74E-05	3.730261525
IL6ST	0.915252245	4.461585045	4.774891927	4.80E-06	8.74E-05	3.728851878
SCPEP1	1.421041133	5.313692399	4.774845091	4.80E-06	8.74E-05	3.728663867
GNS	0.790403865	6.338024125	4.774833233	4.80E-06	8.74E-05	3.72861627
TNFAIP3	0.814012185	5.355255476	4.769840531	4.90E-06	8.92E-05	3.708582138
DMWD	0.788218702	2.063897884	4.768169801	4.94E-06	8.97E-05	3.701881282
CCDC169	-0.660677919	1.444418423	-4.755756711	5.20E-06	9.40E-05	3.652146794
SLC2A3	1.009575685	5.846480206	4.751513655	5.29E-06	9.55E-05	3.635167201
ENOSF1	-0.639161804	4.866787948	-4.750749004	5.31E-06	9.56E-05	3.632108393
KLHL23	-0.867015074	3.347207636	-4.748344838	5.37E-06	9.64E-05	3.62249332
CBR1	0.84522348	3.772782139	4.74573997	5.42E-06	9.70E-05	3.612079402

CENPJ	-0.643999324	4.946063771	-4.739967803	5.56E-06	9.93E-05	3.589017246
GIMAP6	1.058613257	4.684548374	4.73742958	5.62E-06	0.0001001	3.578882218
STOX1	-0.611152974	1.301347057	-4.729799354	5.80E-06	0.000102987	3.548437851
GRK5	0.731497229	2.131961609	4.729786204	5.80E-06	0.000102987	3.548385413
DUSP1	0.959169314	9.045362733	4.724981597	5.92E-06	0.000104893	3.529232824
MS4A4E	1.674807898	4.989269457	4.724253344	5.94E-06	0.000105118	3.52633098
EHD4	0.850511622	3.221929073	4.722706181	5.98E-06	0.000105706	3.520167093
AIM2	0.929605556	2.083669993	4.718717116	6.08E-06	0.00010739	3.504281208
ZSCAN31	-0.667532506	2.681562919	-4.71839395	6.08E-06	0.000107438	3.502994659
PRDM1	0.691902152	2.877429293	4.716914699	6.12E-06	0.000108008	3.497106423
HEG1	0.897645359	2.228791378	4.715985136	6.15E-06	0.000108331	3.493406913
CXCR1	0.841029639	1.216516608	4.713950599	6.20E-06	0.000109159	3.485311567
FAIM	-0.611234446	3.449484657	-4.710964177	6.28E-06	0.000110035	3.473433138
FCRL3	0.85027205	2.253015056	4.700713429	6.55E-06	0.000114238	3.432701203
BAG3	1.037415104	1.951949149	4.699481743	6.59E-06	0.000114568	3.427811223
STK26	-0.601811559	6.802646228	-4.69937997	6.59E-06	0.000114568	3.42740721
VOPP1	0.696327615	3.248072356	4.694631793	6.72E-06	0.000116655	3.408564932
ACSL1	1.075658071	5.533806126	4.692966342	6.77E-06	0.000117052	3.401959065
TRIB1	1.07448602	5.715973837	4.692485704	6.78E-06	0.000117182	3.400052965
ZSWIM6	0.626765333	4.711825204	4.672433161	7.37E-06	0.000126437	3.320651623
FCGBP	0.75023488	0.85413957	4.67234527	7.37E-06	0.000126437	3.320304133
RAB31	1.485886173	4.434589745	4.669235488	7.47E-06	0.000127737	3.308012073
DGAT2	0.766002636	1.544983241	4.669042921	7.48E-06	0.000127737	3.307251106
HACD1	-0.774193427	3.732086916	-4.668574771	7.49E-06	0.000127874	3.305401199
ACP5	0.79972996	2.946741112	4.667802726	7.52E-06	0.000128173	3.30235073
TMEM88	0.606637299	3.01602788	4.661169383	7.73E-06	0.000131299	3.276156063
SDC3	1.036443179	1.507383271	4.659949892	7.76E-06	0.000131805	3.271343233
RNF144B	1.075781732	3.712354852	4.65982396	7.77E-06	0.000131805	3.27084628
CERCAM	-1.066310629	3.69281394	-4.658437084	7.81E-06	0.000132336	3.265374034
ZBTB10	-0.591360105	3.65811377	-4.652160763	8.02E-06	0.000135243	3.240623739
STX3	0.691212378	4.525083153	4.651451486	8.04E-06	0.000135525	3.237828233
TLR6	0.981072149	3.723108823	4.649670493	8.10E-06	0.000136201	3.230810055
BRCA1	-0.658329427	4.534236304	-4.649628338	8.10E-06	0.000136201	3.230643961
CEBPD	1.267837891	5.724702761	4.649335748	8.11E-06	0.000136248	3.22949117
TMEM44	-0.617439569	3.976651619	-4.648284015	8.15E-06	0.000136727	3.225347815
PUDP	0.631849721	3.557066386	4.642911655	8.33E-06	0.000139302	3.204193498
JAK2	0.649863691	5.527801712	4.642749494	8.34E-06	0.000139302	3.203555239
HERPUD1	0.647263243	5.507596729	4.642293259	8.36E-06	0.000139446	3.201759602
PLA2G7	0.899454453	1.0873972	4.640645452	8.41E-06	0.000140247	3.19527525
CCR4	0.746406754	1.590933421	4.639072727	8.47E-06	0.000140947	3.189087875
PDE2A	0.600357379	0.882376113	4.638885109	8.47E-06	0.000140947	3.188349852
LYZ	2.062336639	10.98525357	4.634632535	8.62E-06	0.00014333	3.171627417
ADAM9	0.929385975	3.621388102	4.632140511	8.71E-06	0.000144447	3.161833067
PLEKHO1	0.607802344	5.213325156	4.628709741	8.84E-06	0.000146264	3.148355299
PLK3	0.651664649	4.683337314	4.624304381	9.00E-06	0.000148198	3.131059264
PAOX	0.736914925	2.191585624	4.621636568	9.10E-06	0.000149589	3.120590759
CD53	0.591221063	8.671482078	4.613629779	9.41E-06	0.000153713	3.089197906

CPEB4	0.595513513	4.09860651	4.610678766	9.52E-06	0.000155337	3.077637406
SLC49A3	0.873909412	2.871521809	4.609466903	9.57E-06	0.000155985	3.072891498
MIDN	0.637821209	6.173029957	4.607523191	9.65E-06	0.000157052	3.065281372
IL7R	1.03451935	4.197528013	4.604619359	9.76E-06	0.000158606	3.053916389
PLXNA1	0.831406907	2.048759132	4.603946302	9.79E-06	0.000158781	3.051282918
CDC42EP1	1.156193438	1.759294306	4.601365843	9.90E-06	0.000160347	3.041188888
RMDN2	0.6591303	1.396508695	4.598100672	1.00E-05	0.000162208	3.028422242
CCDC34	-0.617117521	2.860450789	-4.597960305	1.00E-05	0.000162208	3.02787356
PRKCD	0.877652188	6.83528728	4.596425078	1.01E-05	0.000162945	3.021873272
SPIN4	-0.80002107	4.922614286	-4.59625672	1.01E-05	0.000162945	3.021215345
TRNP1	0.638725202	0.721559076	4.595587355	1.01E-05	0.00016326	3.018599713
CENPV	-0.587715243	4.32840902	-4.594985821	1.02E-05	0.000163394	3.016249369
JPH4	0.653183862	1.408421505	4.586618304	1.05E-05	0.000168426	2.983578187
CD300LF	0.94936451	5.758063142	4.583944665	1.06E-05	0.000170009	2.973147865
LILRB5	0.612863844	0.613079755	4.583088273	1.07E-05	0.000170404	2.969807854
TTYH3	0.790150837	5.7893808	4.580309062	1.08E-05	0.000172004	2.958971735
ACSM3	-1.297613058	4.376431386	-4.579636604	1.08E-05	0.000172198	2.956350537
VSIG2	1.142457905	2.999690491	4.578538135	1.09E-05	0.000172629	2.952069363
OAS3	0.997503379	4.367115161	4.578246489	1.09E-05	0.000172629	2.950932823
ZYX	0.657912164	8.346953231	4.578234101	1.09E-05	0.000172629	2.950884548
NBEAL2	0.753497355	7.116461518	4.570289645	1.12E-05	0.000177384	2.919945186
EOMES	0.742695791	1.868455784	4.570234622	1.12E-05	0.000177384	2.919731036
JDP2	0.756179461	3.637464856	4.568458354	1.13E-05	0.000178537	2.912818758
TRABD2A	0.682181675	1.731147122	4.566118578	1.14E-05	0.000180114	2.903716548
AGPAT5	-0.646075851	5.346520497	-4.562801721	1.16E-05	0.000182286	2.89081902
VWCE	-0.904214502	2.724728412	-4.561761225	1.16E-05	0.000182828	2.886774459
TIMP1	0.994717768	7.216851635	4.561684294	1.16E-05	0.000182828	2.886475444
CD3G	0.879188398	2.593186574	4.56146424	1.17E-05	0.000182846	2.885620157
BACH2	0.832846502	2.173591671	4.558787955	1.18E-05	0.000184417	2.875220572
KIAA0040	0.646354908	4.660755946	4.55847597	1.18E-05	0.000184505	2.874008536
FRAT2	0.598163406	5.237327918	4.55733447	1.19E-05	0.000184805	2.869574412
PDZD4	0.692392571	1.396856331	4.556335238	1.19E-05	0.00018509	2.865693577
REXO5	-0.628532478	4.307199943	-4.554364154	1.20E-05	0.000186175	2.858040043
MYO1G	0.728327848	6.308411448	4.547635473	1.23E-05	0.000190736	2.831931123
ZNF831	0.62896947	1.468303994	4.543500931	1.26E-05	0.000193375	2.815901862
SLC35F6	0.599810879	4.484837117	4.541923977	1.26E-05	0.000193872	2.809790909
GOLGA8R	-0.686036984	2.416945118	-4.541766809	1.26E-05	0.000193872	2.809181942
PLD3	0.814221726	6.092690116	4.541594287	1.26E-05	0.000193872	2.808513499
FAM50A	0.620739161	4.713518854	4.541522362	1.27E-05	0.000193872	2.808234831
SIRPA	0.834436139	5.722664984	4.541129655	1.27E-05	0.00019403	2.806713359
RELL2	0.829498843	2.331983565	4.540048963	1.27E-05	0.000194736	2.802526913
LCP1	0.69411603	9.478132629	4.538235346	1.28E-05	0.000195714	2.795502828
LMNA	1.358058273	4.744709858	4.538069802	1.28E-05	0.000195714	2.794861782
TLR4	1.333291401	4.863340009	4.53805572	1.28E-05	0.000195714	2.79480725
HLA-DMA	1.10942667	6.903804456	4.537189393	1.29E-05	0.000196254	2.791452802
THEMIS	0.822025004	1.927451001	4.535674657	1.30E-05	0.000197318	2.785588796
SLC30A1	0.605774544	4.008707634	4.531286498	1.32E-05	0.000200259	2.768608858

C1QB	1.75640665	2.760989761	4.530716588	1.32E-05	0.000200471	2.766404465
CD52	1.395532077	7.030646135	4.525841889	1.35E-05	0.000203963	2.747557465
MNDA	1.495478153	7.196184452	4.518839773	1.39E-05	0.000209383	2.720510863
ADAM22	-0.69008201	1.600604217	-4.515156439	1.41E-05	0.00021222	2.706295635
SYNE2	0.843299123	2.098377199	4.5112379	1.43E-05	0.0002153	2.691181864
CHST10	-0.59966693	3.239815041	-4.511029852	1.43E-05	0.000215316	2.69037969
SLC31A2	0.699506887	2.554655528	4.509734668	1.44E-05	0.000216287	2.685386429
ZCCHC24	0.810590453	2.596728912	4.509292546	1.44E-05	0.00021651	2.683682174
PLK4	-0.587808452	4.203948232	-4.50538184	1.47E-05	0.000219475	2.668612772
ACSS2	0.620157452	4.736895704	4.501490185	1.49E-05	0.000222349	2.653626176
PTPN18	0.599741593	6.231498401	4.501426268	1.49E-05	0.000222349	2.65338011
PECR	0.79694493	1.414107153	4.496848536	1.52E-05	0.000225659	2.63576364
RASL11A	0.697042983	1.794539179	4.495099412	1.53E-05	0.000227095	2.629035922
TMEM176A	1.403878725	2.231555427	4.493964322	1.54E-05	0.00022797	2.624670997
TMEM67	-0.650126841	2.174968661	-4.492431054	1.55E-05	0.000229044	2.618776173
DUSP7	0.61759357	4.348996297	4.488596226	1.57E-05	0.000232284	2.604039116
RBM38	0.870219434	5.400029529	4.481825123	1.61E-05	0.000238207	2.578040395
FBP1	1.492232231	3.899805908	4.479460729	1.63E-05	0.000240319	2.568968638
TFDP2	-0.701531951	5.575139698	-4.474712261	1.66E-05	0.000244801	2.550760127
SLC49A4	0.6080232	3.72477268	4.474024715	1.67E-05	0.000245297	2.548124817
AOAH	1.175236763	6.124721998	4.473233627	1.67E-05	0.000245553	2.545093008
CD1B	0.640360543	0.41282489	4.471871611	1.68E-05	0.000246512	2.539874055
SIPA1L1	0.693528591	3.722346741	4.467539541	1.71E-05	0.000250216	2.523282174
LY86	1.678924768	5.038448468	4.466958335	1.71E-05	0.00025048	2.521057038
HLA-DRA	1.441722145	10.15012988	4.466810013	1.71E-05	0.00025048	2.520489222
EVI2B	0.702903779	8.239165693	4.466100985	1.72E-05	0.000250911	2.517775071
RBP7	0.908797875	1.493807902	4.464789885	1.73E-05	0.000251778	2.512757025
HLA-DPA1	1.497247241	6.408863457	4.463523092	1.74E-05	0.000252882	2.507909574
HMGB3	-0.671271534	3.979637203	-4.463062774	1.74E-05	0.000253164	2.506148391
ZNF385C	-0.967564454	2.269813488	-4.456238658	1.79E-05	0.00025908	2.480054745
TEX30	-0.616781019	4.471910262	-4.453905655	1.81E-05	0.000261144	2.47114062
DIXDC1	-0.629214171	1.686764247	-4.453615375	1.81E-05	0.000261256	2.470031732
SLC26A6	0.675236971	3.885408723	4.452001566	1.82E-05	0.000262573	2.463867845
S1PR1	0.888013664	3.258569335	4.447464482	1.85E-05	0.000266831	2.446547339
SLC22A16	-0.66082542	4.65636483	-4.439506041	1.91E-05	0.000273708	2.416196799
SORT1	1.591754699	3.576674497	4.43761781	1.93E-05	0.000275393	2.40900161
TCTEX1D4	0.697494845	2.674982716	4.437369219	1.93E-05	0.00027542	2.40805451
HLA-DRB5	1.587720154	6.977939748	4.435955672	1.94E-05	0.000276634	2.402669812
IL17RA	1.063943217	6.196942285	4.433964939	1.96E-05	0.000278656	2.395088537
DPY19L2	-1.254474943	3.546105084	-4.433168591	1.96E-05	0.000279346	2.392056513
FAM20A	0.610175264	1.408481832	4.430805609	1.98E-05	0.000281193	2.38306202
ANKRD33B	-0.908393771	3.003574041	-4.424983026	2.03E-05	0.000287437	2.360913812
GZMK	0.884113461	2.677336443	4.424716078	2.03E-05	0.000287536	2.359898893
TMEM150B	1.189243291	3.217428568	4.415087014	2.11E-05	0.000297363	2.323319831
MS4A1	0.896582749	3.451825021	4.413568174	2.12E-05	0.000298346	2.317555368
MPP7	0.966455809	3.002767384	4.413544528	2.12E-05	0.000298346	2.317465634
TARBP1	-0.607417603	6.217062419	-4.413027286	2.13E-05	0.000298646	2.315502882

CARD17	0.821423168	1.189353626	4.412772545	2.13E-05	0.000298646	2.314536292
AHNAK	0.88675463	7.843533598	4.409290624	2.16E-05	0.00030239	2.301328566
FNIP2	0.668426988	3.228650157	4.394448402	2.29E-05	0.000318399	2.24511448
NDC80	-0.641103216	3.76062473	-4.393892809	2.30E-05	0.000318672	2.243012909
LDLR	0.927972654	4.004546918	4.39293758	2.31E-05	0.000319646	2.239400135
FCER2	1.243832958	3.23790388	4.391823295	2.32E-05	0.000320846	2.235186529
ANKRD55	0.599119981	1.17148588	4.391433886	2.32E-05	0.000321118	2.233714189
CLIP4	1.025454861	3.102890036	4.391202583	2.32E-05	0.000321186	2.232839685
ZNF439	-0.735625216	4.635037369	-4.390119192	2.33E-05	0.000322082	2.22874408
MICAL1	0.834530503	6.061981847	4.389973939	2.34E-05	0.000322082	2.228195029
GPR162	0.985059967	3.580561944	4.38696448	2.36E-05	0.00032483	2.216822385
KIF15	-0.701228226	3.758385531	-4.383318181	2.40E-05	0.000329238	2.203050832
NHSL2	1.207162612	3.70204852	4.383238574	2.40E-05	0.000329238	2.202750262
NUDT16	0.697893458	4.155256848	4.380349276	2.43E-05	0.000332123	2.191843929
ARL4C	0.826283765	4.152206861	4.377762367	2.45E-05	0.000335122	2.182083532
PNPLA6	0.634426814	5.187376501	4.374083251	2.49E-05	0.000337921	2.168209558
ALOX5AP	1.298699855	5.747483354	4.37202957	2.51E-05	0.000340154	2.160468852
FAM20C	1.11213718	2.611780418	4.369519293	2.53E-05	0.00034317	2.151010792
KYNU	1.018758331	2.184306621	4.368438525	2.55E-05	0.000344261	2.146939981
LTK	-1.388642453	3.495519566	-4.368374574	2.55E-05	0.000344261	2.146699128
DTL	-0.646076504	4.660682518	-4.36634875	2.57E-05	0.00034633	2.139070756
SASH1	0.758321969	0.656065402	4.362492801	2.61E-05	0.000351204	2.124558149
GPR84	0.823735176	1.736992922	4.355207127	2.68E-05	0.000359267	2.097162969
ADAM8	1.103938605	5.263469026	4.355056957	2.68E-05	0.000359267	2.096598664
KIAA1549	-0.869821104	2.775702783	-4.352055998	2.72E-05	0.000362832	2.085324756
MAP3K6	0.816747864	3.358507655	4.347704896	2.76E-05	0.000368361	2.068988887
HTATIP2	0.815585887	3.857149017	4.345839974	2.78E-05	0.000370382	2.061990885
CD74	1.212072908	10.44922754	4.343976175	2.81E-05	0.000372117	2.05499932
NAAA	0.640834831	4.83813799	4.342067589	2.83E-05	0.000374183	2.047842048
PAPSS2	0.725460807	2.609208898	4.338831259	2.86E-05	0.000378763	2.03571101
SOCS3	1.165619209	5.304589533	4.337379145	2.88E-05	0.000380693	2.030270094
DPEP2	0.646434067	5.382775216	4.332370976	2.94E-05	0.000387804	2.011515376
ZNF253	-0.629174136	4.032726467	-4.329412538	2.97E-05	0.000392112	2.000444092
UBTD1	0.777070951	3.256853734	4.3244635	3.03E-05	0.000397986	1.98193599
ANO9	0.792716099	2.510521313	4.320744213	3.08E-05	0.000403073	1.968037178
FGFBP2	0.994297621	2.863592004	4.319867686	3.09E-05	0.000404202	1.964762928
ZNF532	0.990966685	1.594009767	4.319189495	3.09E-05	0.000405015	1.962229899
POGLUT3	-0.612180561	4.357711516	-4.310412185	3.20E-05	0.000417315	1.929473487
THBD	0.847012475	2.282469083	4.310392472	3.20E-05	0.000417315	1.929399972
DDX60L	0.659640223	4.717952687	4.30948254	3.22E-05	0.000417683	1.926007006
PLCL2	0.695133073	4.307011269	4.307616509	3.24E-05	0.0004203	1.919050594
STXBP2	0.598980496	5.418660382	4.301264878	3.32E-05	0.000429539	1.895389033
FAM151A	-0.604661584	2.18611863	-4.289598186	3.48E-05	0.000444734	1.851995214
CD2	0.953448641	4.226900591	4.289281517	3.48E-05	0.000444996	1.850818603
KANK1	-0.693319062	3.316257048	-4.288829281	3.49E-05	0.000445409	1.849138389
CYBA	0.593012711	7.595451565	4.285563603	3.53E-05	0.000449786	1.837009194
MFSD2A	0.854924752	2.56398814	4.283656596	3.56E-05	0.000452583	1.829929492

FCGR3B	1.125782152	1.333719768	4.282932561	3.57E-05	0.00045331	1.827242148
SMAD3	0.620625688	4.573473138	4.282916945	3.57E-05	0.00045331	1.827184192
PITPNM1	0.652617707	5.212950169	4.281851721	3.59E-05	0.00045462	1.823231116
B4GALT2	-0.7187942	3.488732806	-4.276453848	3.66E-05	0.000463166	1.803210745
CRYBG1	0.710656535	5.507423869	4.274477265	3.69E-05	0.000466475	1.795884442
SKIL	0.671889744	2.986767813	4.274029507	3.70E-05	0.000466993	1.794225159
WT1	-1.107670208	3.087281923	-4.271466664	3.74E-05	0.000471107	1.784730372
GOLGA8Q	-0.631669248	1.547847181	-4.261836049	3.88E-05	0.000486416	1.749089099
KRT23	0.70145239	0.586116104	4.25995452	3.91E-05	0.000489703	1.742132914
GOLGA8O	-0.73834091	2.147618627	-4.259564618	3.91E-05	0.000490136	1.740691697
ANKRD36C	-0.624190546	4.523739324	-4.256875774	3.96E-05	0.000494375	1.730755469
GZMM	0.740497354	2.590080326	4.256644431	3.96E-05	0.000494505	1.729900796
IFITM3	1.53294713	5.681019218	4.253757832	4.00E-05	0.000499168	1.719239465
PLAGL1	-0.628465634	5.676442381	-4.253256767	4.01E-05	0.000499828	1.717389389
SAMD3	0.661976787	1.553667006	4.251656114	4.04E-05	0.000502649	1.711480415
CD8B	0.733588074	2.054062153	4.245828513	4.13E-05	0.00051326	1.689981327
CYTL1	-1.710294702	6.140596357	-4.24304487	4.18E-05	0.000518216	1.679719768
OLR1	0.884105073	1.181428371	4.239658316	4.23E-05	0.000523453	1.66724247
MCOLN2	0.804201154	1.520148551	4.236518247	4.28E-05	0.000528906	1.65568
VNN1	1.781495458	3.874046957	4.235155912	4.31E-05	0.00053139	1.650665563
NAIP	0.928347248	2.530954584	4.233915142	4.33E-05	0.000533633	1.646099636
SETBP1	1.144774431	1.906421851	4.229823959	4.40E-05	0.00054051	1.631051562
ZNF70	-0.603727385	4.464593005	-4.22241611	4.53E-05	0.0005546	1.60383208
GNLY	1.175750098	3.891210421	4.221668996	4.54E-05	0.000555867	1.601088869
SCGB1A1	0.596653433	0.779970444	4.221233012	4.55E-05	0.000556462	1.599488218
FRAT1	0.617257654	4.289900507	4.213654057	4.68E-05	0.000570262	1.571683088
SFXN3	0.809405335	5.274079278	4.206600519	4.81E-05	0.000582442	1.545839425
IFI6	0.928583818	5.908765922	4.196588128	5.00E-05	0.000602582	1.509210869
SMIM14	0.767290316	3.394846774	4.195445497	5.03E-05	0.000604886	1.50503495
XRCC2	-0.596001743	3.832626056	-4.194583996	5.04E-05	0.000606536	1.501887029
LEF1	0.958146749	2.991800903	4.191458741	5.10E-05	0.000612043	1.490471467
CD22	0.879894606	2.867270207	4.188004767	5.17E-05	0.000619912	1.477862688
CDC25A	-0.590498429	3.621643567	-4.185516867	5.22E-05	0.000624764	1.468785451
GSAP	0.683189789	3.924007113	4.181388318	5.31E-05	0.000634055	1.453731218
HLA-DPB1	1.306579291	7.030764599	4.17325068	5.48E-05	0.000652336	1.424091311
GATA3	0.836578698	2.118123862	4.172400791	5.50E-05	0.000653683	1.420998265
FNDC3B	-0.644305571	5.725839445	-4.170771758	5.53E-05	0.00065701	1.415070975
ATP6V0A1	0.835244124	4.329968394	4.167026798	5.61E-05	0.000664945	1.401451469
BUB1B	-0.633812741	4.588069214	-4.166875955	5.61E-05	0.000664945	1.400903083
NCR3LG1	-0.787284485	2.522108946	-4.166088177	5.63E-05	0.000666161	1.398039389
GCH1	0.896749188	4.423953165	4.165272985	5.65E-05	0.000667608	1.39507647
TSC22D3	0.782776566	7.560616866	4.152394327	5.94E-05	0.000696376	1.348325806
CALR	-0.695315948	9.999773734	-4.146313948	6.08E-05	0.000710611	1.326291741
MAF	0.717162859	2.050948606	4.14270821	6.16E-05	0.000715936	1.313236881
BUB1	-0.640091994	4.359068027	-4.14015575	6.22E-05	0.000721708	1.304000721
HEXB	0.638717989	5.337256006	4.137304376	6.29E-05	0.00072793	1.293688059
DEPDC1B	-0.652561157	3.561019698	-4.13490212	6.35E-05	0.000732939	1.28500394

ITGA7	0.973671866	2.464233437	4.134488352	6.36E-05	0.00073367	1.283508562
ADD3	0.657498826	6.038658067	4.134086708	6.37E-05	0.000734152	1.282057109
CDKN1A	1.080406911	5.00246818	4.132714203	6.40E-05	0.000736941	1.277097995
NRBP2	0.873929322	2.686048098	4.130997714	6.45E-05	0.00074094	1.270897771
NR1I2	-0.713636558	2.050262322	-4.129190832	6.49E-05	0.000745422	1.264373151
ADGRE5	0.637788573	7.706583502	4.129119467	6.49E-05	0.000745422	1.264115497
MYO1E	0.69507919	2.069297516	4.126589899	6.56E-05	0.000752251	1.254985053
FYB1	0.672844424	6.701804837	4.123087792	6.64E-05	0.000760186	1.242351283
PRLR	0.967614905	1.188476145	4.122289391	6.67E-05	0.000761706	1.239472219
PARP14	0.640623044	5.570301233	4.115920915	6.83E-05	0.000777702	1.216522473
TMEM176B	1.564506671	3.247649078	4.115034193	6.85E-05	0.000779436	1.213329189
PLAUR	0.940926126	4.809658668	4.112449426	6.92E-05	0.000786287	1.204023865
DPYSL2	1.176921265	4.533313298	4.112444019	6.92E-05	0.000786287	1.204004404
C1QC	1.582469831	2.725427506	4.111031176	6.96E-05	0.00078963	1.198919974
CACNA2D3	0.960341461	1.810206296	4.106581933	7.08E-05	0.000799459	1.182917113
TLN1	0.605433381	7.945676009	4.10487048	7.12E-05	0.000802275	1.176764954
HLA-DQA1	1.309675807	4.246838202	4.101962722	7.20E-05	0.000809944	1.16631694
PLXNC1	0.860553968	4.515434175	4.101364635	7.22E-05	0.000811329	1.164168627
RNF213	0.61396624	5.942536867	4.096534035	7.36E-05	0.000822169	1.146826017
SEPTIN1	0.654956669	3.677594121	4.094783986	7.40E-05	0.000825311	1.14054693
PRSS57	-1.26801037	8.24163735	-4.091601614	7.50E-05	0.000833487	1.129134011
ADAM15	0.606150793	5.668057705	4.08962334	7.55E-05	0.000839321	1.122042766
GCNT2	0.742707883	2.184992366	4.08919837	7.56E-05	0.000840203	1.120519778
CDK14	-0.759088884	2.897264097	-4.086123749	7.65E-05	0.000849142	1.109504707
PM20D2	-0.653573204	5.695302724	-4.085823042	7.66E-05	0.000849632	1.108427744
TBC1D8	0.745795645	3.871018173	4.080226767	7.83E-05	0.000863329	1.088396098
PLTP	-0.789997276	5.006503034	-4.074262024	8.01E-05	0.000879854	1.06706874
PIK3AP1	0.587021696	6.522425452	4.06840025	8.19E-05	0.000896146	1.046132907
NOXA1	0.962762261	3.361168079	4.067831357	8.21E-05	0.000897581	1.044102291
HAVCR2	0.965095465	4.085692229	4.06720863	8.22E-05	0.000899202	1.04187977
SLC24A4	1.058978784	2.665330398	4.055705865	8.59E-05	0.000931492	1.0008733
PPM1H	-0.813657775	3.284255232	-4.052629184	8.69E-05	0.000939615	0.989920327
BCL11B	0.795491902	2.456202607	4.050906353	8.75E-05	0.000944912	0.983789848
OSCAR	1.269907744	5.371567358	4.050534785	8.76E-05	0.000945719	0.982467935
SAMD9L	0.694892317	5.068517746	4.049632576	8.79E-05	0.000947155	0.979258566
MSRB1	0.716364807	5.248645699	4.04673313	8.89E-05	0.000953353	0.968948291
STEAP4	0.625366271	0.855301692	4.045540369	8.93E-05	0.000956882	0.964708548
CCL4	0.714787014	2.534537136	4.043557522	9.00E-05	0.000963032	0.95766253
FMN1	0.642803467	0.923432447	4.04036866	9.11E-05	0.000973338	0.946336544
MLNR	-0.662875437	1.656779304	-4.039000316	9.15E-05	0.000977631	0.941478664
DPP4	0.688799952	1.570606436	4.034395119	9.31E-05	0.000992079	0.92513868
KSR1	0.630114403	4.030734807	4.033304878	9.35E-05	0.000995086	0.921272435
GABRR2	0.650792555	2.091529721	4.033064061	9.36E-05	0.000995447	0.920418554
ST14	1.199506224	2.721226034	4.030984173	9.43E-05	0.001001658	0.913045371
ENHO	0.675959451	0.641646404	4.030294277	9.46E-05	0.001003177	0.910600348
IKZF3	0.745365685	3.261758073	4.029307883	9.49E-05	0.001005824	0.907105081
PRNP	0.638640905	5.928159528	4.024708066	9.66E-05	0.001019003	0.890814456

HLA-DQB1	1.425675423	5.311025635	4.018574103	9.89E-05	0.001036669	0.869112918
SLC44A2	0.79158426	5.139382441	4.018317546	9.90E-05	0.001037114	0.868205794
IRF5	0.630686968	5.830236857	4.015946759	9.99E-05	0.001044733	0.859825389
PYCARD	0.603184065	5.782293231	4.015791514	9.99E-05	0.001044782	0.859276754
IQGAP1	0.727051507	6.465019587	4.015004695	0.000100211	0.001046759	0.856496386
MMP17	1.082670883	2.787543631	4.013429311	0.000100808	0.001050986	0.850930748
IL31RA	0.72344343	0.800184855	4.007884085	0.000102934	0.001070602	0.831353612
HIVEP3	0.66465794	3.378427903	4.007075912	0.000103247	0.001072715	0.828502148
SPON1	1.180501213	1.581908325	4.006787396	0.00010336	0.001073307	0.827484291
TNFSF13	0.678407928	4.107617136	4.004758274	0.000104151	0.001079801	0.820327337
CXCL1	0.942843249	1.723405149	3.998365601	0.000106684	0.001103706	0.797798005
BTG1	0.632446427	6.529325478	3.995464987	0.000107852	0.001112836	0.787584748
HYAL3	-0.815450495	4.370707	-3.992214267	0.000109176	0.001124114	0.776145579
BASP1	1.380685279	4.435116186	3.989937473	0.000110113	0.001130765	0.768137924
BST1	0.953097856	4.817481794	3.989674457	0.000110222	0.001131284	0.767213106
SPATS2L	0.706646917	1.570300345	3.988590864	0.000110671	0.001134341	0.763403469
ACOT11	-0.750464634	3.701998296	-3.98699923	0.000111333	0.001138487	0.75780915
TTK	-0.599205916	3.496293569	-3.982092533	0.000113399	0.00115597	0.740573874
TTC39B	0.627863425	1.80721119	3.981107342	0.000113818	0.001159532	0.737115277
GPR183	0.831851454	5.881356274	3.974831521	0.000116524	0.001183482	0.715099106
APBB1	0.664866752	1.685131923	3.974495415	0.00011667	0.001183736	0.713920778
GIMAP2	0.635996457	5.417910554	3.973224935	0.000117226	0.001188136	0.709467395
KCNA3	1.006931544	3.645045155	3.971568109	0.000117955	0.001192415	0.703661431
SQOR	0.587323313	4.744048682	3.96983124	0.000118723	0.001197694	0.697576996
TRABD	0.596276358	6.323596706	3.967184499	0.000119903	0.001207721	0.688309174
HIST1H3E	0.792571936	1.422312517	3.964307421	0.000121199	0.001220139	0.678240262
ZNF66	-0.641838102	3.833187001	-3.961090108	0.000122663	0.00123297	0.666987374
KLF11	0.979828643	4.067129356	3.953354015	0.000126254	0.001265143	0.639958744
CTTNBP2NL	0.682396629	1.899796714	3.952256087	0.000126772	0.00126772	0.636126104
ACSM1	-1.225218471	3.342376946	-3.943870744	0.000130793	0.001302576	0.606882026
NLRC5	0.633993283	5.347379889	3.942337114	0.000131542	0.001309359	0.601538706
SH2B2	0.604762672	3.523484928	3.941617268	0.000131894	0.001312199	0.59903125
METTL7B	1.329206226	3.135184607	3.940688442	0.000132351	0.001314724	0.595796375
COCH	-0.872242371	3.131143279	-3.93917046	0.0001331	0.001320145	0.590510901
PADI2	1.1418644	2.976435652	3.937700772	0.000133829	0.001324677	0.5853951
ZNF714	-0.60927139	3.774302079	-3.936686378	0.000134335	0.001329005	0.581864987
TEC	-0.611881872	5.699855455	-3.934176734	0.000135593	0.001338733	0.573134438
TTYH2	0.596665464	3.886071104	3.930824934	0.000137292	0.001354128	0.561480986
FUCA1	0.631924772	4.563612851	3.927677842	0.000138905	0.001367019	0.550546334
VCL	0.627678046	5.852328408	3.925911748	0.000139818	0.001373476	0.544412999
CD79B	0.743659615	4.689142297	3.921085121	0.000142343	0.00139378	0.527661987
STK17B	0.588126792	6.587985643	3.9207728	0.000142508	0.00139378	0.526578619
ZBED3	-0.690507831	4.343554387	-3.918046723	0.000143954	0.001404573	0.517125379
NEIL3	-0.722425686	3.664156633	-3.917972509	0.000143994	0.001404573	0.516868099
SAMSN1	0.61363814	5.484405426	3.914382704	0.000145921	0.001420398	0.504427749
PTK2	1.05063189	1.83990499	3.913734647	0.000146271	0.00142251	0.502182885
TNS3	0.952912567	4.626499807	3.912936618	0.000146704	0.001424582	0.499418913

ASIC1	-0.733319867	1.040589693	-3.90982475	0.000148403	0.001439643	0.4886452
LPO	-1.495342081	3.69212961	-3.905149775	0.000150991	0.001458927	0.47247244
PTK6	0.617167536	2.341885982	3.900357787	0.000153688	0.001482043	0.455910646
CABLES1	-0.877158823	2.5999054	-3.898490603	0.000154751	0.001490818	0.449461718
TMIGD3	0.64919793	1.44600248	3.89279274	0.000158039	0.001517235	0.42979729
SPINT1	0.994678288	3.530351217	3.890057397	0.000159641	0.00153035	0.42036513
ULK2	0.709344336	2.719540466	3.88421819	0.000163112	0.001555965	0.400247502
EVI5	0.590493528	3.167106133	3.879806287	0.000165783	0.001578784	0.385063095
KLRF1	0.835453673	2.723356788	3.877572797	0.000167151	0.001589035	0.377381296
ARHGAP4	0.843837296	6.870642265	3.875085932	0.000168686	0.001598946	0.368832146
IFI44	0.735949444	5.361268398	3.871527145	0.000170907	0.001616847	0.356605544
PRF1	0.906320781	4.424196056	3.858273292	0.000179425	0.00168433	0.311148447
CATSPER1	0.606700521	1.665102469	3.853546047	0.00018256	0.001709985	0.294965067
HLA-DQB2	1.181015663	2.484444103	3.853489775	0.000182598	0.001709985	0.294772517
PLBD2	0.604413134	5.026580229	3.851096451	0.000184205	0.001723377	0.286585219
THBS1	1.433708369	3.197779175	3.848207999	0.000186162	0.001736682	0.276709495
TGFBR3	0.602187692	1.659459846	3.846829857	0.000187103	0.001741215	0.271999641
SLC27A2	-0.79318276	3.911035762	-3.846710234	0.000187185	0.001741215	0.271590889
DNM1	-1.049395273	4.057263856	-3.845523734	0.000187999	0.001745284	0.267537148
CCL3L1	0.94456538	2.414699514	3.842459287	0.000190117	0.001760348	0.257071875
SCML4	0.696585543	1.861848547	3.842417261	0.000190147	0.001760348	0.256928402
UGT3A2	-1.469712619	1.916854298	-3.835206362	0.000195221	0.001800476	0.23232901
TMEM63C	0.62515658	0.859533921	3.817307087	0.00020838	0.001900224	0.171425381
GAS7	0.838588927	5.46310221	3.815244179	0.00020995	0.001910549	0.16442071
TNNT1	1.014513178	1.50051106	3.815174152	0.000210003	0.001910549	0.164182983
GCA	0.597140135	5.784868002	3.812190739	0.000212294	0.001929583	0.154058168
GBP5	0.761258911	3.895349187	3.809453566	0.000214416	0.001943426	0.144774555
LGALS9	0.693130218	6.14920072	3.800956263	0.000221134	0.001993173	0.115988181
SH2D2A	0.645673063	2.551340453	3.79438596	0.000226464	0.002027137	0.093764971
TSPO	0.699173912	6.965112357	3.794057544	0.000226734	0.002028617	0.092654948
SELL	1.033474582	8.182077092	3.792177812	0.000228283	0.002039664	0.086303063
GAMT	-0.617482631	4.293350169	-3.788497215	0.000231345	0.002061348	0.073873054
QPRT	-0.711605525	3.746716093	-3.784812783	0.00023445	0.002080438	0.061439718
SERPINF2	0.602484911	2.309178666	3.783310919	0.000235727	0.002089862	0.056374357
LRP12	-0.629261102	2.314729506	-3.783178043	0.00023584	0.002089914	0.055926282
RTL5	0.813234609	2.718172531	3.780968686	0.000237731	0.002102843	0.048477863
PLAU	-0.752746964	3.820487481	-3.779610716	0.000238901	0.002112228	0.043901456
EMID1	-1.063499628	4.730993841	-3.777190143	0.000240999	0.002128845	0.035747284
FMNL2	0.684712489	2.221332519	3.775927689	0.000242101	0.002136634	0.031496116
SORBS3	0.805096044	3.553110195	3.76364349	0.000253069	0.002216345	-0.00981029
SLFN12L	0.627669119	2.794212902	3.760398593	0.000256044	0.002235934	-0.020703516
POU2AF1	0.703759321	2.303866004	3.758571897	0.000257733	0.002248089	-0.026832487
CD93	1.306484229	5.066013782	3.75806171	0.000258207	0.002251212	-0.028543855
SNX10	0.845758988	4.968440172	3.753929249	0.000262074	0.002279825	-0.042398897
CPM	1.01636445	2.211191747	3.752134783	0.000263771	0.002292728	-0.048411466
ZBTB38	0.673894154	2.782506436	3.75106017	0.000264792	0.002300379	-0.052010984
TMEM144	0.633240346	2.130255596	3.750920247	0.000264925	0.002300509	-0.052479608

LTB4R	0.608489888	5.596236737	3.748257177	0.000267472	0.002319525	-0.061395986
KIF1B	0.679999928	3.468558758	3.745988446	0.000269661	0.002336421	-0.068988058
TCF7	0.673714665	3.571056499	3.744535073	0.000271072	0.0023476	-0.073849686
LPAR4	-0.919970372	2.812324155	-3.742994612	0.000272575	0.002358518	-0.079000981
IL18RAP	0.758192338	2.178802968	3.739968741	0.00027555	0.002378975	-0.089114535
SEMA4A	0.785311711	5.010098683	3.738313816	0.000277191	0.002388897	-0.094643119
SCML1	0.653429133	1.643529228	3.736425924	0.000279073	0.002402993	-0.100947579
UBASH3A	0.714427568	2.18818968	3.732045718	0.000283488	0.002433469	-0.115565086
MARCKS	1.08948931	3.66690323	3.731823693	0.000283713	0.002434331	-0.116305654
GPAT3	1.090638076	3.127638741	3.730392615	0.000285171	0.00244253	-0.121078202
EVI2A	0.618267937	6.220863724	3.726277045	0.000289403	0.002473334	-0.134795161
MEX3B	-0.899647641	3.932182179	-3.714468874	0.000301877	0.002558595	-0.174083645
TREML1	0.815931246	1.807884525	3.712504356	0.000304002	0.002574357	-0.180610324
C15orf39	0.661869449	6.194019196	3.70854594	0.000308325	0.002601909	-0.19375286
ZNF117	-0.602881661	4.523526919	-3.700769859	0.000316988	0.00265887	-0.219537779
USP41	-0.708047463	1.479656364	-3.69990842	0.000317961	0.002663594	-0.222391568
CDCA2	-0.594691036	3.154647999	-3.698445348	0.000319622	0.00267635	-0.227237231
CBX2	-0.991349476	3.960311897	-3.696964563	0.00032131	0.002688177	-0.23213999
STS	0.763191231	3.004809289	3.695384032	0.000323122	0.002702088	-0.237371251
PRKCH	0.758020599	3.742416911	3.693496876	0.000325298	0.002716338	-0.243615026
RTP4	0.790374785	2.912683516	3.693045596	0.000325821	0.002717957	-0.245107736
MXD1	0.588326456	5.944898031	3.690888102	0.000328329	0.002730748	-0.252242089
HCST	0.705171207	6.63175077	3.687122719	0.000332751	0.002761335	-0.264685329
HAPLN3	0.62679679	1.910784106	3.686441459	0.000333557	0.002766844	-0.266935552
PLXNB1	-0.921571304	2.672848689	-3.683681084	0.000336842	0.00278815	-0.276049736
NKD1	-0.608264332	1.089040709	-3.671885695	0.000351226	0.002890006	-0.314933584
CXCR3	0.707771364	2.279373201	3.671644798	0.000351526	0.002890894	-0.31572666
PRRT4	-1.267678073	2.861537933	-3.671504635	0.000351701	0.002890894	-0.31618808
CDH24	-0.744037573	2.585694145	-3.669489551	0.000354219	0.002907252	-0.322820238
AFF2	-0.993027357	2.184508503	-3.663708353	0.000361538	0.002958274	-0.341831308
NDFIP1	0.809309094	5.279553457	3.658927847	0.000367698	0.003002685	-0.357533366
MMP25	0.791010254	3.091352037	3.655221352	0.000372542	0.003033334	-0.369696312
KIT	-1.037687174	6.506151086	-3.651939096	0.000376882	0.003062266	-0.380458778
CD40LG	0.654075399	2.298772453	3.648716256	0.00038119	0.003088244	-0.391018806
SCN3A	0.861163852	0.832290487	3.647296314	0.000383103	0.003100306	-0.395669022
GNPDA1	0.720377563	4.096552253	3.641996868	0.000390322	0.003149118	-0.413011405
RTN4R	-0.926525837	3.204676537	-3.640765522	0.000392018	0.003157563	-0.417038046
PEAK3	0.612412994	4.075506288	3.637285877	0.000396848	0.003184592	-0.428410916
SIX3	-1.216608791	0.922184357	-3.635326928	0.000399592	0.00319739	-0.434809661
CD180	1.098810368	4.719905431	3.634450452	0.000400825	0.003204314	-0.437671693
RASGRP1	0.628120297	3.265602154	3.632745952	0.000403234	0.003216767	-0.44323594
RUBCNL	0.843823359	2.384711312	3.631579283	0.000404891	0.003221287	-0.447043246
EGR3	0.942364483	2.223354785	3.631575832	0.000404896	0.003221287	-0.447054506
SUCLG2	0.883786139	3.960445568	3.629268072	0.000408192	0.003242208	-0.454582717
GABARAPL1	0.669863232	4.156290138	3.628311768	0.000409565	0.003251788	-0.457701167
TBC1D12	0.70403008	1.032193487	3.620725048	0.000420615	0.003328658	-0.482417348
NT5M	-0.594830242	4.115793437	-3.619940637	0.000421774	0.003336469	-0.484970421

TCN2	1.048440146	3.927236873	3.613695432	0.000431105	0.003399231	-0.505281057
PRELID3A	-0.692947453	2.497185674	-3.607266304	0.000440914	0.003456968	-0.526160051
P2RY12	0.66714406	1.396756718	3.598049266	0.000455346	0.003560078	-0.556040162
TNFRSF11A	0.636503289	1.094071037	3.596941402	0.00045711	0.003571003	-0.559627481
DEPTOR	-0.77821782	5.004020847	-3.594699142	0.000460701	0.003594725	-0.56688528
PF4	1.50661183	3.410909156	3.593588917	0.000462489	0.003603791	-0.570477517
AMIGO2	0.591159669	1.630772868	3.584695187	0.000477048	0.00368973	-0.599221346
GRAP2	0.779063744	2.724727125	3.581188186	0.000482907	0.003723208	-0.610539723
CARD11	0.793670889	3.940988013	3.57398414	0.000495157	0.003799438	-0.633761441
ZNF610	-0.585681624	2.332072247	-3.573881885	0.000495333	0.003799438	-0.634090778
SLC35F2	-0.590695232	3.834807048	-3.561567233	0.00051696	0.003929707	-0.673696736
C3AR1	0.99391892	5.340604467	3.55679161	0.000525585	0.003984371	-0.689025839
MLC1	-0.810703087	6.332371237	-3.555315108	0.000528279	0.004000932	-0.693761803
TCF7L2	0.68790108	4.224430143	3.545789376	0.000545973	0.004106932	-0.724277422
CARMIL1	0.698711949	1.908409503	3.537262863	0.000562283	0.004202011	-0.751535131
NR5A1	-0.628572018	1.107224275	-3.5338026	0.000569033	0.004239647	-0.762581607
PTGER4	0.668504523	5.577228157	3.533787359	0.000569063	0.004239647	-0.762630243
NELL2	0.585282877	1.48520292	3.530374954	0.000575794	0.004275064	-0.773515207
DYSF	1.032360853	4.462922695	3.529290117	0.000577949	0.004286162	-0.776973836
CLEC12A	0.762127957	6.355024102	3.5216792	0.000593287	0.004381539	-0.801214076
EPCAM	-1.018229148	1.649854596	-3.520990691	0.000594693	0.004388594	-0.803404809
LY6G6E	0.634957193	0.793315993	3.514484039	0.000608138	0.004460748	-0.824090609
NTNG2	-1.333994741	4.286235957	-3.513320024	0.000610573	0.004475237	-0.827787902
EPHB3	0.770661739	1.559184593	3.508217892	0.000621356	0.004532081	-0.843982056
PTP4A3	0.925776496	3.615518525	3.499956803	0.000639196	0.004639597	-0.870161648
ARID5B	0.653838046	3.54446396	3.493235434	0.000654066	0.004728127	-0.891424314
CLIC3	0.634186016	1.698721265	3.49168655	0.000657538	0.004746176	-0.896319345
MANSC1	0.675786784	1.859654071	3.488172443	0.000665481	0.004788362	-0.907418544
HBEGF	1.015091181	4.001547146	3.487692999	0.000666571	0.004790057	-0.908932141
CYSTM1	0.66189274	3.2275145	3.482700169	0.000678029	0.004849115	-0.924684199
SMARCD3	0.764645728	3.307489448	3.480775376	0.000682495	0.004872429	-0.930751824
RAB11FIP5	0.714652691	1.325299792	3.474562706	0.000697101	0.004945184	-0.950317449
BEND6	-0.816349297	1.326802914	-3.473540033	0.000699533	0.004957359	-0.953535396
GGT7	0.654070456	3.335034869	3.471483211	0.000704449	0.004978318	-0.96000503
EIF4E3	0.820511851	4.232426005	3.470888476	0.000705876	0.004984161	-0.961875151
ME1	0.898606762	1.68275917	3.469661521	0.00070883	0.004997763	-0.965732426
KIAA1958	-0.614283183	3.055285597	-3.462318066	0.000726751	0.005100099	-0.988795041
PTGIR	0.944135502	2.96723982	3.457837601	0.000737894	0.005168974	-1.002846392
IL1R2	1.124415608	1.392422779	3.45473038	0.000745715	0.005212505	-1.012582216
RAB7B	-1.050303797	4.760865621	-3.450693436	0.000755993	0.005271095	-1.025220311
PTGDR	0.625689925	1.661772557	3.450067862	0.000757598	0.005280391	-1.027177641
KLRC4	0.623512101	1.573667014	3.449442259	0.000759206	0.005289703	-1.029134772
BST2	0.603569582	7.070199666	3.448101455	0.000762662	0.005304293	-1.033328336
MMP15	-0.825380446	1.838019207	-3.440945662	0.000781362	0.005420789	-1.055686301
SPRY2	-0.961584728	4.097991068	-3.434435995	0.000798747	0.005515834	-1.075992016
PHGR1	-0.618785118	2.232867387	-3.433958007	0.000800038	0.00551968	-1.077481754
CRYBG3	0.783719996	2.563058645	3.432834443	0.00080308	0.005535929	-1.080982874

ATXN1	0.685576762	3.163401902	3.423986954	0.000827414	0.005659567	-1.108519154
MPZL2	0.651234361	1.095138801	3.423470307	0.000828856	0.005667439	-1.110125305
OPTN	0.742956672	3.244693529	3.423205122	0.000829597	0.005668523	-1.110949633
FOS	0.803165346	9.732916802	3.416532335	0.000848452	0.005777073	-1.131674535
DAGLA	0.75229349	2.025476757	3.40281972	0.000888468	0.005997084	-1.174158742
ST6GALNAC4	0.590847033	4.164345554	3.401032941	0.000893811	0.006018534	-1.179684041
KLRG1	0.598278629	3.289808363	3.398721683	0.000900767	0.006061175	-1.186827609
ANKRD18B	-1.085078867	1.795131628	-3.397118726	0.00090562	0.006089624	-1.191779609
FCRLA	0.673188602	1.939993212	3.396685482	0.000906936	0.006094262	-1.19311769
FAM151B	0.592843825	2.210438702	3.394404727	0.000913894	0.00612832	-1.2001595
TREML4	0.627865912	1.200027807	3.393531643	0.000916571	0.006142036	-1.202854095
ABCA9	0.588737991	0.950686221	3.392443403	0.000919918	0.006158101	-1.206211914
ADGRA3	-0.624180332	3.629538884	-3.391492372	0.000922852	0.006165018	-1.209145636
TOP2A	-0.683247275	5.738147062	-3.390634622	0.000925506	0.006180624	-1.211791019
TRPS1	0.994863048	2.871140871	3.388571103	0.000931919	0.006211844	-1.218152818
SGPP1	0.915606511	2.760898048	3.38803782	0.000933584	0.006219402	-1.219796395
MDFIC	0.859471477	4.598136091	3.38794643	0.000933869	0.006219402	-1.22007804
ZBTB8A	-0.641564772	2.504547824	-3.387629379	0.00093486	0.006223872	-1.221055065
CACNB1	0.61334684	2.781458024	3.386960048	0.000936956	0.00623569	-1.223117431
HSPA4L	-0.591381295	1.259430006	-3.384273315	0.000945411	0.006279081	-1.231392468
DDIT4	1.019541214	4.442803153	3.382883849	0.000949812	0.006304007	-1.235669821
GFI1	-0.879272674	5.958782307	-3.375553236	0.000973351	0.006431287	-1.258212216
LAG3	0.715322929	1.672892815	3.371055593	0.000988062	0.006515651	-1.27202272
ITGB5	0.612898105	1.690546027	3.370168008	0.00099099	0.00652832	-1.274746329
PKIB	0.698056352	1.289237565	3.369298182	0.000993867	0.006542843	-1.277414862
HDAC9	0.778491909	2.755337467	3.364772783	0.001008961	0.006637722	-1.291289012
AMN	-0.748454004	2.833381152	-3.364104661	0.001011208	0.006650254	-1.293336049
MRC2	-1.064737538	3.620030146	-3.362120172	0.001017908	0.006689799	-1.299414247
CXCR4	0.672656406	8.165035875	3.361857772	0.001018797	0.006693382	-1.300217716
TBX1	-0.70986741	1.741086836	-3.356404139	0.001037442	0.006799803	-1.316904858
TNFRSF10C	0.720294932	2.39528954	3.354146298	0.001045254	0.006837189	-1.323806809
GALM	0.590862688	2.890048393	3.352182028	0.001052095	0.006875004	-1.32980819
EDA2R	-0.834229121	2.526304305	-3.347911197	0.001067114	0.006956794	-1.342846581
STARD8	0.806008978	3.45278422	3.339601819	0.001096911	0.007126489	-1.368174321
GDF15	0.585462793	1.292156525	3.33464621	0.001115051	0.007206539	-1.383254353
COPZ2	0.588895826	1.381194266	3.334135864	0.001116935	0.007216318	-1.384806279
TLR2	0.834504722	6.243757747	3.33021909	0.001131493	0.007295842	-1.396710293
MYBPH	0.713974189	0.907683858	3.326399939	0.001145859	0.007376253	-1.408306297
ADCY4	0.615798127	2.367364681	3.321739152	0.001163622	0.00747145	-1.422442608
DLL3	-0.671532767	0.596299306	-3.316097748	0.001185466	0.007563663	-1.439530886
PDE4B	0.734158002	3.808307207	3.313798571	0.001194478	0.007611857	-1.44648828
RTN2	0.598348489	2.47485012	3.311625192	0.001203057	0.007646043	-1.453061276
STOX2	-0.658639821	0.80288242	-3.309597676	0.001211111	0.007679444	-1.459189868
NEURL1	0.631725274	1.82975821	3.309297813	0.001212306	0.007681193	-1.460095998
TMEM51	0.860537204	2.779503402	3.309231489	0.001212571	0.007681193	-1.460296407
AIFM3	0.712258552	2.59887262	3.309054743	0.001213276	0.00768316	-1.46083046
TSPOAP1	-0.815876867	5.985814107	-3.30857982	0.001215173	0.00769267	-1.46226536

CEP70	-0.632451529	3.708791317	-3.306377416	0.001224007	0.007723466	-1.468917285
SEMA3C	0.62768689	0.784981063	3.303753748	0.001234608	0.007782626	-1.476836695
RNASE1	0.909445902	2.32543554	3.302215494	0.001240864	0.00781463	-1.481477378
IRX5	-0.919365422	1.109250947	-3.298802716	0.001254848	0.007884837	-1.49176672
TC2N	0.777300226	2.809561511	3.293163844	0.001278277	0.007998479	-1.508747988
RAB38	-0.77366557	2.658579175	-3.292519533	0.00128098	0.008010028	-1.510686752
GNG11	0.881584481	1.512765121	3.292418106	0.001281406	0.008010028	-1.510991922
AHR	0.955231927	5.62403484	3.292331633	0.001281769	0.008010028	-1.511252089
PHLDA3	-0.587768026	2.073338517	-3.288502878	0.001297951	0.008090364	-1.522765849
DES	0.614010378	0.831164195	3.284687352	0.001314266	0.008176346	-1.534228591
HENMT1	-0.680688934	4.991676451	-3.283826853	0.001317971	0.008191544	-1.536812183
YES1	-0.633122838	3.352428673	-3.283691896	0.001318554	0.008192545	-1.537217329
ATP10A	0.99949229	2.886096562	3.282750058	0.001322623	0.008212583	-1.540044383
STARD4	0.652527472	3.59611447	3.279602465	0.001336306	0.008279057	-1.549487347
B3GALNT1	-0.588351393	3.494199047	-3.279299712	0.001337629	0.008282729	-1.550395223
EVL	0.598547384	4.814909547	3.271919325	0.001370264	0.008422407	-1.572505116
ST3GAL4	-0.64158634	4.944078384	-3.270511616	0.001376573	0.008453173	-1.576717505
CLEC11A	-1.264617103	7.955764675	-3.263370767	0.001408993	0.008611494	-1.59806199
NCR1	0.594506076	1.547804541	3.259396326	0.001427344	0.008696342	-1.60992479
NKG7	0.679938546	7.210191927	3.2570706	0.001438186	0.008745968	-1.616860881
SLC12A7	0.645929953	5.432549609	3.254698295	0.001449324	0.008797207	-1.623931573
PADI4	0.85826107	4.937899704	3.251623161	0.001463882	0.00885794	-1.633090574
GBP4	1.027781336	4.302520466	3.248343167	0.001479559	0.00893336	-1.642851655
LGMN	0.718010717	2.308126431	3.247590735	0.001483177	0.008952006	-1.645089677
PDZD8	-0.622106206	4.840937365	-3.247119051	0.00148545	0.008960588	-1.646492421
AC099489.1	-0.989534552	2.892580398	-3.245365205	0.001493928	0.009000571	-1.651706684
C11orf21	0.747741462	5.953283649	3.242480601	0.001507971	0.009071133	-1.660277557
VENTX	1.076488727	2.165945165	3.240638756	0.001517002	0.009097335	-1.665746756
DUSP5	0.629141969	3.618618852	3.238497739	0.001527563	0.00914939	-1.672101013
CAVIN3	0.68803319	0.941463796	3.235330389	0.001543312	0.009221166	-1.681494766
HOXB2	1.181675033	3.181611844	3.235289378	0.001543517	0.009221166	-1.681616345
LDOC1	0.804948669	1.469651518	3.233573782	0.001552111	0.009253702	-1.686701166
CENPF	-0.596257529	4.571072433	-3.221501104	0.001613864	0.009561726	-1.722418456
TGM5	-0.745673163	1.127525146	-3.218018596	0.001632097	0.009653682	-1.732700465
GCAT	-0.604689012	3.117231465	-3.217029511	0.001637311	0.009670463	-1.735618988
MT1E	0.74914553	1.58692208	3.216389851	0.001640691	0.009683908	-1.73750605
KLHDC7B	0.585791043	2.903306329	3.215035294	0.00164787	0.009708612	-1.741501073
RUSC2	0.629661274	4.481779974	3.193252507	0.001767405	0.01027905	-1.805549018
ZBED2	-0.877401423	3.000289049	-3.190708855	0.001781878	0.010347759	-1.813003965
SAMD11	-0.759068191	1.983054079	-3.18730345	0.001801426	0.010439473	-1.822976622
DUSP2	0.618154798	4.937634443	3.182929098	0.001826829	0.010555253	-1.835773509
RHOBTB1	-0.728307641	2.899972978	-3.181745819	0.001833758	0.010585856	-1.839232544
TBC1D16	-0.64010911	2.497811255	-3.180291497	0.001842307	0.010622604	-1.843482406
MOB3B	0.590976477	2.714334801	3.175695521	0.001869568	0.010757478	-1.856902015
ALS2	-0.612869831	4.445526631	-3.175356601	0.001871593	0.010765947	-1.85789096
NID1	1.150879433	3.717340521	3.174884571	0.001874417	0.010779005	-1.859268158
AKAP7	0.654276675	2.953978147	3.172083496	0.001891255	0.010846988	-1.86743701

TPPP3	0.943372098	2.138262478	3.171230318	0.001896412	0.010866955	-1.869923935
CDCA7	-0.737234253	5.705821933	-3.168799046	0.001911179	0.010932257	-1.877007709
BLNK	1.041275588	3.212667832	3.166370465	0.001926036	0.010991393	-1.884079012
MDFI	-1.07986887	2.764564686	-3.16384759	0.001941583	0.011041263	-1.89141997
CLEC2B	0.778656252	5.137752506	3.160996404	0.001959294	0.011119234	-1.899710222
RTL8C	1.053265453	4.119097256	3.156363116	0.001988395	0.011237996	-1.913168586
TJP2	0.649906083	3.299970732	3.155743914	0.001992314	0.011247586	-1.914965913
HIP1	0.684846262	4.81977791	3.135877859	0.002121933	0.011852127	-1.972470041
SLC7A5	-0.683848917	5.700666571	-3.133048388	0.002141018	0.011941591	-1.980634921
TMEM268	0.591285998	4.115626698	3.111807886	0.002289453	0.012635493	-2.041725992
DOCK10	0.586654214	4.764423056	3.105663942	0.002334134	0.01280582	-2.059330476
KCNE5	-1.502649098	2.516532709	-3.095778861	0.00240772	0.013131794	-2.08759186
CCL5	0.670028309	5.873769884	3.093399549	0.002425751	0.01320792	-2.094382739
SPTLC3	-0.602709446	1.678293324	-3.092714841	0.002430962	0.013228894	-2.096336155
BIVM	-0.619636702	3.378769274	-3.092029935	0.002436186	0.01324991	-2.098289766
ZNF804A	-0.763892351	2.996961914	-3.090099814	0.002450962	0.013305136	-2.103793197
MSRB3	-0.643577996	5.761998138	-3.069305933	0.002615502	0.014056245	-2.162896021
CAMK1D	0.704574381	3.96683126	3.068175941	0.002624729	0.01409805	-2.16609797
ZAP70	0.79274174	3.982326633	3.063417357	0.00266392	0.014274157	-2.179570761
SERPINB6	0.808975952	5.624853555	3.055779765	0.002727954	0.014520239	-2.201157069
TLR10	0.594142938	2.241542677	3.054854996	0.002735804	0.014550079	-2.203767611
PTGR1	-0.599976612	2.188644271	-3.051489161	0.002764548	0.014682891	-2.213263309
CPNE8	1.192218283	3.388980725	3.037880686	0.002883635	0.015203344	-2.251563377
HIST1H1C	0.666719315	5.0569196	3.029769159	0.002956854	0.015517901	-2.274322294
SMIM24	-0.841283944	6.145034541	-3.028653949	0.002967053	0.015560432	-2.277447181
DOC2A	-0.613107795	2.158506533	-3.027735892	0.002975474	0.015582005	-2.280018885
VAV2	0.609252385	3.616443724	3.026459605	0.002987218	0.015626681	-2.283592957
MILR1	0.608443315	5.375234278	3.021201332	0.003036052	0.01581836	-2.298304301
RGS10	0.678077587	5.368390318	3.016961825	0.00307596	0.015979229	-2.310149264
RHOU	0.750352359	3.456651518	3.007739076	0.003164455	0.016360334	-2.335867366
PPBP	1.397811592	4.451061477	3.006358586	0.003177901	0.016416767	-2.339711062
RTL8A	0.861693684	3.934289858	3.004673757	0.003194383	0.016484408	-2.344400053
CXorf21	0.616111634	6.084469411	3.00349539	0.003205958	0.016530986	-2.347678172
LPAR1	0.683319141	1.597047974	3.0030382	0.003210459	0.016543912	-2.348949737
PLPPR3	-1.056131004	5.346998307	-2.999497458	0.003245516	0.016677511	-2.358791778
CIITA	0.889901418	5.634339601	2.989427367	0.003347157	0.017095977	-2.386728069
LMO4	0.656261416	4.166863596	2.986772982	0.003374433	0.017180323	-2.394078238
MEX3A	-0.655804059	1.82568986	-2.986409319	0.003378186	0.017191289	-2.395084803
NPW	-0.835481441	3.642083622	-2.985515266	0.003387428	0.017220311	-2.397558956
LONRF3	0.658932467	2.665267586	2.981558306	0.003428615	0.017376599	-2.408501505
CALCRL	0.873124701	2.206865378	2.976319915	0.003483848	0.017600127	-2.422968311
SETD7	0.588216932	3.020349159	2.974137859	0.003507097	0.017703789	-2.428987945
ALDH2	0.738419666	2.725193719	2.96911351	0.003561172	0.017916342	-2.442834006
ZBTB16	-0.720786654	4.115980247	-2.968115658	0.003572003	0.017956903	-2.445581454
LCN2	0.797547684	1.512563973	2.967272346	0.003581179	0.017993739	-2.447902773
SUCNR1	-0.873825809	4.773387365	-2.964438389	0.003612177	0.01810741	-2.455699374
AIF1L	-1.010004667	3.062323124	-2.954544047	0.003722346	0.018521315	-2.482869186

KRT72	0.647316711	1.312842281	2.952740346	0.00374276	0.018603858	-2.487813614
BPI	0.988023148	2.310542449	2.951758845	0.003753911	0.018654523	-2.490503065
HLA-DQA2	1.187031181	3.206769858	2.948261535	0.003793895	0.018814778	-2.500079852
CD38	-0.641413744	6.205602214	-2.944390909	0.003838602	0.018964002	-2.510667362
GYG2	-0.612782543	1.3142317	-2.93703881	0.003924859	0.019282443	-2.530744504
WFS1	0.635956986	1.488093795	2.936510519	0.003931125	0.01930348	-2.532185476
SNX9	0.633858079	3.289884185	2.935369154	0.003944694	0.019355459	-2.535297903
LDLRAD3	1.006083708	2.518757348	2.933994838	0.00396109	0.019415788	-2.539044173
ITPR3	0.68858947	3.122575953	2.933540494	0.003966524	0.019428281	-2.54028234
TMEM170B	0.679987226	4.776909936	2.933139825	0.003971322	0.019446886	-2.541374092
CD3D	0.800721718	4.307404221	2.932284791	0.003981578	0.019482403	-2.543703477
GADD45B	0.612635584	4.600848722	2.919807775	0.00413404	0.02004695	-2.57762734
CD96	-1.103337954	5.686555911	-2.917909742	0.004157696	0.020144925	-2.582776842
CLEC4D	0.957845373	2.054053618	2.916510079	0.004175221	0.020201344	-2.586572352
ENG	0.606948554	5.634579802	2.912292513	0.004228439	0.020392916	-2.597999633
BLK	0.597625172	2.240274317	2.896017935	0.004439679	0.021165151	-2.641959087
AEBP1	-0.761968985	3.86911051	-2.895188201	0.004450703	0.021212511	-2.644194513
IFITM1	0.79189792	5.395516095	2.89308931	0.004478703	0.021319854	-2.649846726
PRKCA	0.696239939	3.400145147	2.892257162	0.004489849	0.02136246	-2.652086666
TAFA5	-0.601433622	0.629435339	-2.889769759	0.004523317	0.021484927	-2.658778788
PGGHG	0.606956501	4.976668131	2.888456486	0.004541079	0.02153524	-2.662309987
ADAMTSL4	0.671002367	5.039732256	2.888314953	0.004542997	0.021535652	-2.662690465
ATP8B4	-0.606012941	6.143161683	-2.887240461	0.004557583	0.021578859	-2.665578445
C2	0.743124375	1.535116029	2.88390753	0.004603099	0.021747102	-2.674530586
TP53I11	0.678446762	3.766295208	2.875890532	0.004714295	0.022143418	-2.696026821
PALM	-0.87531948	2.882472177	-2.872153269	0.004766967	0.022336907	-2.70602972
LCN6	0.743478726	0.877994325	2.86315369	0.004896022	0.022853583	-2.730070491
PANX2	0.613871444	2.406476544	2.862265603	0.004908929	0.022902844	-2.732439265
IRS2	0.647079313	5.226814839	2.859500685	0.004949313	0.023025013	-2.73980993
ADAMTS10	0.814751378	3.083585424	2.85274596	0.005049253	0.02342834	-2.757790207
FCRL1	0.668021138	3.029152373	2.849914997	0.005091684	0.023585855	-2.765314777
CD79A	0.617522512	4.780356134	2.844280256	0.005177112	0.023901923	-2.780272091
KCTD15	-0.608097983	3.55893941	-2.843706223	0.005185888	0.023931085	-2.781794388
CPXM1	-0.696649858	6.970110228	-2.836906376	0.005290886	0.024334825	-2.799806553
COL23A1	-1.091889578	3.257935805	-2.833644344	0.005341942	0.02450594	-2.808433895
CXCR2	0.653686681	2.611827913	2.830252454	0.005395508	0.024710892	-2.817395408
LY6E	0.599227977	6.509382802	2.828475202	0.00542377	0.024805303	-2.822087203
MEST	-0.931783018	5.134202781	-2.81709691	0.005607937	0.025509688	-2.85206336
TCL1A	0.681306576	2.090063177	2.814544666	0.005650023	0.025659146	-2.858772623
HNRNPLL	0.69445088	2.904487968	2.813447908	0.005668196	0.025723671	-2.861654098
GPR153	-0.764622657	2.280817935	-2.811491579	0.005700746	0.025852346	-2.866791433
DPPA4	1.006944	1.547013272	2.806766536	0.005780066	0.02613385	-2.879186426
PLCH1	-0.648718828	1.413101817	-2.805321551	0.005804524	0.026238336	-2.882973312
ITGB3	0.872866699	1.984805458	2.802536867	0.005851923	0.026403531	-2.890266305
IGLL1	-1.239598227	6.01328752	-2.791837853	0.006037338	0.027036488	-2.918227115
TUBB1	0.639094205	2.718755031	2.771195919	0.006410268	0.028337987	-2.971905347
STK32B	-0.773367741	2.871828833	-2.76329717	0.006558434	0.028825095	-2.992352272

GCSAML	-0.961373144	3.751802647	-2.760605034	0.006609641	0.029008483	-2.999309389
PLCG1	0.611141489	3.462117211	2.760173057	0.006617891	0.029016941	-3.00042516
SHANK1	-0.946353588	2.854212811	-2.753619298	0.006744217	0.02947944	-3.017334136
GAS2L1	0.642661377	2.647977163	2.747576028	0.006862642	0.029882975	-3.032894421
TBC1D4	0.595983605	2.612872315	2.738036285	0.007053435	0.030557565	-3.057395682
B4GALT6	-0.703239024	4.68192712	-2.73622202	0.007090261	0.030681894	-3.062046758
CPNE3	0.591231775	4.862912629	2.728556108	0.007247798	0.031252415	-3.081668941
AGRN	-0.66888326	3.325018785	-2.726832485	0.007283654	0.031372267	-3.086074103
HRH2	0.745800613	4.929201733	2.722553725	0.00737336	0.031667531	-3.096998872
MS4A3	-1.229352354	5.554812898	-2.719624002	0.00743536	0.031858942	-3.104470402
UGT2B28	-0.602064448	1.216189243	-2.717387088	0.007483016	0.032025401	-3.110170274
POU4F1	-1.044891594	0.93443827	-2.716427135	0.007503553	0.032070972	-3.112615048
AK5	0.678994405	1.976428208	2.712492664	0.007588258	0.032383223	-3.122627178
SERPINB2	0.958080088	2.753633584	2.702814822	0.007800314	0.033148701	-3.147199584
RAB44	-0.61128365	6.146553219	-2.688076161	0.008133592	0.034222638	-3.184471178
CPA3	-1.273556785	6.534279463	-2.68635658	0.008173305	0.034345175	-3.188807865
PACSIN1	0.808248813	1.375613255	2.682299245	0.008267702	0.034606105	-3.199030424
MMP2	-1.061077149	3.786392557	-2.682235752	0.008269187	0.034606105	-3.199190286
GSDMC	-0.614546179	1.26450319	-2.674273582	0.008457339	0.035279704	-3.219210648
NFIL3	0.604258784	5.427649714	2.666424131	0.00864661	0.035907604	-3.23889553
PPP1R16B	0.596789615	3.699873765	2.665624601	0.008666102	0.035975909	-3.240897691
EPX	-0.790194182	2.562024296	-2.661255822	0.008773314	0.036325254	-3.251828393
JCHAIN	0.783421261	6.790014499	2.661040989	0.008778617	0.036339478	-3.252365491
PEAR1	0.854767315	2.349792549	2.65668729	0.008886709	0.036677701	-3.263241723
NECTIN1	-0.608455394	3.983931143	-2.634376801	0.009459727	0.03844743	-3.318726638
SLC41A1	0.633456912	2.630183601	2.631444172	0.009537477	0.038698772	-3.325988741
IL1B	0.642668653	5.152473789	2.628651479	0.009612053	0.038946413	-3.332897579
SERINC2	0.709833677	1.985150598	2.622876395	0.00976794	0.039412007	-3.347163676
ERG	-0.671550861	5.243629013	-2.621657745	0.009801125	0.039505881	-3.35017049
BAHCC1	-0.753926981	5.7857261	-2.612330368	0.010058501	0.040293668	-3.373142694
COBLL1	0.667460448	1.981388628	2.611400438	0.010084493	0.04036286	-3.375428969
HNMT	0.837076357	2.276150172	2.607188078	0.010202986	0.040728225	-3.385776093
ASS1	-0.732744901	1.255468962	-2.595505898	0.010538191	0.041732439	-3.414393327
ITM2A	-0.947999318	6.526220397	-2.594524006	0.010566812	0.041802387	-3.416793354
BEX2	0.748533515	1.904992101	2.593584703	0.010594257	0.041886274	-3.419088516
NMUR1	0.63093558	1.951157498	2.592185929	0.010635246	0.042005658	-3.422504999
RHOBTB3	-0.628186504	3.389606775	-2.584601498	0.010859988	0.04265954	-3.441000977
DEFA3	0.934548318	1.616913272	2.582893088	0.010911197	0.042826118	-3.445160505
MPO	-1.441239791	10.65714015	-2.577273405	0.011081181	0.043414493	-3.45882549
SPRED1	0.6000151	1.251202423	2.575775886	0.011126878	0.043550977	-3.462462378
IGF1R	-0.675254705	3.494910287	-2.575574251	0.011133044	0.043556298	-3.462951924
S100B	-0.959268491	3.067827632	-2.573012879	0.011211635	0.043810929	-3.469167642
KRT17	-0.737465802	1.295251211	-2.566756578	0.0114057	0.044376616	-3.484326471
UNCX	-0.771673074	0.81513349	-2.561525365	0.011570278	0.044950609	-3.496976004
PLXDC2	0.650328421	4.021922973	2.560701545	0.011596389	0.045016104	-3.498965953
ADRA2C	-0.654847955	0.935869611	-2.560361505	0.011607182	0.045040032	-3.499787157
LPAR6	0.640587646	4.094487669	2.556083259	0.011743749	0.045443102	-3.5101108

ITM2C	-0.641524706	7.346265919	-2.554391865	0.011798137	0.04558105	-3.514187929
SIRPB2	0.688689398	5.355715365	2.553118805	0.011839222	0.045712552	-3.517255052
CAVIN2	0.863022594	3.2196466	2.55254098	0.011857912	0.045766556	-3.518646725
IRX1	-0.823479201	1.24955302	-2.547636722	0.012017614	0.046208806	-3.530447015
EPB41L2	0.745343093	3.406046479	2.534398616	0.01245837	0.04750897	-3.562197314
PLA2G4A	0.614409066	4.231244421	2.529517225	0.012624514	0.04799272	-3.573867134
DLC1	-0.668142774	2.652653113	-2.527242488	0.012702612	0.048242162	-3.579298343
ARPP21	-0.679202333	0.984070109	-2.525764697	0.012753581	0.048379081	-3.582824379
DYTN	-0.630629497	2.507354925	-2.516121404	0.013090695	0.049374636	-3.605787647
COL2A1	-0.619495981	0.759519545	-2.512452769	0.013221023	0.049764238	-3.614502777

Stromal_limma_res

	logFC	AveExpr	t	P.Value	adj.P.Val	B
CPN2	0.643942078	0.182642479	9.64627733	6.50E-17	1.27E-12	27.83614363
PCDH18	1.335572043	0.4846745	9.521486777	1.32E-16	1.28E-12	27.15512695
SMOC2	0.836623468	0.277971873	9.170060321	9.55E-16	4.33E-12	25.24483154
FRZB	1.658392646	0.575646903	9.116435972	1.29E-15	4.33E-12	24.95445385
SEPTIN10	1.304085976	0.660896788	9.110275712	1.34E-15	4.33E-12	24.92111659
YAP1	0.759202437	0.289602127	9.073761076	1.64E-15	4.56E-12	24.72360145
PARVA	0.746863134	0.285278905	8.81297292	7.04E-15	1.25E-11	23.31771176
SNAI2	1.579162172	0.6572711	8.690094892	1.39E-14	2.26E-11	22.6584429
TMEM132C	0.857376687	0.302389472	8.67266747	1.54E-14	2.26E-11	22.56511765
GPX8	0.803120075	0.305287626	8.662616049	1.62E-14	2.26E-11	22.51131202
SLC22A3	1.599385567	0.717680842	8.629863187	1.95E-14	2.53E-11	22.33609021
FNDC4	0.585734271	0.226974678	8.609969676	2.18E-14	2.65E-11	22.22974324
C7	2.100037769	1.00411685	8.531918061	3.35E-14	3.84E-11	21.81308954
ID4	0.971624399	0.354185612	8.467710671	4.78E-14	4.89E-11	21.47107118
FMO3	1.518144003	0.719794097	8.454843134	5.13E-14	4.99E-11	21.40261039
SVEP1	1.114190114	0.367460604	8.44163131	5.52E-14	5.11E-11	21.33234667
SIX2	0.759624527	0.322344084	8.39251128	7.23E-14	5.86E-11	21.07137379
SPARCL1	1.862465069	1.152432216	8.369070042	8.23E-14	6.33E-11	20.94697761
BMP5	1.251954964	0.482324564	8.364094691	8.45E-14	6.33E-11	20.92058711
BCAR1	0.585510439	0.27074052	8.269203807	1.42E-13	1.03E-10	20.41810479
LAMA4	1.099150458	0.445250038	8.250976023	1.57E-13	1.09E-10	20.32176883
COL1A2	2.225972158	1.092954969	8.231222149	1.75E-13	1.18E-10	20.21743679
ISLR	1.448663236	0.639371061	8.216809967	1.90E-13	1.23E-10	20.14136342
CHL1	1.393226107	0.656610451	8.128390653	3.08E-13	1.93E-10	19.67551606
FMO2	1.433464964	0.67284308	8.041982352	4.92E-13	2.90E-10	19.22174583
LGI4	0.677020472	0.566242625	8.005296466	6.01E-13	3.34E-10	19.02954873
COL1A1	2.293446337	1.218545005	7.979234084	6.92E-13	3.74E-10	18.89317757
CDH11	1.383570475	0.547531165	7.919817771	9.55E-13	4.78E-10	18.58281832
SEMA6D	0.920711389	0.357468223	7.914860427	9.81E-13	4.78E-10	18.55695792
FERMT2	0.802368419	0.471272518	7.914435618	9.83E-13	4.78E-10	18.55474211
HTRA1	1.379523022	0.656712965	7.896535985	1.08E-12	5.14E-10	18.46141311
TUSC3	0.662226976	0.226794085	7.876287163	1.21E-12	5.35E-10	18.35591957
PAPPA	1.120458936	0.364631957	7.871066956	1.24E-12	5.38E-10	18.3287376

ADGRF5	0.905793691	0.335762486	7.834339161	1.52E-12	6.15E-10	18.1376639
TMEM108	0.721326638	0.313997005	7.822040059	1.62E-12	6.30E-10	18.07374594
ABCA8	1.028915162	0.337041671	7.816420643	1.67E-12	6.37E-10	18.04455337
IL10	2.152093609	1.073275893	7.794786611	1.88E-12	7.02E-10	17.93223233
CCDC80	1.159058125	1.028582526	7.788538795	1.94E-12	7.12E-10	17.89981427
COL3A1	2.267252709	1.117463316	7.782986774	2.00E-12	7.20E-10	17.87101396
TENM4	0.870491678	0.387058	7.765460698	2.20E-12	7.77E-10	17.78014629
SLIT3	0.623181609	0.182873524	7.743145118	2.48E-12	8.61E-10	17.66454893
DDR2	1.127638402	0.691875218	7.731429148	2.64E-12	9.00E-10	17.60390511
COL5A3	1.208073185	0.745708457	7.722471549	2.77E-12	9.05E-10	17.55756071
EFEMP1	1.385639131	0.482218468	7.7208509	2.79E-12	9.05E-10	17.54917789
GHR	0.832818563	0.368487572	7.688572854	3.32E-12	1.06E-09	17.38234828
BGN	2.493916539	1.548048681	7.6036644	5.23E-12	1.61E-09	16.94468994
GNG12	1.082326316	0.500029319	7.57307192	6.15E-12	1.84E-09	16.78743387
OSR2	0.648758981	0.329975508	7.455555659	1.15E-11	3.20E-09	16.18555493
LTBP2	1.366971176	1.296697775	7.442505222	1.23E-11	3.38E-09	16.11893456
GEM	0.651295674	0.347279372	7.40507331	1.50E-11	4.00E-09	15.92809961
COL16A1	0.813426094	0.375795109	7.388807853	1.64E-11	4.30E-09	15.84529128
FMOD	1.395979138	0.677237242	7.375037576	1.76E-11	4.57E-09	15.77524148
ADAMTS9	0.961528269	0.383868599	7.372180582	1.79E-11	4.57E-09	15.76071427
NR2F1	0.691028156	0.355127952	7.359876108	1.91E-11	4.82E-09	15.69817379
PDGFRA	0.748777473	0.372547653	7.324121865	2.30E-11	5.74E-09	15.51667778
CDH5	1.25972191	0.694094219	7.307091417	2.52E-11	6.12E-09	15.43035091
ESM1	1.429212741	0.646185102	7.301002535	2.60E-11	6.25E-09	15.39950598
RGL1	1.68032545	1.362122904	7.263233568	3.17E-11	7.52E-09	15.20840725
RND3	1.077007315	0.299593314	7.238425414	3.61E-11	8.30E-09	15.08310395
PLPP3	1.662214325	1.399651331	7.23669142	3.64E-11	8.30E-09	15.07435225
SELENOM	0.923601601	0.836222617	7.235440232	3.67E-11	8.30E-09	15.06803786
RGL3	0.982620472	0.445637836	7.231155783	3.75E-11	8.39E-09	15.04641885
HID1	1.291031556	0.989967612	7.221434803	3.95E-11	8.71E-09	14.99738689
NR2F2	0.893053142	0.366776516	7.204987598	4.30E-11	9.30E-09	14.91448982
VCAM1	2.408094847	1.365706792	7.175161913	5.03E-11	1.08E-08	14.76436126
HEY2	0.662908844	0.214229263	7.167278551	5.24E-11	1.10E-08	14.72472316
FZD4	0.842069678	0.482175763	7.163462146	5.35E-11	1.11E-08	14.70554051
FOLR2	2.096742241	1.528494562	7.161562897	5.40E-11	1.11E-08	14.69599577
THY1	1.505780345	0.732422099	7.156241395	5.55E-11	1.13E-08	14.669258
IBSP	0.735527104	0.160435782	7.149212305	5.76E-11	1.16E-08	14.63395318
CP	1.549929714	1.923620778	7.11830415	6.77E-11	1.32E-08	14.47888409
PLEKHS1	0.739226108	0.353316949	7.10737346	7.16E-11	1.38E-08	14.42411145
TJP1	0.659102538	0.309865308	7.092859112	7.72E-11	1.45E-08	14.35143645
ANTXR1	0.91796542	0.53059076	7.092815255	7.73E-11	1.45E-08	14.35121695
APOE	2.500260104	2.687095697	7.077582323	8.36E-11	1.55E-08	14.27501193
TMEM119	1.200128527	0.890858368	7.056834705	9.31E-11	1.69E-08	14.17133129
SLCO2B1	1.259851554	0.535770179	7.036885972	1.03E-10	1.85E-08	14.07176591
SOWAHC	0.864019223	0.334950618	7.034434725	1.05E-10	1.85E-08	14.05953995
TNC	1.94065133	1.756902059	7.033427881	1.05E-10	1.85E-08	14.0545187
MXRA5	1.285277526	0.545730063	7.012093259	1.17E-10	1.99E-08	13.94819338

NGFR	1.57913618	1.130558933	6.997779533	1.26E-10	2.10E-08	13.87693662
MYO6	0.828116373	0.673634159	6.961387753	1.53E-10	2.48E-08	13.696057
ALDH1A3	1.083422351	0.884376724	6.956678512	1.56E-10	2.50E-08	13.67268069
WNT10A	0.922256638	0.800212416	6.956399713	1.57E-10	2.50E-08	13.67129698
DCN	1.295158168	0.673059049	6.94691899	1.64E-10	2.56E-08	13.62425737
LIFR	1.358710889	0.708662011	6.930275455	1.79E-10	2.77E-08	13.5417473
CCL14	0.818495804	0.38366728	6.904514233	2.05E-10	3.11E-08	13.41420975
C1S	1.471384834	1.477919308	6.877153006	2.35E-10	3.55E-08	13.27898345
PARM1	1.315163966	1.25014906	6.873822353	2.40E-10	3.59E-08	13.26253895
COL6A3	1.530608727	0.830792816	6.84349317	2.80E-10	4.16E-08	13.11295958
ANGPTL2	1.062401902	0.792308605	6.829247371	3.01E-10	4.44E-08	13.04280475
HFE	1.229491628	0.972314187	6.801577902	3.47E-10	5.04E-08	12.90673435
MOCS1	1.013773198	0.526997174	6.77805482	3.91E-10	5.64E-08	12.79125403
SEMA3C	1.712070584	0.784981063	6.76278313	4.23E-10	5.96E-08	12.71638054
RHOJ	0.613204524	0.233903423	6.757479703	4.34E-10	6.04E-08	12.69039739
MAOB	0.892688546	0.322039458	6.709544434	5.54E-10	7.54E-08	12.45597844
C1QTNF1	1.052211447	0.532750166	6.694211698	5.99E-10	8.10E-08	12.38116151
CCL4	1.602409472	2.534537136	6.644337098	7.71E-10	1.03E-07	12.13835607
RSPO1	0.745482893	0.402322142	6.640269946	7.87E-10	1.04E-07	12.11859398
GPNMB	1.260344916	0.812348611	6.637414484	7.99E-10	1.05E-07	12.10472288
LDB2	0.752350278	0.386440738	6.612803621	9.05E-10	1.17E-07	11.98528814
COL4A1	1.464522567	0.701871152	6.603574544	9.48E-10	1.21E-07	11.94055507
CADPS2	0.672070643	0.259955297	6.594604731	9.92E-10	1.26E-07	11.89710751
MMRN2	0.921451246	0.559187268	6.572224912	1.11E-09	1.39E-07	11.7888298
FABP4	2.071535985	1.292203332	6.569623365	1.12E-09	1.40E-07	11.77625461
CCL8	0.724525952	0.220248164	6.560556005	1.18E-09	1.46E-07	11.73244435
CALD1	1.351123511	0.993647265	6.54752329	1.26E-09	1.54E-07	11.66952657
LBP	2.049180055	1.065092764	6.539507212	1.31E-09	1.59E-07	11.63085787
PODN	0.880762938	0.562949918	6.526213378	1.40E-09	1.68E-07	11.566781
ALPL	2.053080596	1.565841468	6.515439296	1.48E-09	1.76E-07	11.51489645
EPAS1	1.840122676	1.79317429	6.51497502	1.48E-09	1.76E-07	11.5126616
C4B	0.627766444	0.425229796	6.513485234	1.49E-09	1.76E-07	11.50549083
RNASE1	2.393821842	2.32543554	6.444547977	2.10E-09	2.44E-07	11.17456626
SDC1	2.01308465	1.978761126	6.432088373	2.24E-09	2.58E-07	11.11494306
AFAP1	1.251329358	0.934900069	6.397727227	2.66E-09	2.97E-07	10.95081507
DNASE1L3	1.666833723	1.096796035	6.397577905	2.66E-09	2.97E-07	10.9501028
LYVE1	1.625876381	0.900147666	6.393153589	2.72E-09	3.01E-07	10.92900233
COL15A1	0.903992063	0.378881087	6.392836686	2.72E-09	3.01E-07	10.92749125
TF	1.487531044	1.097532538	6.387026778	2.80E-09	3.06E-07	10.89979456
AMPD1	1.441611966	1.023999641	6.36900191	3.06E-09	3.29E-07	10.81394881
FLT1	0.730564519	0.488615589	6.351768775	3.34E-09	3.57E-07	10.7319895
ERRFI1	0.94095434	1.247912271	6.344507798	3.46E-09	3.66E-07	10.69749094
C1R	1.281920769	1.838622387	6.335244725	3.62E-09	3.81E-07	10.65350933
LEPR	1.594949144	2.726024389	6.32965181	3.72E-09	3.90E-07	10.62696983
NLGN4X	0.653310514	0.212714687	6.325555372	3.80E-09	3.95E-07	10.60753906
VEGFC	1.219980301	0.759935028	6.304738309	4.21E-09	4.36E-07	10.50889708
ADGRL4	1.148844499	0.562676554	6.287018539	4.60E-09	4.73E-07	10.42506425

WASF3	1.123089316	0.631888357	6.203580193	6.92E-09	6.90E-07	10.03197391
SDC3	1.948515961	1.507383271	6.201944826	6.98E-09	6.90E-07	10.02429708
KDR	1.003325782	0.535929512	6.18696759	7.51E-09	7.38E-07	9.954040109
IL33	1.017230084	0.481749391	6.154071306	8.82E-09	8.54E-07	9.800043854
FSTL1	2.09838694	1.760206873	6.152059417	8.91E-09	8.58E-07	9.790639898
PLEKHH2	0.767695957	0.464737562	6.124855082	1.02E-08	9.70E-07	9.663643579
AOC3	0.856256302	0.904160937	6.119427527	1.04E-08	9.91E-07	9.638342683
COL12A1	0.619405269	0.240811926	6.087976257	1.22E-08	1.14E-06	9.491969297
KCNN3	0.611980715	0.41123592	6.082531725	1.25E-08	1.16E-06	9.466672109
C4A	0.61591248	0.581528227	6.034673934	1.57E-08	1.44E-06	9.244839696
PRSS23	0.782117306	0.562815527	6.031942702	1.59E-08	1.46E-06	9.232208721
CXCL12	3.721332779	3.402228626	6.019070679	1.70E-08	1.53E-06	9.172722555
ABCC9	0.625517389	0.299937093	6.014828409	1.73E-08	1.55E-06	9.153132861
NOS3	0.781348628	0.763057473	6.011488171	1.76E-08	1.56E-06	9.137713872
RGS16	1.162617258	0.977605669	5.998462243	1.87E-08	1.64E-06	9.077629639
TMEM63C	1.362029689	0.859533921	5.984281952	2.01E-08	1.74E-06	9.01230275
P3H2	0.833246395	0.531741113	5.969609197	2.15E-08	1.85E-06	8.944797525
MGP	1.383364874	1.006901944	5.960397347	2.25E-08	1.90E-06	8.902463536
BHLHA15	1.170198688	1.542743018	5.946838495	2.40E-08	2.01E-06	8.840218848
RELN	0.766769624	0.198536747	5.92042204	2.73E-08	2.27E-06	8.719176832
TEAD1	0.692570233	0.337785631	5.912179263	2.83E-08	2.34E-06	8.681469858
XCR1	0.664543039	0.313482147	5.899831575	3.01E-08	2.47E-06	8.6250402
RAB3IL1	1.76443867	1.627798367	5.892927451	3.11E-08	2.53E-06	8.593516995
CCL13	0.654055036	0.283206476	5.890309078	3.15E-08	2.54E-06	8.58156735
PRDM1	1.23185691	2.877429293	5.861481431	3.61E-08	2.86E-06	8.450203937
ITGA8	0.717189757	0.459768242	5.834786184	4.10E-08	3.23E-06	8.328885076
CHST3	0.636000869	0.435312361	5.825235256	4.29E-08	3.35E-06	8.285557015
GFRA1	0.729065404	0.326410443	5.817125147	4.46E-08	3.47E-06	8.248797245
COL5A2	0.769645235	0.739511939	5.815087891	4.50E-08	3.49E-06	8.239567828
ARHGAP29	0.66793154	0.3002548	5.810048204	4.61E-08	3.56E-06	8.216744433
FBLIM1	0.89225191	0.614798378	5.777508498	5.37E-08	4.10E-06	8.069655628
CPNE5	1.225095709	2.266424582	5.770395885	5.56E-08	4.21E-06	8.037568225
LGMN	1.759083861	2.308126431	5.760396597	5.83E-08	4.39E-06	7.992496843
TMEM37	1.207256734	0.907372382	5.754600168	5.99E-08	4.50E-06	7.966390479
NAV2	0.680647802	0.570869618	5.753705528	6.01E-08	4.50E-06	7.962362497
SPIC	1.049098078	0.469379369	5.751164497	6.08E-08	4.54E-06	7.950923879
OSMR	1.075586545	1.520120762	5.737259269	6.50E-08	4.77E-06	7.888380731
LUM	2.007154093	1.35221265	5.736063207	6.53E-08	4.78E-06	7.883005189
CCL2	1.058583354	0.569383025	5.73521067	6.56E-08	4.78E-06	7.879173979
COL6A2	2.046040875	2.251493109	5.710155519	7.38E-08	5.34E-06	7.766727568
LOXL2	0.67867119	1.2896569	5.704181029	7.59E-08	5.47E-06	7.739956955
IGFBP3	1.488168449	1.298448783	5.690330289	8.10E-08	5.81E-06	7.677957698
POU2AF1	1.493106271	2.303866004	5.685696978	8.27E-08	5.90E-06	7.657237699
NFATC4	0.821152908	0.364763416	5.684174786	8.33E-08	5.92E-06	7.650432683
KCNJ8	0.807074403	0.348571111	5.666047248	9.07E-08	6.37E-06	7.569475618
SYNPO2	0.784440318	0.648056998	5.659074117	9.37E-08	6.56E-06	7.53837458
AMOTL2	0.960579388	0.572079347	5.649817833	9.78E-08	6.80E-06	7.49712549

FCRL5	1.389575042	1.78082989	5.640590555	1.02E-07	7.07E-06	7.456045591
MAGI1	0.725992278	0.206253122	5.638289165	1.03E-07	7.12E-06	7.445806013
SASH1	1.39644264	0.656065402	5.61923695	1.13E-07	7.73E-06	7.36113265
GP5	1.095260169	0.758955067	5.595415478	1.26E-07	8.54E-06	7.255504572
ADGRL2	0.944217424	0.563819617	5.586401411	1.31E-07	8.84E-06	7.21560503
KIAA1671	0.635257155	0.522341734	5.576941561	1.37E-07	9.09E-06	7.173773905
RAI14	1.161435468	0.773819156	5.576670203	1.37E-07	9.09E-06	7.172574601
NCKAP1	0.714966021	0.376417362	5.55937928	1.49E-07	9.79E-06	7.096227478
CHRDL1	2.424992178	1.525630212	5.553576545	1.53E-07	1.00E-05	7.070637924
TSPAN18	1.305766526	1.621534566	5.551935747	1.54E-07	1.01E-05	7.063405081
JAKMIP1	0.880213042	0.811686591	5.542573022	1.61E-07	1.05E-05	7.022157789
APLNR	1.163455389	0.667856159	5.538388122	1.64E-07	1.06E-05	7.003734946
PDGFB	0.948848166	0.851479557	5.52761785	1.72E-07	1.11E-05	6.956360728
PLA2G2D	0.681125224	0.547743002	5.522562731	1.76E-07	1.13E-05	6.934144569
NFIB	0.837017154	0.373359739	5.519332872	1.79E-07	1.14E-05	6.91995651
RAMP2	1.01085864	0.680074242	5.515823254	1.82E-07	1.15E-05	6.904545269
RGS5	0.827103377	0.810717242	5.493382174	2.02E-07	1.27E-05	6.806144706
PLVAP	1.73820372	1.713290284	5.493125972	2.02E-07	1.27E-05	6.805022713
RAB30	0.933187356	1.297631862	5.487207609	2.08E-07	1.29E-05	6.779113265
IL34	0.616579489	0.526184167	5.485687833	2.09E-07	1.30E-05	6.772462742
PKDCC	0.879585583	0.412476063	5.472660387	2.22E-07	1.37E-05	6.715501144
HINT1	-0.862062342	7.935519505	-5.463835835	2.31E-07	1.42E-05	6.676963691
OLFML3	1.700665242	1.618080521	5.447555573	2.49E-07	1.52E-05	6.605967155
DTHD1	0.901111167	0.831532206	5.433347165	2.65E-07	1.62E-05	6.544112679
BMP2	0.909066345	0.48499962	5.419375127	2.83E-07	1.72E-05	6.483384765
CMKLR1	1.722046126	1.841052602	5.408915481	2.97E-07	1.79E-05	6.437986597
CTTN	1.313635738	1.209487262	5.406103706	3.01E-07	1.81E-05	6.425791901
AC136428.1	1.515693226	1.216063762	5.388455166	3.26E-07	1.93E-05	6.349340222
IGFBP4	2.155185931	2.945744634	5.38448638	3.32E-07	1.96E-05	6.332169324
SLAMF7	1.735228934	3.272258668	5.382886588	3.34E-07	1.96E-05	6.32525008
ADAM19	1.542625404	2.795049451	5.371919206	3.51E-07	2.05E-05	6.277849778
SMOC1	0.773868832	0.390218734	5.368691637	3.56E-07	2.07E-05	6.263911962
GPX3	0.988078759	2.448188882	5.367630519	3.58E-07	2.07E-05	6.259330814
COL4A2	1.257937944	0.774088125	5.363873014	3.64E-07	2.10E-05	6.243113159
LAMB1	0.818951717	1.7827362	5.361714108	3.68E-07	2.11E-05	6.233798391
C1QB	3.010611093	2.760989761	5.356128246	3.77E-07	2.16E-05	6.209708676
KITLG	0.94724439	0.875399976	5.353121947	3.82E-07	2.18E-05	6.196750165
ZDBF2	0.746414465	0.595248411	5.351735415	3.85E-07	2.19E-05	6.190775126
IGLL5	2.326544209	4.040439586	5.346383292	3.94E-07	2.24E-05	6.167720122
EDNRB	0.689678653	0.431572797	5.344072227	3.98E-07	2.25E-05	6.157769377
WFS1	1.604126223	1.488093795	5.317750426	4.48E-07	2.53E-05	6.044627076
CYP1B1	2.326237339	2.426508509	5.316660429	4.51E-07	2.53E-05	6.039949411
TRPC6	0.731704126	0.255680417	5.315523219	4.53E-07	2.54E-05	6.035069777
RAMP3	0.89736895	0.556083084	5.310887357	4.62E-07	2.58E-05	6.015184691
IRF4	1.574181481	2.709615396	5.307450759	4.70E-07	2.61E-05	6.000450818
TEK	1.035247521	0.847739254	5.300365621	4.85E-07	2.68E-05	5.970093476
MAF	1.316785736	2.050948606	5.295161366	4.96E-07	2.72E-05	5.947811449

ZBP1	1.095356953	1.63652197	5.295110009	4.96E-07	2.72E-05	5.947591633
NUGGC	0.867394355	1.046724421	5.290120053	5.08E-07	2.78E-05	5.926240308
LPL	2.040319686	1.774079386	5.279700288	5.32E-07	2.88E-05	5.881696845
TIMP4	1.404110027	1.045780077	5.271602366	5.52E-07	2.97E-05	5.847117646
LIMCH1	0.786388562	0.376930563	5.270978387	5.53E-07	2.97E-05	5.844454574
EMCN	0.870756803	0.417472472	5.268661695	5.59E-07	2.99E-05	5.834568962
PRG4	0.624440426	0.981185974	5.258116743	5.86E-07	3.12E-05	5.789607389
LRRC32	1.236132437	0.835118831	5.255511408	5.93E-07	3.15E-05	5.778507623
FBN1	1.452548644	1.61653078	5.25162555	6.03E-07	3.20E-05	5.761958854
UTS2R	0.687551759	0.503846622	5.242510912	6.28E-07	3.31E-05	5.723172938
FCRL6	1.505141487	2.398626527	5.229661035	6.66E-07	3.49E-05	5.668565674
SGK1	1.985284737	2.824965197	5.227467889	6.72E-07	3.52E-05	5.659254204
TIMP3	0.979498248	0.405186002	5.223065021	6.85E-07	3.58E-05	5.640568458
EOMES	1.235211351	1.868455784	5.21043839	7.25E-07	3.76E-05	5.587037308
SSTR3	0.675447479	0.448094788	5.198580953	7.64E-07	3.95E-05	5.536843235
ABCC3	1.487017018	1.29277221	5.177927024	8.38E-07	4.29E-05	5.449589002
HES1	1.113500446	0.846707699	5.171947342	8.60E-07	4.38E-05	5.424369338
PDGFRB	1.473106708	2.024940228	5.163694855	8.92E-07	4.51E-05	5.38959505
KCNK1	0.953723362	0.236004612	5.1587709	9.12E-07	4.59E-05	5.368863699
VGLL3	1.00585393	0.465859032	5.158408758	9.13E-07	4.59E-05	5.367339477
A2M	1.517908928	2.659366425	5.15066704	9.45E-07	4.74E-05	5.33477199
KLRD1	1.205540789	1.783325612	5.145684247	9.66E-07	4.83E-05	5.31382746
FPR3	1.328092303	1.263081689	5.138976125	9.95E-07	4.97E-05	5.285651604
CCL4L2	1.194002994	1.251793978	5.133744919	1.02E-06	5.07E-05	5.263695825
CXCL14	1.063622003	0.592105126	5.13129714	1.03E-06	5.11E-05	5.253427324
TBC1D12	1.417552284	1.032193487	5.116240297	1.10E-06	5.41E-05	5.190333877
B3GAT1	0.65761713	0.473349374	5.115988499	1.10E-06	5.41E-05	5.189279787
CXCL9	1.321795109	0.617808402	5.110545452	1.13E-06	5.52E-05	5.16650213
DAAM2	1.433121946	0.644770166	5.098719739	1.19E-06	5.78E-05	5.117069593
ACKR1	1.311660528	0.955173505	5.096575352	1.20E-06	5.81E-05	5.108113921
FHL2	1.22290196	1.298910317	5.09553812	1.21E-06	5.82E-05	5.103782982
C1QC	2.829120763	2.725427506	5.085372675	1.26E-06	6.04E-05	5.061368102
THBD	1.449365718	2.282469083	5.072136989	1.34E-06	6.35E-05	5.006226409
COL6A1	2.050087358	2.127747657	5.063929971	1.38E-06	6.57E-05	4.972082484
FCN2	0.776696193	0.575716081	5.057304439	1.43E-06	6.72E-05	4.944544729
KRT79	0.770289912	0.177334233	5.057171658	1.43E-06	6.72E-05	4.943993093
SLC16A6	1.303472545	1.403485118	5.036242624	1.56E-06	7.31E-05	4.857163985
ARSG	0.979027005	1.722179836	5.034319836	1.58E-06	7.34E-05	4.849198822
EDNRA	0.629887314	0.276549136	4.999674961	1.83E-06	8.49E-05	4.706029239
KIR2DL1	0.6220127	0.470441145	4.99752712	1.85E-06	8.55E-05	4.697175023
NR4A1	2.050809935	3.42797839	4.995651198	1.86E-06	8.59E-05	4.689443841
LILRB5	0.981770603	0.613079755	4.995382741	1.87E-06	8.59E-05	4.688337614
HERPUD1	1.025197753	5.507596729	4.993551562	1.88E-06	8.64E-05	4.680792975
SYNPO	0.78479203	1.06282451	4.986736708	1.94E-06	8.85E-05	4.652731352
TNFRSF9	0.623369756	0.712320872	4.975133587	2.04E-06	9.29E-05	4.605012086
LAMP3	0.632656791	0.551883352	4.964264572	2.14E-06	9.67E-05	4.560379591
TNFRSF13B	0.7488496	0.825573505	4.958940653	2.19E-06	9.87E-05	4.538541396

JCHAIN	2.04545971	6.790014499	4.956776744	2.21E-06	9.94E-05	4.529669759
APOD	1.350247156	0.727408022	4.95411285	2.23E-06	0.000100221	4.518751853
MYO10	0.678612115	0.531133913	4.953764363	2.24E-06	0.000100221	4.517323878
ZNF208	0.700213312	0.367523791	4.944126328	2.33E-06	0.000104238	4.477857518
XCL1	0.794512074	0.738978695	4.94289953	2.34E-06	0.000104363	4.472837674
RBP7	1.47523137	1.493807902	4.940102495	2.37E-06	0.000105336	4.461395836
PCOLCE	0.968083444	1.938033183	4.936717688	2.41E-06	0.000106641	4.447555442
C1QA	2.882103464	3.16539599	4.93479495	2.43E-06	0.000107283	4.439696268
XCL2	1.195019536	1.585166974	4.924725046	2.53E-06	0.000111783	4.398569408
CSF1	1.532791503	2.56938114	4.923921711	2.54E-06	0.000111917	4.395290923
IGF2	1.582010174	1.30768404	4.908763443	2.71E-06	0.000119182	4.333496568
ACVRL1	1.110785919	0.99021796	4.898310943	2.84E-06	0.00012437	4.290961078
SGMS1	0.987692985	3.413588929	4.877355985	3.10E-06	0.000134534	4.205872551
TGFBR3	1.102270056	1.659459846	4.874124105	3.15E-06	0.000136103	4.192771476
CLMP	0.616465364	1.15343062	4.873427568	3.16E-06	0.000136126	4.189948704
EPOR	1.50826897	3.347868837	4.872497783	3.17E-06	0.000136145	4.186181098
ZCCHC24	1.288726338	2.596728912	4.869007769	3.22E-06	0.000137887	4.172043502
IKZF3	1.307695188	3.261758073	4.868163537	3.23E-06	0.000138081	4.168624667
BMP6	1.166695142	1.449880384	4.865895499	3.26E-06	0.00013872	4.159441932
SLC7A2	0.926302471	0.640904331	4.85018662	3.49E-06	0.000147376	4.095920815
DPF3	0.692255796	0.576403537	4.849530182	3.50E-06	0.000147376	4.093269471
PCSK1N	0.790590899	0.433689814	4.849319306	3.50E-06	0.000147376	4.092417801
ID3	1.074024645	1.102483459	4.844714221	3.57E-06	0.000149649	4.073825404
PMEPA1	1.09508894	1.408604303	4.81410388	4.06E-06	0.000167921	3.950548681
LTA	0.865884056	1.141035509	4.814094499	4.06E-06	0.000167921	3.950510984
ACP5	1.221946735	2.946741112	4.811743724	4.11E-06	0.000169245	3.941065966
ZFP36L1	1.742284715	5.058603032	4.804617502	4.23E-06	0.000174067	3.912453445
TIMD4	1.608282874	1.49371738	4.80164517	4.28E-06	0.000175901	3.900527865
RIPOR3	1.273124528	3.471729503	4.796614229	4.38E-06	0.000179311	3.880354353
SPAG4	0.824312408	2.554653447	4.793913755	4.43E-06	0.000180991	3.869531777
NRP2	0.861287436	0.588136816	4.789211177	4.52E-06	0.000184241	3.850695506
ENPEP	1.085895851	0.689498072	4.776666684	4.76E-06	0.000192655	3.800510859
EXT1	1.080347061	1.35437553	4.77488581	4.80E-06	0.000193707	3.793393802
TGFBI	2.324272174	2.420146301	4.7729752	4.84E-06	0.000194871	3.785760316
SFRP5	0.961156818	1.054732127	4.764384276	5.02E-06	0.000201644	3.751463075
CPEB4	0.908847809	4.09860651	4.745797232	5.42E-06	0.00021716	3.677405604
MAN1C1	0.973367924	1.702334219	4.743539856	5.48E-06	0.000218781	3.668425122
FASLG	0.8890162	0.948402207	4.733369557	5.71E-06	0.000226471	3.628001714
IGFBP5	1.767804256	1.640421088	4.724940856	5.92E-06	0.000234147	3.594546424
TSKU	0.878008382	0.615679974	4.722737311	5.97E-06	0.000235841	3.585806959
CHST2	1.413354254	3.517207301	4.721481291	6.01E-06	0.000236606	3.580826738
CEMIP2	1.058239286	3.526161179	4.716079405	6.14E-06	0.000241533	3.559418385
PLOD2	0.86571294	0.366421244	4.708985413	6.33E-06	0.000247813	3.531330049
S1PR5	1.19827417	1.684389511	4.706077317	6.41E-06	0.000250342	3.51982413
SEMA4B	0.974857506	3.76542624	4.699901138	6.57E-06	0.000256376	3.495404523
SLC9A9	1.538730161	2.325691058	4.684063388	7.02E-06	0.000272808	3.432887495
PAQR9	0.966614577	0.527092038	4.676171879	7.26E-06	0.000280533	3.40179237

EYA1	0.783342562	0.440540662	4.671973117	7.39E-06	0.000284631	3.385262914
ATP1B2	1.70463263	1.438374284	4.670216843	7.44E-06	0.000286153	3.378352013
BCL2L11	1.172540554	3.444237968	4.668735239	7.49E-06	0.000287356	3.372523355
IPP	0.615065283	1.951437223	4.664637322	7.62E-06	0.000291148	3.356408864
CH25H	0.687156485	0.228098834	4.654579252	7.94E-06	0.0003018	3.31689921
GFRA2	0.65059231	0.631832247	4.62799909	8.87E-06	0.000334349	3.212778524
IFNLR1	0.922673771	0.9621571	4.626064231	8.94E-06	0.000336385	3.205215725
DMPK	1.122429477	1.748038919	4.620271069	9.15E-06	0.000343877	3.182585353
MYO1E	1.139911472	2.069297516	4.610477349	9.53E-06	0.000356698	3.144373034
STEAP4	1.043400893	0.855301692	4.608640029	9.60E-06	0.000358724	3.137210754
CD27	1.081055805	3.435622954	4.596369576	1.01E-05	0.000374469	3.089429889
TGM2	1.43295204	1.819322983	4.593570596	1.02E-05	0.000378091	3.078543431
EPHA3	0.690359652	0.139724498	4.590019988	1.04E-05	0.000382932	3.064740369
CFI	1.023082326	0.619610532	4.588344565	1.04E-05	0.000384851	3.05822977
DMWD	1.135518819	2.063897884	4.587745524	1.05E-05	0.000385072	3.055902339
CLCN5	1.244871386	2.431983634	4.587239541	1.05E-05	0.000385147	3.053936635
WLS	1.091074439	0.764931041	4.584882503	1.06E-05	0.000388168	3.044781762
BTNL9	0.971761793	0.73033023	4.577929471	1.09E-05	0.000398674	3.017795352
IRS1	0.741808779	0.796951141	4.570861141	1.12E-05	0.000408631	2.990391443
MYBL1	0.936327254	2.218749981	4.570555452	1.12E-05	0.000408631	2.989206969
F2RL3	0.891726806	0.781842833	4.568867931	1.13E-05	0.000410704	2.982669243
CHPF	1.2213204	2.275239853	4.567545468	1.14E-05	0.00041217	2.977547016
TMEM63B	0.998445947	3.68528116	4.551742709	1.21E-05	0.000438122	2.916421191
ACSBG1	0.892614369	0.846390654	4.548837907	1.23E-05	0.000442542	2.905201806
CD5L	1.496205391	0.974171084	4.530565698	1.32E-05	0.00047162	2.834745959
ANG	0.889492533	1.097202738	4.520148883	1.38E-05	0.000488514	2.794671061
TTC16	0.746241507	0.895363162	4.516602815	1.40E-05	0.000494725	2.781044019
HTRA4	0.795139139	0.884852385	4.510891912	1.43E-05	0.000505376	2.759114037
SNTB2	1.028840616	2.000947153	4.510482369	1.44E-05	0.000505376	2.757542152
STUM	0.726948435	0.397810437	4.497560243	1.51E-05	0.00052882	2.707998142
HPSE2	0.706103325	0.141371653	4.491870211	1.55E-05	0.000540213	2.686214888
CCL18	1.195437636	0.717770431	4.47431435	1.66E-05	0.000577995	2.619131366
RNF182	0.946807961	0.366551197	4.468050704	1.71E-05	0.000589677	2.595243179
RGS1	1.704893275	3.331255547	4.459732842	1.76E-05	0.000608764	2.563558227
COL18A1	1.06705044	2.546381136	4.443917718	1.88E-05	0.00064774	2.503432706
ITPRIP	0.922755024	4.467103504	4.439337961	1.92E-05	0.00065864	2.486050564
CD40LG	1.158152682	2.298772453	4.434869979	1.95E-05	0.000669416	2.469105252
THBS1	2.419303724	3.197779175	4.434187694	1.96E-05	0.000670076	2.466518707
IPCEF1	1.251934205	2.515343029	4.433041274	1.96E-05	0.000671992	2.462173279
NRN1	0.623419339	0.435189597	4.404649413	2.20E-05	0.000750525	2.35481799
CMTM5	1.285734808	0.886689899	4.403114589	2.22E-05	0.000753835	2.349028901
RCN3	1.52953789	3.033534293	4.402263266	2.22E-05	0.000754455	2.345818501
CLCN4	1.179770295	1.147412359	4.402038205	2.23E-05	0.000754455	2.344969853
TPM1	1.04454683	2.042599097	4.393823202	2.30E-05	0.000778328	2.314015019
KIR3DL1	0.618468333	0.526490258	4.392480103	2.31E-05	0.000781163	2.308958146
MTHFR	0.748154263	4.352752926	4.39153123	2.32E-05	0.000782775	2.305386246
SRPX	0.8857123	0.663801088	4.390630408	2.33E-05	0.000784242	2.301995754

SIK1B	1.336357626	1.931211902	4.384095633	2.39E-05	0.000800842	2.277415616
UBASH3A	1.226976128	2.18818968	4.383606269	2.40E-05	0.000801031	2.275575986
DUSP5	1.228366651	3.618618852	4.376458662	2.47E-05	0.000821401	2.248723752
SPRED1	1.451352669	1.251202423	4.372663241	2.50E-05	0.000832509	2.234478168
CD200R1	1.336093347	2.099388365	4.370837592	2.52E-05	0.000837162	2.227629088
NMU	1.58580128	1.054976457	4.34605933	2.78E-05	0.000917672	2.13487996
PROS1	1.250543019	1.033598994	4.339356411	2.86E-05	0.00094083	2.109856769
CHST15	2.117132155	3.220297497	4.335839086	2.90E-05	0.00095244	2.096737396
WNT5B	0.739726516	0.867034989	4.33035893	2.96E-05	0.000970085	2.076312501
SLAMF8	0.808188659	1.099477988	4.327373821	3.00E-05	0.000979977	2.065194857
EPHB2	0.894158462	0.60080451	4.324045798	3.04E-05	0.000989653	2.052806776
WIPI1	1.041076572	3.212956555	4.318999072	3.10E-05	0.001007932	2.034034553
CDH1	1.350647237	1.219128317	4.317979919	3.11E-05	0.001010314	2.030245602
AGAP1	0.996383922	0.900972901	4.316827904	3.12E-05	0.001013237	2.025963501
PPARD	1.105554587	3.356654163	4.314636415	3.15E-05	0.00101866	2.017819963
ESYT2	-0.723225558	7.043807759	-4.310439691	3.20E-05	0.001033981	2.002233572
JAG2	0.591447683	0.659173656	4.307548153	3.24E-05	0.001040682	1.991501123
FCAR	2.252162954	3.726637299	4.304579214	3.28E-05	0.001047767	1.980486956
TBC1D9	1.48529109	2.592882436	4.291785725	3.45E-05	0.001094797	1.933090308
TNS2	0.670312149	1.968686036	4.287429326	3.51E-05	0.00111193	1.916974926
ZNF703	1.393093113	1.678454142	4.286651199	3.52E-05	0.001113528	1.914097727
RGS2	2.082639935	5.67184092	4.284566806	3.55E-05	0.001119062	1.906392407
CLEC1A	0.615517253	0.519701502	4.282988291	3.57E-05	0.001124213	1.900559013
CAMLG	-0.656389539	5.144950235	-4.279570547	3.62E-05	0.001135753	1.887934247
JUNB	1.273249278	8.415425677	4.279139552	3.62E-05	0.001135848	1.88634273
NRP1	1.503488462	1.411776292	4.277795052	3.64E-05	0.001140032	1.88137873
AMIGO2	1.029355644	1.630772868	4.271015795	3.74E-05	0.001165188	1.856366921
DKK1	0.810980471	0.232641982	4.267939595	3.79E-05	0.001175585	1.845027172
CPAMD8	0.895175001	0.830138972	4.261581649	3.88E-05	0.001199539	1.821609327
SUPT3H	-0.648238901	3.282671391	-4.257528831	3.95E-05	0.001214892	1.806695451
FCRLA	1.22708308	1.939993212	4.254176396	4.00E-05	0.001225139	1.794366924
C16orf46	-0.639218433	1.966510153	-4.249341495	4.07E-05	0.001246589	1.776599461
PAPSS2	1.060132081	2.609208898	4.247032504	4.11E-05	0.001253961	1.768119637
GZMK	1.267816097	2.677336443	4.238273509	4.25E-05	0.001289563	1.735983453
FN1	1.383828225	2.25340817	4.234154681	4.32E-05	0.001306196	1.720888949
DEPP1	1.240519282	3.176148014	4.23379855	4.33E-05	0.001306196	1.719584335
PLEKHH3	1.06137763	1.844181121	4.233022562	4.34E-05	0.001308131	1.716741937
CCN2	1.63275679	1.397555668	4.232450061	4.35E-05	0.001309031	1.714645156
SLC18B1	1.222371406	2.622138592	4.229998044	4.39E-05	0.001317693	1.705667056
TNFAIP3	1.092438029	5.355255476	4.229969423	4.39E-05	0.001317693	1.705562281
ABHD5	0.820657787	4.180501553	4.224361534	4.49E-05	0.001342694	1.68504371
OLFML2B	1.520355879	2.357517418	4.220924406	4.55E-05	0.001356634	1.672477798
ATP8B4	-1.275229518	6.143161683	-4.21856688	4.59E-05	0.00136497	1.663863288
NIBAN2	1.755439908	3.846634564	4.214070322	4.68E-05	0.001382745	1.647442697
ZNF831	0.876227985	1.468303994	4.209439691	4.76E-05	0.001405747	1.630546278
IL2RB	1.422654113	3.794522532	4.207654254	4.79E-05	0.001412926	1.624035246
KLRC4	1.10974579	1.573667014	4.207350332	4.80E-05	0.001412926	1.622927127

KLHL14	0.601603497	0.778907661	4.200743456	4.92E-05	0.001447482	1.598852965
SIGLEC1	2.017959947	1.859803923	4.198350032	4.97E-05	0.001458794	1.590138837
ZNF560	0.846293513	0.289160619	4.196806431	5.00E-05	0.001465352	1.584520785
ZNF532	1.438610493	1.594009767	4.196335589	5.01E-05	0.001465825	1.58280743
CHAC1	0.864321474	1.010758812	4.1959369	5.02E-05	0.00146589	1.581356748
ABCA6	0.82490923	0.693274695	4.191634081	5.10E-05	0.001488159	1.565706995
LDOC1	1.514746223	1.469651518	4.189322548	5.15E-05	0.001497242	1.557304742
FAM83D	1.027069101	3.147827305	4.188296621	5.17E-05	0.001500965	1.553576694
IFI27	1.146572568	1.185830068	4.186398466	5.21E-05	0.001507547	1.546680934
ESR2	0.671601994	0.701018642	4.185659532	5.22E-05	0.001508078	1.54399712
GZMM	1.086346112	2.590080326	4.185540677	5.22E-05	0.001508078	1.543565468
KLRC4-KLRI	0.65226849	0.760224882	4.18174014	5.30E-05	0.001521413	1.529767832
NR4A2	1.666174278	5.150946496	4.173868801	5.46E-05	0.001563854	1.501221537
BICDL1	0.847791955	1.671430122	4.171992895	5.50E-05	0.00156962	1.494424357
CLEC4G	1.224761321	1.395207483	4.171287749	5.52E-05	0.00156962	1.491869923
GOLGA8N	-0.933564416	3.312459848	-4.169737194	5.55E-05	0.001575136	1.486254089
RNF157	0.908136394	1.53649929	4.169238039	5.56E-05	0.00157588	1.484446576
SYTL3	1.172947831	3.348075545	4.168642654	5.58E-05	0.001577214	1.482290816
FZD1	0.963614885	2.104285752	4.167026896	5.61E-05	0.001584787	1.476441676
AGTR1	0.678737255	0.453612577	4.161522744	5.73E-05	0.001614143	1.456529222
CD99	-0.927659488	7.75317878	-4.159858781	5.77E-05	0.001621956	1.450513405
FHDC1	1.263948319	1.725395289	4.15952245	5.78E-05	0.001621956	1.44929767
ADGRD1	0.72831802	0.602957029	4.151869976	5.95E-05	0.001665722	1.421656463
SIT1	1.147987423	2.751298247	4.142661886	6.16E-05	0.001715967	1.388447524
NLRP7	0.640858451	0.633707826	4.139417148	6.24E-05	0.001730094	1.376758741
SIRPG	1.112872235	2.172632281	4.137991	6.28E-05	0.001737133	1.37162342
MYL4	1.70979952	2.105493138	4.121974268	6.67E-05	0.001836871	1.314042343
CXCL16	1.739607873	2.879244845	4.119024555	6.75E-05	0.001855137	1.303456511
GYPE	1.476164281	1.92628174	4.11654926	6.81E-05	0.001870177	1.294577713
HSH2D	-1.285389556	7.758139136	-4.115108218	6.85E-05	0.00187524	1.289410622
PKLR	1.767654491	1.711637667	4.114723558	6.86E-05	0.001875367	1.288031594
SCAMP5	1.132727924	2.693461791	4.112304364	6.93E-05	0.00188752	1.279360899
PDZD4	0.941209959	1.396856331	4.105274087	7.11E-05	0.001926162	1.254185608
AVPI1	0.828430213	2.492739682	4.10494318	7.12E-05	0.001926162	1.253001447
APOC1	1.419485032	1.499128387	4.104821028	7.13E-05	0.001926162	1.252564339
SH3BP5	1.183025632	2.022147657	4.103803038	7.15E-05	0.001930992	1.248921973
SELENBP1	1.977621687	2.552277197	4.096620226	7.35E-05	0.001971055	1.223241496
EPHX2	0.871991524	1.082499962	4.090962163	7.51E-05	0.002008554	1.203036636
ETV6	-0.667905483	7.28047334	-4.088940932	7.57E-05	0.002021118	1.19582403
THRB	0.870688433	0.46112433	4.085400781	7.68E-05	0.002043187	1.183197859
WWTR1	0.650089918	0.652974389	4.080216181	7.83E-05	0.002078263	1.164721774
PI4K2A	1.113305217	2.731147188	4.079087225	7.86E-05	0.002084373	1.160700957
TINAGL1	0.917697514	0.681518321	4.078434377	7.88E-05	0.002086718	1.158376206
MRVI1	0.93484158	1.621305751	4.076286365	7.95E-05	0.002100992	1.150729282
NFIA	1.682778899	1.693403135	4.070969729	8.11E-05	0.00214101	1.131815347
PLCH2	0.71770502	1.036084385	4.069647203	8.15E-05	0.002148888	1.1271134
ANKRD9	0.976915232	2.048454513	4.064857139	8.30E-05	0.002182448	1.11009322

SLC44A2	1.190423084	5.139382441	4.063704179	8.34E-05	0.002189069	1.10599879
GLI3	0.682625233	0.399168269	4.060705091	8.43E-05	0.002205212	1.095352514
GBP1	1.63084408	2.531349265	4.056235121	8.58E-05	0.002236922	1.079496056
IL21R	0.877389974	1.63030608	4.055632334	8.59E-05	0.002239041	1.077358796
PTGDR	1.077720285	1.661772557	4.054882027	8.62E-05	0.00224242	1.07469883
ARHGEF12	1.1617955	1.968928645	4.038421232	9.17E-05	0.002364542	1.016437854
FAM20A	0.837455785	1.408481832	4.038420123	9.17E-05	0.002364542	1.016433935
TBX21	1.14051222	2.167369119	4.036527986	9.24E-05	0.002378371	1.009748647
TNXB	1.060329655	1.348647482	4.034964012	9.29E-05	0.002389313	1.00422465
SLC41A2	0.727768878	0.935695887	4.031733171	9.41E-05	0.002412288	0.992818457
IL10RA	1.620665743	4.86945179	4.031183608	9.43E-05	0.00241412	0.990878977
NNMT	1.146806499	1.293469789	4.029265064	9.50E-05	0.00242848	0.984109781
ARL4C	1.143252639	4.152206861	4.027523084	9.56E-05	0.002441297	0.977965707
VPS37C	0.687285283	3.836060615	4.02547625	9.63E-05	0.002457005	0.970749016
SPTB	1.595201323	2.040056068	4.024139761	9.68E-05	0.002462971	0.966038376
REM2	0.62649874	1.368418908	4.022289831	9.75E-05	0.002473751	0.959520035
SLC6A8	1.188843162	2.017847632	4.021829929	9.77E-05	0.002474819	0.957899901
EGR2	1.750194415	2.422666244	4.021183251	9.79E-05	0.002477636	0.955622038
COL6A6	0.6803196	0.394804933	4.016209453	9.98E-05	0.002521245	0.938111766
ASAP3	0.592385959	0.551420379	4.015282265	0.000100111	0.002525645	0.934849451
CR1L	1.086383491	1.065000665	4.007792	0.000102974	0.002585638	0.908516278
PCED1B	1.078638017	2.975299748	4.004273012	0.000104346	0.002616712	0.896157877
IL6ST	1.168978738	4.461585045	4.003672576	0.000104582	0.002619251	0.894050031
LRP1	2.362757712	4.014806711	4.002930183	0.000104874	0.002623195	0.891444185
GASK1B	1.966844658	3.242688766	3.999081399	0.000106402	0.002650741	0.877940699
TLCD4	1.417968339	1.475888425	3.999018348	0.000106427	0.002650741	0.877719567
PMM1	0.83843342	2.032967349	3.998785705	0.00010652	0.002650741	0.876903671
HK1	0.974944109	5.685478198	3.998284898	0.000106721	0.002651253	0.875147427
LILRB1	1.783134975	3.486813013	3.997714986	0.000106949	0.002651253	0.87314905
TMPRSS13	0.648641518	0.928636035	3.99446453	0.000108263	0.002680395	0.861755663
HHEX	-0.618870596	6.542910424	-3.989483983	0.000110305	0.002721647	0.844311958
CKAP4	1.685487415	3.842491992	3.989041011	0.000110488	0.002721647	0.842761324
HESX1	-0.992606691	2.939117006	-3.988625614	0.00011066	0.002722444	0.84130734
DGAT2	0.993550738	1.544983241	3.987047533	0.000111317	0.00273175	0.835784766
CD274	0.760028451	1.082477355	3.984661688	0.000112317	0.002749298	0.827438599
RHCE	1.433971981	2.023492114	3.98365315	0.000112742	0.002754462	0.823911697
SLC22A17	0.956754965	0.984681716	3.983330986	0.000112879	0.002754462	0.822785221
TIGIT	0.723179709	1.446929476	3.981303919	0.000113739	0.002770164	0.815699018
FLT3	-1.285952913	7.803361834	-3.980905622	0.000113909	0.002770831	0.814306987
CTLA4	0.911635588	1.387833039	3.966082613	0.000120403	0.002922157	0.762578078
EFNB2	0.721324422	0.744489947	3.965760251	0.000120548	0.002922157	0.761454777
CD8A	1.194217614	2.931047869	3.965687672	0.00012058	0.002922157	0.761201878
GZMH	1.476729646	3.070309772	3.965254072	0.000120776	0.002923253	0.759691089
HMOX1	1.948844374	3.843200019	3.962243464	0.000122141	0.002952628	0.749204801
ADM	1.236450857	1.610964733	3.960731231	0.000122832	0.002965658	0.743939863
GALM	1.022321784	2.890048393	3.956192014	0.00012493	0.002999601	0.72814568
SEC14L4	1.235998294	1.136095445	3.955769016	0.000125127	0.002999601	0.72667458

MFAP3L	0.96735813	0.957259331	3.955692433	0.000125163	0.002999601	0.726408254
ERN1	0.755710185	3.873807039	3.954307356	0.000125811	0.003011422	0.72159217
CCL3	1.249576448	2.613314957	3.953852538	0.000126025	0.003012261	0.720010998
ABCB6	0.792357446	1.585093324	3.953573558	0.000126156	0.003012261	0.719041193
BLVRB	1.326495283	5.750857815	3.953217879	0.000126323	0.00301256	0.717804844
CA3	0.978379744	1.276381452	3.951721213	0.00012703	0.003025702	0.712603346
ISG20	1.039315559	2.95057698	3.949746456	0.000127968	0.003040456	0.705742643
KRT13	0.692359264	0.369812104	3.9475161	0.000129035	0.003051062	0.697997156
CA1	2.297076119	4.596043375	3.947064019	0.000129253	0.003052493	0.696427602
TMEM158	0.873657198	0.898758032	3.942329744	0.00013155	0.003102447	0.679999387
TREML1	1.282123439	1.807884525	3.942050938	0.000131687	0.003102447	0.679032393
TCTN2	-0.61574535	2.961237598	-3.934179504	0.000135597	0.003171687	0.65175363
CD248	1.165187388	1.54086522	3.934166393	0.000135603	0.003171687	0.651708231
IFNG	0.882285148	1.1570379	3.93218629	0.000136604	0.003187438	0.644852829
TUBA4A	1.438588205	4.333136835	3.931291796	0.000137059	0.003192671	0.641756845
TUBAL3	0.856562096	0.460073724	3.931099482	0.000137157	0.003192671	0.641091288
TIAM1	1.212723152	1.645129149	3.930292335	0.000137568	0.003197626	0.638298207
PCSK5	0.627097819	0.840097274	3.930038123	0.000137698	0.003197626	0.637418613
SNPH	0.784544876	1.274188826	3.928664661	0.000138402	0.003210138	0.632667104
COL14A1	1.172149333	1.121359659	3.928086606	0.000138699	0.003212479	0.630667707
HPSE	1.439752598	2.930659831	3.927120171	0.000139198	0.00321327	0.627325474
PEAR1	1.825492444	2.349792549	3.925752475	0.000139906	0.003225789	0.622596659
GLDN	0.927161045	0.700933439	3.922276396	0.000141721	0.003256065	0.610583896
ACVR2A	0.747696612	1.582562417	3.920813927	0.000142491	0.003268065	0.605532327
ARHGAP6	1.003839475	0.844017316	3.920554152	0.000142628	0.003268065	0.604635184
CDC25B	0.698835333	5.135693147	3.920012788	0.000142915	0.003268065	0.602765712
MSC	0.820903704	0.715029366	3.918938472	0.000143485	0.003277249	0.599056409
TMCC3	0.903486236	1.22545433	3.917852376	0.000144063	0.003286604	0.595307246
RPL22L1	-0.675698361	5.150983224	-3.915024473	0.00014558	0.003305762	0.585549242
GNAZ	0.790617065	0.798851892	3.915019697	0.000145583	0.003305762	0.585532767
CAMK2D	1.192035646	2.243508408	3.906580879	0.0001502	0.003394755	0.556446505
FTH1	0.952339907	7.943376372	3.905419957	0.000150846	0.003401453	0.552448979
LY6G6E	1.039301474	0.793315993	3.902638413	0.000152404	0.003427323	0.542874784
FAM167B	0.705099616	0.960267166	3.902274069	0.000152609	0.003427323	0.541621093
CHRFAM7A	-0.685751095	1.433893149	-3.901093919	0.000153276	0.003436349	0.537560879
EPHA2	0.865512862	0.656056372	3.898506346	0.000154748	0.00345739	0.528661909
IQSEC1	1.109620065	5.009006179	3.893811237	0.000157452	0.003512045	0.512526713
RBMS3	0.686511699	0.466551716	3.893143247	0.00015784	0.003512045	0.510232341
C20orf27	0.963522679	5.496546352	3.893013897	0.000157916	0.003512045	0.509788091
SLC41A1	1.35391623	2.630183601	3.889624676	0.000159901	0.003548098	0.498152065
Z82206.1	0.97712204	1.868475977	3.88868523	0.000160456	0.003556349	0.494928127
PECR	1.043863259	1.414107153	3.887723981	0.000161025	0.003562107	0.491629998
TMEM38A	0.946240242	1.364118067	3.887628747	0.000161082	0.003562107	0.49130328
IFITM10	0.882938577	1.337152899	3.884048159	0.00016322	0.003605301	0.479023852
ARHGAP42	0.640150221	0.531828889	3.883203425	0.000163729	0.00360834	0.47612818
KCNA3	1.468332627	3.645045155	3.880007298	0.000165666	0.003646909	0.46517663
GLT1D1	1.557666794	1.684444456	3.876471623	0.000167835	0.00368631	0.453069879

NOTCH4	0.628910308	1.193754163	3.874999135	0.000168746	0.003702143	0.448030395
HSPA12B	0.786881598	2.04697203	3.859296008	0.00017876	0.003891132	0.394381369
EML4	0.658503734	4.571928355	3.857990751	0.000179617	0.003905426	0.389929749
LILRA5	2.429455093	3.447420747	3.855901149	0.000180998	0.003931053	0.382805569
NEDD4L	0.933834884	1.131980216	3.855453007	0.000181296	0.003933123	0.381278095
SOX6	0.627086779	0.889212662	3.851097631	0.00018421	0.003987468	0.366440253
TUBB2A	1.211133865	1.842000711	3.84791999	0.000186365	0.004016239	0.355623061
ZMYND15	0.853806262	0.843572233	3.846831471	0.000187109	0.004027803	0.351919188
RTN1	1.079097358	0.898680262	3.84644239	0.000187375	0.004029082	0.350595478
ART4	0.831761889	0.691602422	3.84313164	0.000189657	0.004073652	0.339336082
SLFN5	1.126404582	2.91429314	3.84062448	0.000191403	0.00410211	0.330814678
ITSN1	0.803946273	1.979791592	3.84046501	0.000191515	0.00410211	0.330272817
IMMP2L	-0.614980651	2.555962881	-3.84032214	0.000191614	0.00410211	0.329787374
MAPK13	0.722502932	2.955988617	3.836249387	0.000194486	0.004147958	0.315955029
VIPR1	1.131965597	2.077591009	3.834863089	0.000195473	0.004161815	0.311249373
PTGS2	1.620103238	3.796980922	3.831582552	0.000197827	0.004202736	0.300119266
PPP1R3B	0.736281371	2.746489398	3.830848811	0.000198357	0.004209404	0.297630887
RASD1	1.581429896	2.341395821	3.824625997	0.000202908	0.004291939	0.276542231
CLIC3	1.025357478	1.698721265	3.824049827	0.000203334	0.004293806	0.274591008
SOCS3	1.552112741	5.304589533	3.82365049	0.00020363	0.004293806	0.27323877
SIGLEC11	1.011988399	0.919149087	3.823612945	0.000203658	0.004293806	0.273111643
ABCG2	0.78327392	0.828349488	3.822741039	0.000204306	0.004299705	0.270159606
NOTCH3	1.396178792	1.962223653	3.82264174	0.00020438	0.004299705	0.26982344
TSPAN14	0.883237035	4.480868472	3.822195923	0.000204712	0.004300256	0.268314262
C11orf96	0.727068053	1.111018393	3.821675223	0.0002051	0.004300256	0.266551766
GRAP2	1.230104447	2.724727125	3.821421616	0.00020529	0.004300256	0.265693414
CD163	2.557231903	3.301821017	3.819824971	0.000206486	0.004316033	0.26029047
CD40	1.13142722	2.520841476	3.815880078	0.000209472	0.004355045	0.246948876
FMN1	0.908920817	0.923432447	3.81345356	0.000211328	0.004388957	0.238747846
FCRL3	1.053128028	2.253015056	3.811197335	0.000213069	0.004420384	0.231126078
PTK2	1.526932836	1.83990499	3.809550066	0.000214348	0.004437461	0.225563688
RBM38	1.122010742	5.400029529	3.807694469	0.000215798	0.004462726	0.219300112
SPATA24	-0.709873397	3.325065352	-3.793542184	0.000227165	0.004672974	0.171608775
FOXO1	1.321069476	3.636399279	3.791893927	0.000228525	0.004690838	0.166063543
PRSS57	-1.763755928	8.24163735	-3.791244969	0.000229063	0.004690838	0.163880778
CD226	0.924865557	1.700815825	3.79010381	0.000230012	0.004701706	0.160043222
SAMD3	0.889491678	1.553667006	3.788047311	0.00023173	0.004726905	0.153129827
CRTAM	0.790047372	1.59680086	3.785709193	0.000233699	0.004761615	0.145273333
PLEKHG5	0.759610685	0.831376425	3.778940451	0.000239488	0.004867781	0.122550918
ACHE	1.221058626	1.267677293	3.778584125	0.000239797	0.004867781	0.121355642
NUAK2	1.070612614	2.625451277	3.775786645	0.000242232	0.00491024	0.111974771
AKAP12	0.819183429	0.780056852	3.774288168	0.000243546	0.004926613	0.106952163
SH2B3	0.712729053	6.301788747	3.768906663	0.000248321	0.005012785	0.088927487
NIBAN3	1.379689085	2.725523976	3.766989813	0.000250043	0.005042321	0.082512192
GPBAR1	1.447136039	2.655034188	3.766647608	0.000250352	0.005043319	0.081367174
SLC26A11	0.991101457	1.902615127	3.764993237	0.000251849	0.005068234	0.075832836
G0S2	1.87992251	2.389957301	3.762949056	0.000253711	0.005100426	0.068997154

SCML4	1.017771839	1.861848547	3.762605814	0.000254025	0.005101466	0.067849652
EPB42	1.577451404	2.549048647	3.759647287	0.000256746	0.005147631	0.057962391
HS3ST1	0.816830139	0.693359458	3.750221784	0.000265599	0.005306533	0.026504212
CD2	1.259430769	4.226900591	3.748853229	0.000266909	0.005327224	0.021941823
PNOC	0.816627787	1.577597908	3.748007489	0.000267721	0.00533796	0.019123024
ETS1	1.120230094	4.65559004	3.747108526	0.000268587	0.005349743	0.016127391
DDIT4	1.665984286	4.442803153	3.746694967	0.000268986	0.005352217	0.014749474
CORO1C	0.703168333	5.828314827	3.744546085	0.00027107	0.005371705	0.007591676
ARHGAP24	1.001285864	1.541763167	3.743283107	0.000272301	0.005390624	0.003386304
GZMA	1.248206186	3.88794326	3.742060198	0.000273499	0.005408832	-0.000684571
TRPV4	0.73933679	0.482502313	3.741459589	0.000274089	0.005414998	-0.002683514
ERFE	1.291329103	1.611796673	3.733600795	0.000281922	0.005541615	-0.028815441
CCND1	1.206874262	1.447234427	3.730894465	0.000284668	0.00558719	-0.037804295
VSIG4	2.033952569	3.359857215	3.729974059	0.000285608	0.005589006	-0.040860161
MAFB	2.39273009	3.802632868	3.729816494	0.000285769	0.005589006	-0.041383235
TNFRSF13C	0.841537827	2.997215641	3.729182923	0.000286418	0.005591449	-0.043486348
ANGPTL4	0.608099054	2.059255711	3.728340128	0.000287283	0.005601732	-0.046283527
NREP	-0.816876002	5.67287383	-3.728003274	0.00028763	0.005602876	-0.047401381
SMIM30	-0.626062603	4.796155803	-3.727582369	0.000288063	0.005605711	-0.048798046
PLCXD2	0.6091148	1.339921643	3.725758525	0.000289949	0.005636776	-0.054848541
OASL	1.084941572	1.806095849	3.724783269	0.000290963	0.005648339	-0.058082921
RGS6	0.599804769	0.297173162	3.72426153	0.000291506	0.005650107	-0.05981296
TNFRSF17	1.014497444	2.567946785	3.722253679	0.000293606	0.005677397	-0.066468995
PDK4	1.707888026	2.195121961	3.721237103	0.000294675	0.005688872	-0.069837854
SYNE2	1.058525588	2.098377199	3.719305178	0.000296717	0.005722609	-0.076238073
PRDX2	1.445782287	5.652588735	3.71657175	0.000299628	0.005767329	-0.085289017
APOA2	1.287820853	2.219255462	3.713494499	0.000302939	0.00582528	-0.095472036
SPIB	1.261577988	2.103675986	3.712064303	0.000304489	0.005849309	-0.100202433
E2F2	0.900789735	3.898120268	3.709676651	0.000307093	0.005893528	-0.108096371
KCNK13	0.597652564	0.419936514	3.705127323	0.000312115	0.005978104	-0.123125841
MAL	1.311064365	2.271359421	3.704193425	0.000313155	0.005992133	-0.126209299
GZMB	1.404440543	2.868471129	3.697688412	0.000320493	0.006102547	-0.147669594
SETBP1	1.511254782	1.906421851	3.69594446	0.000322488	0.006134529	-0.153417806
PSAP	1.13599834	9.466183206	3.688099881	0.000331608	0.006295696	-0.1792472
HIST3H2A	1.450643874	1.330651854	3.686487739	0.000333512	0.00630777	-0.184549932
CD4	1.50790139	5.787242216	3.686463791	0.00033354	0.00630777	-0.18462869
VASN	0.616372691	2.30692119	3.68597364	0.000334121	0.006312624	-0.186240539
SLC15A3	1.63065556	2.565575558	3.684137038	0.000336307	0.006347755	-0.192278621
CARMIL1	1.079204219	1.908409503	3.683035949	0.000337624	0.006360298	-0.195897443
CYGB	0.997650458	1.090934801	3.682722475	0.000338	0.006361193	-0.19692754
CD3E	1.199988001	4.440541852	3.680344058	0.000340864	0.006396537	-0.204740895
NCR1	0.988965575	1.547804541	3.679323097	0.0003421	0.006413556	-0.208093611
ZFP36	1.136300739	8.546543736	3.674395975	0.000348127	0.006507742	-0.224263165
SLC6A9	1.114562231	1.754403924	3.669132098	0.000354677	0.006611133	-0.241518547
SNX25	0.953137497	1.524095317	3.666794493	0.000357623	0.006655648	-0.249174988
GPRC5D	0.911338254	1.01254227	3.66669478	0.00035775	0.006655648	-0.249501493
ITPR3	1.258758121	3.122575953	3.664099925	0.000361048	0.006704194	-0.257995716

KLRC1	0.604216507	0.662620506	3.663229557	0.000362161	0.006718446	-0.260843766
SLITRK6	0.84711052	0.26312341	3.657829839	0.000369137	0.006841344	-0.278500707
BMPR2	1.221894739	2.793791249	3.655036439	0.000372796	0.00690258	-0.287626791
FMNL2	0.989244112	2.221332519	3.65214563	0.000376619	0.006960111	-0.297065178
CTSL	1.588968118	3.106139485	3.651820445	0.000377051	0.006961489	-0.298126516
CD209	0.858297898	0.798878388	3.649007781	0.00038081	0.007024222	-0.307303305
PDE4A	1.254783869	2.198651345	3.647384794	0.000382995	0.007057836	-0.312595966
MCOLN2	1.04690305	1.520148551	3.64486872	0.000386405	0.007113954	-0.320797278
ICOS	0.874840167	1.592425246	3.644478146	0.000386937	0.007117015	-0.322069969
FZD5	0.681404788	2.284053745	3.643461084	0.000388326	0.007129081	-0.325383564
ICAM1	1.082990957	4.030184653	3.635477194	0.000399392	0.007297808	-0.351369103
TNIK	1.199964041	2.645393709	3.634411505	0.000400891	0.007311476	-0.354834158
PCDH12	0.727725182	1.872310533	3.630635779	0.000406247	0.007381474	-0.367104183
RASL11A	0.858324308	1.794539179	3.628924422	0.000408696	0.007412251	-0.372662197
MAST3	0.775106627	4.944478148	3.628568113	0.000409208	0.007414511	-0.373819124
ADD2	1.60140572	2.280084242	3.627331833	0.000410988	0.007439842	-0.377832576
GPR65	1.184242861	2.970233865	3.623602233	0.000416403	0.007530865	-0.38993361
ASAP1	0.814766849	5.167334825	3.620781855	0.000420543	0.007591624	-0.399077881
NUCB2	-0.874399214	6.633682321	-3.619970214	0.000421742	0.007606203	-0.401708324
FPR2	1.651915606	2.345868024	3.616915721	0.000426281	0.007672905	-0.411603326
PLS3	0.654197245	0.418768118	3.616686959	0.000426623	0.007672905	-0.412344125
ELL2	0.987409369	3.559657238	3.615697288	0.000428105	0.007685573	-0.415548534
FN3K	1.022041041	2.115165551	3.615688979	0.000428117	0.007685573	-0.415575433
THEMIS	1.000960747	1.927451001	3.615240013	0.000428791	0.007685903	-0.417028885
C5AR1	2.259406207	4.493231657	3.61515049	0.000428926	0.007685903	-0.417318679
GPR155	0.837948719	2.495819983	3.614870575	0.000429346	0.007686365	-0.418224765
ARPIN	0.924725492	1.133339315	3.614352848	0.000430126	0.007693238	-0.419900493
UNC5B	0.847257787	1.342354507	3.611738721	0.00043408	0.007756847	-0.428358671
ECE1	1.181182887	3.4831055	3.611335995	0.000434693	0.007760663	-0.429661276
FOS	1.256531104	9.732916802	3.606874798	0.000441531	0.007847373	-0.444082976
SECTM1	1.86241807	2.843133595	3.60415192	0.000445754	0.007907361	-0.452878093
PHOSPHO1	1.305362827	2.673896969	3.602970958	0.000447598	0.007932833	-0.456691018
LBH	0.907063599	3.92186704	3.600455553	0.000451548	0.007974884	-0.464809013
TACR2	0.600463765	1.520162	3.597884233	0.000455621	0.008038427	-0.473102698
F5	1.388510816	2.113048935	3.597067596	0.000456922	0.008054072	-0.475735717
ITGB5	0.968498543	1.690546027	3.59678391	0.000457374	0.008054755	-0.47665027
LY6G6F	0.812557037	0.637390553	3.59487198	0.000460436	0.00808672	-0.482812459
LCK	1.156790272	3.49076574	3.58910247	0.000469793	0.00821399	-0.501391532
UBXN10	0.723185482	0.80995906	3.588265562	0.000471165	0.008223498	-0.504084539
BAHCC1	-1.508705459	5.7857261	-3.588255295	0.000471182	0.008223498	-0.504117571
FCGRT	1.378755985	5.530985589	3.587038858	0.000473183	0.008243636	-0.508030906
DAB2	0.984651289	3.591693431	3.585964164	0.000474958	0.008259764	-0.511487349
RPH3A	0.695374849	0.383798903	3.58399637	0.000478223	0.008301718	-0.517813996
SH2D2A	0.912727159	2.551340453	3.582791456	0.000480233	0.008321765	-0.521686516
SLFN12L	0.893367528	2.794212902	3.577827559	0.000488599	0.008436674	-0.537628973
NPL	1.371034907	3.043714223	3.577177612	0.000489704	0.008448265	-0.539715061
PDZK1IP1	1.103848636	1.318574812	3.575411528	0.00049272	0.008492762	-0.545381973

OTUD1	0.909116877	4.231977993	3.566514534	0.00050818	0.008736032	-0.573895406
DENND2D	0.672092299	4.501240202	3.566244662	0.000508656	0.008736498	-0.574759394
RPGR	-0.645334867	3.702137322	-3.562526077	0.000515259	0.008842092	-0.586658931
BAG3	1.208461834	1.951949149	3.560489901	0.000518908	0.008896871	-0.593170429
ST3GAL4	-1.03226225	4.944078384	-3.558875793	0.000521818	0.008929513	-0.598330033
P2RY10	1.052809819	2.997399211	3.55860311	0.000522311	0.008929513	-0.599201494
CPM	1.442110009	2.211191747	3.557862692	0.000523653	0.008929513	-0.601567501
MPDZ	0.629687561	0.277272322	3.557778061	0.000523806	0.008929513	-0.601837913
PLXNA1	0.984960895	2.048759132	3.557658052	0.000524024	0.008929513	-0.602221359
UGCG	0.975705953	4.054802358	3.555498954	0.000527956	0.008972952	-0.609118138
AGPAT3	0.914668564	4.471736908	3.55319856	0.000532177	0.009013193	-0.616462488
TMEM117	0.671157142	0.890965032	3.552103365	0.000534197	0.009031572	-0.619957694
GYPA	1.774474668	2.575499878	3.548119658	0.000541608	0.009141096	-0.632663868
MARCO	1.741738895	1.82324443	3.546797087	0.000544089	0.009168572	-0.636879674
DEF8	0.712226823	4.13572961	3.546749912	0.000544178	0.009168572	-0.637030022
GOLGA8R	-0.819562665	2.416945118	-3.544616408	0.000548204	0.00922842	-0.64382796
PRDM8	-1.035391264	4.880075457	-3.543138878	0.000551009	0.009267619	-0.648533817
ITK	1.021498793	2.556901192	3.542533521	0.000552162	0.00927425	-0.650461384
KCTD12	2.118848705	4.685683084	3.541952826	0.00055327	0.009281587	-0.65231017
ANK1	1.737980527	3.008525855	3.541663045	0.000553824	0.009282875	-0.653232665
IGF2R	1.427304227	3.903582084	3.539521899	0.000557932	0.009343687	-0.66004693
ST6GALNAC	0.912169777	4.164345554	3.538367298	0.00056016	0.009372921	-0.663720081
GAD1	0.786111386	0.596627002	3.537646078	0.000561555	0.009383895	-0.666014013
B4GALNT3	0.616298302	0.664623168	3.537530085	0.00056178	0.009383895	-0.666382906
IER3	1.437051865	2.800883896	3.536782362	0.000563231	0.009393188	-0.668760666
KLF4	1.916738279	4.945466196	3.536560719	0.000563661	0.009393188	-0.669465409
PLPP4	0.657510435	0.429898546	3.536497482	0.000563784	0.009393188	-0.669666476
DAB2IP	0.647274676	0.763255359	3.536120544	0.000564518	0.009397362	-0.670864903
MYO1C	0.963748818	2.470039854	3.535408847	0.000565905	0.009412401	-0.673127367
ABLIM3	0.880731044	0.670950908	3.533183836	0.000570262	0.009468691	-0.680198212
KIRREL1	1.310633057	1.34460618	3.532199704	0.0005722	0.009492759	-0.683324512
CNN3	1.093224379	1.071809843	3.531214266	0.000574146	0.00951432	-0.686454247
SLC30A1	0.722430869	4.008707634	3.530191032	0.000576173	0.009530903	-0.689703261
MYCL	1.288771813	1.484927119	3.530047612	0.000576458	0.009530903	-0.690158595
PIF1	0.86087662	2.024954965	3.529475769	0.000577595	0.009541587	-0.691973938
FYN	0.930318446	4.568401008	3.527989549	0.000580558	0.00958241	-0.696690886
TEX14	0.902798788	0.999877762	3.525135003	0.000586291	0.009668825	-0.705746038
H2AFY	-0.636057131	7.146861833	-3.523766333	0.000589058	0.009706238	-0.710085583
CDH23	0.886467107	1.753270731	3.523026508	0.000590559	0.009722738	-0.712430716
CR1	1.614093788	2.757106434	3.520206511	0.000596313	0.009800894	-0.721365975
SLA	1.083469186	5.03414878	3.519059595	0.000598669	0.009831304	-0.724998337
VMO1	0.686238631	0.661809449	3.518701925	0.000599405	0.009835097	-0.726130904
RHAG	1.939533156	3.696758024	3.516781196	0.000603374	0.00987524	-0.732211301
TRIM10	1.329871587	1.824556382	3.513924744	0.000609322	0.009947493	-0.741248846
SGCB	0.731447123	1.269575067	3.513355753	0.000610514	0.009950248	-0.743048359
SEC14L3	0.631190624	0.538516862	3.511371911	0.000614685	0.010009848	-0.749320661
CDC14B	0.59721458	0.78589893	3.509289611	0.000619092	0.01006477	-0.755901127

PLSCR4	1.184816879	1.074272953	3.505087477	0.000628076	0.010181948	-0.769170896
HBQ1	1.013829729	2.156669687	3.504940568	0.000628393	0.010181948	-0.769634577
KMO	0.688417956	1.929862187	3.495563088	0.000648896	0.010453929	-0.799199192
HEG1	1.025059101	2.228791378	3.495152124	0.000649809	0.010453929	-0.800493359
TBC1D4	1.112828807	2.612872315	3.492205041	0.00065639	0.010537176	-0.809770333
CXCL1	1.242287282	1.723405149	3.491704365	0.000657514	0.010539726	-0.811345746
GYPB	1.37297884	1.941688228	3.490866496	0.0006594	0.010556001	-0.813981744
CEP85L	0.94384566	2.765529398	3.490771262	0.000659614	0.010556001	-0.814281325
IER5	0.655345398	4.234253417	3.49027673	0.00066073	0.010565166	-0.815836879
HMBS	0.947977107	4.260734582	3.489760343	0.000661897	0.010575134	-0.817460984
CDC42BPA	1.374780286	1.288579384	3.489092348	0.000663409	0.0105906	-0.819561623
KBTBD12	0.741784032	0.454089044	3.485278958	0.000672105	0.010703072	-0.83154722
VASH1	0.777776866	3.20983687	3.484368837	0.000674196	0.010718829	-0.83440616
TMEM246	0.737019452	0.390567612	3.482324579	0.000678915	0.01077625	-0.840825492
ALDH1A1	1.823681046	2.409832865	3.481147963	0.000681645	0.010810769	-0.844518865
ZBTB7B	1.035716331	5.450325155	3.480679338	0.000682735	0.010819243	-0.845989581
SLAMF1	0.839112103	1.968709657	3.479297577	0.00068596	0.010835046	-0.850325107
FCN1	2.313130483	5.620795074	3.475769614	0.000694257	0.010948336	-0.861388281
NEO1	1.127154628	1.480913362	3.475319003	0.000695324	0.010956278	-0.862800662
SLC10A4	0.716214912	0.780945414	3.475027078	0.000696016	0.010958305	-0.863715586
HSPA6	1.098287315	1.778903607	3.470028568	0.000707962	0.011119403	-0.879371554
FAM89A	1.202057981	2.909079038	3.466901658	0.000715533	0.011211168	-0.889155974
ST6GAL2	0.768488764	0.357480485	3.465177209	0.000719741	0.011268023	-0.894548832
ESPN	1.061767066	0.937020658	3.464558033	0.000721257	0.011273626	-0.896484636
CACNA1I	0.606005031	0.852321188	3.463016347	0.000725046	0.011323754	-0.901303354
CR2	0.767235366	0.946701954	3.461877296	0.000727858	0.011349445	-0.904862449
DNAJB9	0.594572451	4.05672557	3.459967466	0.000732594	0.011414161	-0.910827761
BACE2	0.969341545	1.160370937	3.458276258	0.000736813	0.011453299	-0.916107939
CD14	2.591026728	4.724960659	3.458252722	0.000736872	0.011453299	-0.916181405
FGFBP2	1.21275627	2.863592004	3.457566819	0.00073859	0.011470835	-0.918322267
KCNJ15	0.65046946	0.467367111	3.45575233	0.000743152	0.011523293	-0.92398401
AHSP	2.307007153	5.238932366	3.454786598	0.000745591	0.011551905	-0.926996378
SPACA6	0.743823312	1.564571633	3.453986581	0.000747617	0.011574081	-0.929491313
GADD45G	0.714670813	1.072707666	3.449741323	0.000758455	0.01170462	-0.942722572
RASSF5	0.643786392	5.323003895	3.446798281	0.000766054	0.011810745	-0.95188729
VOPP1	0.788105133	3.248072356	3.446609147	0.000766545	0.011810745	-0.952476037
DTX1	0.907718029	1.169510854	3.442450618	0.000777412	0.011968708	-0.965414187
RUBCNL	1.196223714	2.384711312	3.441270051	0.000780524	0.012003088	-0.969084848
ZBTB38	0.92772611	2.782506436	3.44113548	0.000780879	0.012003088	-0.969503195
GIMAP7	1.498650335	4.336643265	3.438941535	0.000786695	0.012054395	-0.976321693
KLRK1	0.613974507	1.024701002	3.434463079	0.000798693	0.012199812	-0.990228974
PID1	1.055450769	1.097646982	3.432785636	0.000803231	0.012259505	-0.995434193
FAM214B	0.642592957	4.26618798	3.425745266	0.000822542	0.01252478	-1.017257938
TSPO2	0.893812284	2.428792666	3.424931863	0.000824801	0.012549358	-1.019776929
SLC37A3	0.801426958	1.643015083	3.421771517	0.000833633	0.012663939	-1.029559352
TNFAIP6	1.110890057	1.692843978	3.419557433	0.000839874	0.01274841	-1.03640828
NUP62CL	0.676403481	0.630870889	3.419334726	0.000840504	0.01274841	-1.037096989

ITGB3	1.563637444	1.984805458	3.418664028	0.000842405	0.012767283	-1.039170852
IFITM3	1.874732049	5.681019218	3.416890946	0.000847448	0.012813781	-1.044651764
PTPRS	1.411741412	2.221992155	3.414972713	0.000852936	0.012866771	-1.050578707
DOCK9	0.887331716	1.427134182	3.414382699	0.000854631	0.01287478	-1.052401171
ALDH1L2	0.666287423	1.45914864	3.412079198	0.000861278	0.012922484	-1.059513836
LY9	0.876529669	2.740044819	3.408893999	0.000870548	0.013041473	-1.0693424
LILRA4	1.38768677	1.542147071	3.408654687	0.000871249	0.013041924	-1.070080536
FPR1	2.106145475	3.767089206	3.407021237	0.000876043	0.013093546	-1.075117617
BTG2	0.928279678	6.686992103	3.405762565	0.000879754	0.013138921	-1.078997624
ZC2HC1A	0.941993819	1.270022491	3.404862322	0.000882417	0.013168591	-1.081771997
ANKRD28	-0.841171304	6.217980677	-3.403800084	0.00088557	0.013195395	-1.085044823
CDCA7L	-0.79412285	5.333160083	-3.400722922	0.000894761	0.013301797	-1.094520972
SLC2A3	1.117003602	5.846480206	3.400222846	0.000896263	0.013313958	-1.096060286
EPSTI1	1.302853744	2.661008052	3.399727	0.000897755	0.013325946	-1.097586394
SGSH	1.284940865	2.999590106	3.399234777	0.000899238	0.013337788	-1.099101168
PDE2A	0.677530074	0.882376113	3.397699958	0.000903878	0.013386155	-1.103823272
CLEC3B	0.735070868	1.049696754	3.397473953	0.000904563	0.013386155	-1.104518461
EPN2	0.815008515	1.318538174	3.39627198	0.000908215	0.013407875	-1.108215069
CCR2	1.971483393	3.989666273	3.395943547	0.000909215	0.013407875	-1.109224957
TRIB2	1.123191106	2.834896625	3.395139534	0.000911668	0.013430017	-1.111696852
SERPINI1	0.749570445	1.826683583	3.394363932	0.00091404	0.013447539	-1.114080937
OTUD7B	0.621308907	1.132551937	3.393611977	0.000916345	0.013451305	-1.1163919
SULF2	1.521236163	3.940552996	3.393539021	0.000916569	0.013451305	-1.116616093
TUBB1	1.148965128	2.718755031	3.39257248	0.000919541	0.013474595	-1.119585857
C1orf115	0.642808336	0.77783333	3.391638935	0.00092242	0.01350125	-1.122453571
CBLN3	0.620224228	1.333940439	3.391532865	0.000922747	0.01350125	-1.122779362
SKAP1	1.074963741	3.108107698	3.390829673	0.000924922	0.013521192	-1.124938976
CREB3L4	-0.59749284	4.482782426	-3.388510364	0.000932129	0.013582977	-1.132059295
MS4A1	1.053608149	3.451825021	3.38838488	0.000932521	0.013582977	-1.132444416
MYO7A	1.14410236	1.324821171	3.387687752	0.000934698	0.013604511	-1.134583753
PM20D1	0.67132187	0.819011369	3.384158324	0.000945796	0.013755745	-1.145409178
ARHGAP31	1.051894277	2.588767677	3.379932534	0.000959245	0.013920144	-1.158358099
CCNA1	-2.066838286	4.5321583	-3.379140329	0.000961786	0.01394662	-1.160784119
CXCL10	1.097420765	1.240527128	3.37669904	0.000969657	0.014050164	-1.168257256
CPED1	1.02819319	1.204859892	3.371262338	0.000987403	0.014254345	-1.184883583
FBLN2	1.212060513	1.353629312	3.370421619	0.000990174	0.014273173	-1.187452644
IL32	0.958115164	2.878735507	3.369502558	0.000993212	0.014295782	-1.190260488
TMEM220	-0.6206788	4.264433752	-3.36839119	0.000996897	0.014331283	-1.193655001
LHFPL6	1.243181136	2.477433964	3.363224989	0.001014195	0.014553441	-1.209422136
ITLN1	0.720116457	0.657314997	3.363030341	0.001014852	0.014553441	-1.210015802
RMDN2	0.742392074	1.396508695	3.362556995	0.001016452	0.01456565	-1.211459363
PXDC1	0.784929303	1.090027699	3.360418396	0.00102371	0.01464808	-1.21797932
CD5	1.043258714	3.298699325	3.355657604	0.001040041	0.014849007	-1.232481118
TAC3	0.585919606	0.38512327	3.35494944	0.001042491	0.014873075	-1.234636784
SHTN1	1.402642083	1.810420318	3.350696484	0.001057319	0.015062536	-1.247574841
ABCA9	0.866140569	0.950686221	3.348470955	0.001065156	0.01515417	-1.254339727
KCNK6	0.984152347	3.561846924	3.348427919	0.001065309	0.01515417	-1.254470505

KIF21B	0.752152003	4.105214372	3.346904279	0.001070706	0.01521983	-1.259099657
MCUB	0.660550955	4.191990363	3.345648741	0.001075174	0.015272176	-1.262912929
PDCD1LG2	0.589697645	0.905833115	3.3436086	0.00108247	0.015364598	-1.269106616
APBB1	0.84567834	1.685131923	3.340547821	0.001093503	0.015499519	-1.278392932
CARD11	1.110865782	3.940988013	3.340529814	0.001093568	0.015499519	-1.278447546
SNX9	1.06305821	3.289884185	3.336430114	0.001108512	0.015654317	-1.290874704
TUFT1	0.606804115	0.949723865	3.33375488	0.001118367	0.015777489	-1.298977074
SLC6A19	0.820659199	0.570166379	3.332964409	0.001121294	0.01580042	-1.301370099
SNN	0.733324982	4.624059473	3.331731704	0.001125873	0.015847255	-1.305100965
PFKM	0.691382894	2.309973472	3.331468983	0.001126852	0.015847255	-1.305895958
COLEC12	0.690654534	0.570933345	3.331412861	0.001127061	0.015847255	-1.306065778
GIMAP4	1.560765681	4.412642555	3.33080925	0.001129312	0.015860064	-1.307892079
MSR1	1.280692904	1.175253943	3.328844061	0.001136669	0.015947791	-1.313836087
RARG	0.78477404	2.52109688	3.326072733	0.001147121	0.0160597	-1.322213385
IER5L	0.744121519	2.07073051	3.325513498	0.001149241	0.016072771	-1.323903155
CD247	1.01395645	2.978758849	3.325390834	0.001149707	0.016072771	-1.324273762
SH2D1B	0.783665112	1.413644902	3.324373019	0.001153576	0.016115287	-1.327348462
EHD4	0.926136531	3.221929073	3.323028284	0.001158707	0.016175352	-1.331409535
NRGN	1.623642762	5.738642237	3.320993206	0.001166513	0.016265409	-1.337552812
PFKFB2	0.987663023	2.222810876	3.32091074	0.00116683	0.016265409	-1.337801685
RAB39A	0.976977524	1.329752205	3.317220545	0.001181113	0.016405747	-1.348932961
RAB7B	-1.507457092	4.760865621	-3.315499582	0.001187829	0.016487273	-1.3541206
CD1D	1.864395199	3.613320376	3.314244092	0.001192752	0.016532014	-1.357903699
NR4A3	0.800943086	1.198829808	3.311990032	0.001201637	0.016631479	-1.364692718
MAP2K1	0.611725819	5.353249431	3.310222678	0.001208647	0.016692886	-1.370013109
DDIAS	0.596645357	2.71297586	3.306012147	0.001225502	0.016878226	-1.382678749
ASPHD2	0.630519116	2.319940704	3.305710632	0.001226718	0.016882552	-1.383585213
APBB2	0.603077263	0.709199818	3.302380311	0.001240217	0.017020187	-1.393592739
RUFY4	0.870898513	0.969611965	3.291551991	0.001285075	0.017583814	-1.4260729
JAZF1	0.971244132	3.56795389	3.288963147	0.001296021	0.017673842	-1.433824984
ANKRD65	0.719369142	0.55302206	3.288629547	0.001297438	0.017680775	-1.43482355
AQP1	1.174561002	2.461005056	3.287607658	0.001301787	0.017711759	-1.437881838
ZNF366	0.815068105	1.062885044	3.28745408	0.001302442	0.017711759	-1.438341393
CEACAM3	1.014384643	1.080493125	3.284919043	0.001313296	0.017834427	-1.445924445
ZNF883	0.81589071	0.768432294	3.284493095	0.001315128	0.01784685	-1.447198101
MYH10	0.898744568	3.055247773	3.282803026	0.00132242	0.017920813	-1.452250311
ABI3	1.308783009	3.334619586	3.281009834	0.001330198	0.017991041	-1.457608396
LILRB2	1.957391715	4.319628714	3.28096904	0.001330376	0.017991041	-1.457730259
LILRA6	1.621523964	2.622608198	3.280642697	0.001331796	0.017997741	-1.458705103
TGFBR2	0.649655598	5.97042516	3.279685815	0.001335969	0.018041603	-1.461562999
GNLY	1.393358923	3.891210421	3.278265209	0.001342186	0.018112998	-1.465804598
MYB	-0.65503303	7.576041513	-3.277833608	0.001344081	0.018125992	-1.467092951
RAB20	0.733407385	3.546054125	3.276607833	0.001349474	0.018186126	-1.470751177
SNX18	0.645813817	4.369589038	3.275938317	0.001352429	0.018213329	-1.472748804
SIDT1	0.841640907	2.609255884	3.27538179	0.001354889	0.018221245	-1.474409046
BACH2	0.92227492	2.173591671	3.274858794	0.001357205	0.018239787	-1.475969044
FCGR2B	0.968251191	1.712670385	3.273343375	0.001363937	0.018317607	-1.480488056

MXRA8	0.688362635	2.568351299	3.270654199	0.00137596	0.018453599	-1.488502896
CD83	0.864666325	4.630808514	3.270314464	0.001377485	0.01846134	-1.489515048
KLHDC8A	0.669716202	0.508178444	3.267972435	0.001388047	0.018567073	-1.496490111
SEPTIN1	0.79502808	3.677594121	3.267930554	0.001388237	0.018567073	-1.496614801
PELI1	1.089926532	4.575684199	3.266565935	0.001394427	0.018611483	-1.500676938
DTX4	1.178676818	2.660175077	3.26351006	0.001408381	0.018771983	-1.509768337
GJA1	1.41497863	1.241748959	3.257716978	0.001435193	0.019064063	-1.526983351
PLA2G7	0.97527814	1.0873972	3.254474042	0.001450409	0.019239917	-1.53660894
PLD2	1.078936421	2.516458522	3.253158118	0.001456626	0.019296083	-1.540512516
CTSB	0.939282302	6.799956979	3.250916036	0.001467276	0.019423945	-1.547160389
IL7	0.970473843	1.025038355	3.249440677	0.001474324	0.019477494	-1.55153278
NLRP12	1.643230845	3.048773692	3.244420527	0.001498542	0.019757204	-1.566397977
TRPC1	0.71402092	0.771063051	3.24353453	0.001502855	0.01978725	-1.569019491
TFEB	1.015243169	3.251001517	3.242950335	0.001505705	0.019811368	-1.57074769
MS4A4A	1.698998792	3.116552624	3.239959993	0.001520371	0.019932416	-1.579589776
KAZALD1	0.605641161	0.555633902	3.239529355	0.001522494	0.019932416	-1.580862552
PIM2	0.655337625	6.202352409	3.239016504	0.001525026	0.019932416	-1.582378129
MARCKS	1.425099923	3.66690323	3.238842494	0.001525886	0.019932416	-1.582892315
THEM5	0.654567545	0.973665242	3.238784797	0.001526172	0.019932416	-1.583062801
NACC2	1.14497466	3.554447265	3.230785568	0.001566204	0.020373212	-1.606674354
NLRC5	0.787028097	5.347379889	3.228204808	0.001579326	0.020491832	-1.614281505
LSR	0.687839219	2.894887798	3.228095824	0.001579883	0.020491832	-1.614602636
HEPACAM2	0.603801777	0.563385651	3.227549074	0.001582677	0.020491832	-1.61621355
TCEAL4	-0.65827051	4.858847369	-3.227448064	0.001583194	0.020491832	-1.616511136
SLFN14	0.783741536	1.50921734	3.227106378	0.001584943	0.020493679	-1.617517716
LGALSL	0.924765336	1.432622276	3.225705207	0.001592134	0.020545709	-1.621644513
EBF1	0.990861013	1.318784984	3.221824406	0.00161221	0.020726983	-1.633066507
KIAA0513	1.198332211	3.393287331	3.221099867	0.001615985	0.020757137	-1.635197681
OAT	0.863028903	5.072695003	3.220734785	0.00161789	0.020767897	-1.636271389
AVPR1A	0.637193915	0.464614735	3.220418298	0.001619543	0.020775412	-1.637202093
TNFRSF1B	1.564441249	6.132103482	3.219843283	0.00162255	0.020800279	-1.638892862
GAS6	0.950606954	3.536834526	3.218452591	0.001629845	0.020880041	-1.642980982
HOOK1	0.839764733	1.070075763	3.217474431	0.001634994	0.020918466	-1.645855512
SLC8A1	1.107807823	1.386920272	3.215815699	0.001643761	0.021013389	-1.650728353
ITGAE	-0.631293664	3.486924252	-3.215458753	0.001645653	0.021013389	-1.651776671
SLC1A3	1.106335185	2.854774884	3.21472285	0.00164956	0.021048264	-1.653937641
CD6	1.004186091	3.031255374	3.214537233	0.001650547	0.021048264	-1.654482636
NFKB2	0.8358511	4.401280987	3.213973237	0.00165355	0.02107274	-1.656138438
FRMD6	0.81032946	0.924646404	3.212896762	0.001659294	0.021132106	-1.659298108
CCR5	1.301288461	2.615431007	3.211097949	0.001668934	0.021238367	-1.664575984
HS3ST4	0.59584542	0.266713276	3.210462259	0.001672353	0.021242819	-1.666440555
PIK3R3	0.67086485	1.218347109	3.209917057	0.001675291	0.021266254	-1.668039461
GBGT1	0.655508306	4.621746009	3.208616197	0.00168232	0.021299902	-1.671853542
GIMAP5	0.75334605	1.573096694	3.208256958	0.001684266	0.021310674	-1.672906591
FFAR2	1.141385746	1.744588345	3.205273442	0.001700508	0.021474294	-1.681648377
MEIS2	0.6004984	0.297071423	3.201900982	0.001719043	0.021638148	-1.691521464
TXNDC5	0.711004227	3.136883338	3.200987544	0.001724095	0.021670127	-1.69419409

SH2D3A	0.590900746	1.500748546	3.200706302	0.001725654	0.021670127	-1.695016845
KIF21A	0.660743181	0.891016642	3.20063703	0.001726038	0.021670127	-1.695219485
SMIM1	0.919144879	2.062667795	3.196320019	0.001750129	0.021944273	-1.707840671
MYCT1	1.487357733	1.279316527	3.195839914	0.001752828	0.021963956	-1.709243407
CCR4	0.796000932	1.590933421	3.195601498	0.001754169	0.021966621	-1.709939929
PF4	2.013375691	3.410909156	3.194447709	0.001760674	0.022033906	-1.713310042
KLF1	1.404123452	3.720762642	3.191693731	0.001776293	0.022215079	-1.721349977
PPM1N	0.735531598	1.701845403	3.190235625	0.001784614	0.022290499	-1.725604369
HIST4H4	0.59174868	1.629331204	3.188801312	0.001792835	0.022378819	-1.729787725
FGD2	1.537399029	3.63376478	3.1884932	0.001794606	0.02238656	-1.730686163
STK26	-0.632828081	6.802646228	-3.185194205	0.001813666	0.022516423	-1.740301235
BCL11B	0.951406942	2.456202607	3.182982844	0.001826547	0.022634051	-1.746741601
TAGLN	0.7303341	3.701632326	3.18286433	0.00182724	0.022634051	-1.747086653
ITGAD	0.697996243	0.784284533	3.18237178	0.001830122	0.022655326	-1.748520592
SESN2	0.714887117	3.667362942	3.181011602	0.001838102	0.022739643	-1.752479436
CD28	0.906180401	2.137424488	3.179855015	0.001844913	0.022794919	-1.755844589
CD3G	0.946809702	2.593186574	3.179349382	0.001847897	0.022817312	-1.757315425
SPOCK2	1.053620434	4.148595906	3.178155484	0.001854963	0.022868935	-1.760787572
NEDD9	0.76181378	3.128009152	3.178047462	0.001855604	0.022868935	-1.761101671
TAL1	1.541710681	3.125007829	3.174430791	0.001877168	0.023090833	-1.771612746
IVNS1ABP	0.726798746	5.6964934	3.1718421	0.001892746	0.02325306	-1.779129953
MX1	1.309359613	3.124276432	3.167603865	0.001918511	0.023512961	-1.791425927
GPRIN3	1.043209986	2.606896478	3.165697239	0.001930207	0.0235793	-1.796952864
VNN3	1.182731091	1.52826499	3.160662701	0.00196141	0.023885511	-1.811533354
FAM110A	0.707128173	3.992871358	3.15992054	0.00196605	0.023927033	-1.813681046
LMO4	1.029112326	4.166863596	3.157606266	0.001980582	0.024058752	-1.820375413
DUSP1	0.995483226	9.045362733	3.15628535	0.001988921	0.024129925	-1.824194473
LLCFC1	0.767152301	1.095431206	3.155491287	0.00199395	0.024175862	-1.826489629
PDE4B	1.044357843	3.808307207	3.154329176	0.00200133	0.024235152	-1.829847698
MICAL2	0.993955768	3.008725041	3.150472162	0.002026009	0.024518743	-1.840985471
PLEKHO2	0.634384188	5.426521826	3.149667067	0.002031196	0.024566236	-1.843308847
SIGLEC9	1.690600035	3.413608415	3.149159852	0.00203447	0.024590551	-1.84477233
C17orf99	0.686525439	1.031174885	3.147584119	0.002044673	0.024695982	-1.849317554
STX11	1.301668874	5.098080974	3.147421104	0.002045731	0.024695982	-1.849787661
TSPAN9	0.711559612	0.885985061	3.145413356	0.002058806	0.024776971	-1.855575965
GSTA4	0.775510164	1.410972307	3.142455501	0.002078209	0.024964171	-1.864097662
GJA4	0.850433342	1.303515301	3.136974665	0.002114613	0.025276641	-1.879870041
KLRG1	0.827095103	3.289808363	3.136398032	0.002118477	0.025307286	-1.881528069
GRK5	0.753873715	2.131961609	3.13541452	0.002125083	0.025357556	-1.884355416
SYNE1	0.648631442	2.596530077	3.129225975	0.00216709	0.025714373	-1.902128503
CCDC50	1.25187565	2.734793043	3.1287722	0.0021702	0.025735587	-1.903430531
PPP1R16B	1.032388451	3.699873765	3.127279602	0.00218046	0.025841506	-1.907712143
VDR	1.168538964	2.954289216	3.126536284	0.002185586	0.025886493	-1.909843746
EBF3	1.020383684	1.144859796	3.125366449	0.002193676	0.025934959	-1.913197584
AGT	0.761253221	0.696443999	3.123365106	0.002207581	0.026067678	-1.918932808
QPCT	1.364824816	2.227826729	3.119399279	0.002235377	0.026331983	-1.930288335
ARHGEF10L	1.465166919	2.02562405	3.117101241	0.002251632	0.0265013	-1.93686275

CHI3L1	1.463492283	1.964997313	3.116334219	0.002257081	0.026539457	-1.939056183
TRIP10	0.778527741	2.246675249	3.115153678	0.002265493	0.02660621	-1.942431247
C5AR2	0.926087413	1.765420941	3.113500178	0.002277323	0.026696813	-1.94715661
RNF144B	1.114629349	3.712354852	3.110599987	0.002298212	0.026909266	-1.95543957
DUSP10	-0.696013773	5.57023297	-3.109584128	0.00230557	0.026962979	-1.958339301
DEPDC1	0.796485222	2.763156459	3.109176307	0.002308531	0.026981384	-1.959503183
CPOX	0.585162141	4.672420234	3.10860009	0.002312719	0.027014114	-1.961147428
CAVIN1	1.697496739	2.7429152	3.108097225	0.00231638	0.02703517	-1.962582147
BLK	0.950319212	2.240274317	3.107125793	0.002323468	0.027055418	-1.965353168
CDKL5	0.712616924	1.30364299	3.106885296	0.002325226	0.027055418	-1.966039072
TM7SF3	-0.753776097	7.259520097	-3.105642128	0.002334332	0.027136262	-1.969583902
ZNF318	0.644340918	4.346796393	3.103321827	0.002351417	0.027302248	-1.976196864
RBM47	1.626028248	3.442842442	3.100058541	0.00237564	0.027548688	-1.985490197
CDC42EP2	0.700431794	2.778643968	3.09781969	0.002392393	0.027695403	-1.991861242
TLR5	1.372381692	2.382632377	3.095754129	0.002407946	0.027842327	-1.997735656
GPR84	0.900553038	1.736992922	3.094814614	0.002415052	0.027905756	-2.000406503
IFI30	1.275352248	2.217785321	3.094649791	0.0024163	0.027905756	-2.000874989
GNGT2	0.740446002	1.510502407	3.089349516	0.002456768	0.028285978	-2.01592884
SIDT2	0.710952762	4.685085183	3.088712858	0.002461671	0.02831213	-2.017735585
KIAA0040	0.677808041	4.660755946	3.087820347	0.00246856	0.028357836	-2.020267868
NR1I2	-0.815749617	2.050262322	-3.0875058	0.002470992	0.028369027	-2.021160168
PLAG1	1.031342859	1.365224045	3.086832902	0.002476202	0.028412081	-2.023068768
ANKRD55	0.647992485	1.17148588	3.083115954	0.002505165	0.028696205	-2.033605031
EVA1C	0.772304708	1.7075997	3.082899304	0.002506863	0.028696205	-2.034218821
SMAD7	0.720112867	1.860028218	3.082586286	0.002509318	0.02870742	-2.035105566
RIN2	1.106767679	2.534822554	3.080113319	0.002528791	0.028913198	-2.04210849
TEC	-0.728334959	5.699855455	-3.079280613	0.002535379	0.028954503	-2.044465451
XKR6	0.758712774	0.924243137	3.077737333	0.002547631	0.0290756	-2.04883223
SPTA1	1.337578256	3.223408277	3.073927373	0.002578114	0.029287862	-2.05960461
FMNL3	0.804409097	3.286079646	3.069818336	0.002611368	0.029562491	-2.071209736
SLC7A7	1.501959979	3.926749247	3.068661502	0.002620802	0.029634401	-2.074474564
RASGRP1	0.800120627	3.265602154	3.06623563	0.002640686	0.02982458	-2.08131744
MCOLN1	0.644421459	4.216811789	3.063280645	0.002665096	0.03006537	-2.089646534
P2RY12	0.854545803	1.396756718	3.055789281	0.002727916	0.030649681	-2.110731114
MEFV	1.689394496	3.011967381	3.053267789	0.002749367	0.03085506	-2.117817886
AMMECR1	0.593204028	3.605529054	3.048209111	0.002792872	0.031307192	-2.13202032
SGPP1	1.235875527	2.760898048	3.047387353	0.002799999	0.031327014	-2.134325519
CD48	1.163573967	5.177797106	3.047305028	0.002800714	0.031327014	-2.134556428
SIRPB1	1.626707644	3.958171753	3.046569029	0.002807113	0.031376457	-2.136620554
FLOT1	0.863833892	5.537973537	3.046157412	0.002810697	0.031398489	-2.137774754
CEBPB	1.086134313	5.441123409	3.044573043	0.002824534	0.031480778	-2.14221617
LONRF3	1.000023705	2.665267586	3.043131189	0.002837181	0.031578675	-2.146256342
S1PR3	1.079360765	1.708505766	3.041132734	0.002854797	0.031672949	-2.151853418
RHOU	1.127298447	3.456651518	3.036789633	0.002893429	0.031974786	-2.164006198
EPHX3	0.610095291	1.258684249	3.036565507	0.002895436	0.031977968	-2.164632935
FAM178B	1.050793221	1.24756625	3.034636697	0.002912758	0.032132806	-2.170024937
AXL	0.757584525	1.182982267	3.030880278	0.002946768	0.03241174	-2.180517537

S100A8	2.480451133	7.835949218	3.030503437	0.0029502	0.032417222	-2.181569527
CD8B	0.803476341	2.054062153	3.027804945	0.002974885	0.032633178	-2.189099331
CRISPLD2	1.231961301	2.270341399	3.02736456	0.002978931	0.032651893	-2.190327622
ST14	1.373942271	2.721226034	3.024319952	0.003007046	0.032856322	-2.198815189
SLC45A4	0.636195953	2.772906	3.023295392	0.003016562	0.032941801	-2.201669731
CXCR4	0.907548926	8.165035875	3.022794025	0.003021228	0.032974259	-2.203066292
HELZ2	0.924414991	3.37990549	3.021459275	0.003033685	0.033049502	-2.206783272
CDKN2D	0.78662516	4.977938426	3.021146118	0.003036614	0.033049502	-2.20765514
CLEC17A	0.693739794	1.923756282	3.020557828	0.003042124	0.033090964	-2.209292797
YPEL4	0.941752234	1.848303647	3.018417329	0.00306225	0.033235579	-2.215249102
DUSP4	0.646360087	0.763983904	3.017874375	0.003067375	0.033254055	-2.216759386
SLC2A6	1.068466468	3.273328709	3.017693999	0.003069079	0.033254055	-2.217261067
ARHGEF10	0.886229471	1.4511076	3.016873335	0.003076844	0.033319637	-2.219543263
MME	0.848350481	0.802934355	3.014986172	0.003094769	0.033457889	-2.224789262
CDK6	-0.820127172	7.442171529	-3.013975853	0.003104404	0.033520773	-2.227596613
CUEDC1	0.932852287	2.087713464	3.01349552	0.003108995	0.033537165	-2.228931017
ANKH	0.649444821	3.63294092	3.012942442	0.003114289	0.033575658	-2.230467286
ALAS2	1.963727027	4.670875098	3.011708037	0.003126134	0.033666059	-2.233895176
C9orf43	-1.120290009	3.183069606	-3.010773059	0.003135134	0.033744302	-2.23649076
TNFRSF11A	0.803773908	1.094071037	3.008642747	0.003155728	0.033890984	-2.242402092
SIGLEC7	1.278508585	2.466187909	3.007557517	0.003166267	0.033985414	-2.245412068
COL6A5	0.617997606	0.326106479	3.001677553	0.003223936	0.034566279	-2.261704276
LGR6	0.867026187	1.24566232	2.996395583	0.003276564	0.035014794	-2.276315995
KDM5B	-0.70792611	5.705007534	-2.996072475	0.003279809	0.035023647	-2.277209097
ITGAM	1.389506515	5.560710542	2.995498504	0.003285581	0.035034192	-2.278795402
SLC4A1	1.927707247	4.515279185	2.99388117	0.003301894	0.035150362	-2.283263871
CETP	0.998309484	2.145837103	2.991025006	0.003330886	0.035381572	-2.291149943
EFCAB2	-0.665374971	3.650141132	-2.990821771	0.003332958	0.035384266	-2.29171084
KEL	1.347988156	2.719384862	2.990266914	0.00333862	0.03540575	-2.293241993
MAML2	0.902811835	3.097252776	2.989144015	0.003350107	0.035469576	-2.296339925
CPVL	1.546766374	4.473917581	2.988658749	0.003355082	0.035500948	-2.2976784
BEX5	0.635742309	0.681796702	2.985062518	0.003392166	0.035798019	-2.307591743
EGR3	1.170392361	2.223354785	2.983252324	0.003410975	0.035938044	-2.312577791
SOD2	0.713564266	4.396461445	2.982711239	0.003416615	0.035977996	-2.314067658
MYO1D	1.015969281	1.839841765	2.98252386	0.003418571	0.035979118	-2.31458355
SLC31A1	0.618631925	4.050980923	2.981636754	0.003427842	0.036038762	-2.317025541
LAG3	0.950432748	1.672892815	2.981627252	0.003427942	0.036038762	-2.317051694
DMTN	1.225685556	3.344927597	2.980825723	0.003436339	0.036107537	-2.319257565
FOLR3	1.128759656	1.401628761	2.979697534	0.003448191	0.036198721	-2.322361554
SLC7A8	0.801811385	1.595295688	2.979645579	0.003448737	0.036198721	-2.322504474
NADK	0.670391848	5.62475828	2.970569441	0.003545474	0.03699542	-2.347438155
IFIT2	0.902161401	2.80014252	2.969218289	0.003560087	0.037087413	-2.351144342
MGST1	-1.204565726	5.64099004	-2.966949985	0.003584743	0.037304333	-2.357362966
PPP1R13B	0.656097587	1.368983976	2.965680684	0.003598609	0.037348763	-2.36084099
MCEMP1	1.320063531	3.912467619	2.964200896	0.003614836	0.037497183	-2.364894135
AANAT	0.669334266	1.678927891	2.962804762	0.003630208	0.037576516	-2.368716541
KLHDC8B	0.9621955	2.952325694	2.962606559	0.003632395	0.037579166	-2.369259063

PFKFB3	0.652635954	4.720387861	2.959558517	0.003666183	0.037830772	-2.377598212
KCTD17	0.775933227	2.526598002	2.959550486	0.003666272	0.037830772	-2.377620175
RAB29	0.701816733	4.251233131	2.959535807	0.003666436	0.037830772	-2.377660315
CACNA2D3	1.059942866	1.810206296	2.958357973	0.00367957	0.037926076	-2.380880734
BEST1	0.688073128	2.276921244	2.956537116	0.00369996	0.038106687	-2.385857099
NELL2	0.739561123	1.48520292	2.956443684	0.003701009	0.038106687	-2.386112377
CCR1	1.532456293	4.37543277	2.954419117	0.003723808	0.038298142	-2.391642194
TRAF1	0.6488739	3.385148349	2.954020512	0.003728312	0.038298142	-2.392730537
CDC20	0.817229385	4.866352987	2.953840028	0.003730353	0.038298142	-2.393223287
FCGR3A	1.568152692	4.109797777	2.952752923	0.003742669	0.038393427	-2.396190692
PRF1	1.055129089	4.424196056	2.951389001	0.003758173	0.03853216	-2.399912361
FCMR	0.941509689	4.768723256	2.946515205	0.003814058	0.038931293	-2.413199024
MAPK7	0.702147869	3.361176524	2.94636361	0.003815809	0.038931293	-2.413611986
CLCN3	0.603762194	4.001851367	2.94625119	0.003817107	0.038931293	-2.413918218
DNAJA4	0.867227711	3.723927255	2.944105815	0.003841967	0.039102863	-2.419760297
DOK2	1.053539874	4.973141898	2.942171533	0.003864508	0.039270664	-2.425024363
ADAP2	1.313423735	3.31793606	2.941009792	0.003878104	0.039326686	-2.428184543
S100A12	2.391836616	5.358097062	2.94053374	0.003883689	0.03934269	-2.429479193
ZFPM2	0.586164317	0.334508584	2.94047102	0.003884425	0.03934269	-2.429649748
LRRK2	1.393361176	2.52631382	2.940006499	0.003889882	0.039364068	-2.430912836
LGALS3	1.308064779	4.435377141	2.938552845	0.003907005	0.039496264	-2.434864373
CD36	1.569326412	4.978541522	2.936007362	0.003937155	0.039745273	-2.441779774
CYP27A1	1.408392826	1.991508029	2.935956111	0.003937764	0.039745273	-2.441918956
CXCL8	1.485817792	4.8988748	2.935310735	0.003945443	0.039760913	-2.443671413
SESN3	1.030240902	4.774595973	2.9346789	0.003952974	0.039795594	-2.445386778
PSAT1	0.943760161	1.911364149	2.934317173	0.003957292	0.039818462	-2.446368682
CREB5	1.045289136	2.288509032	2.931242644	0.003994166	0.040091338	-2.454710198
RESF1	0.652331739	5.944809479	2.93012991	0.004007589	0.040199835	-2.457727279
S100A9	2.453067574	9.319288163	2.924315578	0.004078404	0.040825995	-2.47347607
ACCS	-0.845715483	5.216566894	-2.922979167	0.004094842	0.040943924	-2.477092036
SLC37A2	1.153117982	2.676983118	2.921299473	0.004115589	0.041029383	-2.481634782
PPM1M	0.748571937	4.674538714	2.920628284	0.004123906	0.041091248	-2.483449379
TP53I11	1.024410802	3.766295208	2.919115473	0.004142709	0.041257479	-2.487538018
FKBP10	0.760346047	0.984147583	2.918924007	0.004145094	0.041260119	-2.488055355
BASP1	1.542782248	4.435116186	2.916849171	0.004171024	0.041475796	-2.493659632
STAT4	0.900774111	3.034701278	2.915746559	0.004184865	0.041577158	-2.496636448
FKBP15	0.599270548	4.845502874	2.913773055	0.004209742	0.041761801	-2.50196203
HLA-DOB	0.728196174	3.044809889	2.91359835	0.004211951	0.041761801	-2.502433325
IRF8	1.558631099	5.103193362	2.912689436	0.004223459	0.041811206	-2.504884879
PTPRM	1.25883449	1.77664404	2.912639529	0.004224092	0.041811206	-2.505019469
OPTN	0.951466544	3.244693529	2.910188635	0.004255273	0.042055671	-2.511626668
TICAM1	0.587962215	4.707214636	2.905286309	0.004318277	0.042591824	-2.524827924
TMIGD3	0.738021496	1.44600248	2.902049882	0.004360339	0.042876295	-2.533532486
BCL6	1.233186173	4.502893014	2.901382268	0.004369062	0.042940374	-2.535327017
SPON1	1.306060885	1.581908325	2.897703912	0.004417411	0.043328035	-2.545207873
PTPRJ	1.119126585	4.429822461	2.896877812	0.004428337	0.043407241	-2.547425445
OSBPL11	0.675349725	4.487273237	2.896587814	0.004432178	0.043407241	-2.548203783

TLDC2	1.141412641	3.688747872	2.895286526	0.004449453	0.043532603	-2.551695516
C19orf38	1.153025691	4.756748242	2.894947701	0.004453961	0.043554811	-2.552604455
HP	0.914557965	1.695769517	2.893531361	0.00447285	0.043646908	-2.556402957
GIMAP1	0.856150653	3.270798128	2.892671641	0.004484352	0.043720182	-2.558707857
VPREB3	0.860759077	2.037533199	2.891986751	0.004493534	0.04376585	-2.560543611
KIAA1549	-0.891549889	2.775702783	-2.886667967	0.00456543	0.044288766	-2.574786922
SHISA4	0.584977091	1.070552444	2.88338203	0.004610372	0.044680201	-2.583574939
RAP1GAP	0.736698081	1.130749054	2.882849011	0.004617701	0.044720467	-2.584999635
PIK3R5	0.737716174	4.979591384	2.882745614	0.004619123	0.044720467	-2.585275976
CTSH	0.936232205	4.45395517	2.880078444	0.004655964	0.04502728	-2.592401311
TTC39B	0.694511945	1.80721119	2.879523778	0.004663659	0.045061972	-2.593882372
PLCL2	0.717132914	4.307011269	2.879064031	0.004670047	0.045101293	-2.595109792
THEMIS2	0.915172777	6.067769506	2.87874055	0.004674545	0.045122348	-2.595973311
BLVRA	1.121792479	4.257384028	2.878439228	0.00467874	0.045140443	-2.596777601
PPP1R14A	0.884043023	1.151369925	2.874320179	0.004736421	0.045634127	-2.607764769
RRAS	0.928433347	4.442522319	2.874102092	0.004739493	0.04563609	-2.608346112
IL1RN	1.385908703	3.106985169	2.873331748	0.004750359	0.045718096	-2.61039926
PPFIBP2	0.746538204	1.726621015	2.873035263	0.004754547	0.045735784	-2.611189336
SNAI1	1.035006011	2.649897899	2.871971426	0.004769603	0.045829315	-2.614023665
CD22	0.927944354	2.867270207	2.870812599	0.004786052	0.045947992	-2.617110022
VAV2	0.863117344	3.616443724	2.869982829	0.004797863	0.046032145	-2.619319316
GAS2L3	0.724324874	1.697978699	2.866000732	0.004854914	0.046471529	-2.629913994
CFH	1.167908065	1.901724829	2.865649987	0.004859968	0.046497042	-2.630846562
MTSS1	1.100695578	4.09110557	2.865331123	0.004864568	0.046518176	-2.631694274
TMOD1	0.894506754	2.086981894	2.863530195	0.00489062	0.046698462	-2.63648056
LTA4H	0.677768769	6.13045807	2.860228156	0.004938719	0.047088425	-2.645249452
RASGEF1B	0.898593629	2.921523395	2.859948332	0.004942815	0.047104399	-2.645992143
GOLGA8O	-0.763835778	2.147618627	-2.857605206	0.004977234	0.047386238	-2.652208621
EHBP1L1	0.665152149	6.17191974	2.855441269	0.005009216	0.047643866	-2.657945721
MS4A6A	1.841031925	4.821041588	2.854533262	0.005022692	0.047725384	-2.660351922
RHOC	0.828340712	3.530700423	2.853020171	0.005045222	0.047892688	-2.664360086
TNFRSF19	0.654793757	0.881878075	2.852514465	0.005052772	0.047940976	-2.665699283
FCRL2	0.693121845	1.672319614	2.852348031	0.005055259	0.0479412	-2.666139982
OSBP2	0.93280718	2.84660978	2.85190008	0.005061959	0.047981354	-2.667325997
ALDH2	1.058520252	2.725193719	2.85145993	0.00506855	0.047982091	-2.668491196
FGD6	0.817798898	1.984201415	2.847807513	0.005123546	0.048354976	-2.678154073
TC2N	1.010748637	2.809561511	2.847794755	0.005123739	0.048354976	-2.678187807
SETD7	0.840389115	3.020349159	2.846122563	0.0051491	0.048523727	-2.682608126
TLR7	1.379833879	2.431583542	2.845130681	0.005164198	0.048642447	-2.685229015
GIMAP8	1.227658244	3.311034213	2.842835909	0.005199283	0.048901904	-2.691289501
PLK1	0.714874704	4.240948878	2.842344072	0.005206831	0.048925602	-2.692587882
MMP17	1.17412032	2.787543631	2.841189688	0.005224586	0.049068742	-2.695634517
LRRC25	1.553103475	4.601091751	2.838986761	0.005258621	0.04932062	-2.701445429
LY96	1.174874366	3.912163435	2.83896183	0.005259008	0.04932062	-2.701511169
KCTD7	0.727664978	1.999590188	2.836677852	0.005294512	0.04953423	-2.707531646
CXCR3	0.829212766	2.279373201	2.83330883	0.005347283	0.049836266	-2.716404469
PC	0.70873857	1.578331633	2.832781162	0.005355591	0.049865937	-2.717793319

PRDX4	0.770479294	4.772674609	2.832446992	0.005360859	0.049891114	-2.718672756
SRGAP2	0.690557661	3.871591001	2.83184326	0.005370388	0.049920665	-2.720261367
COTL1	0.924545358	6.377000959	2.83163	0.005373758	0.049920665	-2.72082245
SIGLEC14	1.128126249	4.806286001	2.831596006	0.005374296	0.049920665	-2.720911885
