# Supplementary material for: CD86 Is Associated with Immune Infiltration and Immunotherapy Signatures in AML and Promotes Its Progression
Source: J Oncol. 2023 Apr 7;2023:9988405. doi: 10.1155/2023/9988405 (PMC10104747; doi:10.1155/2023/9988405)
Supplement: Supplementary Materials — Supplementary Figure 1. CD86 mRNA expression in various cancers. Supplementary Figure 2. OS survival and prognosis of CD86 in high- and low-expression group in various cancers. Supplementary Figure 3. CD86 and univariate analysis in various cancers. Supplementary Figure 4. Heat and volcano maps showed the high and low expression of CD86, immune score and matrix score. A. Gene heat map and volcano map of CD86 high- and low-expression group; B. Gene heat map and volcano map of the high- and low-expression immune score group; C. Gene heat map and volcano map of high- and low-expression group with matrix score. Supplementary Figure 5. The PPI network, GO and KEGG analyzed of Mcode2. A. the PPI network diagram of Mcode2; B-C. Enrichment of the top ten of Go analysis; D-E. KEGG was used to analyze the top ten enrichment pathways. Supplementary Figure 6. The PPI network, GO and KEGG analyzed of Mcode4. A. the PPInetwork diagram of Mcode4; B-C. Enrichment of the top ten of Go analysis; D-E. KEGG was used to analyze the top ten enrichment pathways. Supplementary Figure 7. IRS different risk groups scores of survival prognosis in GEO data set were correlated with GSE146173, GSE106291, GSE37642 (the GPL96 platform), GSE12417 (the GPL97 platform), And GSE12417 (the GPL96 Platform) OS curve and ROC curve. Supplementary Table 1. Chemokines, immunostimulators, MHC proteins, and receptors in TISIDB website. Supplementary Table 2. Differences in expression of chemokines, immune stimulators, MHC proteins and immune receptors between CD86high-expression group and low expression group. Supplement Table 3. Differences between CD86 high- and low-expression group and CD8+ T cell recruitment, macrophage, NK cell, and Th1. Supplementary Table 4. Differential genes in StromalScore, ImmuneScore and CD86 high- and low-expression groups. Supplement Table 5. 65 genes screened out by Cox regression analysis. [file 9988405.f1.zip › 9988405.f1/Supplementary table 5 (1).pdf]

Supplement Table 5:65 genes screened out by COX regression analysis

	p.value	HR	Low 95%CI	High 95%CI
TLR7	0.0246979	1.2202144	1.0256783	1.4516474
FGD2	0.0287049	1.2154253	1.0205016	1.4475808
MS4A4A	0.0072583	1.2647508	1.0654617	1.5013158
MS4A6A	0.0163999	1.206692	1.035041	1.4068095
IL1RN	0.0365759	1.2162637	1.0122973	1.461327
CCR2	0.0257716	1.1846446	1.0207054	1.3749148
CUEDC1	0.0122557	1.4257976	1.0802025	1.8819609
SLC8A1	0.0153197	1.3269974	1.0557268	1.6679714
KIAA0513	0.0474948	1.2679092	1.002626	1.6033833
IRF8	0.0077443	1.2718492	1.0655447	1.5180972
TFEB	0.0081675	1.4482038	1.100669	1.9054724
DOK2	0.0314916	1.3186144	1.0248447	1.6965926
GPR65	0.0406375	1.3715321	1.0135549	1.8559431
GIMAP1	0.0348198	1.409455	1.0247737	1.9385386
CD163	0.0320764	1.1568871	1.0125538	1.3217941
MAP2K1	0.0364398	1.6716057	1.0329555	2.7051172
MSR1	0.000743	1.4353411	1.1634777	1.7707293
ABI3	0.021171	1.2950438	1.0394523	1.6134829
KMO	0.0025142	1.8876276	1.2500984	2.850286
SLC31A1	0.0431743	1.6211051	1.0149371	2.589305
SH2B3	0.0339599	1.7028355	1.0411328	2.7850901
OTUD1	0.0238068	1.5893163	1.063483	2.3751451
PIK3R5	0.0263907	1.5474195	1.052545	2.274969
CACNA2D3	0.0146745	1.3464744	1.0603062	1.7098772
MYO7A	0.0012004	1.4604031	1.1612925	1.8365546
EHBP1L1	0.0306813	1.5677751	1.0427542	2.3571411
GNGT2	0.0015095	1.7770003	1.2457985	2.5347037
PDK4	0.0330099	1.2314662	1.0169392	1.4912485
ST14	0.0041284	1.3276604	1.0938955	1.6113806
FTH1	0.0186236	1.5193223	1.0723606	2.1525785
SLA	0.0282612	1.4501518	1.0403862	2.0213073
IL10	0.0004251	1.5717409	1.222243	2.0211769
CLCN5	0.0007159	1.7341122	1.2606166	2.3854556
CPVL	0.0150637	1.2383713	1.0422843	1.4713485
MRVI1	0.0274625	1.492353	1.0454746	2.1302454
IQSEC1	0.0399479	1.3897903	1.015228	1.9025453
PMM1	0.0368434	1.5155276	1.0257255	2.239219
FOLR2	0.0095997	1.3851835	1.082479	1.7725363
HFE	0.0103601	1.7629011	1.1428149	2.7194431
NEDD9	0.0209072	1.6601327	1.0797684	2.5524368
CORO1C	0.0107024	2.0373213	1.1795691	3.5188089
MCOLN1	0.0222703	1.6790901	1.0766576	2.6186076
VOPP1	0.0459019	1.4629806	1.0069483	2.1255434
KIAA0040	0.0123301	1.789046	1.134415	2.8214415
BCL2L11	0.0004392	1.9228876	1.3354773	2.7686707

C1QA	0.0456294	1.1566592	1.0028321	1.3340823
TRPV4	0.0002829	2.1623848	1.4259882	3.2790651
KCTD17	0.0283424	1.5312672	1.0462308	2.2411683
MYO1E	0.0122486	1.4528215	1.0846483	1.9459676
CRTAM	0.0088907	1.8356962	1.1645896	2.8935348
AGPAT3	0.0290139	1.5572067	1.0463463	2.3174857
IFIT2	0.0465474	1.3384079	1.0044664	1.7833704
HK1	0.0212256	1.6727088	1.0798341	2.591097
RASL11A	0.0018088	1.8238049	1.250348	2.6602708
ZBTB38	0.0101472	1.6511203	1.126532	2.4199919
RNF144B	0.0055102	1.5031895	1.1272407	2.004522
SASH1	0.0005663	1.6500188	1.24118	2.1935272
THBS1	0.0070464	1.230121	1.0580928	1.4301181
MX1	0.047178	1.229801	1.0025813	1.5085167
BAG3	0.0019712	1.563377	1.1780161	2.0748
PTK2	0.0031733	1.4380602	1.1297077	1.8305773
ZNF532	0.0009404	1.4789014	1.172856	1.8648064
KDM5B	0.0418495	0.7261481	0.5335507	0.9882679
MYB	0.0186839	0.6441775	0.4465102	0.9293507
KIAA1549	0.0140297	0.7046644	0.5329573	0.9316919
